# Non‐Covalent Interactions Enforce Conformation in Switchable and Water‐Soluble Diketopiperazine‐Pyridine Foldamers

**DOI:** 10.1002/anie.202307180

**Published:** 2023-07-21

**Authors:** Sinead McCann, William E. Roe, Hannah E. Agnew, Peter C. Knipe

**Affiliations:** ^1^ School of Chemistry and Chemical Engineering Queen's University Belfast David Keir Building, Stranmillis Road Belfast BT9 5AG UK

**Keywords:** Conformation, Crystallography, Foldamers, Molecular Switches, NMR Spectroscopy

## Abstract

To reach their potential as mimics of the dynamic molecules present in biological systems, foldamers must be designed to display stimulus‐responsive behavior. Here we report such a foldamer architecture based on alternating pyridine‐diketopiperazine linkers. Epimerization is conveniently prevented through a copper‐catalyzed coupling protocol. The compounds’ native unswitched conformation is first discovered in the solid and solution state. The foldamers can be solubilized in DMSO and pH 9.5 buffer, retaining conformational control to a large degree. Lastly, dynamic switching is demonstrated through treatment with acid, leading to behaviour we describe as stimulus‐responsive sidechain reconfiguration.

## Graphical abstract

This study introduces a diketopiperazine‐based foldamer design that maintains its conformation in challenging environments, making it a promising platform for future therapeutic applications. The conformation is enforced by juxtaposition of dipolar repulsion and torsional strain interactions. The foldamers show dynamic switching capabilities, responding to external stimuli and paving the way for new applications in mimicking biological systems.

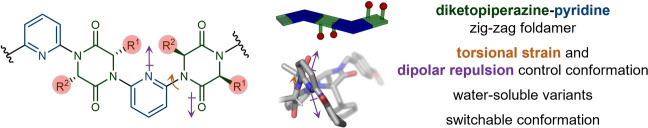

The well‐defined conformations of Nature's oligomeric biomacromolecules have inspired chemists to develop unnatural analogues—foldamers.^[1]^ The interactions between biomacromolecules (such as protein‐protein interactions) are crucial for many processes, but are also implicated in a large number of disease states.^[2]^ Foldamers hold great potential in disease prevention, since they may exhibit many of the properties of biomacromolecules whilst avoiding drawbacks such as proteolysis.^[3]^ A key feature in the design of foldamers is restriction of conformational freedom through non‐covalent interactions. These are often hydrogen bonds, but torsional strain, halogen bonding, metal templation, hydrophobic effects and more have been explored. However, the mimicry of static structures does not fully reflect the dynamic nature of biomacromolecules, where switching and allostery are crucial to function.^[4]^ As a result, switching within foldamers has been reported in response to diverse stimuli including light, solvent polarity and ion‐binding.[^1a^, ^5^]

In our previous studies dipolar repulsion between adjacent monomers was used as the primary determinant of conformation, specifically between azenes and imidazolidine‐2‐one^[6]^ and spirocyclic bis‐lactam^[7]^ linkers. Related systems undergo acid‐mediated switching between an extended and helical conformation.[^5k^, ^5o^] We reasoned that through judicious design, a switching foldamer might be devised where the overall backbone conformation is the same in both switched states, but the facial display of sidechains is altered (Figure 1A). Here, switching of one functional group (pink⇌blue) converts a repulsive interaction between functionalities (pink↔yellow) into an attractive one (blue↔yellow). In a *para*,*meta*‐linked copolymer this is hypothesized to allow switching between two zig‐zag conformers, with the facial display of the *para*‐linking groups the only significant change (maroon⇌green).

**Figure 1:**
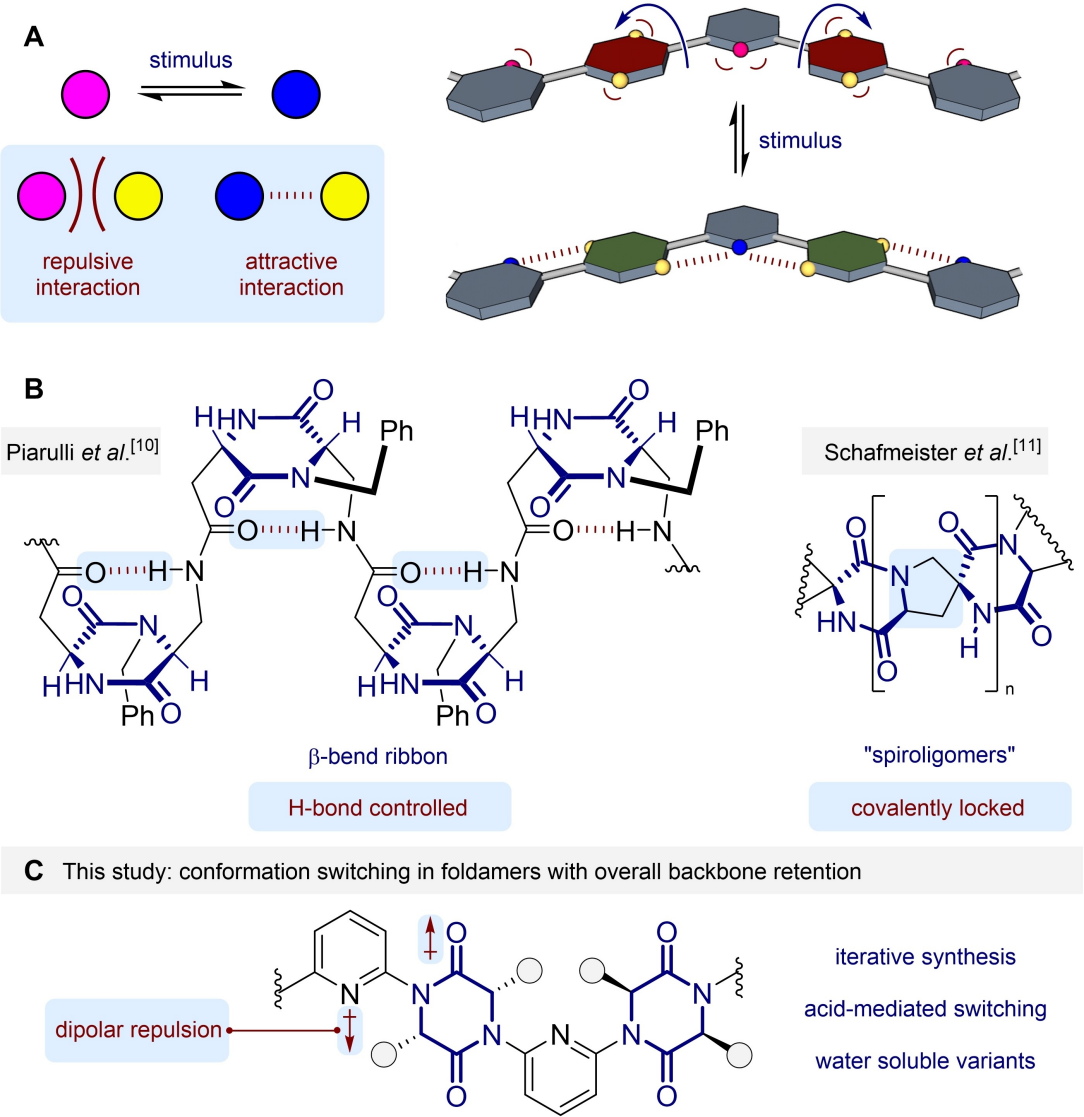
A. Concept for switching with retention of backbone conformation. Blue arrows indicate the rotation of *para*‐linking groups expected upon applying the stimulus. B. Previous DKP‐containing foldamers and related molecules. C. Hypothesised foldamer comprising alternating DKP and pyridine linkers. Blue boxes highlight the factors controlling overall molecular shape.

We considered that the diketopiperazine (DKP) motif should exhibit similar dipolar preferences to our previous scaffolds, and could serve as the *para*‐linking group. Diketopiperazines have previously been investigated in the context of foldamers,^[8]^ most notably as artificial β‐turn mimetics. For example, Gennari, Piarulli et al. deployed a diketopiperazine to induce β‐hairpin formation in a series of hexapeptides.^[9]^ Subsequently, Piarulli reported oligomers formed from the same DKP, which adopt a β‐bend ribbon conformation in CD_3_OH, stabilized by inter‐residue 10‐membered ring hydrogen bonds (Figure 1B).^[10]^ In an approach complementary to foldamers, Schafmeister et al. formed a series of covalently stabilized “spiroligomers” that were applied as catalysts and therapeutic agents.^[11]^ We sought to develop a new foldamer where juxtaposition of diketopiperazines with adjacent azenes would lead to conformational control about the C−N bond. Based upon our prior studies we expected that dipolar repulsion would favour an *anti*‐arrangement between the amide groups and adjacent azene nitrogens (Figure 1C). Protonation of the pyridines would subvert this interaction and lead to the manner of switching outlined in Figure 1A. We have utilized the principal axes of aviation to convey the conformational effects of monomers within foldamers.^[7]^ Here, we anticipate that the substitution pattern on the azene linker and dipolar repulsion effects will achieve control about both the yaw and roll axes.

An iterative synthetic approach to the sequence‐defined foldamers necessitated end group differentiated DKP precursors. These were formed from commercially available amino acids (Scheme 1): *N*‐PMB protection of alanine methyl ester was followed by coupling with *N*‐protected amino acids. Both Boc and Fmoc protected amino acids were tolerated; the latter was particularly valuable since it allowed the inclusion of an acid‐labile protected sidechain (CO_2_ *t*‐Bu). The resulting dipeptides **1 a**, **b** were cyclized, with **1 a** requiring initial treatment with acid to remove the Boc protecting group followed by basic conditions to neutralise the resulting TFA salt and enable lactamisation forming **2 a**. **1 b** underwent both deprotection and spontaneous cyclization under basic conditions to afford DKP **2 b**. The *N_Ala_*‐PMB DKPs thus obtained were reacted with 2,6‐dibromopyridine under Pd‐catalyzed Buchwald–Hartwig conditions to afford monomers **3 a** and **3 b** in 73 % and 67 % yield respectively.

**Scheme 1:**
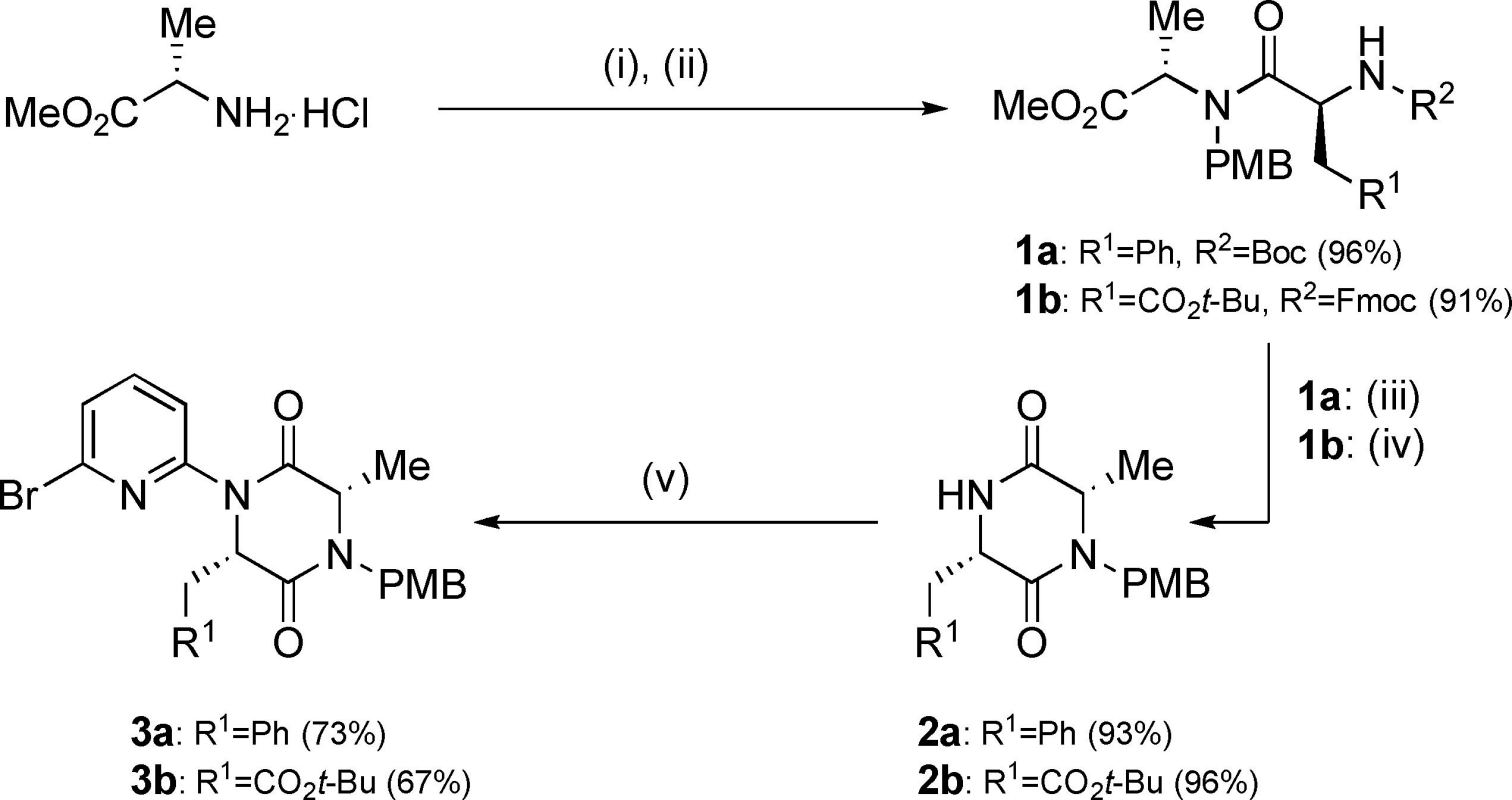
Synthesis of monomers **3 a**,**b** from amino acid precursors. (i) 4‐methoxybenzaldehyde, Et_3_N, MeOH then NaBH_4,_ 75 %; (ii) **1 a**: Boc‐Phe‐OH, HATU, DIPEA, CH_2_Cl_2_, 96 %; **1 b**: Fmoc‐Asp(O*t*‐Bu)‐OH, HATU, DIPEA, CH_2_Cl_2_, 91 %; (iii) TFA, CH_2_Cl_2_, then NaHCO_3_ (sat. aq.) 93 %; (iv) piperidine:CH_2_Cl_2_ (1 : 4 *v*:*v*), 96 %; (v) 2,6‐dibromopyridine (5 equiv.), Pd_2_dba_3_ (5 mol%), Xantphos (15 mol%), Cs_2_CO_3_, PhMe, reflux, 4–6 h, **3 a**: 73 %, **3 b**: 67 %.

With this key intermediate in‐hand, we embarked on constructing an oligomer using an iterative coupling‐deprotection approach (Scheme 2A). Treatment of **3 a** with 2‐pyrrolidinone effectively “capped” the monomer, allowing the foldamer to be grown from the *N*‐PMB terminus. Single crystal X‐ray diffraction of **4** revealed a conformation consistent with our dipole opposition hypothesis.^[12]^ Deprotection of the PMB group was achieved by treatment with CAN, affording **5** in 61 % yield. Subsequent iterative cycles of cross‐coupling and deprotection allowed access to dimer **6** and trimer **8** and their *N*‐deprotected derivatives **7** and **9**.

**Scheme 2:**
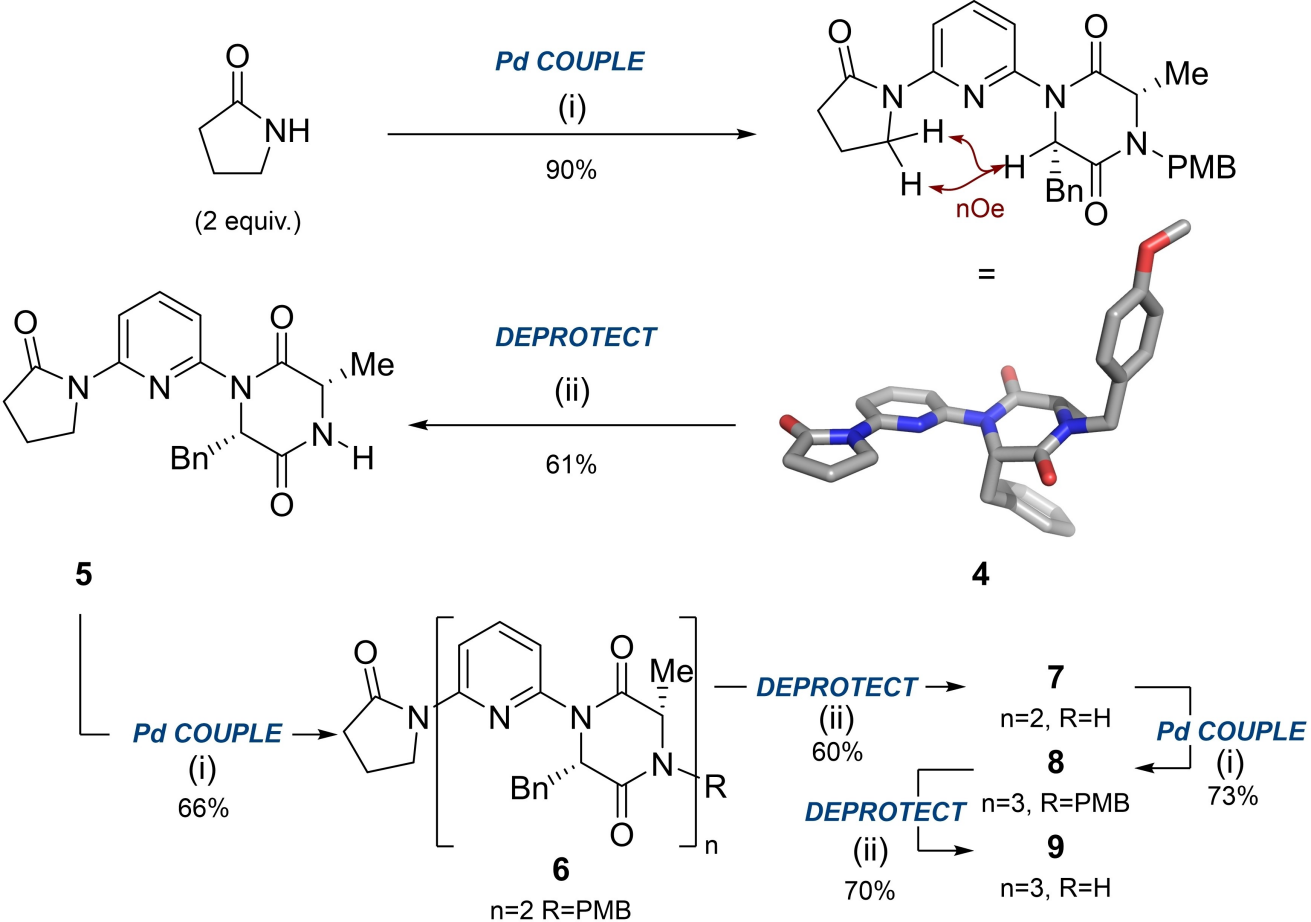
A. Coupling and deprotection steps to form dimeric and trimeric foldamers and single crystal x‐ray structure of **4** (CCDC 2258873) displaying the expected conformation as determined by dipolar repulsion between the DKP carbonyl and pyridine nitrogen. Displacement ellipsoids are drawn at the 50 % probability level. nOe cross‐peaks suggesting this conformation is also adopted in solution are indicated in maroon. (i) Monomer **3 a**, Pd_2_dba_3_ (5 mol%), Xantphos (15 mol%), Cs_2_CO_3_, PhMe, reflux, 2–4.5 h, **4** (90 %), **6** (66 %), **8** (73 %); (ii) CAN (4 equiv.), MeCN:H_2_O, 22 h, 0–60 °C, **5** (61 %), **7** (60 %), **9** (70 %). See Supporting Information for complete experimental details.

Surprisingly, attempts to form the analogous homo‐oligomers of aspartic acid‐derived monomer **3 b** were hampered by epimerisation, something that had not been observed for the phenylalanine/alanine series (Scheme 3A). When monomer **3 b** was treated with 2‐pyrrolidinone under the standard Pd‐catalysed conditions, the product **10** was obtained as a 3 : 1 mixture of epimers. Boyd and Sperry observed epimerisation of DKPs during Pd‐catalysed *N*‐arylation with Xantphos, but found that XPhos led to improved reactivity and reduced epimerisation.^[13]^ In our case, however, the epimerization persisted regardless of ligand choice (see supplementary data). Pleasingly, switching to Goldberg coupling conditions (CuI, DMEDA) afforded the product **10** as a single diastereoisomer. Rajanbabu has previously reported the superiority of copper vs. palladium‐catalysed *N*‐arylation of diketopiperazines in avoiding epimerization.^[14]^ In the context of these foldamers, it is our suggestion therefore that these conditions be adopted in instances where epimerisation is observed. Deprotection of **10** under the standard oxidative conditions afforded **11** in 83 % yield, which was subsequently reacted with **3 b** under Goldberg conditions to form dimer **12** in 64 % and with no observable epimerisation (Scheme 3B). Lastly, deprotection of the sidechains upon treatment with TFA afforded diacid **13** in 84 % yield.

**Scheme 3:**
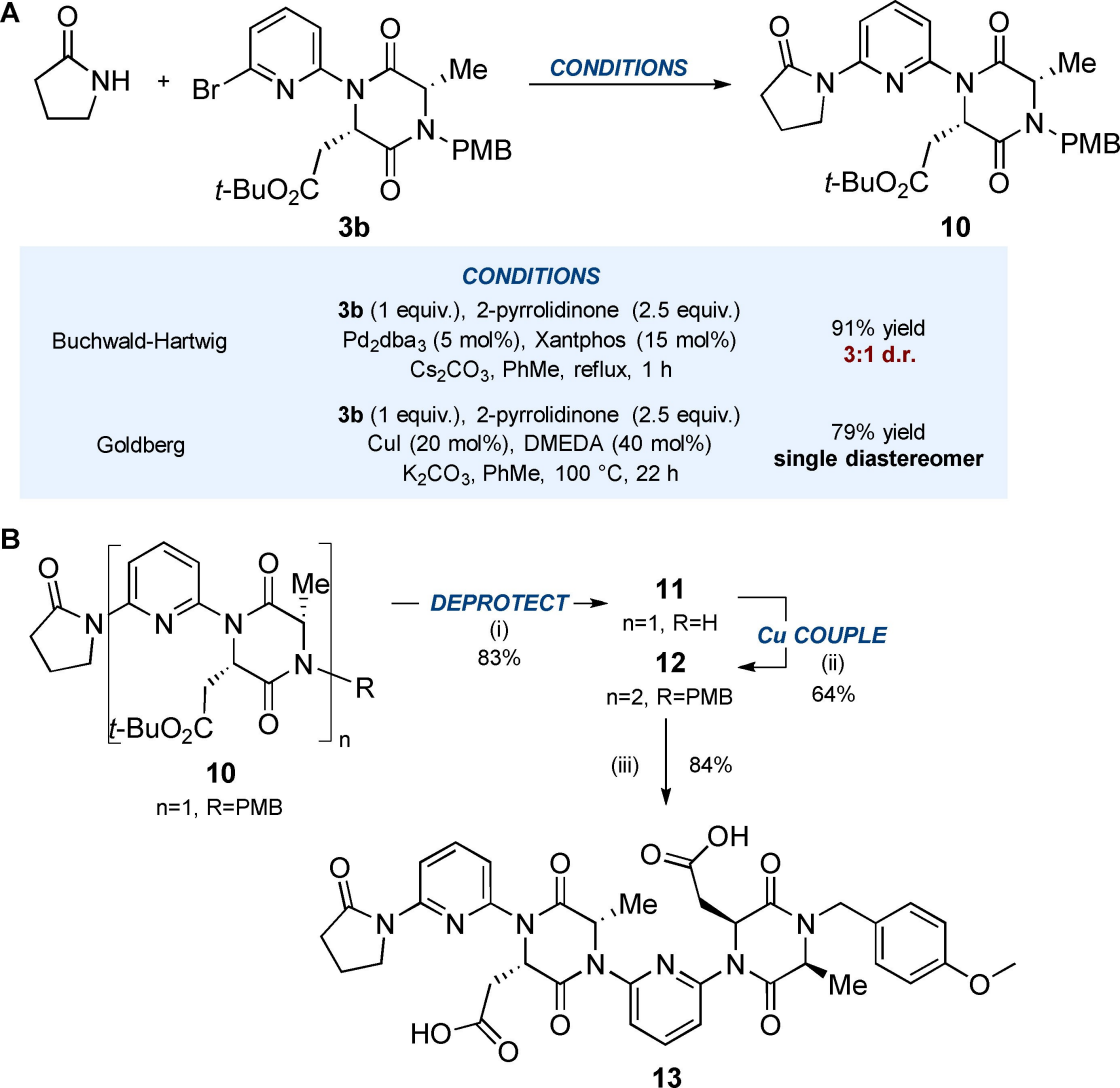
A. Epimerization observed in Pd‐catalysed cross‐coupling of monomer **3 b** with 2‐pyrrolidinone. The formation of epimers became even more severe when Pd catalysis was attempted in the synthesis of dimer **12** from deprotected monomer **11**, see Supporting Information. B. Synthesis of water soluble diacid foldamer **13** using Goldberg conditions. (i) CAN (4 equiv.), MeCN:H_2_O (3 : 1 *v*:*v*), 0 °C, 2.5 h, 83 %; (ii) Monomer **3 b** (1.1 equiv.), CuI (20 mol%), DMEDA (40 mol%), K_2_CO_3_, PhMe, 100 °C, 22 h, 64 %; (iii) TFA:CH_2_Cl_2_ (1 : 1 *v*:*v*), rt, 4 h, 84 %.

Single crystals of dimer **6** allowed its native conformation to be examined in detail prior to switching (Figure 2A).^[12]^ Inspection of the structure confirmed its adherence to the dipole repulsion hypothesis: in all cases lactams adjacent to pyridine linkers adopt a conformation where the carbonyl is *anti* to the pyridine nitrogen. In contrast with our earlier studies,[^6^, ^7^] the plane of the aliphatic linker is significantly twisted relative to the pyridine. Without exception this torsion places the C_α_H in the “inside” position, likely to minimise torsional strain between the side‐chain and pyridine. The effect is that there is a left‐handed (*M*)‐helical twist in the backbone at each C_pyr_−N_DKP_ linkage. Evidence for this conformational preference was also gained in the solution state via ^1^H‐ROESY experiments in CDCl_3_ (Figure 2B). Strong enhancements were observed between C_α_−H hydrogens on adjacent DKP monomers, while these same hydrogens displayed no cross‐peaks with the *meta*‐CH on the adjacent pyridines. Strong enhancements between *ortho*‐CH hydrogens on the benzyl sidechains and the pyridine *meta*‐CH positions were observed. These observations are consistent with the distances observed in the crystal structure. Trimer **8** displayed broadly the same features in its ROESY spectrum, but its greater complexity meant enhancements could not be fully assigned (see Supporting Information). Rotation is likely facile around the C_pyr_‐N_DKP_ bonds in the solution state, but these nOe data are consistent with the ensemble of conformers being biased towards a conformation where the DKP carbonyls are *anti* to the pyridine nitrogen atoms, and the C_α_−H hydrogens positioned “inside”. The inter‐DKP resonances between C_α_−H hydrogens are particularly compelling since they are consistent with simultaneous biasing of the conformation around two C_pyr_‐N_DKP_ bonds. The general agreement between the solid‐ and solution‐state conformation of the foldamers (in CDCl_3_) is additionally supported through infra‐red spectroscopy experiments (see Supporting Information).

**Figure 2:**
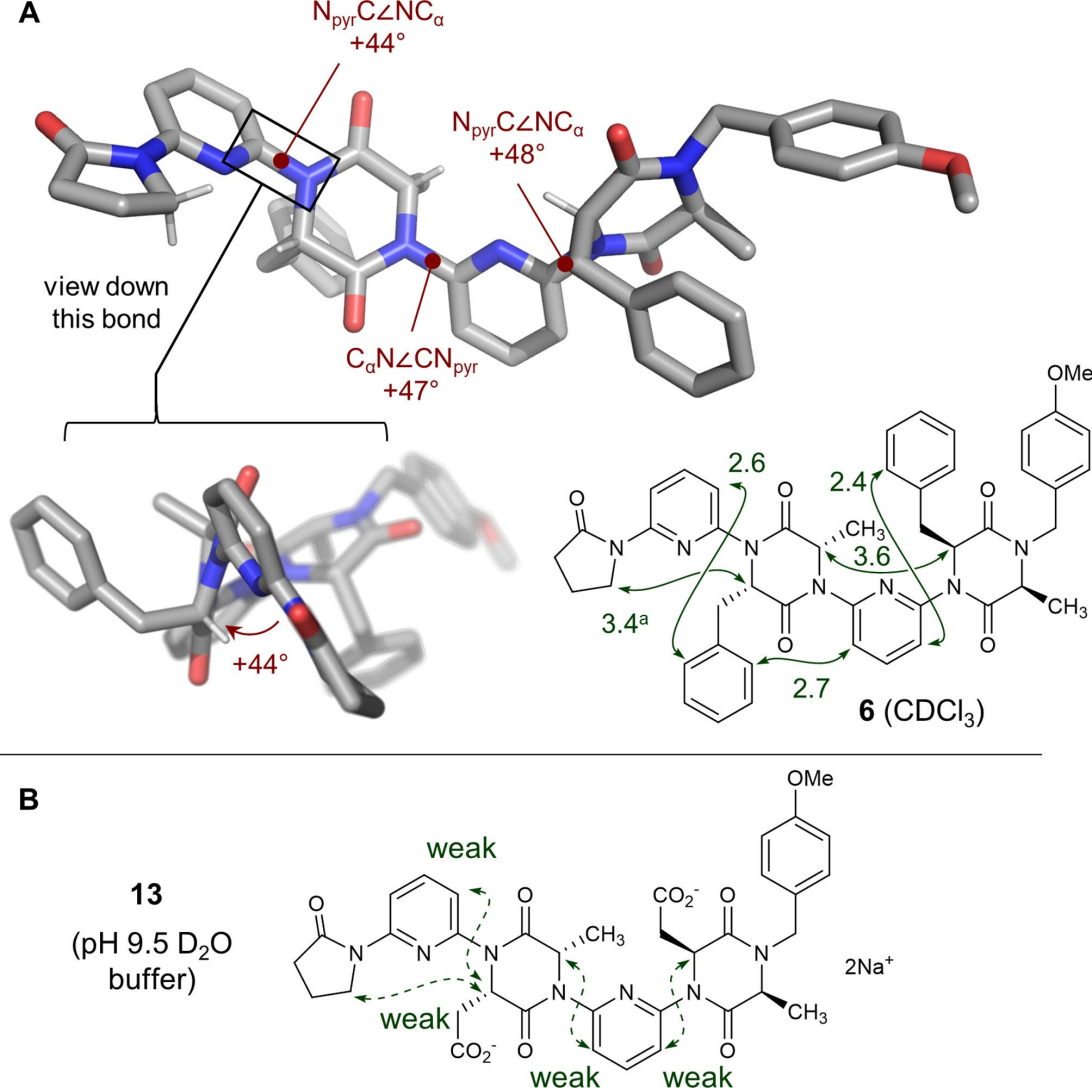
A. Single crystal X‐ray structure of dimer **6** (CCDC 2258872). Key dihedral angles are indicated in maroon. Bottom right: key ROESY correlations (in green) observed for **6** (600 MHz, CDCl_3_, *t_mix_* 0.2 s). The corresponding distances measured from the single crystal data are provided (in Å). B. Key ROESY correlations observed for **13** (400 MHz, D_2_O, *t_mix_* 0.2 s). ^a^ The average distance to the diastereotopic methylene hydrogens is given.

Diacid **13** displayed similar features to **6** in CDCl_3_ and *d_6_*‐DMSO (see Supporting Information). Though insoluble at neutral pH, diacid **13** dissolved in a pH 9.5 buffer solution (NaDCO_3_/Na_2_CO_3_ in D_2_O), allowing its conformation to be examined in an aqueous medium. This indicates partial loss of conformational rigidity: the nOe observed between C_α_−H hydrogens in adjacent DKP monomers observed in CDCl_3_ above is absent (though a weak correlation between the terminal 2‐pyrrolidinone and adjacent DKP remains). These same hydrogens also now display weak cross‐peaks with the *meta*‐CH on the adjacent pyridines. These data are consistent with increased rotation around the C_pyr_‐N_DKP_, but with retention of the bias towards a dipole‐opposed conformation. The weak nature of these nOes sits in contrast with the strong nOes observed in the protonated and fully switched [(*syn*,*syn*)‐**6** ⋅ 2H]^2+^ (see following section), so is inconsistent with a complete conformational change to a *syn* arrangement between DKP carbonyls and adjacent pyridines. We reasoned that (i) Lewis basic interactions with Na^+^; (ii) an increased solvent dipole moment; and (iii) solvent‐foldamer hydrogen bonding may all contribute to the observed partial reduction in conformational control. To determine if (i) was a significant factor, **13** was dissolved in D_2_O containing 2 equivalents of tetrabutylammonium hydroxide (TBAOH) such that the TBA cation would serve as a non‐coordinating counter‐ion. The ROESY data obtained were nearly identical to those observed in NaDCO_3_/Na_2_CO_3_ buffer, suggesting that metal templation is not a significant factor in the conformational changes observed. However, the fact that some control is maintained even in such a challenging solvent environment is promising for future applications of these materials in vitro.

To test our initial hypothesis of a switchable foldamer in which the overall backbone conformation is retained, we examined the response of these molecules to the presence of Bronsted acids (Figure 3). We reasoned that upon treatment with acid the weakly basic pyridine linkers would become protonated. The presence of an adjacent N^+^−H hydrogen bond donor to the DKP carbonyls was expected to overturn the dipolar repulsion effect to facilitate formation of the highly favourable 6‐membered ring hydrogen‐bond.^[5k]^ The primary indicator of conformation used in this study was the nOe enhancement observed between the pyridine *meta*‐C−H and the adjacent C_α_−H on the DKP. As discussed above, this nOe is absent when the dipole opposed conformation is adopted in CDCl_3_. Dimer **6** was treated with an excess of *d*‐TFA, causing large chemical shifts in H^2^, H^3^ and H^4^ suggesting pyridine 1 had undergone protonation. Two strong nOe enhancements (H^1^↔H^2^, H^4^↔H^5^) suggest a conformational change had occurred, with the ensemble of conformers now biased towards the two amides flanking pyridine 1 adopting a *syn* conformation relative to the pyridine nitrogen ([(*syn*,*anti*)‐**6** ⋅ H]^+^). Further treatment with TfOH led to the same changes around pyridine 2: H^7^‐H^9^ underwent a large change in chemical shift, while strong H^6^↔H^7^ and H^9^↔H^10^ nOes were observed. These results are consistent with protonation of both pyridines, with a further biasing in the ensemble of conformers towards [(*syn*,*syn*)‐**6** ⋅ 2H]^2+^ having occurred. Pyridine 2′s reduced basicity relative to pyridine 1 may be a result of the increased torsional strain required to adopt the hydrogen bond‐stabilised conformation, since pyridine 1 is flanked by a 2‐pyrrolidinone and a DKP while pyridine 2 is flanked by two DKPs. In longer oligomers it is likely that all the non‐terminal pyridines would have approximately equal basicities, such that there would only ever be three well‐defined switched states: neutral, mono‐protonated on pyridine 1, and fully protonated on all pyridines. In agreement with our design, the overall backbone conformation of [(*syn*,*syn*)‐**6** ⋅ 2H]^2+^ is the same as that of the neutral precursor (*anti*,*anti*)‐**6**, but with the sidechains occupying different positions. We describe this behaviour as *stimulus‐responsive sidechain reconfiguration*. The switching is fully reversible: treatment of **6** ⋅ (TfOH)_2_ with NaHCO_3_ led to complete recovery of neutral dimer **6** with no epimerization observed.^[15]^ However, attempts to achieve demonstrate the same switching effect in a more polar DMSO solvent were unsuccessful, likely due to protonation of the solvent itself. Dimer **13** bearing functional acid sidechains displayed analogous switching behaviour upon titration with TFA and TfOH (see Supporting Information). The acid‐mediated switching of trimer **8** has also been examined and is consistent with the behaviour of dimer **6**, though the complexity of spectra preclude unambiguous assignment of specific conformationally relevant nOes (see Supporting Information).

**Figure 3:**
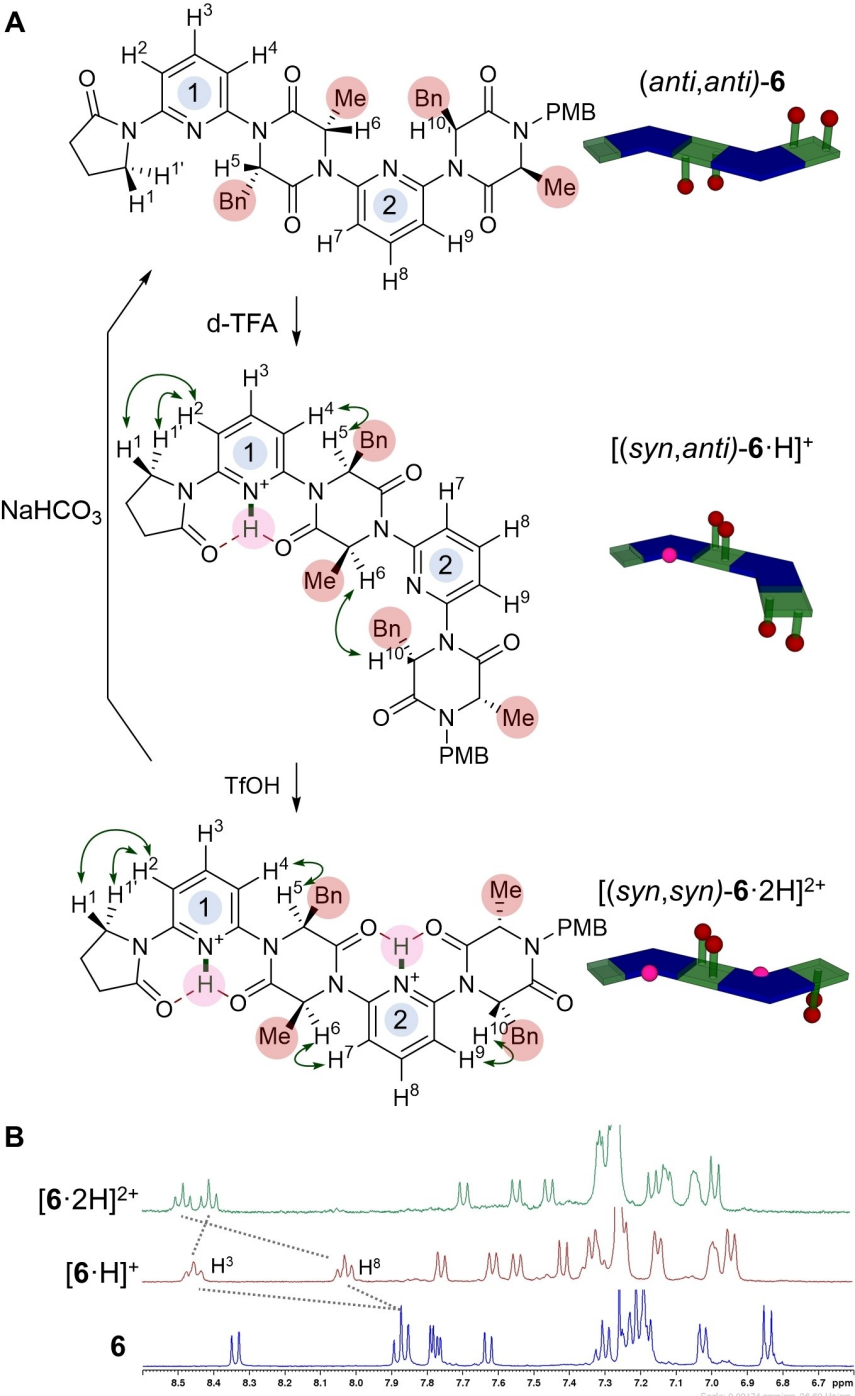
A. Acid‐mediated three‐state switching behaviour of dimer **6**. Selected ROESY cross‐peaks (600 MHz, CDCl_3_, *t_mix_* 0.2 s) indicative of overall conformation are indicated (green arrows). Cartoons indicate overall conformation of foldamer and disposition of sidechains (pyridine—blue chevrons; DKP—green squares; sidechains—red spheres; protons—pink spheres).^[15]^ B. ^1^H NMR spectra of the aromatic region during acid titration of dimer **6**. The pyridine *para*‐hydrogens are highlighted and are diagnostic of which pyridine is protonated at each stage.

To summarise, an iteratively synthesised foldamer was formed comprising amino acid‐derived diketopiperazine and pyridine linkers, with the ground state conformation controlled by a combination of dipolar repulsion and torsional strain. This conformational control was sufficiently robust to be retained even in highly competitive and polar aqueous media, suggesting that these and related compounds may find applications in biological systems, such as protein‐protein interaction inhibition. Upon treatment with an acid stimulus the foldamers undergo multi‐state switching, such that the neutral and exhaustively protonated structures adopt the same backbone conformation with an alternative presentation of sidechains.

## Conflict of interest

The authors declare no conflict of interest.

## Supporting information

As a service to our authors and readers, this journal provides supporting information supplied by the authors. Such materials are peer reviewed and may be re‐organized for online delivery, but are not copy‐edited or typeset. Technical support issues arising from supporting information (other than missing files) should be addressed to the authors.

Supporting Information

Supporting Information

Supporting Information

## Data Availability

The data that support the findings of this study are available in the supplementary material of this article.
