# Supplementary material for: Non‐Covalent Interactions Enforce Conformation in Switchable and Water‐Soluble Diketopiperazine‐Pyridine Foldamers
Source: Angew Chem Int Ed Engl. 2023 Jul 21;62(35):e202307180. doi: 10.1002/anie.202307180 (PMC10952507; doi:10.1002/anie.202307180)
Supplement: Supplementary file 3 — Supporting Information [file ANIE-62-0-s002.pdf]

Angewandte Chemie
Eine Zeitschrift der Gesellschaft Deutscher Chemiker

Supporting Information

Non-Covalent Interactions Enforce Conformation in Switchable and Water-Soluble Diketopiperazine-Pyridine Foldamers

*S. McCann, W. E. Roe, H. E. Agnew, P. C. Knipe**

Supplementary Information

General Experimental 4
1.1 Naming and Numbering of Compounds 4
1.2 Solvents and Reagents 4
1.3 Purification 4
1.4 Spectroscopy 4
1.5 Mass Spectrometry 5
1.6 Crystallography 5
2 Experimental Procedures and Characterisation Data 6
2.1 General Procedures 6
2.2 Diketopiperazine Synthesis 8
2.3 Foldamer Synthesis 14
2.3.1 Monomers 14
2.3.2 Dimers 21
2.3.3 Trimers 25
3 Solution-Phase Conformational Analysis by NMR 29
3.1 General Comments 29
3.2 Monomer 4 29
3.3 Dimer 6 32

3.4 Trimer 8 39
4 Solution-Phase Conformation of Carboxylic Acid Bearing DKPs 43
4.1 General Comments 43
4.2 Monomer 10a 43
4.3 Dimer 13 47
4.3.1 d_6-DMSO and D_2O Influences on Conformational Control for Diacid 13 50
5 Acid-Mediated Conformational Change 59
5.1 Dimer 6 Acid-Switching Experiment 59
5.1.1 Neutralisation/Reversal of the Conformationally Switched Dimer 6 62
5.2 Dimer 6 Acid-Switching Conformational Control Spectra 65
5.3 Attempted Acid-Mediated Conformational Switching of Dimer 6 in *d6*-DMSO 73
5.4 Monomer 4 Acid-Switching Experiment and Conformational Control Spectra 76
5.5 Dimer 13 Acid-Switching Experiment 80
5.6 Dimer 13 Acid-Switching Conformational Control Spectra 82
5.7 Trimer 8 Acid-Switching Experiment 89
5.7.1 Neutralisation/Reversal of the Conformationally Switched Trimer 8 91
5.8 Trimer 8 Acid-Switching Conformational Control Spectra 93
6 Further Discussion on the Issue of Epimerisation of 10 102
7 Infrared Spectroscopy 107
7.1 IR Spectra of Neutral Foldamers 107
7.1.1 IR of Monomer 4 108
7.1.2 IR of Dimer 6 109
7.1.3 IR of Dimer 13 110
7.1.4 IR of trimer 8 111
7.2 Attempts to Obtain IR Spectra of Protonated Foldamers 111
7.2.1 Solid-Phase 111
7.2.2 Solution-Phase 112
8 X-ray Crystallography 114
8.1 Single Crystal Data for 4 (CCDC 2258873) 114

8.2 Single Crystal Data for 6 (CCDC 2258872) 116
9 NMR Spectra 118
10 References 157

General Experimental

1.1 Naming and Numbering of Compounds

Systematic compound names are those generated by ChemBioDraw™ Ultra version 15.1.0.144 (Perkin Elmer) following IUPAC nomenclature.

1.2 Solvents and Reagents

Unless stated otherwise, all other solvents and reagents used were directly obtained from commercial sources. $Pd_2(dba)_3$ was recrystalized from Acetone/$CHCl_3$.[1]

Due to the air and moisture sensitive nature of several of the reactions, glassware was oven-dried prior to use and the reaction performed under an inert (argon) atmosphere, with solvents being dried over 3 Å molecular sieves prior to their use. Anhydrous solvents were degassed with argon for 15 min immediately prior to use.

1.3 Purification

Flash column chromatography was carried out using Fluorochem 60 40-63 micron silica gel. Thin-layer chromatography was carried out using Merck Kieselgel 60 F254 (230-400 mesh) fluorescent treated silica, visualized under UV light (254 nm) or by staining with aqueous potassium permanganate solution, ninhydrin or ceric ammonium molybdate solutions.

1.4 Spectroscopy

^{1}H, and ^{13}C NMR spectra were obtained using Bruker 600, or 400 MHz spectrometers using $CDCl_3$, d_6-DMSO or D_2O as the solvent and the residual non-deuteriated solvent as the internal reference. To analyse and process the NMR spectra, TopSpin™ software was used, and spectra were calibrated against residual non-deuteriated solvent peaks as internal standards. The chemical shifts are reported in parts per million (ppm) and coupling constants (*J*) are reported in Hertz (Hz). ^{1}H NMR spectra are reported as follows: δ/ppm (number of protons, multiplicity, coupling constant, assignment of peak (if possible)). The multiplicities are abbreviated as follows: s = singlet, d = doublet, t = triplet, q = quartet, m = multiplet. ^{13}C NMR spectra are reported as follows: δ/ppm (assignment). Chemical structures are numbered arbitrarily for the purpose of assignment; this numbering scheme does not necessarily correspond with the systematic name of the compound. Two-dimensional NMR experiments (COSY, HSQC, HMBC, NOESY, ROESY) were also recorded when necessary to help aid assignment of the proton and carbon peaks.

Infra-red (IR) spectra were recorded on an Agilent Cary 630 spectrometer. Samples were analysed by two methods:

1. An attenuated total reflectance (ATR) accessory. Samples were deposited on the ATR as a thin film or neat solid.
2. A DialPath accessory for the solution phase. Samples were deposited as a 0.1 M solution in $CHCl_3$, with a background scan of $CHCl_3$ run before each sample to minimise absorbances resulting from $CHCl_3$.

Only selected maximum absorbances (ν_{max}) of the most intense peaks are reported (cm^{-1}).

Optical rotations were recorded at the sodium D-line (589 nm) using a Perkin Elmer 341 polarimeter at a temperature of 20 °C and are reported in degrees using concentrations (*c*) in g.100 mL^{-1}. Reported values are the average of eight readings.

1.5 Mass Spectrometry

Liquid chromatography-mass spectrometry (LCMS) analyses were conducted using an instrument comprising an Agilent 1260 HPLC (equipped with Infinity II quaternary pump, vial sampler, integrated column compartment and variable wavelength detector) and MSD single quadrupole mass spectrometer. Samples were analysed using an Agilent Infinitylab poroshell 120 column (2.7 µm, 2.1 x 150 mm) under an acetonitrile/water gradient with 0.1% HCOOH additive.

High resolution mass spectra (HRMS) were recorded by Analytical Services and Environmental Projects (ASEP) at Queen's University Belfast on a Waters LCT Premier ToF mass spectrometer using the electrospray ionisation (ESI) technique.

1.6 Crystallography

Low temperature[2] single crystal X-ray diffraction studies were carried out using CuK_{α} radiation on an Agilent Supernova diffractometer equipped with an area detector and graphite monochromator. Raw frame data were reduced using CrysAlisPRO[3] solved using SHELXT.[4] Full-matrix least-squares refinement of the structures were carried out using CRYSTALS.[5,6] Full refinement details are given in the supplementary material (CIF). CCDC 2258872-2258873 contain the supplementary crystallographic data for this paper. These data are provided free of charge by The Cambridge Crystallographic Data Centre and copies can be obtained free of charge *via* www.ccdc.cam.ac.uk/data_request/cif.

2 Experimental Procedures and Characterisation Data

2.1 General Procedures

General Procedure A: Palladium-Catalysed Coupling of Deprotected diketopiperazines with Aryl Halides

This reaction was carried out by analogy to a literature procedure.[7] To a sealed-tube under an inert atmosphere of argon and equipped with a magnetic stir bar, was added deprotected diketopiperazine (1.0 equiv.), aryl halide (1.1-5.0 equiv.), freshly recrystallized $Pd_2(dba)_3$ (5 mol%) Xantphos (15 mol%) and Cs_2CO_3 (2.5 equiv.). Anhydrous toluene (ca. 0.1 M) was added to the flask, and the resulting suspension was then simultaneously sonicated and de-gassed by sparging with argon gas for 15-30 min. Following this the reaction mixture was then heated at the specified temperature until all the diketopiperazine starting material was consumed. After complete consumption of the diketopiperazine starting material by TLC analysis, the reaction was cooled to room temperature, diluted with dichloromethane (ca. 20 mL/mmol deprotected diketopiperazine) and filtered over Celite®, which was also then washed with EtOAc. The crude product was then purified by flash column chromatography.

General Procedure B: Copper-Catalysed Coupling of Deprotected diketopiperazines with Aryl Halides

This reaction was carried out by analogy to a literature procedure by Shibata *et al.*[8] To a sealed-tube under an inert atmosphere of argon and equipped with a magnetic stir bar, was added deprotected diketopiperazine (1.0 equiv.), aryl halide (1.1 equiv.), CuI (20 mol%) DMEDA (40 mol%) and K_2CO_3 (2.0 equiv.). Anhydrous toluene (ca. 0.1 M) was added to the flask, and the resulting suspension was then simultaneously sonicated and de-gassed by sparging with argon gas for 15-30 min. Following this the reaction mixture was then heated at the specified temperature until all the diketopiperazine starting material was consumed. After complete consumption of the diketopiperazine starting material by TLC analysis, the reaction was cooled to room temperature, diluted with dichloromethane (ca. 20 mL/mmol deprotected diketopiperazine) and filtered over Celite®, and washed through with DCM. The crude product was then purified by flash column chromatography.

*Note: as discussed in **Section 6**, the quality/purity of the CuI used significantly affects the reactivity/success of the cross-coupling and therefore it is vital to use high-quality/pure material to achieve optimal cross-coupling.*

General Procedure C: PMB Deprotection

This reaction was carried out with slight modification of Yoshimur *et al.* conditions.[9] To a flask equipped with a magnetic stir bar was added *N*-PMB diketopiperazine (1.0 equiv.) and MeCN. The solution was cooled to 0 °C and an aqueous solution of ceric ammonium nitrate (4 equiv., 1.0 M in H_2O) added to the reaction mixture dropwise. The reaction mixture was then stirred at the specified temperature until all the diketopiperazine starting material was consumed by TLC analysis. Upon completion of the reaction either solid or saturated aqueous sodium bicarbonate was added and stirred for 5-10 min, before filtering through celite. The solution was then diluted with brine, transferred to a separatory funnel, and the layers separated. The aqueous layer then washed with EtOAc (2 x 10 mL per mmol of SM). The combined organic layers were dried over $MgSO_4$, filtered under gravity, and concentrated in vacuo. The crude product was purified by flash column chromatography.

Note: due to solubility of the DKP foldamers changing upon elongation, the ratio of MeCN: H_2O increases with length (3:1 for the monomer and 100:1 for the dimer/trimer). With the trimer, it also required heating to 60 °C to ensure full dissolution.

2.2 Diketopiperazine Synthesis

Methyl (4-methoxybenzyl)-*L*-alaninate (**S1**)

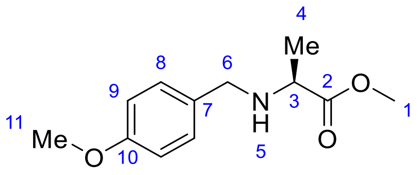

L-alanine methyl ester hydrochloride (5.0 g, 0.036 mol, 1 equiv.) was dissolved in anhydrous methanol (30 mL) under argon at room temperature. Triethylamine (5.0 mL, 0.036 mol, 1 equiv.) was added, and the solution was stirred for 15 min. 4-methoxybenzaldehyde (4.4 mL, 0.036 mol, 1 equiv.) was added and stirred for 2.5 h. Sodium borohydride (2.7 g, 0.072 mol, 2 equiv.) was then added portion wise over 30 min then left to stir for 30 min. The solution was partitioned between 20% HCl (2 mL/mmol of amino acid) and diethyl ether (2 mL/mmol amino acid). The organic phase was then extracted twice with 20% HCl (2 × 1 mL/mmol amino acid). The organic phase was discarded, and the combined aqueous layers were washed with diethyl ether (1 mL/mmol amino acid) before being neutralised to pH 7 using solid sodium carbonate. The resulting solution was extracted with diethyl ether three times (3 x 5 mL/mmol amino acid). The combined organic extracts were washed with brine, dried over $MgSO_4$ and filtered. The solution was concentrated *in vacuo* to afford the product as a yellow oil (6.0 g, 75%); δ_H (600 MHz, $CDCl_3$): 7.24 (2H, d, *J* 8.5 Hz, **H9**), 6.86 (2H, d, *J* 8.6 Hz, **H8**), 3.79 (3H, s, **H11**), 3.73 (3H, s, **H1**), 3.72 (1H, d, *J* 12.6 Hz, **H6'**), 3.61 (1H, d, *J* 12.6 Hz, **H6**), 3.38 (1H, q, *J* 7.0 Hz, **H3**), 1.83 (1H, s, **H5**), 1.31 (3H, d, *J* 7.0 Hz, **H4**); δ_C (151 MHz, $CDCl_3$): 176.4 (**C2**) 158.9 (**C10**), 132.0 (**C7**), 129.6 (**C9**), 113.9 (**C8**), 55.9 (C3**),** 55.4 (**C11**), 51.9 (**C1**), 51.5 (**C6**), 19.2 (**C4**); **HRMS** (ESI+):found 224.1241; $C_{12}H_{17}NO_3H$, $[M+H]^+$ requires 224.1281; $[\alpha]_D^{20}$ −33.3 (*c* = 1.85, $CHCl_3$) (lit. $[\alpha]_D$ −8.1 (*c* = 0.83, $CHCl_3$)); ν_{max} (neat): 2950, 2835, 1731, 1511, 1443, 1243, 1148, 1033 cm^{-1}. Characterisation data in agreement with literature data.[10,11]

Methyl (4-methoxybenzyl)-*L*-alaninate (**1a**)

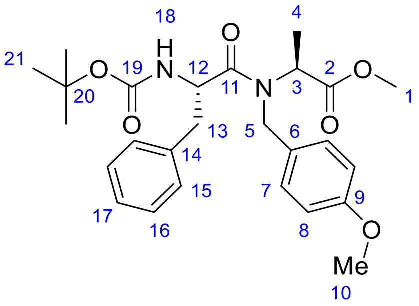

A solution of Boc-*L*-phenylalanine (1 g, 3.8 mmol, 1.0 equiv.) in anhydrous DCM (20 mL) was cooled to 0 ºC under an argon atmosphere. HATU (1.6 g, 4.1 mmol, 1.1 equiv.) and DIPEA (1.3 mL, 7.5 mmol, 2.0 equiv.) were added and left to stir for 30 min. Then a solution of *N*-PMB-*L*-alanine methyl ester **S1** (0.84 g, 3.8 mmol, 1.0 equiv.) in DCM (6 mL) was added. The reaction was stirred for 1 h before allowing the temperature to increase to room temperature. After 45 h, the reaction mixture was diluted with EtOAc (150 mL) and washed twice with a 1M $KHSO_4$ (2 × 60 mL). The organic layer was then washed twice with aqueous sodium bicarbonate (2 × 60 mL) and brine (2 × 60 mL). The organic layer was dried over $MgSO_4$, filtered and volatiles removed under reduced pressure. The crude product was purified by flash column chromatography (silica gel, 50% EtOAc/petroleum ether) to afford the product **1a** as a colourless/pale yellow oil (1.7 g, 96%), product exists as rotamers 4.5:1.

Major rotamer δ_H (600 MHz, $CDCl_3$: 7.32-7.24 (3H, m, **H15/H17+H16**), 7.17 (2H, d, *J* 8.5 Hz, **H15/17**), 6.95 (2H, d, *J* 8.5 Hz, **H8**), 6.78 (2H, d, *J* 8.3 Hz, **H7**), 5.23 (1H, d, *J* 8.8 Hz, **H18**), 4.80 (1H, q, *J* 8.3 Hz, **H12**), 4.31 (1H, d, *J* 16.6 Hz, **H5'**), 4.23 (1H, d, *J* 16.6 Hz, **H5**), 4.18-4.13 (1H, m, **H3**), 3.77 (3H, s, **H10**), 3.66 (3H, s, **H1**), 3.06 (1H, m, **H13'**), 2.94 (1H, dd, *J* 13.1, 5.7 Hz, **H13**), 1.40 (9H, s, **H21**), 1.26 (3H, d, *J* 7.1 Hz, **H4**); **Minor rotamer δ_H** (600 MHz, $CDCl_3$)**:*** 7.32-7.24 (3H, m, **H15/H17+H16**), 7.19 (2H, d, **H15/17**), 6.99 (2H, d, *J* 8.5 Hz, **H8**), 6.76 (2H, d, *J* 8.6 Hz, **H7**), 5.40 (1H, d, *J* 8.7 Hz, **H18**), 4.94 (1H, q, *J* 8.2 Hz, **H12**), 4.65 (1H, d, *J* Hz, **H5'**), 4.48 (1H, q, *J* 7.5 Hz, **H3**), 4.18-4.13 (1H, m, **H5**), 3.78 (3H, s, **H10**), 3.53 (3H, s, **H1**), 3.06 (1H, m, **H13'**), 2.85-2.80 (1H, m, **H13**), 1.42 (9H, s, **H21**), 0.98 (3H, d, *J* 7.1 Hz, **H4**); **Major rotamer δ_C** (151 MHz, $CDCl_3$): 172.4 (**C11**), 171.7 (**C2**), 159.3 (**C9**), 155.0 (**C19**), 136.6 (**C14**), 129.9 (**C15**), 129.7 (**C6**), 128.6 (**C16**), 128.5 (**C8**), 127.0 (**C17**), 114.2 (**C7**), 79.8 (**C20**), 55.4 (**C10**), 54.9 (**C3**), 52.2 (**C1+C12**)†, 50.5 (**C5**), 40.1 (**C13**), 28.4 (**C21**), 14.6 (**C4**);

* Due to the proximity of the minor rotamer peaks to the major rotamer, a few of the minor rotamer peaks (**H5**, **H13, H15/17, H16**) cannot be observed due to being obscured by the major rotamer. Therefore, approximations of the position have been given because of cross-peaks visible in the COSY. Where possible coupling constants are given, however, for some due to partial overlap with the major rotamer these are not quoted.

† HSQC & HMBC cross-peaks indicate the presence of two co-incident ^{13}C resonances.

HRMS (ESI+): found 471.2495; $C_{26}H_{34}N_2O_6H$, $[M+H]^+$ requires 471.2490; **$[\alpha]_D^{20.0}$** −47.4 (*c* = 1.42, $CHCl_3$); ***v*max** (neat): 3314, 2951, 1705, 1640, 1511, 1245, 1165, 731 cm^{-1}.

(3*S*,6*S*)-3-Benzyl-1-(4-methoxybenzyl)-6-methylpiperazine-2,5-dione (**2a**)

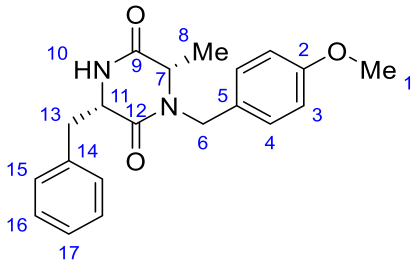

Dipeptide **1a** (1.5 g, 3.2 mmol, 1.0 equiv.) was dissolved in TFA (15 mL) and DCM (15 mL). After stirring for 3 h at room temperature, the solvent was evaporated using compressed air. MeOH (3 × 5 mL) was added and evaporated 3 times, followed by the addition of diethyl ether (1 × 5 mL), and evaporating once more. To the residue, ethyl acetate (5 mL/mmol dipeptide) and saturated aqueous sodium bicarbonate (5 mL/mmol dipeptide) was added and stirred for 22 h. The layers were then separated, and the aqueous layer was extracted three times with EtOAc. The combined organic layers were washed with brine, dried over $MgSO_4$ and filtered. Volatiles were removed under reduced pressure to afford the product **2a** as a yellow solid (1.0 g, 93%).

δ_H (600 MHz, $CDCl_3$): 7.36-7.31 (2H, m, **H16**), 7.29-7.25 (1H, m, **H17**), 7.22 (2H, d, *J* 7.3 Hz, **H15**), 7.16 (2H, d, *J* 8.6 Hz, **H4**), 6.86 (1H, s, **H10**), 6.84 (2H, d, *J* 8.6 Hz, **H3**), 5.27 (1H, d, *J* 14.7 Hz, **H6'**), 4.35-4.28 (1H, m, **H11**), 3.81 (1H, d, *J* 14.5 Hz, **H6**), 3.78 (3H, s, **H1**), 3.71 (1H, q, *J* 7.0 Hz, **H7**), 3.21-3.13 (2H, m, **H13**), 0.93 (3H, d, *J* 7.1 Hz, **H8**); **δ_C** (151 MHz, $CDCl_3$): 169.0 (**C9**), 165.0 (**C12**), 159.5 (**C2**), 135.6 (**C14**), 130.2 (**C15**), 129.9 (**C4**), 129.0 (**C16**), 127.6 (**C17**), 127.5 (**C5**), 114.4 (**C3**), 57.2 (**C11**), 55.4 (**C1**), 54.0 (**C7**), 46.4 (**C6**), 41.4 (**C13**), 18.3 (**C8**); **HRMS** (ESI+): found 339.1709; $C_{20}H_{22}N_2O_3H$, $[M+H]^+$ requires 339.1703; **$[\alpha]_D^{20.0}$** −92.6 (*c* = 1.03, $CHCl_3$); **v_{max}** (neat): 3250, 2920, 1677, 1638, 1422, 1247, 1176, 1034 cm^{-1}.

tert-Butyl (*S*)-3-((((9H-fluoren-9-yl)methoxy)carbonyl)amino)-4-(((*R*)-1-methoxy-1-oxopropan-2-yl)(4-methoxybenzyl)amino)-4-oxobutanoate (**1b**)

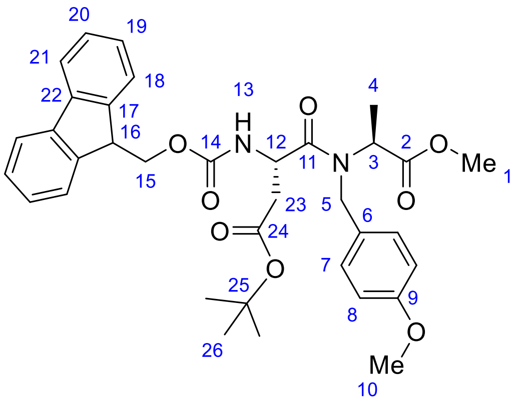

Fmoc-Asp(O*t*Bu)-OH (1.76 g, 8.3 mmol, 1.0 equiv.) was suspended in anhydrous DCM (75 mL) under argon. At 0 ºC, HATU (3.46 g, 9.1 mmol, 1.1 equiv.) and DIPEA (2.9 mL, 16.5 mmol, 2.0 equiv.) were added and stirred for 30 min. A solution of *N*-PMB-*L*-alanine methyl ester **S1** (3.4 g, 8.3 mmol, 1.0 equiv.) in dry DCM (8 mL) was then added. The reaction was stirred for 1 h before allowing the temperature to increase to room temperature and left to stir overnight. Following this, the reaction mixture was diluted with EtOAc (200 mL) and washed with 1M $KHSO_4$ twice (2 × 60 mL). Organic layer then washed twice with aqueous sodium bicarbonate (2 × 60 mL) and brine (2 × 60 mL). Organic layer dried over $MgSO_4$, filtered and concentrated *in vacuo.* Crude solid purified by flash column chromatography (25% EtOAc/petrol) to afford the product **1b** as a colourless oil (4.61 g, 91%), with the product as 3.5:1 mixture of rotamers.

Major rotamer δ_H (600 MHz, $CDCl_3$): 7.76 (2H, d, *J* 7.4 Hz, **H21**), 7.62-7.54 (2H, m, **H18**), 7.40 (2H, t, *J* 7.2 Hz, **H20**), 7.35-7.29 (2H, m, **H19**), 7.28 (2H, d, *J* 8.3 Hz, **H7**), 6.83 (2H, d, *J* 8.3 Hz, **H8**), 5.62 (1H, d, *J* 9.0 Hz, **H13**), 5.03 (1H, q, *J* 6.7 Hz, **H12**), 4.79 (1H, d *J* 16.7 Hz, **H5'**), 4.55 (1H, d, *J* 16.5 Hz, **H5**), 4.36-4.18 (4H, m, **H3**, **H15**, **H16**), 3.71 (3H, s, **H10**), 3.68 (3H, s, **H1**), 2.74 (1H, dd, *J* 15.7, 5.9 Hz, **H23'**), 2.57 (1H, dd, *J* 15.7, 5.7 Hz, **H23**), 1.43 (9H, s, **H26**), 1.37 (3H, d, *J* 7.0 Hz, **H4**); **Minor rotamer δ_H** (600 MHz, $CDCl_3$):* 7.76 (2H, d, *J* 7.4 Hz, **H21**), 7.62-7.54 (2H, m, **H18**), 7.40 (2H, t, *J* 7.2 Hz, **H20**), 7.35-7.29 (2H, m, **H19**), 7.12 (2H, d, *J* 8.1 Hz, **H7**), 6.79 (2H, d, *J* 8.1 Hz, **H8**), 5.66-5.55 (1H, d, *J* 8.8 Hz, **H13**), 5.10 (1H, q, *J* 8.1 Hz, **H12**), 5.03 (1H, q, *J* 7.1 Hz, **HX**), 4.87 (1H, d, *J* 16.0 Hz, **H5'**), 4.40 (1H, m, **H15'**), 4.32-4.29 (1H, m, **H15**), 4.25-4.22 (1H, m, **H3**), 4.21-4.20 (1H, m, **H5+H16**), 3.76 (3H, s, **H10**),

* Due to the proximity of the minor rotamer peaks to the major rotamer, a few of the minor rotamer peaks (**H3**, **H4, H18-21**) cannot be observed or are partially obscured by the major rotamer. Therefore, approximations of the position have been given because of cross-peaks visible in the COSY. Where possible coupling constants are given, and in the case of the aromatic region the major rotamers have been quoted for the minor rotamer.

3.48 (3H, s, **H1**), 2.89 (1H, dd, *J* 15.7, 5.9 Hz, **H23'**), 2.57 (1H, dd, *J* 16.7, 7.8 Hz, **H23'**), 2.61 (1H, dd, *J* 16.3, 5.0 Hz, **H23**), 1.44 (9H, s, **H26**), 1.37 (3H, d, *J* 7.0 Hz, **H4**); **Major rotamer δ_C** (151 MHz, $CDCl_3$): 171.7 (**C2**), 171.3 (**C11**), 169.7 (**C24**), 159.3 (**C9**), 155.5 (**C14**), 143.9 (**C22**), 141.4 (**C17**), 128.5 (**C7**), 128.3 (**C6**), 127.9 (**C20**), 127.2 (**C19**), 125.3 (**C18**), 120.1 (**C21**), 114.3 (**C8**), 81.5 (**C25**), 67.4 (**C16**), 55.3 (**C10**), 55.2 (**C15**), 52.3 (**C1**), 50.8 (**C5**), 48.7 (**C12**), 47.2 (**C3**), 39.0 (**C23**), 28.1 (**C26**),14.4 (**C4**); **HRMS** (ESI+): found 617.2863; $C_{35}H_{40}N_2O_8H$, $[M+H]^+$ requires 617.2857; $[\alpha]_D^{20.0}$ −49.8 (*c* = 0.84, $CHCl_3$); ν_{max} (neat): 2950, 1722, 1649, 1513, 1247, 1161, 740 cm^{-1}.

tert-Butyl 2-((2*S*,5*S*)-4-(4-methoxybenzyl)-5-methyl-3,6-dioxopiperazin-2-yl)acetate (**2b**)

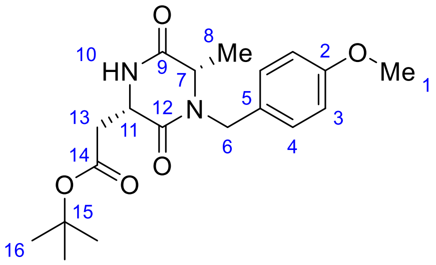

To a solution of dipeptide **1b** (4.6 g, 7.48 mmol, 1 equiv.) in DCM (60 mL) at rt, was added piperidine (15 mL, 20% of the total volume) dropwise. The reaction mixture was then left at rt overnight. After 24 hours the solvent was evaporated using compressed air, and the crude reaction mixture then sequentially washed and evaporated with DCM and then two portions of MeCN. The crude product was then purified by flash column chromatography (10% then 30% EtOAc/DCM) to afford the product **2b** as a colourless oil (2.60 g, 96%); $\boldsymbol{\delta_H}$ (400 MHz, $CDCl_3$): 7.18 (2H, d, *J* 8.6 Hz, **H4**), 6.86 (2H, d, *J* 8.7 Hz, **H3**), 6.48 (1H, s, **H10**), 5.22 (1H, d, *J* 14.7 Hz, **H6'**), 4.41 (1H, dt, *J* 11.3, 2.9 Hz, **H11**), 3.94 (1H, d, J 14.7 Hz, **H6**), 3.86 (1H, q, *J* 7.0 Hz, **H7**), 3.80 (3H, s, **H1**), 3.09 (1H, dd, *J* 17.5, 2.5 Hz, **H13**), 2.57 (1H, dd, *J* 17.4, 11.4, **H13**), 1.50 (3H, d, J 7.1 Hz, **H8**), 1.47 (9H, s, **H16**); $\boldsymbol{\delta_C}$ (101 MHz, $CDCl_3$): 170.0 (**C14**), 168.2 (**C9**), 164.9 (**C12**), 159.6 (**C2**), 129.8 (**C4**), 127.4 (**C5**), 114.5 (**C3**), 82.6 (**C15**), 55.5 (**C1**), 54.3 (**C7**), 52.6 (**C11**), 46.8 (**C6**), 41.0 (**C13**), 28.2 (**C16**), 19.3 (**C8**); HRMS (ESI+): found 385.1739; $C_{19}H_{26}N_2O_5Na$, $[M+Na]^+$ requires 385.1734; $\boldsymbol{[\alpha]_D^{20.0}}$ −72.0 (*c* = 0.76, $CHCl_3$); $\boldsymbol{\nu_{max}}$ (neat): 2935, 1685, 1657, 1513, 1249, 1154, 910, 731 cm^{-1}.

2.3 Foldamer Synthesis

2.3.1 Monomers

(3*S*,6*S*)-3-Benzyl-4-(6-bromopyridin-2-yl)-1-(4-methoxybenzyl)-6-methylpiperazine-2,5-dione (**3a**):

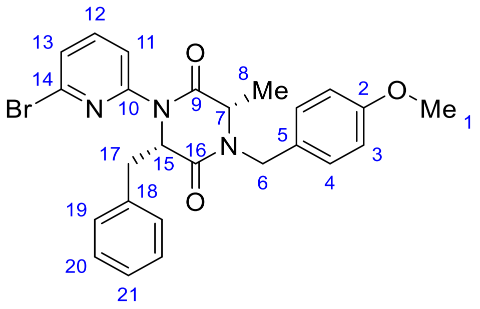

Prepared according to **General Procedure A** using **2a** (750 mg, 2.2 mmol, 1 equiv.), 2,6-dibromopyridine (2.6 g, 11.1 mmol, 5.0 equiv.), $Pd_2(dba)_3$ (101.5 mg, 0.11 mmol, 5 mol%), Xantphos (192.4 mg, 0.33 mmol, 15 mol%), Cs_2CO_3 (1.8 g, 5.5 mmol, 2.5 equiv.) and toluene (22 mL). Reaction temperature = 120 ºC and reaction time = 4 h. The crude product was purified by flash column chromatography (5% EtOAc/DCM) to afford the product **3a** as a yellow solid (802 mg, 73%).

δ_H (600 MHz, $CDCl_3$): 7.88 (1H, d, *J* 8.1 Hz, **H11**), 7.59 (1H, t, *J* 7.9 Hz, **H12**), 7.33 (1H, d, *J* 7.7 Hz, **H13**), 7.26-7.22 (2H, m, **H20**), 7.21-7.16 (3H, m, **H4 + H21**), 7.07 (2H, d, *J* 7.3 Hz, **H19**), 6.84 (d, 2H, *J* 8.5 Hz, **H3**), 5.88 (1H, t, *J* 4.9 Hz, **H15**), 5.30 (1H, d, *J* 14.7 Hz, **H6'**), 3.84-3.79 (2H, m, **H6 + H7**), 3.78 (3H, s, **H1**), 3.41 (1H, dd, *J* 4.6, 14.0 Hz, **H17'**), 3.05 (1H, dd, *J* 14.0, 5.2 Hz, **H17**), 0.71 (3H, d, *J* 7.1 Hz, **H8**); **δ_C** (151 MHz, $CDCl_3$): 167.4 (**C9**), 165.1 (**C16**), 159.6 (**C2**), 151.0 (**C10**), 139.8 (**C12**), 139.5 (**C14**), 135.9 (**C18**), 130.4 (**C19**), 130.1 (**C4**), 128.8 (**C20**), 127.5 (**C21**), 127.4 (**C5**), 125.7 (**C13**), 119.8 (**C11**), 114.4 (**C3**), 59.8 (**C15**), 55.4 (**C1**), 55.1 (**C7**), 46.1 (**C6**), 38.8 (**C17**), 18.1 (**C8**); **HRMS** (ESI+): found 494.1079; $C_{25}H_{24}BrN_3O_3H$, $[M+H]^+$ requires 494.1074; **$[\alpha]_D^{20.0}$** −54.1 (*c* = 1.02, $CHCl_3$); **ν_{max}** (neat): 2935, 1653, 1511, 1424, 1392, 1243, 1163, 703 cm^{-1}.

(3*S*,6*S*)-3-Benzyl-1-(4-methoxybenzyl)-6-methyl-4-(6-(2-oxopyrrolidin-1-yl)pyridin-2-yl)piperazine-2,5-dione (**4**)

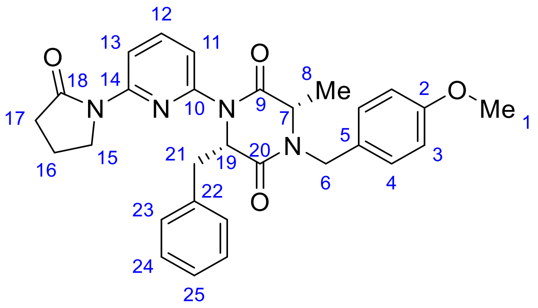

Prepared according to **General Procedure A** using **3a** (450 mg, 0.91 mmol, 1 equiv.), 2-pyrrolidinone (0.14 mL, 1.8 mmol, 2.0 equiv.), $Pd_2(dba)_3$ (41.7 mg, 0.046 mmol, 5 mol%), Xantphos (79.0 mg, 0.14 mmol, 15 mol%), Cs_2CO_3 (741.4 mg, 2.3 mmol, 2.5 equiv.) and toluene (9 mL). Reaction temperature = 120 ºC and reaction time = 2 h. The crude product was purified by flash column chromatography (40% EtOAc/DCM) to afford the product **4** as a pale-yellow solid (410.5 mg, 90%).

δ_H (600 MHz, $CDCl_3$): 8.29 (1H, d, *J* 8.2 Hz, **H13**), 7.80 (1H, t, *J* 8.1 Hz, **H12**), 7.65 (1H, d, *J* 7.9 Hz, **H11**), 7.26-7.17 (5H, m, **H4 + H24 + H25**), 7.07 (2H, m, **H23**), 6.84 (2H, d, *J* 8.6 Hz, **H3**), 5.77 (1H, t, *J* 4.4 Hz, **H19**), 5.28 (1H, d, *J* 14.7 Hz, **H6'**), 4.10-4.01 (2H, m, **H15**), 3.83-3.89 (2H, m, **H6** + **H7**), 3.78 (3H, s, **H1**), 3.40 (1H, dd, *J* 13.9, 4.0 Hz, **H21'**), 3.02 (1H, dd, *J* 14.0, 4.8 Hz, **H21**), 2.69 (2H, t, *J* 7.8 Hz, **H17**), 2.15 (2H, quint, *J* 7.4 Hz, **H16**), 0.58 (3H, d, *J* 7.1 Hz, **H8**); **δ_C** (151 MHz, $CDCl_3$): 175.1 (**C18**), 167.1 (**C9**), 165.3 (**C20**), 159.6 (**C2**), 150.6 (**C10**), 148.7 (**C14**), 139.6 (**C12**), 135.9 (**C22**), 130.5 (**C23**), 130.1 (**C5**), 128.8 (**C24**), 127.52 (**C25**), 127.47 (**C4**), 116.6 (**C11**), 114.4 (**C3**), 112.2 (**C13**), 59.7 (**C19**), 55.4 (**C1**), 55.1 (**C7**), 47.5 (**C15**), 46.1 (**C6**), 38.3 (**C21**), 33.7 (**C17**), 18.0 (**C8**), 17.6 (**C16**); **HRMS** (ESI+): found 499.2345; $C_{29}H_{30}N_4O_4H$, $[M+H]^+$ requires 499.2340; **$[\alpha]_D^{20.0}$** −54.4 (*c* = 1.04, $CHCl_3$); **ν_{max}** (neat): 2939, 1705, 1649, 1439, 1403, 1236, 729 cm^{-1}.

(3*S*,6*S*)-6-Benzyl-3-methyl-1-(6-(2-oxopyrrolidin-1-yl)pyridin-2-yl)piperazine-2,5-dione **(5)**

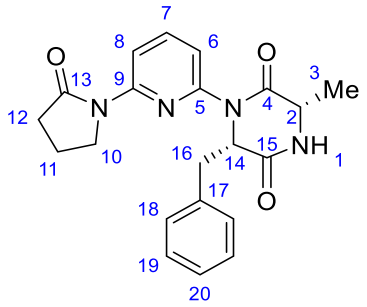

Prepared according to **General Procedure C** using **4** (400 mg, 0.80 mmol, 1 equiv.), ceric ammonium nitrate (1.80 g, 3.3 mmol, 4.1 equiv.) in H_2O (3.3 mL) and MeCN (9.8 mL), MeCN:H_2O ratio and total reaction concentration (3:1, 0.06 M). Reaction temperature = 0 °C for 1 h then rt for 21 h, total reaction time = 22 h. The crude product was purified twice by flash column chromatography (5% MeOH/DCM) to afford the product **5** as a white solid (185.0 mg, 61% yield).

$\boldsymbol{\delta_H}$ (600 MHz, $CDCl_3$): 8.33 (1H, d, *J* 8.2 Hz, **H6**), 7.84 (1H, t, *J* 8.1 Hz, **H7**), 7.71 (1H, d, *J* 7.8 Hz, **H8**), 7.33-7.24 (3H, m, **H19+H20**), 7.10 (2H, m, **H18**), 7.02 (1H, s, **H1**), 5.67 (1H, t, *J* 4.0 Hz, **H14**), 4.07 (2H, t, *J* 7.2 Hz, **H10**), 4.03 (1H, dq, *J* 7.1, 2.6 Hz, **H2**), 3.36 (1H, dd, *J* 14.0, 3.6 Hz, **H16'**), 3.00 (1H, dd, *J* 14.0, 4.9 Hz, **H16**), 2.69 (2H, t, *J* 8.1 Hz, **H12**), 2.19-2.15 (2H, quint, *J* 7.7 Hz, **H11**), 0.63 (3H, d, *J* 7.1 **Hz**, **H3**); $\boldsymbol{\delta_C}$ (151 MHz, $CDCl_3$): 175.1 (**C13**), 167.3 (**C15**), 166.7 (**C4**), 150.6 (**C9**), 148.8 (**C5**), 139.7 (**C7**), 135.7 (**C17**), 130.5 (**C18**), 128.8 (**C19**), 127.6 (**C20**), 116.8 (**C8**), 112.3 (**C6**), 59.7 (**C14**), 52.2 (**C2**), 47.5 (**C10**), 37.8 (**C16**), 33.7 (**C12**), 21.1 (**C3**), 17.6 (**C11**); **HRMS** (ESI+): found 379.1770; $C_{21}H_{22}N_4O_3H$, $[M+H]^+$ requires 379.1765; $\boldsymbol{[\alpha]_D^{20.0}}$ +134 (*c* = 0.55, $CHCl_3$); $\boldsymbol{\nu_{max}}$ (neat): 2935, 2246, 1674, 1439, 1377, 1228, 906, 723 cm^{-1}.

tert-Butyl 2-((2*S*,5*S*)-1-(6-bromopyridin-2-yl)-4-(4-methoxybenzyl)-5-methyl-3,6-dioxopiperazin-2-yl)acetate (**3b**)

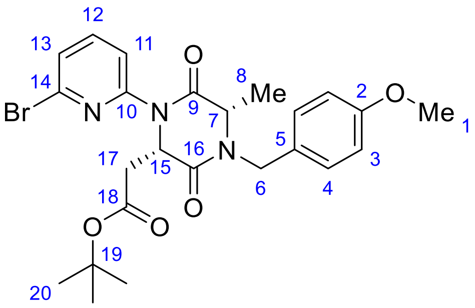

Prepared according to **General Procedure A** using **3a** (1 equiv.), 2,6-dibromopyridine (5.0 equiv.), $Pd_2(dba)_3$ (5 mol%), Xantphos (15 mol%), Cs_2CO_3 (1.5 equiv.) and toluene (0.1 M). Reaction temperature = 120 ºC and reaction time = 6 h. The crude product was purified by flash column chromatography (20% EtOAc/DCM) to afford the product **3b** as a yellow oil: 40 mg SM (73.1 mg, 79%), 955 mg SM (922.9 mg, 67%).

$\boldsymbol{\delta_H}$ (600 MHz, $CDCl_3$): 7.74 (1H, d, *J* 8.0 Hz, **H11**), 7.57 (1H, t, *J* 7.9 Hz, **H12**), 7.35 (1H, d, *J* 7.7 Hz, **H13**), 7.22 (2H, d, *J* 8.6 Hz, **H4**), 6.86 (2H, d, *J* 8.7 Hz, **H3**), 5.59 (1H, dd, *J* 6.3, 4.5 Hz, **H15**), 5.28 (1H, d, *J* 14.8 Hz, **H6'**), 4.06 (1H, d *J* 14.8 Hz, **H6**), 4.04 (1H, q, *J* 7.2 Hz, **H7**), 3.79 (3H, s, **H1**), 3.09 (1H, dd, *J* 15.8, 4.4 Hz, **H17'**), 2.63 (1H, dd, *J* 15.8, 6.3 Hz, **H17**), 1.68 (3H, d, *J* 7.1 Hz, **H8**), 1.39 (9H, s, **H20**); $\boldsymbol{\delta_C}$ (151 MHz, $CDCl_3$): 168.7 (**C18**), 167.5 (**C9**), 165.0 (**C16**), 159.4 (**C2**), 150.8 (**C10**), 139.8 (**C12**), 139.6 (**C14**), 129.8 (**C4**), 127.4 (**C5**), 126.0 (**C13**), 119.8 (**C11**), 114.3 (**C3**), 81.5 (**C19**), 55.9 (**C15**), 55.4 (**C7**), 55.3 (**C1**), 46.4 (**C6**), 38.8 (**C17**), 28.0 (**C20**), 18.4 (**C8**); $[\alpha]_D^{20.0}$ −33.8 (*c* = 0.99, $CHCl_3$); **HRMS** (ESI+): found 540.1110; $C_{24}H_{28}BrN_3O_5Na$, $[M+Na]^+$ requires 540.1105; ν_{max} (neat): 2976, 1664, 1513, 1426, 1392, 1247, 1154 cm^{-1}.

tert-Butyl 2-((2*S*,5*S*)-4-(4-methoxybenzyl)-5-methyl-3,6-dioxo-1-(6-(2-oxopyrrolidin-1-yl)pyridin-2-yl)piperazin-2-yl)acetate (**10**)

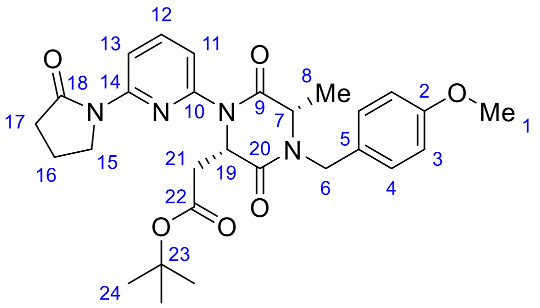

Prepared according to **General Procedure B** using **3b** (198 mg, 0.382 mmol, 1 equiv.), 2-pyrrolidinone (73 μL, 0.955 mmol, 2.5 equiv.), CuI, (230 mg, 0.0734 mmol, 20 mol%), 1,2-dimethylethylenediamine (16.5 μL, 0.153 mmol, 40 mol%), K_2CO_3 (106 mg, 0.764 mmol, 2.0 equiv.), and toluene (4 mL). Reaction temperature = 100 °C and reaction time = 22 h. Purification by flash column chromatography (silica gel, 30% then 50% ethyl acetate in petroleum ether) afforded the product **10** as a yellow oil, (155 mg, 79%).

δ_H (600 MHz, $CDCl_3$): 8.27 (1H, d, *J* 8.2 Hz, **H13**), 7.72 (1H, t, *J* 8.2 Hz, **H12**), 7.53 (1H, d, *J* 8.2 Hz, **H11**), 7.23 (2H, d, *J* 8.4 Hz, **H4**), 6.85 (2H, d, *J* 8.5 Hz, **H3**), 5.61 (1H, dd, *J* 7.4, 4.5 Hz, **H19**), 5.21 (1H, d, *J* 14.8 Hz, **H6'**), 4.13-4.04 (4H, m, **H6+H7+H15**), 3.78 (3H, s, **H1**), 3.06 (1H, dd, *J* 15.2, 4.5 Hz, **H21'**), 2.66 (2H, t, *J* 8.2 Hz, **H17**), 2.59 (1H, dd, *J* 15.2, 7.6 Hz, **H21**), 2.13 (2H, quint, *J* 7.7 Hz, **H16**), 1.66 (3H, d, *J* 7.1 Hz, **H8**), 1.38 (9H, s, **H24**); **δ_C** (151 MHz, $CDCl_3$): 175.1 (**C18**), 168.7 (**C20/22**), 167.6 (**C9**), 165.4 (**C20/22**), 159.5 (**C2**), 150.6 (**C10/14**), 148.6 (**C10/14**), 139.6 (**C12**), 129.9 (**C4**), 127.8 (**C5**), 115.8 (**C11**), 114.4 (**C3**), 112.2 (**C13**), 81.6 (**C23**), 55.9 (**C19**), 55.8 (**C7**), 55.4 (**C1**), 47.4 (**C15**), 46.7 (**C6**), 39.7 (**C21**), 33.7 (**C17**), 28.1 (**C24**), 18.8 (**C8**), 17.6 (**C16**); **$[\alpha]_D^{20}$** -35.0 (*c* = 0.78, $CHCl_3$); **HRMS** (ESI+): found 523.2547; $C_{25}H_{34}N_4O_6H$, $[M+H]^+$ requires 523.2551; **ν_{max} (thin film)**: 2978.1, 2929.7, 1707.1, 1669.8, 1513.3, 1446.2, 1405.2, 1241.2, 1155.5.

2-((2*S*,5*S*)-4-(4-Methoxybenzyl)-5-methyl-3,6-dioxo-1-(6-(2-oxopyrrolidin-1-yl)pyridin-2-yl)piperazin-2-yl)acetic acid (**10a**)

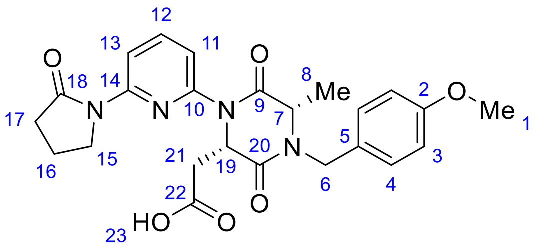

To a cooled (0°C) solution of **10** (60 mg, 0.115 mmol, 1.0 equiv.) in dry DCM (0.6 mL) under argon was added TFA (0.6 mL) [1:1 (DCM:TFA), 0.1 M]. The reaction mixture was then warmed to rt then left to stir for 2 h. Once the reaction had completed the solvent was evaporated off under a stream of compressed air and the concentrated reaction mixture was first neutralised with saturated aqueous $NaHCO_3$, and then the pH adjusted to ~pH 3-4 using 1M HCl. Following this the reaction mixture was then diluted with DCM (10 mL) and transferred to a separatory funnel. The layers were separated, and the aqueous layer then washed with DCM (3 x 5 ml). The organic layers were then combined, dried over $MgSO_4$, filtered under gravity, and then concentrated in vacuo to yield a faint yellow solid. Purification by flash column chromatography (silica gel, 1% MeOH then 5% MeOH:DCM) afforded the product as a white solid (36.3 mg, 68%).

δ_H (400 MHz, *d*$_6$-DMSO): 12.53 (1H, s, **H23**), 8.18 (1H, d, *J* 8.2 Hz, **H13**), 7.87 (1H, t, *J* 8.1 Hz, **H12**), 7.45 (1H, d, *J* 7.9 Hz, **H11**), 7.30 (2H, d, *J* 8.3 Hz, **H4**), 6.91 (2H, d, *J* 8.3 Hz, **H3**), 5.40 (1H, dd, *J* 6.8, 4.4 Hz, **H19**), 4.85 (1H, d, *J* 14.9 Hz, **H6'**), 4.33 (1H, d, *J* 14.9 Hz, **H6**), 4.08 (1H, q, *J* 7.0 Hz, **H7**), 4.01 (2H, t, *J* 7.2 Hz, **H15**), 3.74 (3H, s, **H1**), 2.99 (1H, dd, *J* 15.6, 4.4 Hz, **H21'**), 2.63 (1H, dd, *J* 15.9, 6.9 Hz, **H21**), 2.59 (2H, t, *J* 7.8 Hz ,**H17**), 2.05 (2H, quint, *J* 7.7 Hz, **H16**), 1.52 (3H, d, *J* 7.0 Hz, **H8**); **δ_C** (101 MHz, *d*$_6$-DMSO): 174.8 (**C18**), 170.9 (**C22**), 166.9 (**C9**), 164.7 (**C20**), 158.6 (**C2**), 150.3 (**C10/14**), 148.6 (**C10/14**), 139.6 (**C12**), 129.4 (**C4**), 128.6 (**C5**), 115.7 (**C11**), 113.9 (**C3**), 111.1(**C13**), 55.9 (**C7**), 55.6 (**C19**), 55.0 (**C1**), 46.7 (**C15**), 46.1 (**C6**), 38.1 (**C21**), 33.1 (**C17**), 18.7 (**C8**), 17.0 (**C16**); **$[\alpha]_D^{20}$** -49.0 (*c* = 1.00, $CHCl_3$); **HRMS** (ESI+): found 467.1927; $C_{24}H_{26}N_4O_6H$, $[M+H]^+$ requires 467.1925; **ν_{max} (thin film)**: 2989.3, 2359.4, 1707.1, 1662.2, 1513.3,1446.2,1408.9, 1319.5, 1244.9, 1162.9.

tert-Butyl 2-((2*S*,5*S*)-5-methyl-3,6-dioxo-1-(6-(2-oxopyrrolidin-1-yl)pyridin-2-yl)piperazin-2-yl)acetate (**11**)

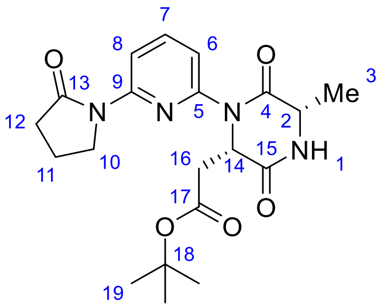

Prepared according to **General Procedure C** using **10** (286 mg, 0.546 mmol, 1 equiv.), ceric ammonium nitrate (1.20 g, 2.19 mmol, 4.0 equiv.) in H_2O (2.4 mL), and MeCN (7.1 mL), MeCN:H_2O ratio and total reaction concentration (3:1, 0.06 M). Reaction temperature = 0 °C and reaction time = 2.5 h. Purification by flash column chromatography (silica gel, 2% MeOH:DCM) afforded the product **11** as a yellow oil, (182 mg, 83%).

δ_H (600 MHz, $CDCl_3$): 8.29 (1H, d, *J* 8.3 Hz, **H6/8**), 7.74 (1H, t, *J* 8.1 Hz, **H7**), 7.49 (1H, s, **H1**), 7.42 (1H, d, *J* 7.9 Hz, **H6/8**), 5.37 (1H, dd, *J* 6.0, 3.4 Hz, **H14**), 4.27 (1H, q, *J* 6.8 Hz, **H2**), 4.03 (2H, t, *J* 7.2 Hz, **H10**), 3.03 (1H, dd, *J* 16.4, 3.3 Hz, **H16'**), 2.66 (2H, t, *J* 8.0 Hz, **H12**), (1H, dd, *J* 16.4, 6.5 Hz, **H16**), 2.11 (2H, quint, *J* 7.5 Hz, **H11**), 1.71 (3H, *J* 7.0 Hz, **H3**), 1.36 (9H, s, **H19**); **δ_C** (151 MHz, $CDCl_3$): 175.1 (**C13**), 168.9 (**C15/17**), 167.5 (**C15/17**), 167.2 (**C4**) 150.8 (**C5/9**), 148.7 (**C5/9**), 139.5 (**C7**), 116.9 (**C6/8**), 112.5 (**C6/8**), 81.6 (**C18**), 55.7 (**C14**), 52.4 (**C2**), 47.4 (**C10**), 38.0 (**C16**), 33.7 (**C12**), 28.1 (**C19**), 21.3 (**C3**), 17.6 (**C11**); **$[\alpha]_D^{20}$** -89.2 (*c* = 1.11, $CHCl_3$); **HRMS** (ESI+): found 403.1958; $C_{20}H_{26}N_4O_5H$, $[M+H]^+$ requires 403.1976; **ν_{max} (thin film)**: 3218.6, 2980.0, 2249.4, 1679.2, 1587.8, 1446.2, 1377.3, 1239.3, 1148.0.

2.3.2 Dimers

(3*S*,6*S*)-3-Benzyl-1-(6-((2*S*,5*S*)-2-benzyl-4-(4-methoxybenzyl)-5-methyl-3,6-dioxopiperazin-1-yl)pyridin-2-yl)-6-methyl-4-(6-(2-oxopyrrolidin-1-yl)pyridin-2-yl)piperazine-2,5-dione (**6**)

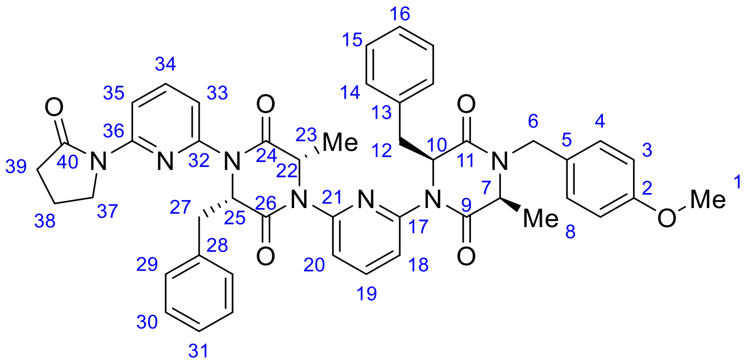

Prepared according to **General Procedure A** using **5** (1 equiv.), **3a** (1.1 equiv.), $Pd_2(dba)_3$ (5 mol%), Xantphos (15 mol%), Cs_2CO_3 (2.5 equiv.) and toluene (0.1 M). Reaction temperature = 120 ºC and reaction time = 3 h. The crude product was purified by flash column chromatography (5% MeOH:DCM) to afford the product as a white solid: 40 mg SM (59.7 mg, 71%) 180 mg SM (248.6 mg, 66%).

δ_H (600 MHz, $CDCl_3$): 8.34 (1H, d, *J* 8.2 Hz, **H35**), 7.89-7.84 (2H, m, **H19**, **H34**), 7.80-7.77 (2H, m, **H18, H33**), 7.63 (1H, d, *J* 7.8 Hz, **H20**), 7.33-7.15 (10H, m, **H4**, **H15-16**, **H29-31**), 7.03 (2H, d, *J* 7.7 Hz, **H14**), 6.84 (2H, d, *J* 8.7 Hz, **H3**), 5.84 (1H, t, *J* 4.6 Hz, **H25**), 5.65 (1H, t, *J* 4.5 Hz, **H10**), 5.25 (1H, d, *J* 14.7 Hz, **H6'**), 5.06 (1H, q, *J* 7.1 Hz, **H22**), 4.15-4.08 (2H, m, **H37**), 3.84-3.79 (2H, m, **H6**, **H7**), 3.78 (3H, s, **H1**), 3.51 (1H, dd, *J* 14.1, 4.2 Hz, **H27'**), 3.34 (1H, dd, *J* 14.0, 4.3 Hz, **H12'**), 3.18 (1H, dd, *J* 14.1, 5.0 Hz, **H27**), 2.92 (1H, dd, *J* 14.0, 4.7 Hz, **H12**), 2.70 (2H, t, *J* 8.0 Hz, **H39**), 2.17 (2H, quint, *J* 7.6 Hz, **H38**), 0.63 (3H, d, *J* 7.1 Hz, **H8**), 0.61 (3H, d, *J* 7.3 Hz, **H23**); **δ_C** (151 MHz, $CDCl_3$): 175.1 (**C40**), 167.2 (**C9**), 170.0 (**C24**), 165.6 (**C26**), 164.9 (**C11**), 159.6 (**C2**), 150.6 (**C32**), 149.8 (**C21**), 149.3 (**C17**), 148.7 (**C36**), 139.8 (**C34**), 139.5 (**C19**), 135.8 (**C13**), 135.7 (**C28**), 130.4 (**C29**), 130.3 (**C5**), 130.2 (**C14**), 129.0 (**C16**), 128.9 (**C30/31**), 127.8 (**C30/31**), 127.5 (**C15**), 127.4 (**C4**), 119.7 (**C18**), 119.5 (**C20**), 116.6 (**C33**), 114.4 (**C3**), 112.3 (**C35**), 60.5 (**C25**), 59.8 (**C10**), 56.0 (**C22**), 55.4 (**C1**), 55.0 (**C7**), 47.5 (**C37**), 46.1 (**C6**), 38.9 (**C27**), 38.4 (**C12**), 33.7 (**C39**), 19.2 (**C23**), 18.0 (**C8**), 17.7 (**C38**); **HRMS** (ESI+): found 792.3451; $C_{46}H_{45}N_7O_6H$, $[M+H]^+$ requires 792.3504; **$[\alpha]_D^{20.0}$** −57.9 (*c* = 0.76, $CHCl_3$); **ν_{max}** (neat): 2932, 1668, 1586, 1415, 1228, 1144 cm^{-1}.

(3*S*,6*S*)-3-Benzyl-1-(6-((2*S*,5*S*)-2-benzyl-5-methyl-3,6-dioxopiperazin-1-yl)pyridin-2-yl)-6-methyl-4-(6-(2-oxopyrrolidin-1-yl)pyridin-2-yl)piperazine-2,5-dione (**7**)

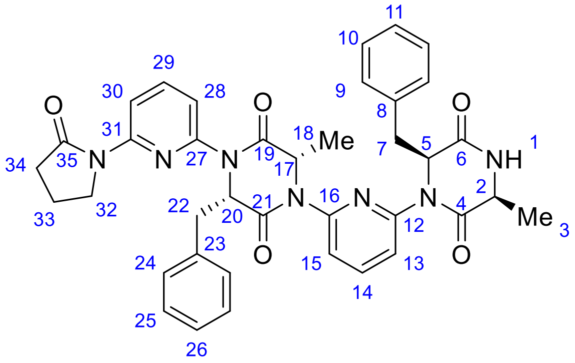

Prepared according to **General Procedure C** using **6** (190 mg, 0.240 mmol, 1 equiv.), ceric ammonium nitrate (539.3 mg, 0.980 mmol, 4.1 equiv.) in H_2O (1 mL), and MeCN (100 mL), MeCN:H_2O ratio and total reaction concentration (100:1, 0.002 M). Reaction temperature = 0 °C for 1 h then rt for 21 h, total reaction time = 22 h. Purification by flash column chromatography (5% MeOH/DCM) to afford the product as a white solid (96.7 mg, 60%);

δ_H (600 MHz, $CDCl_3$): 8.33 (1H, d, *J* 8.2 Hz, **H30**), 7.92 (1H, t, *J* 8.1 Hz, **H14**), 7.86 (1H, t, *J* 8.1 Hz, **H29**), 7.82 (1H, d, *J* 8.0 Hz, **H13**), 7.77 (1H, d, *J* 7.9 Hz, **H28**), 7.73 (1H, d, *J* 7.9 Hz, **H15**), 7.32-7.27 (3H, m, **H25-26**), 7.25-7.20 (3H, m, **H10-11**), 7.18 (2H, d, *J* 7.6 Hz, **H24**), 7.05 (2H, d, *J* 7.4 Hz, **H9**), 6.35 (1H, s, **H1**), 5.85 (1H, t, *J* 4.6 Hz, **H20**), 5.59 (1H, t, *J* 4.2 Hz, **H5**), 5.12 (1H, q, *J* 7.1 Hz, **H17**), 4.09 (2H, t, *J* 7.4 Hz, **H32**), 4.01 (1H, qd, *J* 7.1, 2.6 Hz, **H2**), 3.51 (1H, dd, *J* 14.0, 4.2 Hz, **H22'**), 3.32 (1H, dd, *J* 14.1, 3.7 Hz, **H7'**), 3.18 (1H, dd, *J* 14.1, 5.0 Hz, **H22**), 2.86 (1H, dd, *J* 14.1, 4.7 Hz, **H7**), 2.70 (2H, t, *J* 8.1 Hz, **H34**), 2.17 (2H, quint, *J* 7.7 Hz, **H33**), 0.64 (3H, d, *J* 0.64 Hz **H18**), 0.63 (3H, d, *J* 0.63 Hz **H3**); δ_C (151 MHz, $CDCl_3$): 175.1 (**C35**), 167.0 (**C19**), 166.7 (**C6**), 166.5 (**C4**), 165.7 (**C21**), 150.6 (**C27**), 149.8 (**C16**), 149.3 (**C12**), 148.6 (**C31**), 139.8 (**C29**), 139.6 (**C14**), 135.7 (**C23**), 135.4 (**C8**), 130.5 (**C9**), 130.4 (**C24**), 129.0 (**C25**), 128.8 (**C10**), 127.8 (**C26**), 127.6 (**C11**), 120.0 (**C13**), 119.5 (**C15**), 116.3 (**C28**), 112.4 (**C30**), 60.6 (**C20**), 59.8 (**C5**), 56.0 (**C17**), 52.2 (**C2**), 47.5 (**C32**), 38.9 (**C22**), 37.8 (**C7**), 33.7 (**C34**), 21.3 (**C3**), 19.2 (**C18**), 17.7 (**C33**); **HRMS** (ESI+): found 672.2905; $C_{38}H_{37}N_7O_5H$, $[M+H]^+$ requires 672.2929; $[\alpha]_D^{20.0}$ +32.0 (*c* = 0.70, $CHCl_3$); ν_{max} (neat): 2937, 1681, 1588, 1444, 1405, 1230, 753 cm^{-1}.

tert-Butyl 2-((2*S*,5*S*)-4-(6-((2*S*,5*S*)-2-(2-(*tert*-butoxy)-2-oxoethyl)-4-(4-methoxybenzyl)-5-methyl-3,6-dioxopiperazin-1-yl)pyridin-2-yl)-5-methyl-3,6-dioxo-1-(6-(2-oxopyrrolidin-1-yl)pyridin-2-yl)piperazin-2-yl)acetate (**12**)

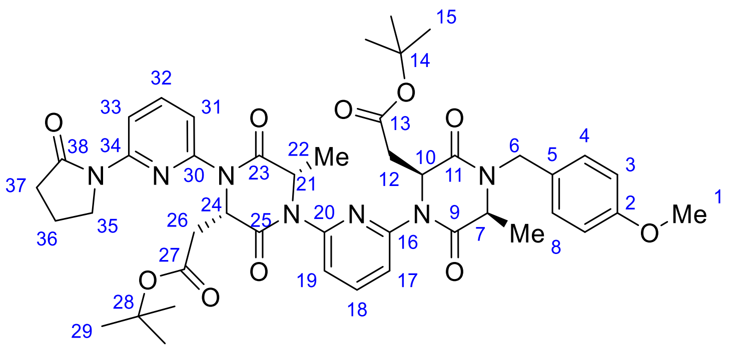

Prepared according to **General Procedure B** using **11** (57.7 mg, 0.148 mmol, 1 equiv.), **3b** (84.6 mg, 0.162 mmol, 1.1 equiv.), CuI (5.7 mg, 0.0300 mmol, 20 mol%), 1,2-dimethylethylenediamine (6.4 μL, 0.054 mmol, 40 mol%), K_2CO_3 (41 mg, 0.297 mmol, 2.0 equiv.), and toluene (1.5 mL). Reaction temperature = 100 °C and reaction time = 23 h. Purification by flash column chromatography (silica gel, 1% then 2% MeOH:DCM) afforded the product as a yellow foam solid, (79.3 mg, 64%).

δ_H (400 MHz, $CDCl_3$): 8.31 (1H, d, *J* 8.2 Hz, **H31/33**), 7.83-7.76 (3H, m, **H18** + **H31/33** + **H32**), 7.67 (1H, d, *J* 8.0 Hz, **H17/19**), 7.60 (1H, dd, *J* 6.9, 1.8 Hz, **H17/19**), 7.27 (2H, d, *J* 8.6 Hz, **H4**), 6.89 (2H, d, *J* 8.7 Hz, **H3**), 5.71 (1H, dd, *J* 7.9, 4.5 Hz**, H24**), 5.55-5.40 (2H, m, **H10 + H21**), 5.25 (1H, d, *J* 14.8 Hz, **H6'**), 4.16-4.07 (4H, m, **H6** + **H7** + **H35**), 3.80 (3H, s, **H1**), 3.22 (1H, dd, *J* 15.0, 4.6 Hz, **H26'**), 3.10 (1H, dd, *J* 16.0, 4.2 Hz, **H12'**), 2.78-2.65 (1H, m, **H26**) 2.68 (2H, t, *J* 8.3 Hz, **H37**), 2.56 (1H, dd, *J* 16.1, 6.4 Hz, **H12**), 2.15 (2H, quint, *J* 8.2 Hz, **H37**), 1.71 (6H, m, **H8 + H22**), 1.42 (9H, s, **H15/29**), 1.38 (9H, s, **H15/29**); **δ_C** (101 MHz, $CDCl_3$): 175.1 (**C38**), 168.7 (**C11/C13**), 168.6 (**C25/C27**), 167.6 (**C23**), 167.5 (**C9**), 165.7 (**C25/C27**), 164.9 (**C11/C13**), 159.5 (**C2**), 150.6 (**C30/C34**), 149.7 (**C30/C34**), 149.5 (**C16/20**), 148.5 (**C18/C31/C32/C33**), 139.7 (**C16/C20**), 139.6 (**C18/C31/C32/C33**), 129.9 (**C4**), 127.7 (**C2**), 119.4 (**C17/19**), 119.3 (**C18/C31/C32/C33**), 115.6 (**C17/19**), 114.4 (**C3**), 112.1 (**C31/C33**), 81.8 (**C14/C28**), 81.6 (**C14/C28**), 56.8 (**C24**), 56.1 (**C21**), 56.0 (**C10**), 55.6 (**C7**), 55.4 (**C1**), 47.4 (**C35**), 46.6 (**C6**), 40.1 (**C26**), 38.7 (**C12**), 33.7 (**C37**), 28.1 (**C15/C29**), 28.0 (**C15/C29**), 20.0 (**C22**), 18.6 (**C8**), 17.6 (**C36**); **$[\alpha]_D^{20}$** -20.0 (*c* = 1.07, $CHCl_3$); **HRMS** (ESI+): found 840.3932; $C_{44}H_{53}N_7O_{10}H$, $[M+H]^+$ requires 840.3927; **ν_{max} (thin film)**: 2974.4, 2251.3, 1722.0,1677.3, 1587.8, 1513.3, 1446.2, 1401.5, 1230.0, 1148.0.

2-((2*S*,5*S*)-4-(6-((2*S*,5*S*)-2-(Carboxymethyl)-4-(4-methoxybenzyl)-5-methyl-3,6-dioxopiperazin-1-yl)pyridin-2-yl)-5-methyl-3,6-dioxo-1-(6-(2-oxopyrrolidin-1-yl)pyridin-2-yl)piperazin-2-yl)acetic acid (**13**)

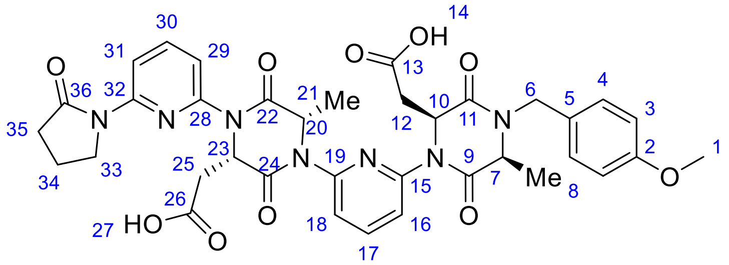

To a cooled (0°C) solution of **12** (25.2 mg, 0.030 mmol, 1.0 equiv.) in dry DCM (0.3 mL) under argon was added TFA (0.3 mL) [1:1 (DCM:TFA), 0.1 M]. The reaction mixture was warmed to rt then left to stir for 4 h. Once the reaction had completed the solvent was evaporated off under a stream of compressed air and the concentrated reaction mixture neutralised with saturated aqueous $NaHCO_3$. Following this the reaction mixture was diluted with DCM (10 mL) and transferred to a separatory funnel. The layers were separated, and the aqueous layer washed with DCM (3 x 5 ml). The combined organic layers were then discarded, and the aqueous layer acidified with 1 M HCl to ~pH 4. The aqueous layer was then re-extracted with DCM (3 x 10 mL). The combined organic layers were then dried over $MgSO_4$, filtered under gravity, and then concentrated in vacuo to yield **13** as a white solid, 18.3 mg, 84%.

δ_H (400 MHz, *d*$_6$-DMSO): 12.63 (2H, s, **H14+H27**), 8.20 (1H, d, *J* 8.3 Hz, **H31**), 7.99 (1H, t, *J* 7.9 Hz, **H17**), 7.91 (1H, t, *J* 8.1 Hz, **H30**), 7.71 (1H, d, *J* 8.2 Hz, **H18**), 7.61 (1H, d, *J* 8.0 Hz, **H16**), 7.55 (1H, d, *J* 7.9 Hz, **H29**), 7.31 (1H, d, *J* 8.4 Hz, **H4**), 6.91 (1H, d, *J* 8.5 Hz, **H3**), 5.52 (1H, dd, *J* 6.5, 4.4 Hz, **H23**), 5.34-5.30 (1H, m, **H10**), 5.26 (1H, q, *J* 7.2 Hz, **H20**), 4.84 (1H, d, *J* 15.1 Hz, **H6'**), 4.37 (1H, d, *J* 15.0 Hz, **H6**), 4.09 (1H, q, *J* 7.1 Hz, **H7**), 4.05-3.99 (2H, m, **H33**), 3.74 (3H, s, **H1**), 3.11 (1H, dd, *J* 16.0, 4.4 Hz, **H25'**), 2.98 (1H, dd, *J* 15.7, 3.8 Hz, **H12'**), 2.78 (1H, dd, *J* 15.7, 6.7 Hz, **H25**), 2.69-2.63 (1H, m, **H12**), 2.61 (1H, t, *J* 8.0 Hz, **H35**), 2.06 (1H, quint, *J* 7.4 Hz, **H34**), 1.60 (3H, d, *J* 7.0 Hz, **H21**), 1.53 (3H, d, *J* 6.9 Hz, **H8**); **δ_C** (101 MHz, *d*$_6$-DMSO): 174.8 (**C36**), 171.0 (**C11/13 + C24/26**)*, 167.1 (**C22**), 167.0 (**C9**), 165.4 (**C24/26**), 164.6 (**C11/13**), 158.6 (**C2**), 150.3 (**C28/32**), 149.5 (**C15/19**), 149.4 (**C15/19**), 148.7 (**C28/32**), 139.8 (**C17**), 139.6 (**C30**), 129.4 (**C4**), 128.6 (**C2**), 118.8 (**C16**), 118.7 (**C18**), 115.6 (**C29**), 113.9 (**C3**), 111.0 (**C31**), 56.5 (**C23**), 55.9 (**C10**), 55.8 (**C7**), 55.5 (**C20**), 55.0 (**C1**), 46.7 (**C33**), 46.2 (**C6**), 38.5 (**C25**), 37.9 (**C12**), 33.1 (**C35**), 19.6 (**C21**), 18.6 (**C8**), 17.0 (**C4**); **$[\alpha]_D^{20}$** -38.0 (*c* = 0.90, $CHCl_3$); **HRMS** (ESI+): found 726.2524; $C_{36}H_{36}N_7O_{10}$, $[M-H]^+$ requires 726.2529; **ν_{max} (thin film)**: 3015.4, 2926.0, 1733.2, 1673.6, 1587.3, 1442.5, 1401.1, 1230.0, 1159.2.

---

* HSQC & HMBC cross-peaks indicate the presence of two co-incident ^{13}C resonances.

2.3.3 Trimers

3*S*,6*S*)-3-Benzyl-1-(6-((2*S*,5*S*)-2-benzyl-4-(4-methoxybenzyl)-5-methyl-3,6-dioxopiperazin-1-yl)pyridin-2-yl)-4-(6-((3*S*,6*S*)-3-benzyl-6-methyl-2,5-dioxo-4-(6-(2-oxopyrrolidin-1-yl)pyridin-2-yl)piperazin-1-yl)pyridin-2-yl)-6-methylpiperazine-2,5-dione (**8**)

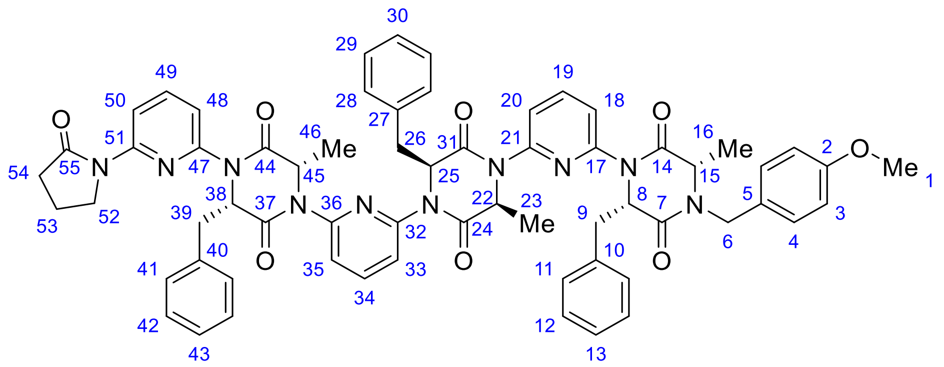

Prepared according to **General Procedure A** using **7** (80.0 mg, 0.12 mmol, 1 equiv.), **3a** (64.8 mg, 0.13 mmol, 1.1 equiv.), $Pd_2(dba)_3$ (5.5 mg, 6.01 µmol 5 mol%), Xantphos (10.3 mg, 0.018 mmol, 15 mol%), Cs_2CO_3 (97.0 mg, 0.30 mmol, 2.5 equiv.) and toluene (1.2 mL). Reaction temperature = 120 ºC and reaction time = 4.5 h. The crude product was purified by flash column chromatography (5% MeOH/DCM) to afford the product **8** as a yellow solid, (94.5 mg, 73%).

δ_H (600 MHz, $CDCl_3$):* 8.33 (1H, d, *J* 8.1 Hz, **H50**), 7.95-7.91 (2H, m, **H34**, **H33**), 7.88-7.33 (2H, m, **H49**, **H19**), 7.78 (1H, d, *J* 7.9 Hz, **H48**), 7.76 (1H, d, *J* 7.9 Hz, **H18**), 7.71-7.66 (2H, m, **H20**, **H35**), 7.35-7.15 (13H, m, **H4**, **H12-13**, **H41-43**, **H29-30**), 7.13 (2H, d, *J* 7.2 Hz, **H28**), 7.02 (2H, d, *J* 7.3 Hz, **H11**), 6.84 (2H, d, *J* 8.6 Hz, **H3**), 5.87 (1H, t, *J* 4.6 Hz, **H38**), 5.77 (1H, t, *J* 4.7 Hz, **H25**), 5.65 (1H, t, *J* 4.4 Hz, **H8**), 5.25 (1H, d, *J* 14.7 Hz, **H6'**), 5.10 (2H, q, *J* 7.1 Hz, **H22**, **H45**), 4.10 (2H, t, *J* 7.5 Hz, **H52**), 3.83-3.78 (2H, m, **H6**, **H15**), 3.78 (3H, s, **H1**), 3.52 (1H, dd, *J* 14.0, 4.1 Hz, **H39'**), 3.45 (1H, dd, *J* 14.1, 4.5 Hz, **H26'**), 3.33 (1H, dd, *J* 14.1, 4.2 Hz, **H9'**), 3.17 (1H, dd, *J* 14.0, 5.0 Hz, **H39**), 3.07 (1H, dd, *J* 14.1, 4.8 Hz, **H26**), 2.91 (1H, dd, *J* 14.0, 4.7 Hz, **H9**), 2.70 (2H, t, *J* 8.1 Hz, **H54**), 2.21-2.13 (2H, quint, *J* 7.7 Hz, **H53**), 0.68 (3H, d, *J* 7.1 Hz, **H23**), 0.65 (3H, d, *J* 7.1 Hz, **H46**), 0.62 (3H, d, *J* 7.1 Hz, **H16**); **δ_C** (151 MHz, $CDCl_3$): 175.0 (**C55**), 167.0 (**C14**), 166.9 (**C31**), 166.8 (**C37**), 165.5 (**C44**), 165.2 (**C24**), 164.8 (**C7**), 159.4 (**C2**), 150.5 (**C47/C51**), 149.6 (**C17/21**), 149.5 (**C32/36**), 149.1 (**C17/21**), 149.0 (**C32/36**), 148.5 (**C47/C51**), 139.7 (**C34**), 139.6 (**C49**), 139.4 (**C19**), 135.7 (**C10**), 135.6 (**C40**), 135.5 (**C27**), 130.3 (**C41**), 130.2 (**C11**), 130.2 (**C28**), 130.1 (**C5**), 128.9 (**C42**), 128.8 (**C29**), 128.7 (**C12**), 127.8 (**C43**), 127.7 (**C30**), 127.5 (**C13**), 127.4 (**C4**), 119.6 (**C20**), 119.6 (**C33**),

* Due to no observable nOe interaction between the pyridine protons and CH of adjacent DKP unit, the pyridine peaks have been assigned on the basis on analogous peak positions in the dimer.

119.4 (**C18**), 119.2 (**C35**), 116.5 (**C48**), 114.2 (**C3**), 112.2 (**C50**), 60.4 (**C38**), 60.3 (**C25**), 59.6 (**C8**), 55.9 (**C22**), 55.7 (**C45**), 55.3 (**C1**), 54.9 (**C15**), 47.4 (**C52**), 46.0 (**C6**), 38.9 (**C26**), 38.8 (**C39**), 38.3 (**C9**), 33.6 (**C54**), 19.2 (**C23**), 19.0 (**C46**), 17.9 (**C16**), 17.5 (**C53**); **HRMS** (ESI+): found 1085.4674; $C_{63}H_{60}N_{10}O_8H$, $[M+H]^+$ requires 1085.4668; found 1107.4493; $C_{63}H_{60}N_{10}O_8Na$, $[M+Na]^+$ requires 1107.4488; $[\alpha]_D^{20.0}$ −72.5 (c = 1.36, $CHCl_3$); ν_{max} (neat): 2935, 2248, 1668, 1398, 1224, 727 cm^{-1}.

(3*S*,6*S*)-3-Benzyl-1-(6-((2*S*,5*S*)-2-benzyl-4-(6-((2*S*,5*S*)-2-benzyl-5-methyl-3,6-dioxopiperazin-1-yl)pyridin-2-yl)-5-methyl-3,6-dioxopiperazin-1-yl)pyridin-2-yl)-6-methyl-4-(6-(2-oxopyrrolidin-1-yl)pyridin-2-yl)piperazine-2,5-dione (**9**):

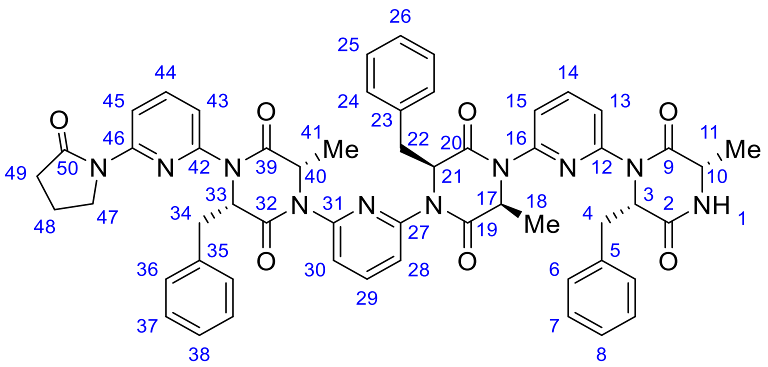

Prepared according to **General Procedure C** using **8** (35 mg, 0.032 mmol, 1 equiv.), ceric ammonium nitrate ((72.5 mg, 0.13 mmol, 4.1 equiv.) in H_2O (0.13 mL), and MeCN (13 mL), MeCN:H_2O ratio and total reaction concentration (100:1, 0.002 M). Reaction temperature = 60 °C 21 h. Purification by flash column chromatography (4% MeOH/DCM) to afford the product **9** as a yellow solid (21.7 mg, 70%).

δ_H (600 MHz, $CDCl_3$): 8.33 (1H, d, *J* 8.1 Hz, **H45**), 7.94-7.66 (8H, m, **H13-15**, **H28-30**, **H43-44**), 7.36-7.28 (4H, m, **H36-37**), 7.25-7.16 (7H, m, **H7-8**, **H25-26**, **H38**), 7.13 (2H, d, *J* 7.2 Hz, **H24**), 7.04 (2H, d, *J* 7.0 Hz, **H6**), 6.26 (1H, s, **H1**), 5.86 (1H, t, *J* 4.6 Hz, **H33**), 5.78 (1H, t, *J* 4.7 Hz, **H21**), 5.58 (1H, t, *J* 4.2 Hz, **H3**), 5.14 (1H, q, *J* 7.1 Hz, **H17**), 5.09 (1H, q, *J* 7.1 Hz, **H40**), 4.13-4.05 (2H, m, **H47**), 4.04-3.96 (1H, m, **H10**), 3.51 (1H, dd, *J* 14.0, 4.0 Hz, **H34'**), 3.44 (1H, dd, J 14.1, 4.5 Hz, **H22'**), 3.31 (1H, dd, *J* 14.1, 3.7 Hz, **H4'**), 3.17 (1H, dd, *J* 14.0, 5.1 Hz, **H34**), 3.07 (1H, dd, *J* 14.1, 4.9 Hz, **H22**), 2.85 (1H, dd, *J* 14.1, 4.7 Hz, **H4**), 2.73-2.66 (2H, m, **H49**), 2.23-2.12 (2H, m, **H48**), 0.70 (3H, d, *J* 7.1 Hz, **H18**), 0.63 (3H, d, *J* 2.6 Hz, **H41**), 0.62 (3H, d, *J* 2.5 Hz, **H11**); **δ_C** (151 MHz, $CDCl_3$):* 175.1 (**C50**), 167.1 (**C19**), 167.0 (**C39**), 166.7 (**C9**), 166.6 (**C2**), 165.6 (**C32**), 165.4 (**C20**), 150.7 (**C42/46**), 149.7 (**C12**), 149.3 (**C27**), 149.2 (**C16**), 148.5 (**C31**), 139.8 (**C29**), 139.7 (**C44**), 139.6 (**C14**), 135.7 (**C35**), 135.6 (**C23**), 135.3 (**C5**), 130.5 (**C6**), 130.4 (**C36**), 130.3 (**C24**), 129.0 (**C25/26**), 128.9 (**C7/8**), 128.8 (**C37/38**), 127.8 (**C7/8**), 127.7 (**C25/26**), 127.6 (**C37/38**), 119.9 (**C15**), 119.7 (**C28**), 119.6 (**C13**), 119.3 (**C30**), 116.7 (**C43**), 112.4 (**C45**), 60.5 (**C33**), 60.5 (**C21**), 59.8 (**C3**), 56.0 (**C40**), 55.8 (**C17**), 52.2 (**C10**), 47.5 (**C47**), 39.0 (**C22**), 38.9 (**C34**), 37.8 (**C4**), 33.7 (**C49**), 21.3 (**C11**), 19.3 (**C18**), 19.2 (**C41**), 17.7 (**C48**); **HRMS** (ESI+): found 965.4099; $C_{55}H_{52}N_{10}O_7H$, $[M+H]^+$

* One of the peaks associated with **C42/46** was unable to be detected and may be co-incident with another carbon. Due to no HMBC peak with **H45**, this peak can therefore not be located.

requires 965.4093; found 987.3918; $C_{55}H_{52}N_{10}O_7Na$, $[M+Na]^+$ requires 987.3913; **$[\alpha]_D^{20.0}$** −7.5 (c = 1.34, $CHCl_3$); **ν_{max}** (neat): 3012, 2933, 1675, 1443, 1403, 1228, 753 cm^{-1}.

3 Solution-Phase Conformational Analysis by NMR

3.1 General Comments

All NOESY and ROESY data were acquired on a Bruker 400 or 600 MHz spectrometer with a mixing time (t_{mix}) of 200 ms for ROESY and 600 ms for NOESY. Unless otherwise stated, spectra were acquired in $CDCl_3$ at room temperature. Full NOESY/ROESY spectra at two levels of zoom are given, followed by a zoomed view of the cross-peak region of greatest interest. Integrations were carried out manually. Where coloured bands are placed over spectra and with alternating orange/blue colouration, this is done to aid visualization and the colours are arbitrary (unless otherwise stated). Since some of the signals examined are very weak, bands of noise are frequently present at similar levels of intensity.

3.2 Monomer 4

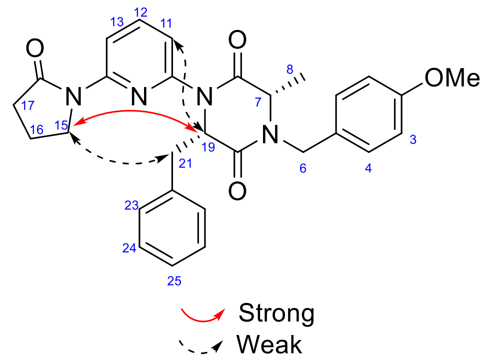

Figure S1: nOe correlations ($CDCl_3$, 600 MHz) from NOESY spectrum of **4**, solid red arrows are strong cross-peaks; dashed black arrows are weak cross-peaks.

nOe's were observed between the pyrrolidinone and adjacent diketopiperazine hydrogen atoms with a strong interaction between **H15↔H19** and slightly weaker one between **H15↔H21**. (**Figure S2**) In addition, very weak nOe interactions were observed between the diketopiperazine and pyridine (**H11↔H19**). (**Figures S3-S4**) This suggests that the diketopiperazine monomer is adopting a dipole-opposed conformation (forcing the nitrogen group *anti* to the adjacent carbonyl groups), as in a dipole-aligned/unopposed conformation, these protons would like much closer, and hence a more prevalent nOe would be observed. In addition to this, no visible interactions can be observed between lactam protons (**H13↔ H15**), which would not be the case in a dipole-aligned conformation.

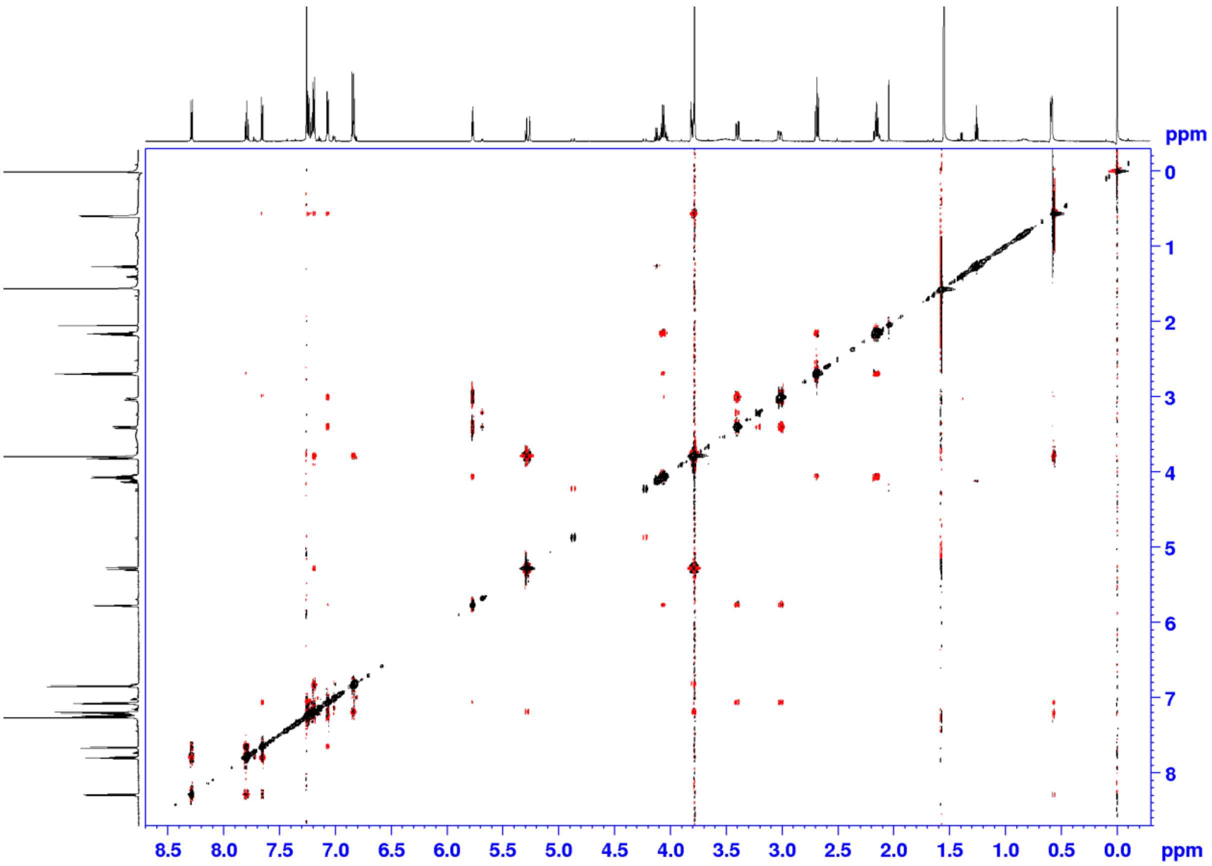

Figure S2: Full NOESY spectrum of monomer **4**

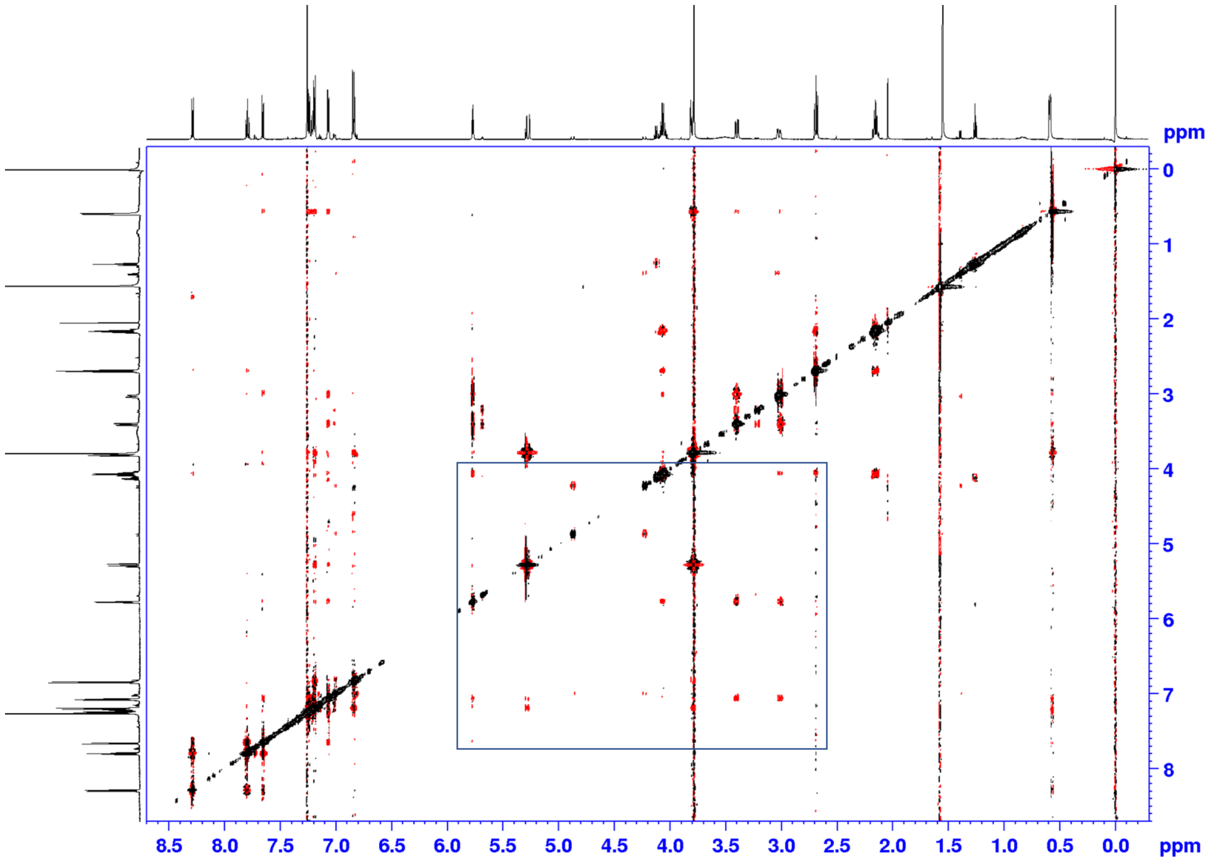

Figure S3: Full NOESY spectrum of monomer **4**, with weak interactions

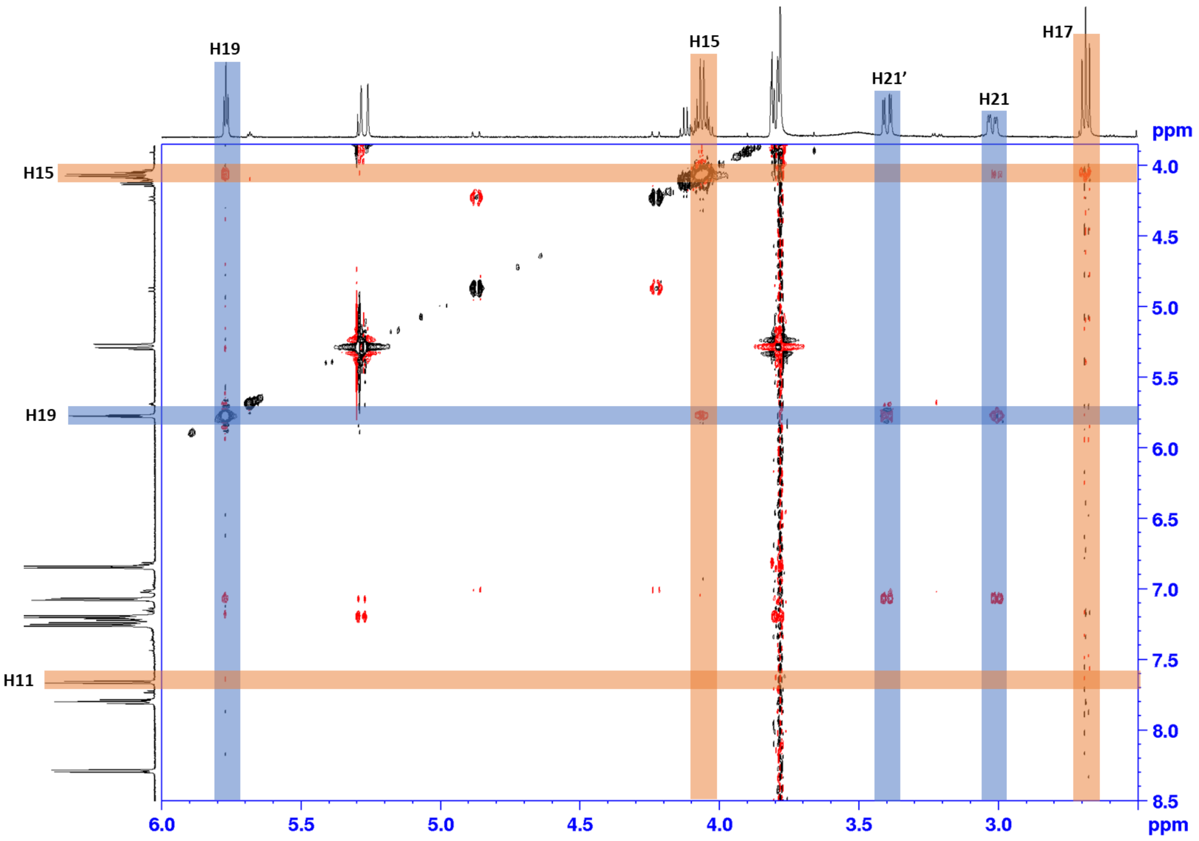

Figure S4: Zoomed view of greatest interest of monomer **4**

3.3 Dimer 6

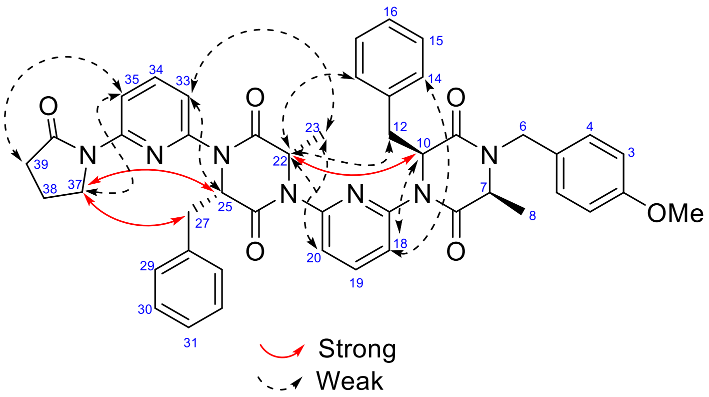

Figure S5: nOe correlations ($CDCl_3$, 600 MHz) from ROESY spectrum of **6**, solid red arrows are strong cross-peaks; dashed black arrows are weak cross-peaks.

Like monomer **4's** conformation, strong interactions were observed between the pyrrolidinone and adjacent diketopiperazine hydrogen atoms (**H37↔H25** and **H37↔H27**). (**Figure S6**) Furthermore, faint, or weak interactions were present between the pyridine protons (**H33 & H35**) and the attached pyrrolidinone (**H37 & H39**) and diketopiperazine units (**H25↔H23**), suggesting that the pyrrolidinone and diketopiperazine carbonyl groups are positioned *anti* to the pyridine nitrogen atom. (**Figure S7+8**) In addition to the pyridine adjacent to the pyrrolidinone, the first diketopiperazine linker also possesses a weak nOe to the other adjacent pyridine (**H22↔H20**), thereby demonstrating that both pyridine groups are opposing each other, which is unlike the imidazolidinone foldamers synthesised by the Hamilton and Knipe groups,[7,12,13] gives rise to a zig-zag shape with sidechains of each unit projecting from opposite faces, contrasting the linear or curved dipolar repulsion foldamers that exist currently. Further evidence for this zig-zag shape can be seen in the strong nOe interaction between the individual diketopiperazine units (**H22↔H10** and **H22↔H12**), which would not be possible in a dipole-aligned conformation.

The second pyridine linker (**H18 and H20**) also exhibited faint or weak nOe interaction with the protons of the attached diketopiperazine units, indicating that the nitrogen is positioned *anti* to the two adjacent carbonyl groups. Confirming the individual diketopiperazine unit both experience dipolar repulsion.

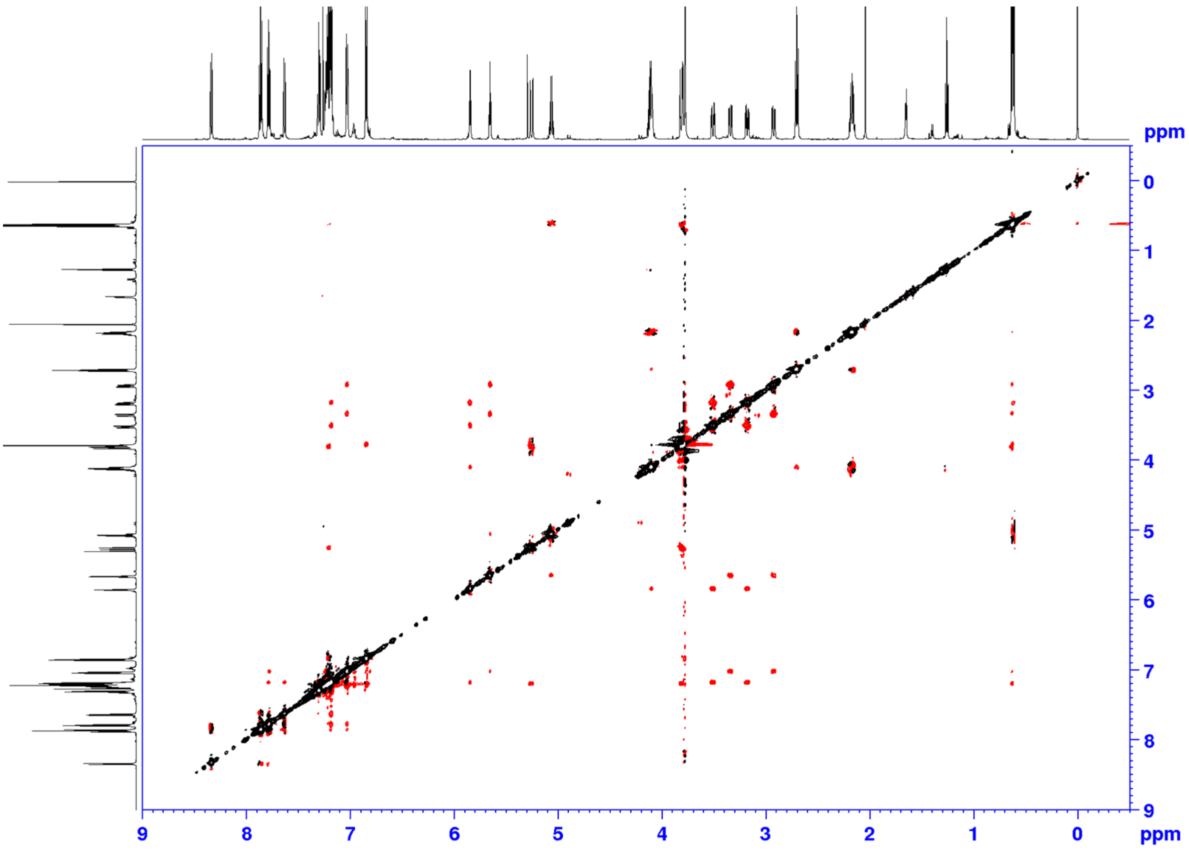

Figure S6: Full ROESY spectrum of dimer **6**

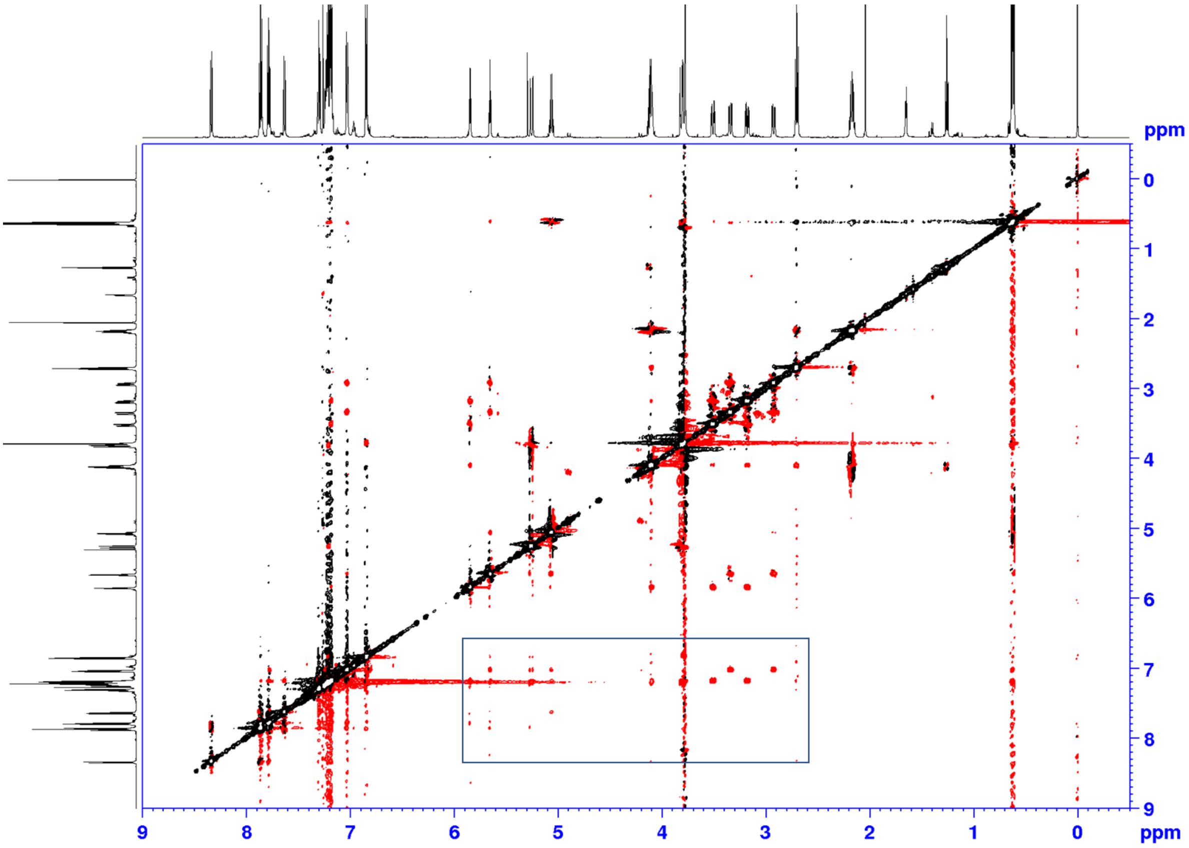

Figure S7: Full ROESY spectrum of dimer **6**, with weak interactions

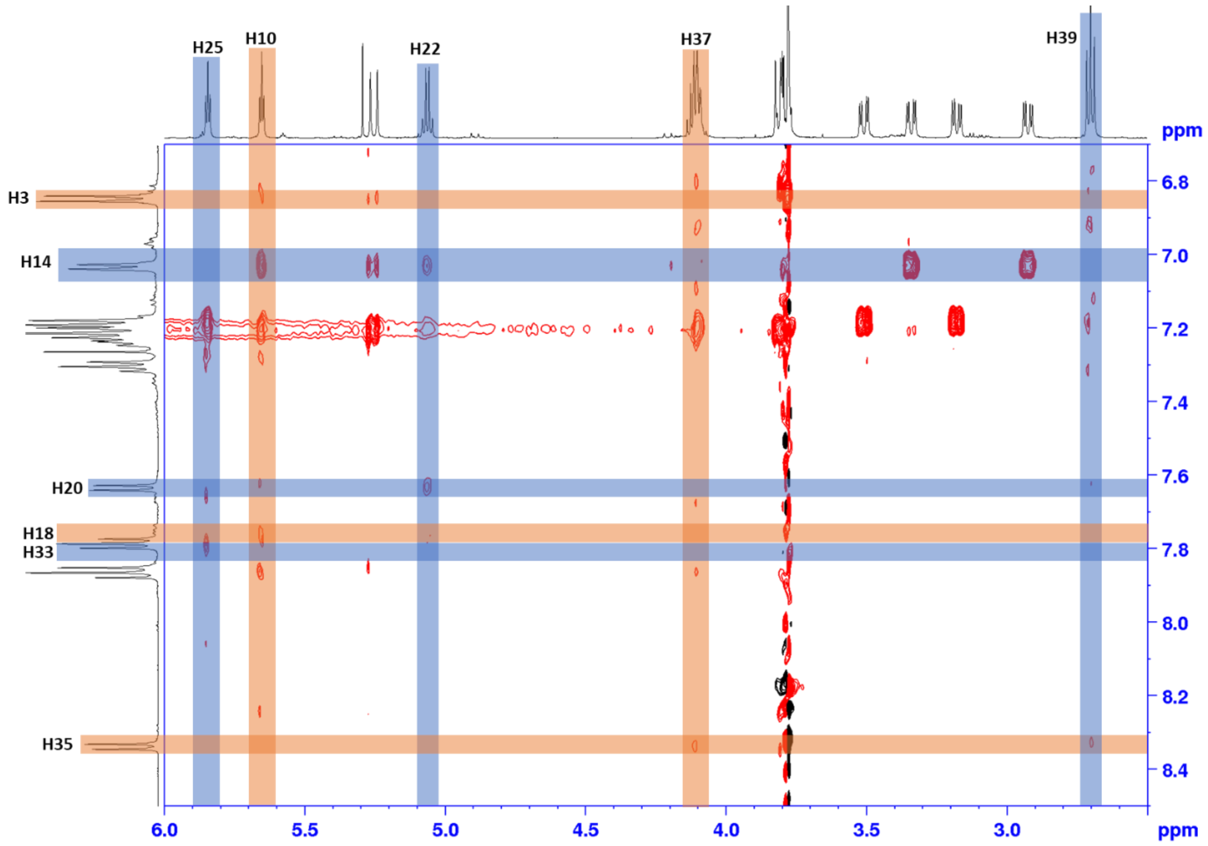

Figure S8: Zoomed view of greatest interest of dimer **6**

Dimer 6 in *d6*-DMSO

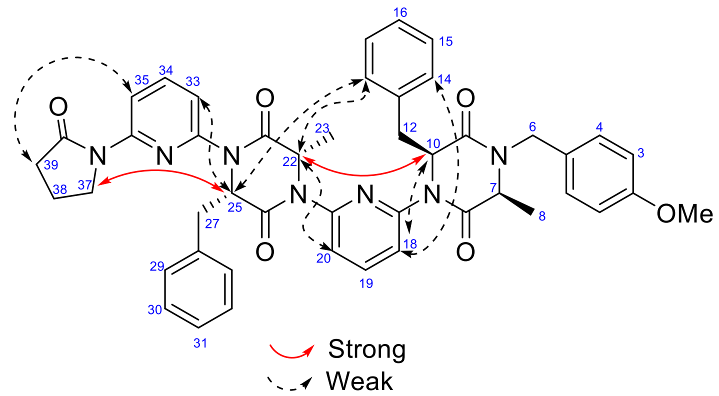

Figure S9: nOe correlations (*d6*-DMSO, 400 MHz) from ROESY spectrum of **6**, solid red arrows are strong cross-peaks; dashed black arrows are weak cross-peaks.

The conformation of the dimer was then analysed in *d6*-DMSO to see if the dipole-opposed conformation would be retained in a more polar solvent. Looking at the key nOe interactions between the DKP's C_α-H and *meta*-pyridine peaks, weak nOe were observed between (**H33↔H25**, **H22↔H20**, **H10↔H18**), (**Figure S11-12**) in addition to strong inter-residue nOe between **H37↔H25** and **H22↔H10**. (**Figure S10**) Overall, this is consistent with the nOe interaction observed when dimer **6's** conformation was analysed in $CDCl_3$, indicating that the diketopiperazine unit are still occupying a dipole-opposed/*anti*-conformation, demonstrating that even in a more polar solvent, the dipole-opposed conformation is still highly favourable.

Overall, the only major difference between dimer **6** in *d6*-DMSO vs. $CDCl_3$ was the absence of weak nOe between **H37** and the *meta*-pyridine **H35**, together with the lack of a strong inter-residue nOe between **H37↔H35**, which may indicate slight rotation about *C*-*N* pyrrolidinone bond, but still populating a dipole opposed conformation.

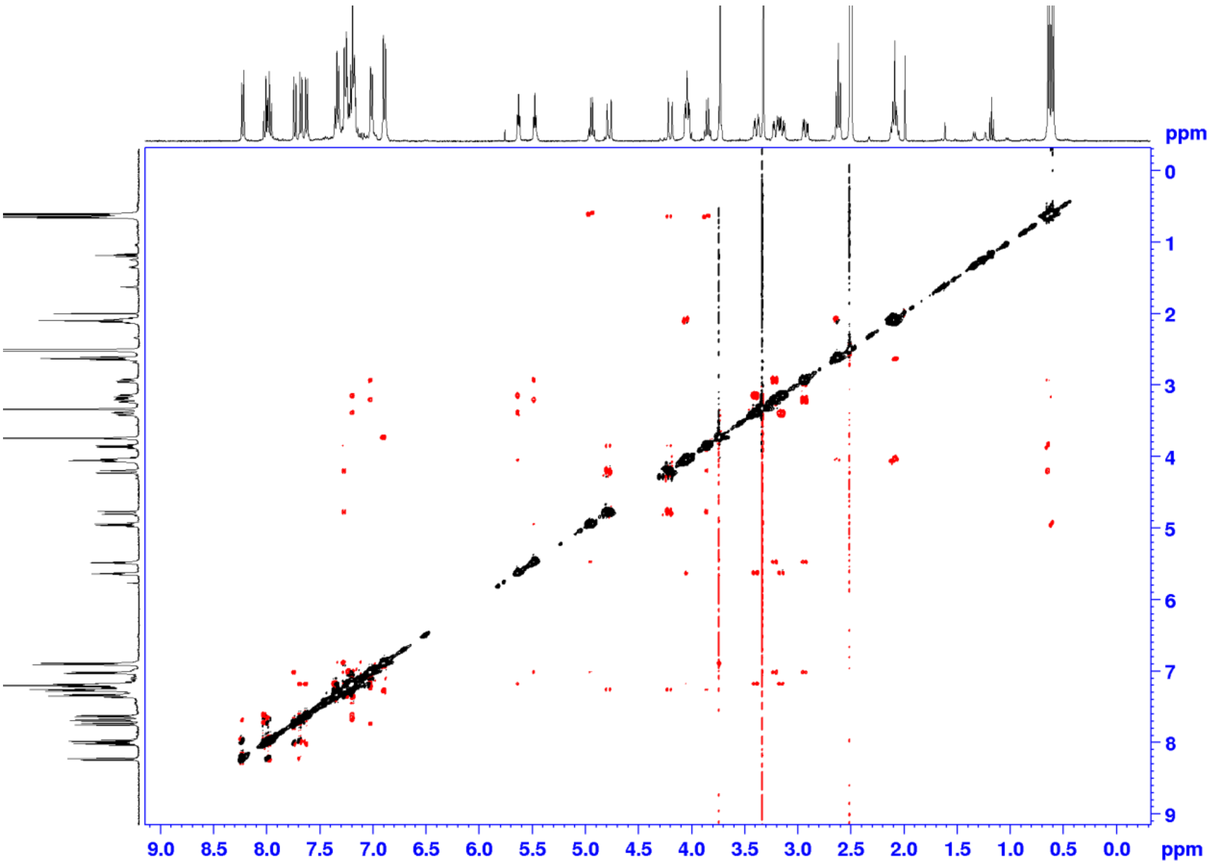

Figure S10: Full ROESY spectrum of dimer **6** (*d6*-DMSO)

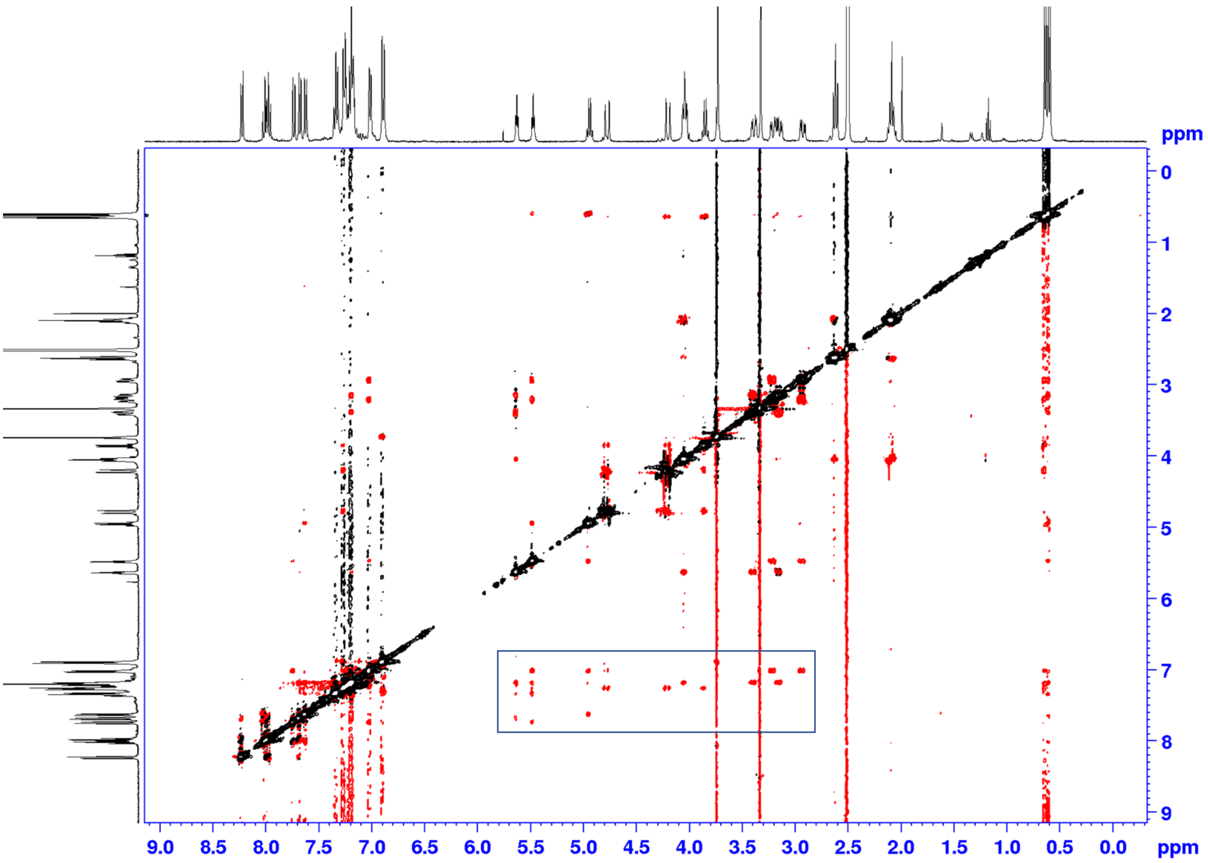

Figure S11: Full ROESY spectrum of dimer **6** (*d6*-DMSO), with weak interactions

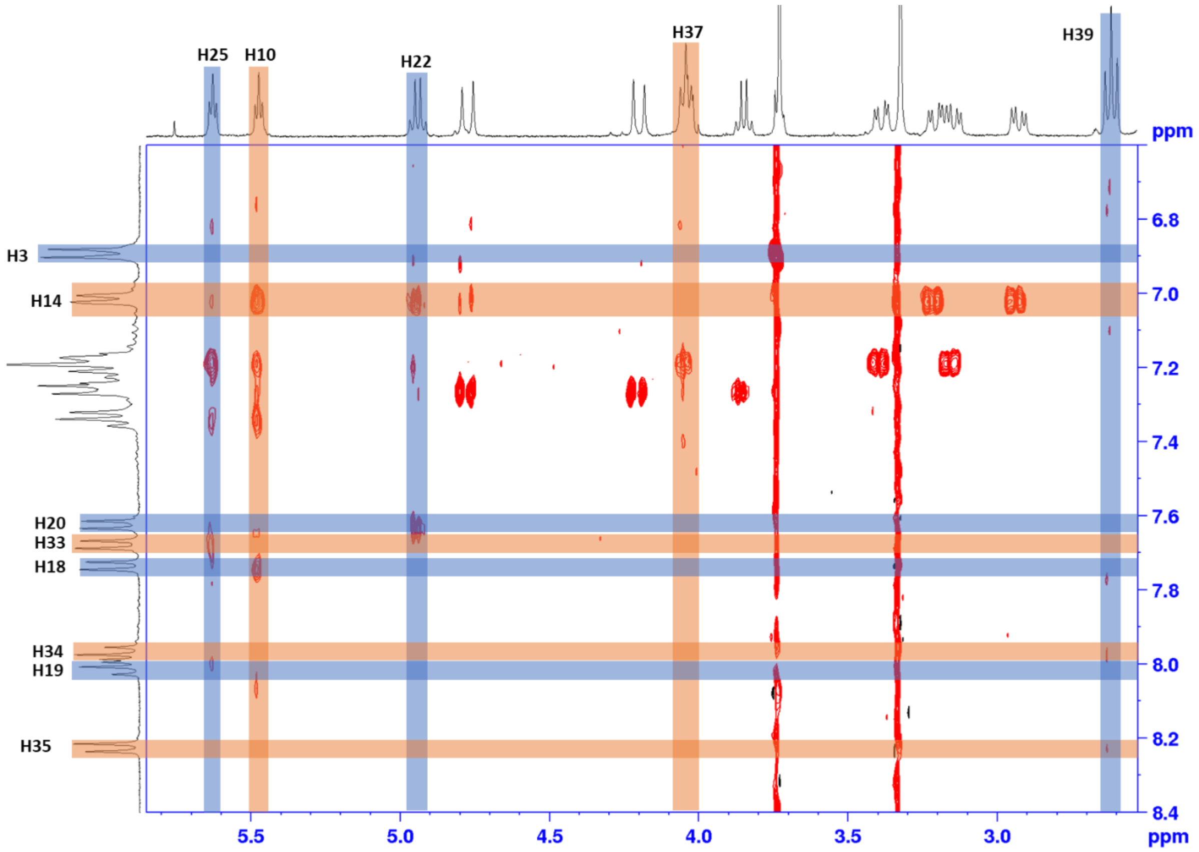

Figure S12: Zoomed view of greatest interest of dimer **6** (*d6*-DMSO)

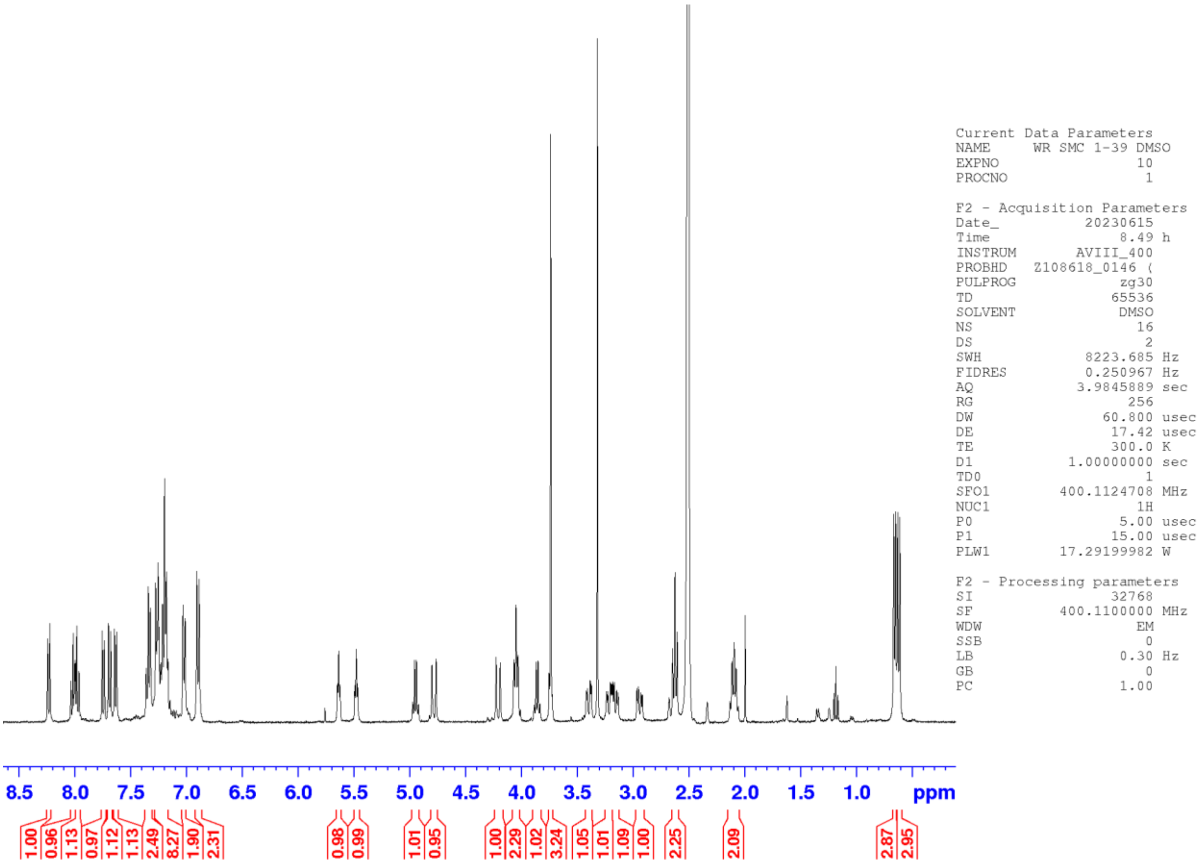

Figure S13: Proton spectrum of dimer **13** (9 mM), in *d6*-DMSO

δ_H (400 MHz, d_6-DMSO): 8.23 (1H, d, *J* 8.2 Hz, **H35**), 8.01 (1H, t, *J* 8.1 Hz, **H19**), 7.98 (1H, t, *J* 8.1 Hz, **H34**), 7.74 (1H, d, *J* 8.1 Hz, **H18**), 7.69 (1H, d, *J* 8.1 Hz, **H33**), 7.63 (1H, d, *J* 8.0 Hz, **H20**), 7.37-7.32 (2H, m, **H29**), 7.29-7.16 (8H, m, **H4**, **H15-16**, **H30+H31**), 7.02 (2H, d, *J* 7.1 Hz, **H14**), 6.90 (2H, d, *J* 8.6 Hz, **H3**), 5.64 (1H, t, *J* 4.8 Hz, **H25**), 5.48 (1H, t, *J* 4.7 Hz, **H10**), 4.95 (1H, q, *J* 7.0 Hz, **H22**), 4.78 (1H, d, *J* 14.7 Hz, **H6'**), 4.21 (1H, d, *J* 14.8 Hz, **H6**), 4.05 (2H, t, *J* 7.6 Hz, **H37**), 3.86 (1H, q, *J* 7.1 Hz, **H7**), 3.79 (3H, s, **H1**), 3.40 (1H, dd, *J* 13.9, 4.1 Hz, **H27'**), 3.22 (1H, dd, *J* 13.9, 4.3 Hz, **H12'**), 3.16 (1H, dd, *J* 14.0, 5.4 Hz, **H27**), 2.94 (1H, dd, *J* 13.9, 5.3 Hz, **H12**), 2.63 (2H, t, *J* 8.1 Hz, **H39**), 2.09 (2H, quint, *J* 7.6 Hz, **H38**), 0.66 (3H, d, *J* 7.0 Hz, **H8**), 0.62 (3H, d, *J* 7.1 Hz, **H23**).

3.4 Trimer 8

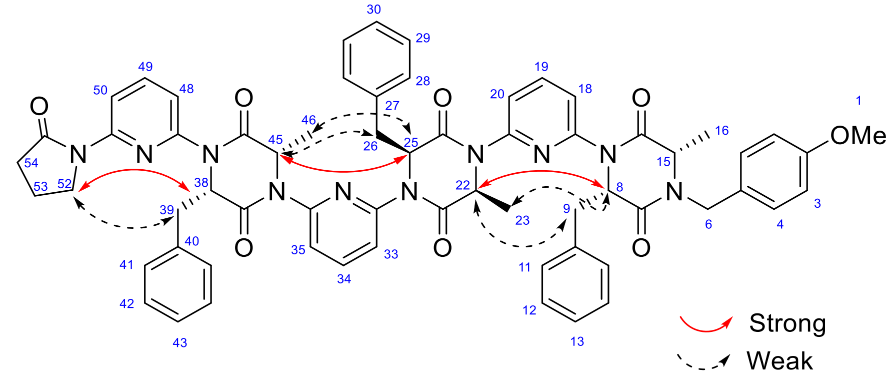

Figure S14: nOe correlations ($CDCl_3$, 600 MHz) from ROESY spectrum of **8** confirms the predicted conformation. Solid red arrows are strong cross-peaks; dashed black arrows are weak cross-peaks.

As observed with the monomer and dimer, the shape continued to be dictated by the dipole repulsion effect as the foldamer was extended. This dipole opposition positioned the pyridine nitrogen *anti* to the pyrrolidinone and adjacent diketopiperazine carbonyl groups. This is evident by the absence of interactions between the pyridine protons and the protons of the pyrrolidinone or diketopiperazine units. Additionally, interactions were observed between the pyrrolidinone and first diketopiperazine unit (**H52↔H38**, **H52↔H39**) and strong interactions between **H22↔H8** and **H45↔H25**, (**Figures S15-S17**) although, the quartets for **H45** and **H22** overlap, meaning the interaction could be attributed to an intra-DKP nOe (**H25↔H22**), as other intramolecular DKP nOes within the units have not been detected, it would suggest this is an inter-DKP nOe. Therefore, the zig-zag shape is clearly displayed in the trimer and is created through the independent control of linker groups due to the extra diketopiperazine carbonyl group. Other very weak interactions between neighbouring diketopiperazine units (**H45↔H26**, **H46↔H25**, **H22↔H9**, and **H23↔H8**), (**Figures S18-S19**) also help confirm this conformation, though some are so weak that they may be attributed to baseline noise rather than a true correlation. Nonetheless, if a dipole-aligned *syn* conformation were to be adopted, we would expect these interactions to be significantly stronger and more distinguishable from noise.

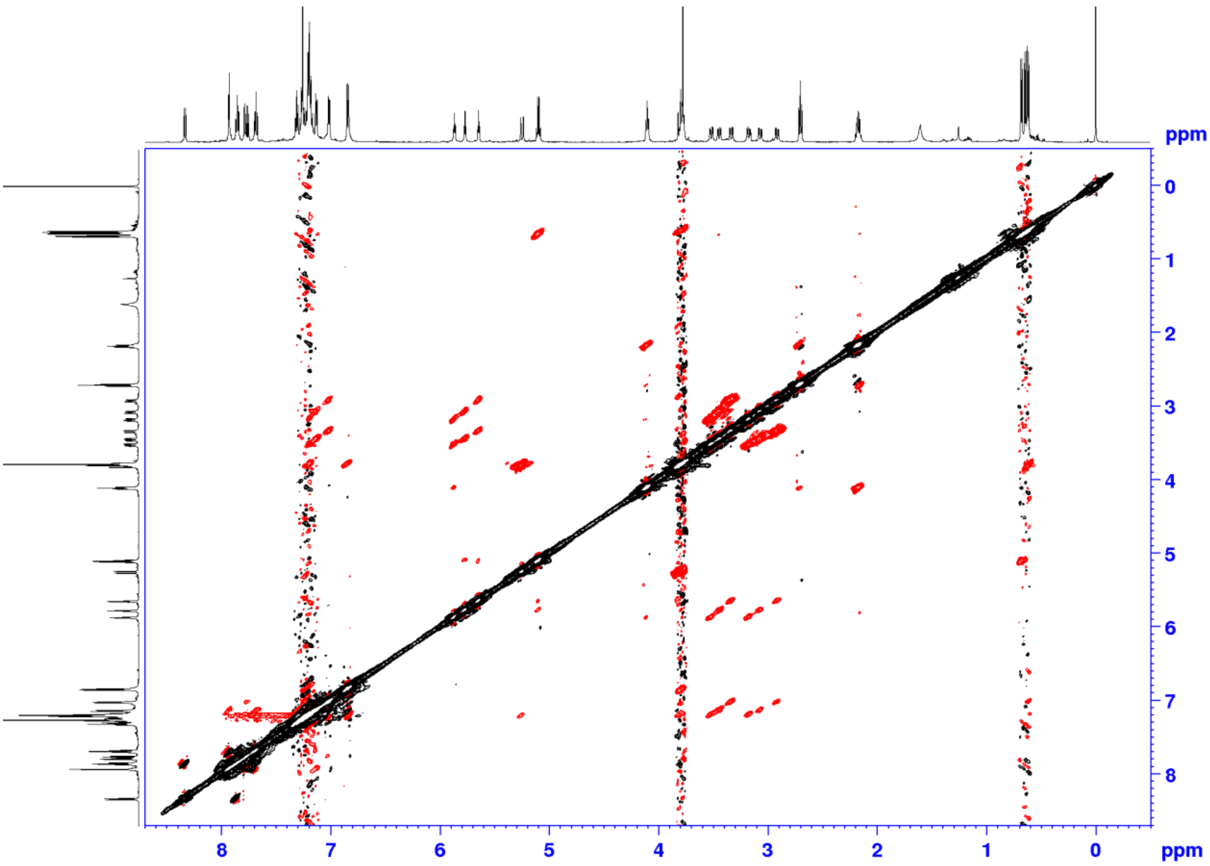

Figure S15: Full ROESY spectrum of trimer **8**, with strong nOe's

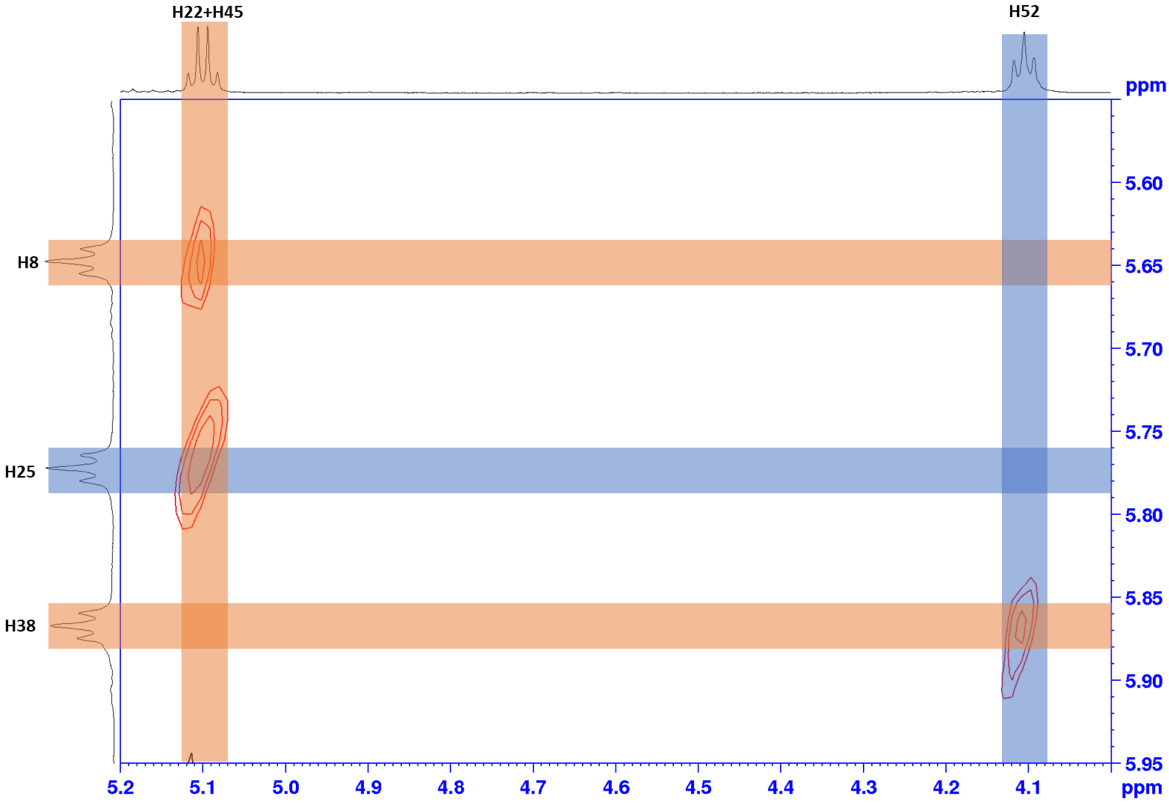

Figure S16: Zoomed ROESY spectrum of trimer **8**, showing the strong interactions between neighbouring diketopiperazine units

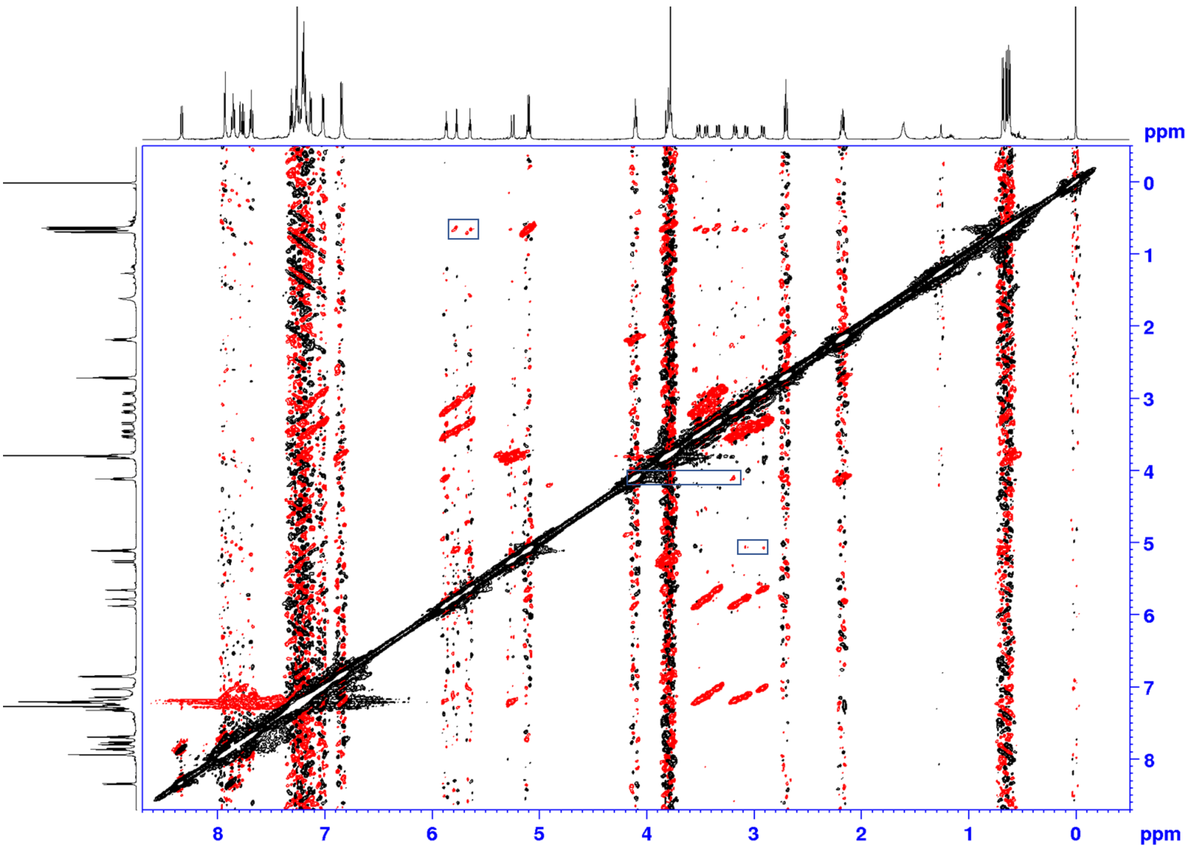

Figure S17: Full ROESY spectrum of trimer **8**, with weak interactions

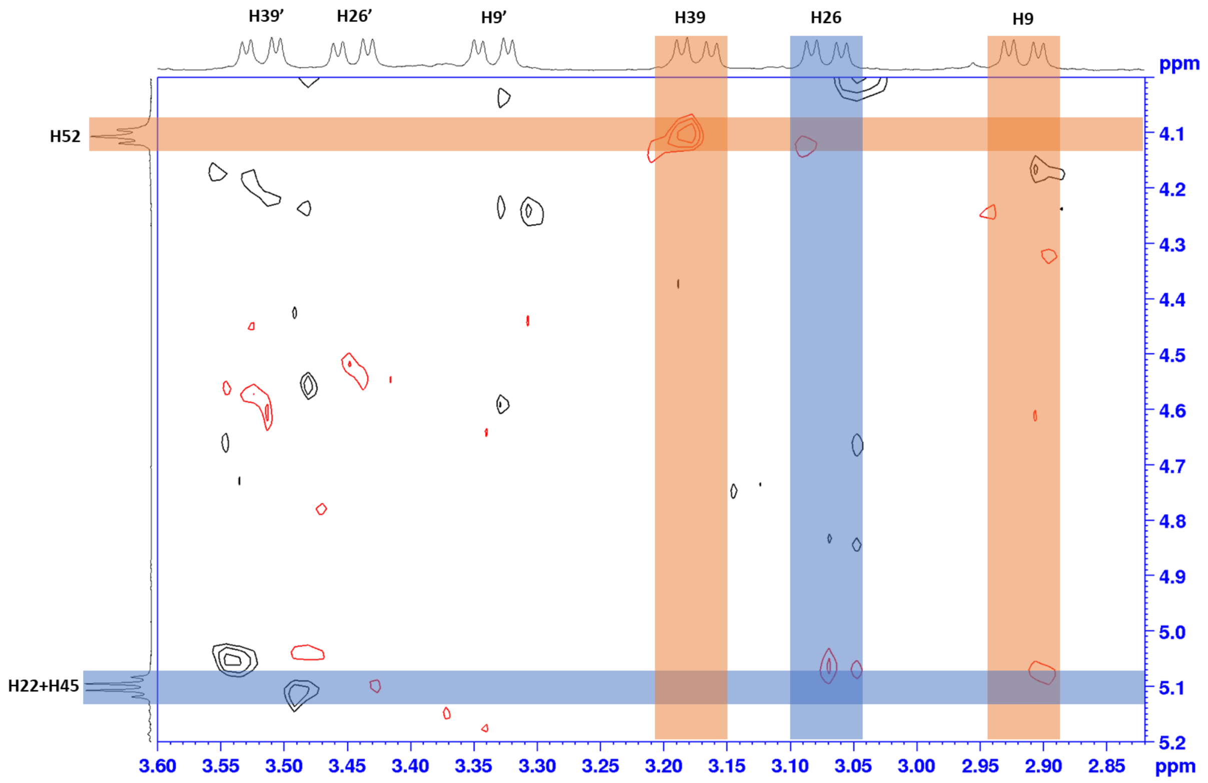

Figure S18: Zoomed ROESY spectrum of trimer **8**, showing the weak interactions between neighbouring diketopiperazine units and benzyl CH_2’s

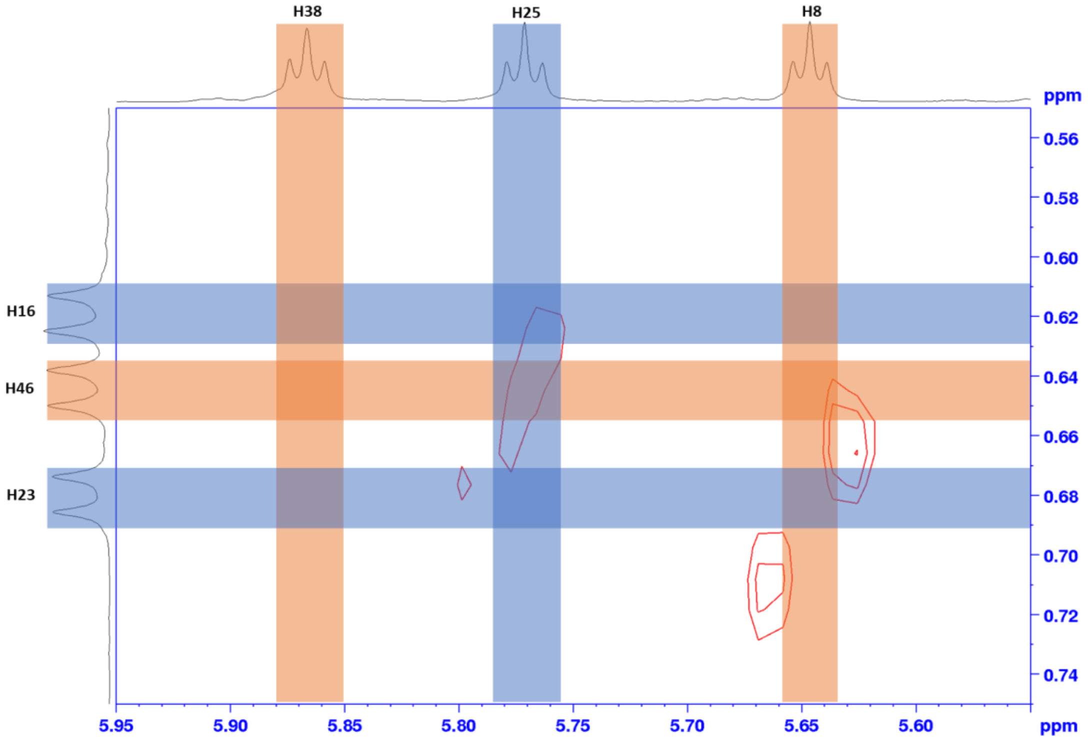

Figure S19: Zoomed ROESY spectrum of trimer **8**, showing the weak interactions between neighbouring diketopiperazine Me and α-CH’s

4 Solution-Phase Conformation of Carboxylic Acid Bearing DKPs

4.1 General Comments

All ROESY data were acquired on a Bruker 400 MHz spectrometer with a mixing time (t_{mix}) of 200 ms for ROESY data, in specified solvent at room temperature. For the solution-phase conformational study for dimer **13**, all sample in specified solvent were run at 5 mM concentration to allow for parity between the interactions. Full ROESY spectra at two levels of zoom are given, followed by a zoomed view of the cross-peak region of greatest interest. Integrations were carried out manually. Where coloured bands are placed over spectra and with alternating orange/blue colouration, this is done to aid visualization and the colours are arbitrary (unless otherwise stated). Since some of the signals examined are very weak, bands of noise are frequently present at similar levels of intensity.

4.2 Monomer 10a

Monomer 10a in $CDCl_3$

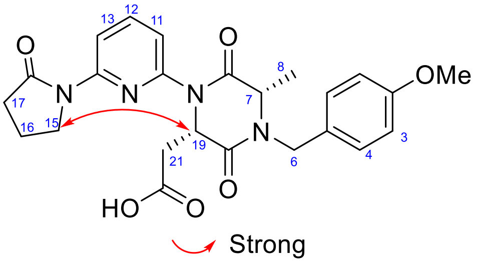

Figure S20: nOe correlations ($CDCl_3$ 400 MHz) from ROESY spectrum of **10a**, solid red arrows are strong cross-peaks; dashed black arrows are weak cross-peaks.

For monomer **10a** in $CDCl_3$, very few key nOes were present, with the only strong interaction being between **H15↔H19**, and no interaction between $C_α$-H of the DKP and *meta*-pyridine being observed. (**Figure S21**) These interactions and lack thereof are consistent with DKP monomer adopting a dipole-opposed conformation, as in a dipole-unopposed conformation, the pyridine moiety would like much closer to the $C_α$-H of the DKP, resulting in a much strong nOe, like those see within the acid-switched conformations for **6, 13,** and **8**. See **Section 5**.

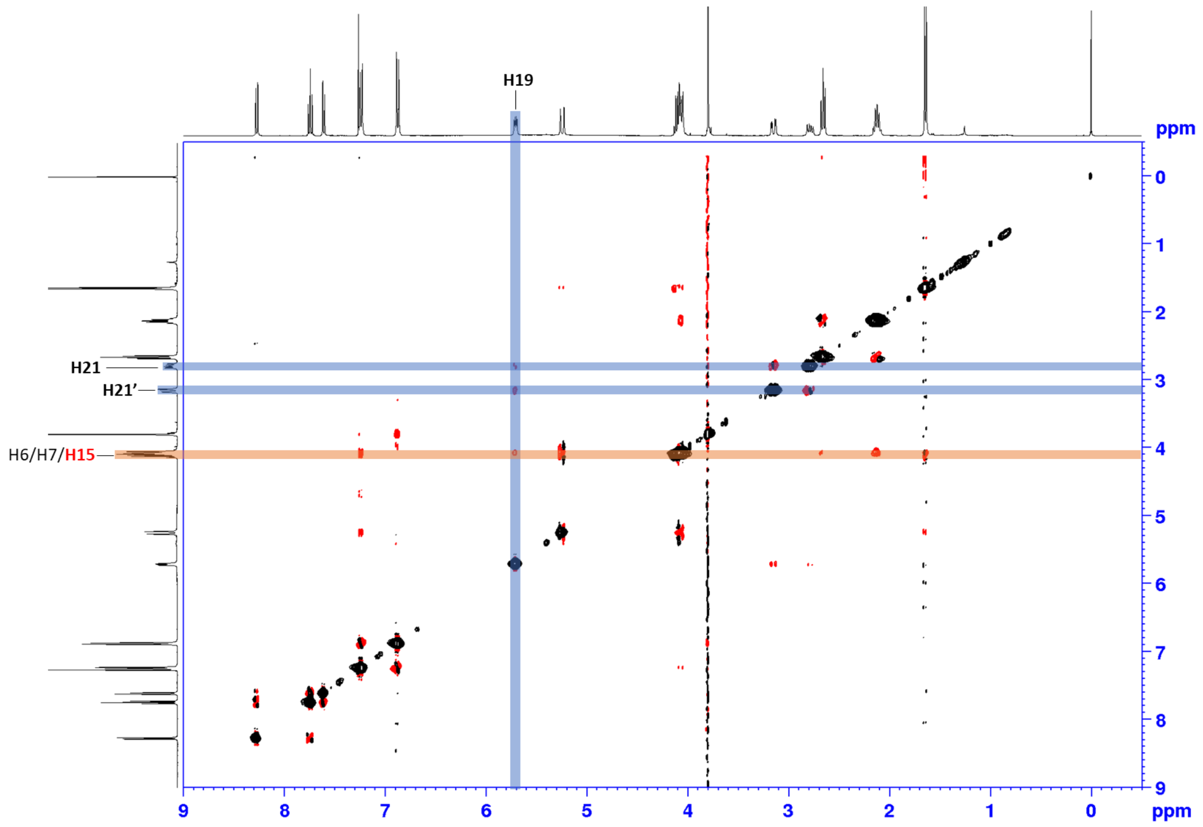

Figure S21: Full ROESY spectrum of monomer **10a**, in $CDCl_3$

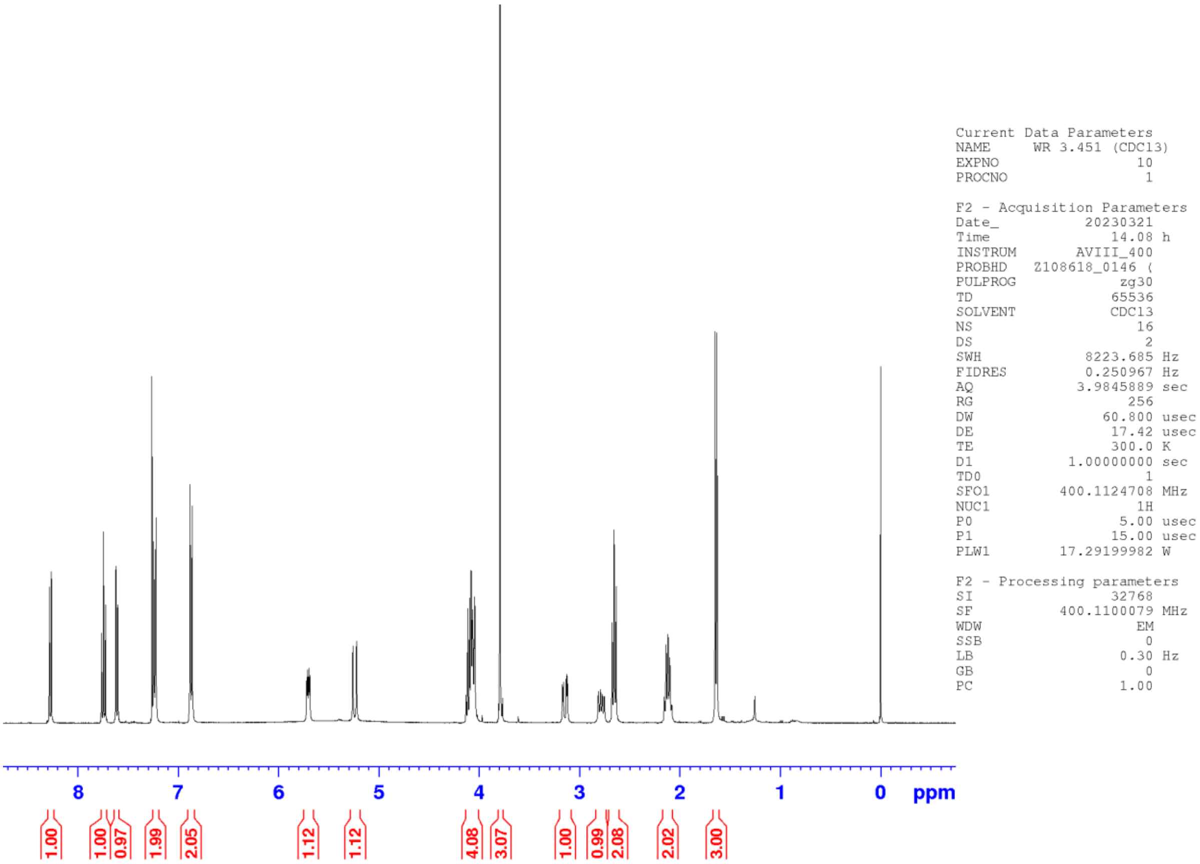

Figure S22: Proton spectrum of monomer **13a** in $CHCl_3$

δ_H (400 MHz, $CDCl_3$): 8.27 (1H, d, *J* 8.3 Hz, **H13**), 7.74 (1H, t, *J* 8.1 Hz, **H12**), 7.60 (1H, d, *J* 8.1 Hz, **H11**), 7.24 (2H, d, *J* 8.7 Hz, **H4**), 6.88 (2H, d, *J* 8.7 Hz, **H3**), 5.71 (1H, dd, *J* 8.1, 3.8 Hz, **H19**), 5.24 (1H, d, *J* 14.9 Hz, **H6'**), 4.15-4.02 (4H, m, **H6+H7+H15**), 3.80 (3H, s, **H1**), 3.14 (1H, dd, *J* 15.6, 3.8 Hz, **H21'**), 2.79 (1H, dd, *J* 15.6, 8.2 Hz, **H21**), 2.66 (2H, t, *J* 8.1 Hz, **H17**), 2.12 (2H, quint, *J* 7.7 Hz, **H16**), 1.64 (3H, d, *J* 7.1 Hz, **H8**).

Monomer 10a in *d*6-DMSO

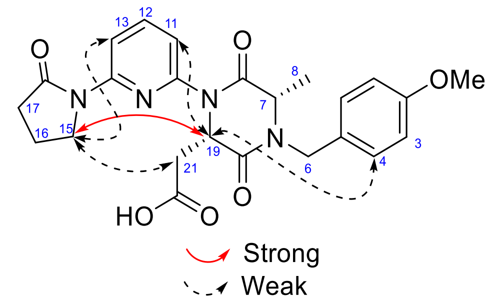

Figure S23: nOe correlations (*d*6-DMSO 400 MHz) from ROESY spectrum of **10a**, solid red arrows are strong cross-peaks; dashed black arrows are weak cross-peaks.

Carboxylic acid bearing monomer **10a** in *d*6-DMSO displayed an extremely similar ROESY spectrum to benzyl bearing monomer **4**, with nOe's being observed between the pyrrolidinone and adjacent diketopiperazine hydrogen atoms, with a strong interaction between **H15↔H19** and slightly weaker one between **H15↔H21**. (**Figures S24-S25**) In addition, very weak nOe interactions were observed between the diketopiperazine and pyridine (**H11↔H19**) and the pyrrolidinone and pyridine protons (**H13↔ H15**), (**Figure S26**) further demonstrating that monomer **10a** is adopting a dipole-opposed conformation with the nitrogen group *anti* to the adjacent carbonyl groups. This also demonstrated that the dipole repulsion-mediated conformation control was still being retained in a much more polar *d*6-DMSO, demonstrating good conformational rigidity of the backbone.

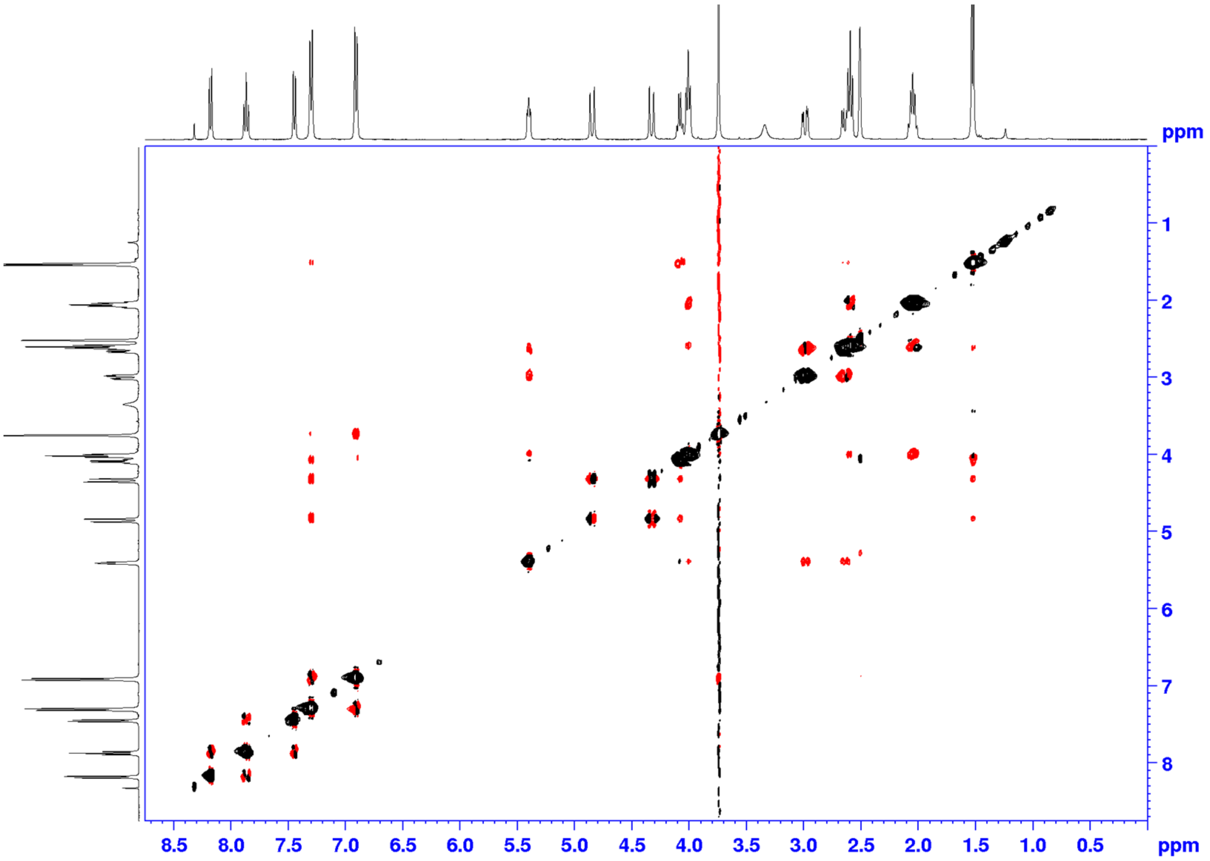

Figure S24: Full ROESY spectrum of monomer **10a**, in d_6-DMSO

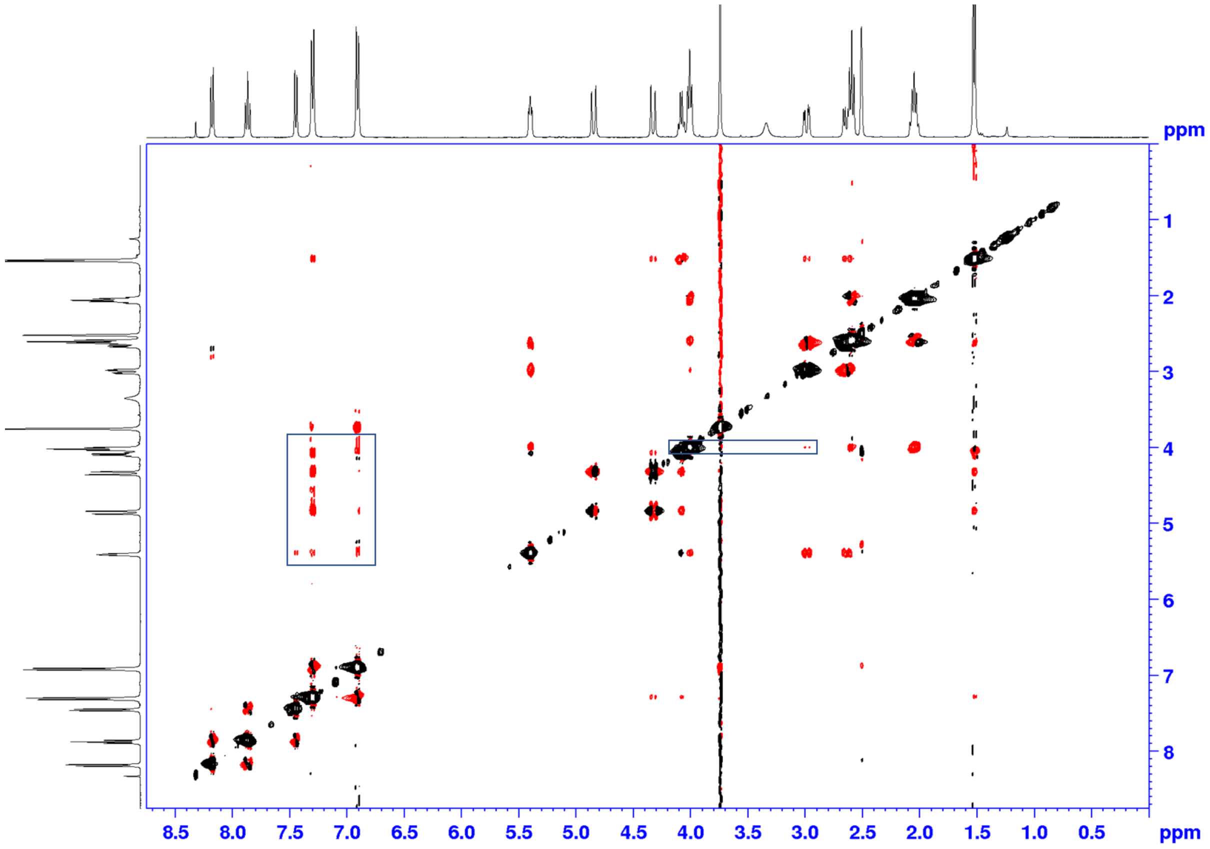

Figure S25: Full ROESY spectrum of monomer **10a**, with weak interactions in d_6-DMSO

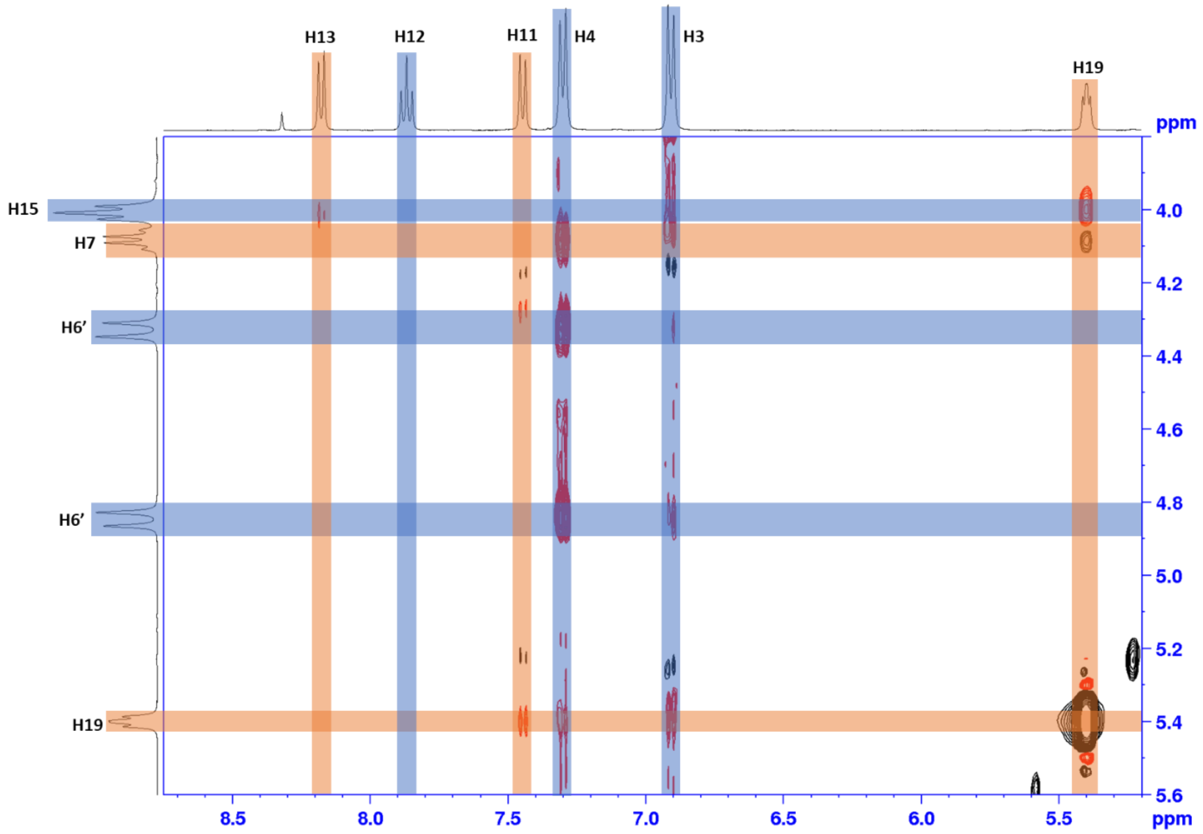

Figure S26: Zoomed view of greatest interest of monomer **10a**, in d_6-DMSO

We also then wished to analyse the conformation in a more polar solvent to determine if the conformation would be retained or interrupted, therefore attempts were made to analyse the conformation in D_2O or buffer solution ($NaDCO_3$/Na2CO_3 in D_2O), but monomer **10a** was insoluble in both.

4.3 Dimer 13

Dimer 13 in $CDCl_3$

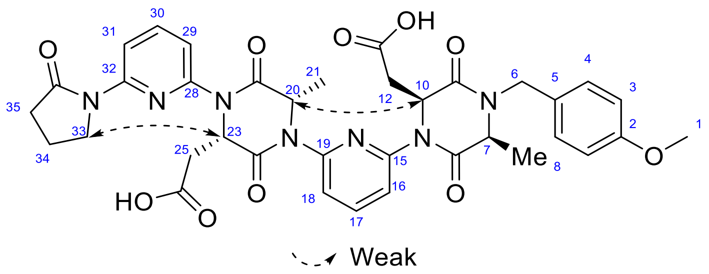

Figure S27: nOe correlations ($CDCl_3$, 400 MHz) from ROESY spectrum of **13** (5 mM), dashed black arrows are weak cross-peaks.

Looking at the nOe interaction for dimer **13**, weak interactions were observed between the pyrrolidinone and adjacent diketopiperazine hydrogen (**H33↔H23**), however, unlike previous

DKP foldamer examples, no interaction could be observed between (**H33↔H25**). (**Figures S28-S29**) The presence of the **H33↔H23** nOe interaction is consistent with the pyrrolidinone and diketopiperazine carbonyl groups being positioned *anti* to the pyridine nitrogen atom, which is to be expected in the dipole-opposed conformation. (**Figures S28-S29**) Further evidence of the dipole-opposed conformation can be seen in the inter-residue interaction between (**H20↔H10**), which would be absent in a dipole-unopposed conformation. Secondly, nOe interactions between C_α-H of the DKP and *meta*-pyridine were absent for dimer **13**, which is in good agreement with dipole opposed conformation, as demonstrated by the acid-switching conformation studies (**Section 5**), which displayed intense DKP and *meta*-pyridine nOes.

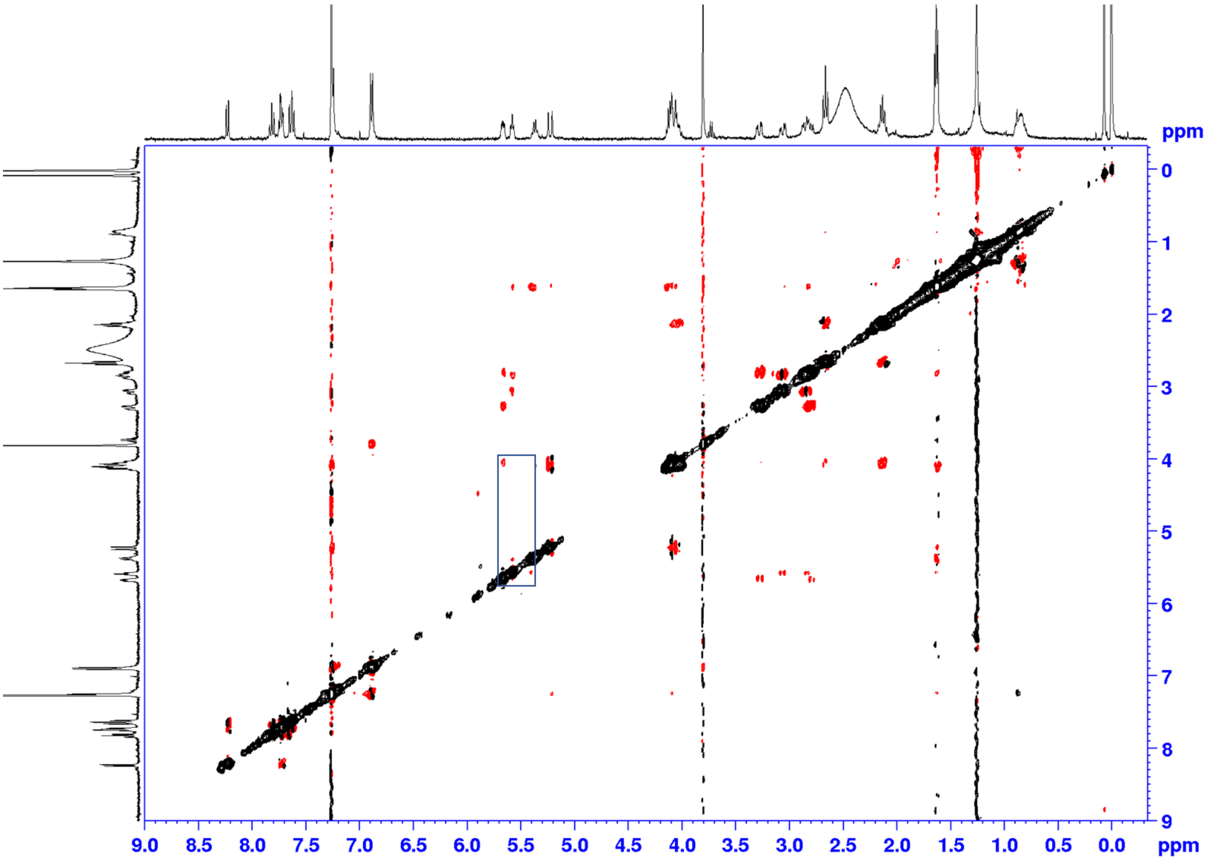

Figure S28: Full ROESY spectrum of dimer **13** (5 mM), with inter-DKP interactions in $CDCl_3$

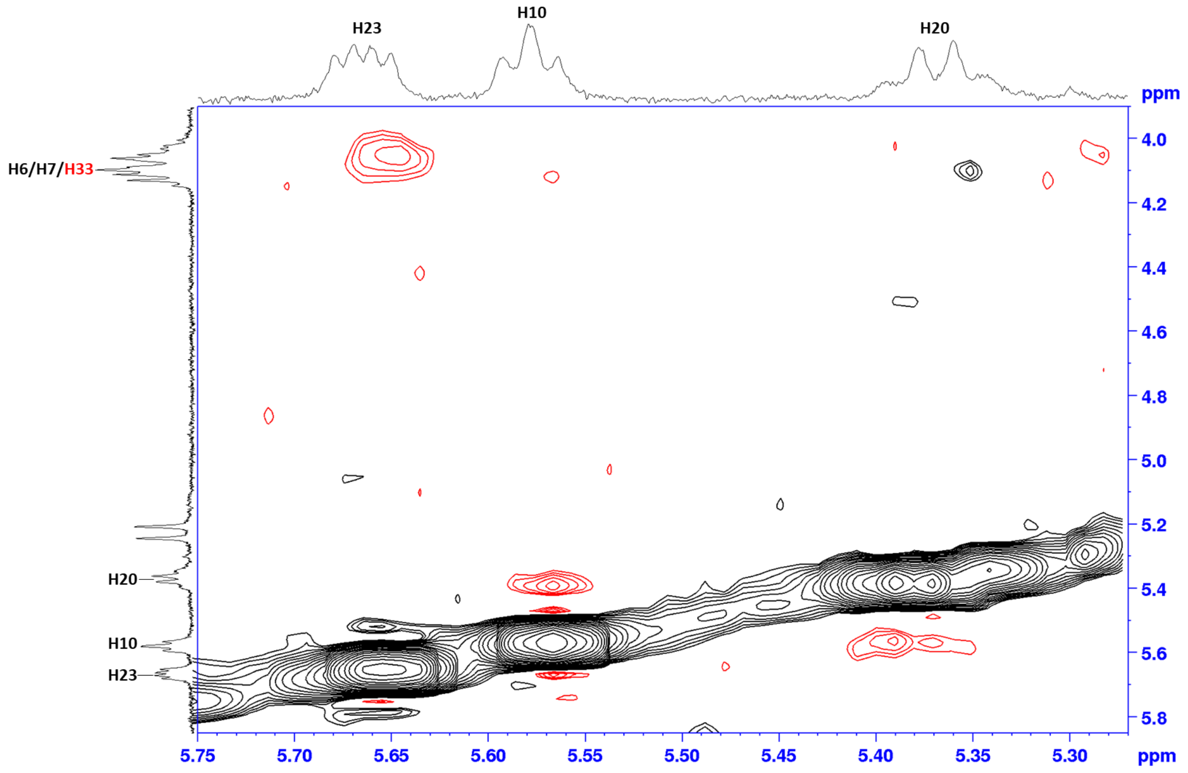

Figure S29: Zoomed ROESY spectrum of dimer **13** (5 mM), with inter-DKP interactions in $CDCl_3$

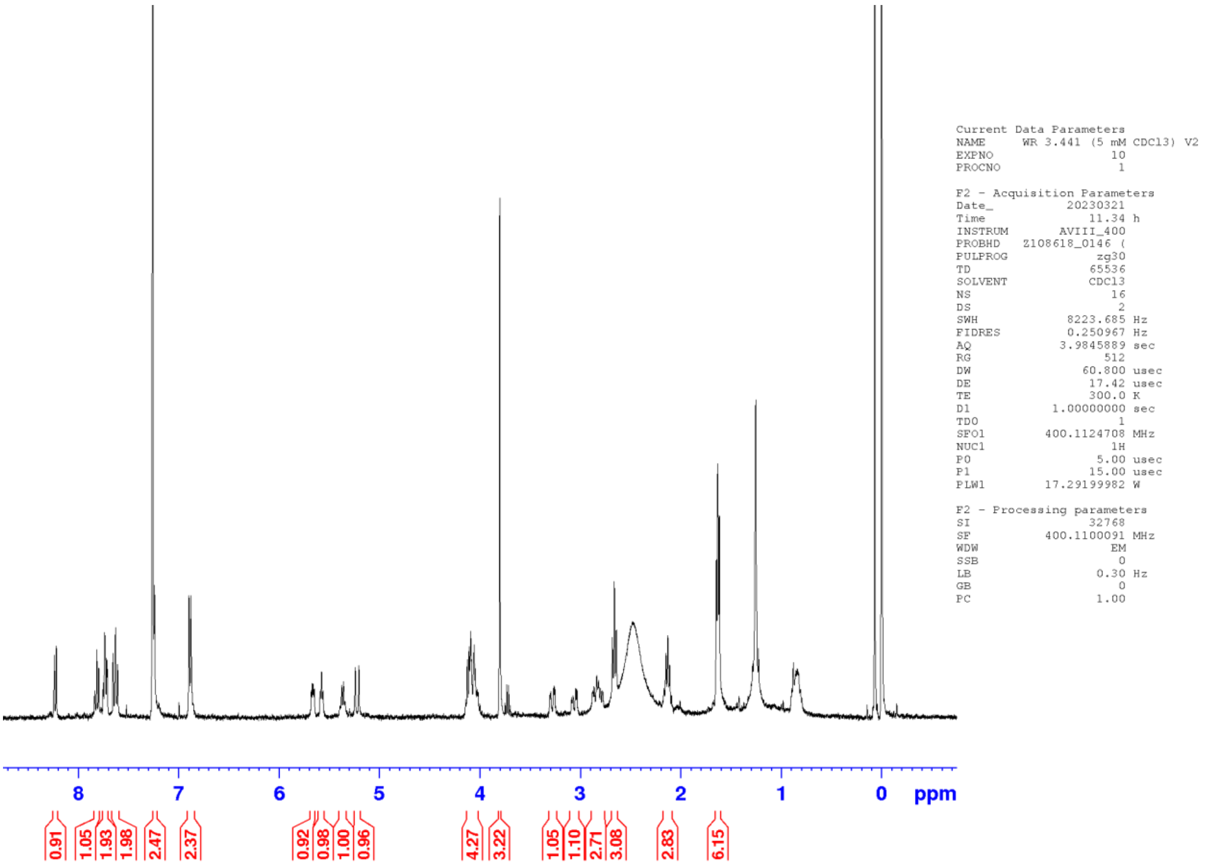

Figure S30: Proton spectrum of dimer **13** (5 mM) in $CDCl_3$

δH (400 MHz, $CDCl_3$):* 8.22 (1H, d, *J* 8.1 Hz, **H31**), 7.82 (1H, t, *J* 8.1 Hz, **H17**), 7.76-7.71 (2H, m, **H18+H30**), 7.66-7.60 (2H, m, **H29+H16**), 7.25 (1H, d, **H4**), 6.89 (1H, d, *J* 8.6 Hz, **H3**), 5.66 (1H, dd, *J* 7.8, 4.0 Hz, **H23**), 5.58 (1H, t, *J* 5.8 Hz, **H10**), 5.37 (1H, q, *J* 7.2 Hz, **H20**), 5.22 (1H, d, *J* 14.7 Hz, **H6'**), 4.15-4.00 (4H, m, **H6+H7+H33**), 3.80 (3H, s, **H1**), 3.28 (1H, dd, *J* 15.2, 3.6 Hz, **H25'**), 3.07 (1H, dd, *J* 15.6, 5.1 Hz, **H12'**), 2.90-2.76 (2H, m, **H25+H12**), 2.66 (1H, t, *J* 8.1 Hz, **H35**), 2.13 (1H, quint, *J* 7.6 Hz, **H34**), 1.66-1.60 (6H, m, **H8+H21**).

4.3.1 *d*6-DMSO and D_2O Influences on Conformational Control for Diacid 13

Following the solution phase conformation study in $CDCl_3$ (dielectric constant ε 4.8), the conformation of **13** was then analysed in more polar solvent analysis in *d*6-DMSO (ε 46.7) and D_2O (ε 78.3) to see if the conformation control would be affected. Solubility and retention of conformation in D_2O is of particular interest due to known biomimetic capabilities of foldamer such as molecular recognition (PPIs),[14] signalling,[15] and molecular transport.[16] Therefore solubility in a biologically relevant media is of interest for examining the potential capabilities *in vivo*. In addition, polar protic solvents such as water can disrupt the conformation/structure of foldamers controlled by intramolecular hydrogen-bonding networks or dipolar-repulsion. Therefore, the ability to retain conformation control in a highly polar solvent is vital to maintain the activity of the foldamer, should it be modified to display bio-mimetic properties.

Dimer 13 in *d*6-DMSO

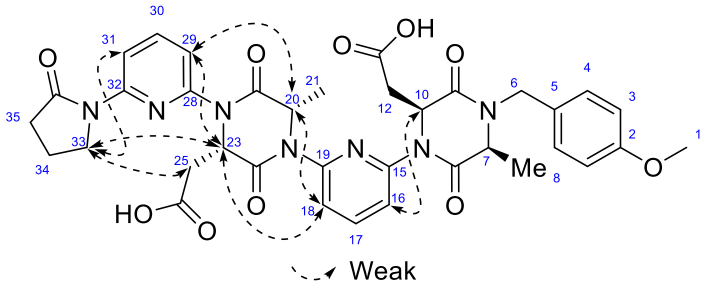

Figure S31: nOe correlations (*d*6-DMSO, 400 MHz) from ROESY spectrum of **13** (5 mM), dashed black arrows are weak cross-peaks.

As seen with all the DKP foldamers, and consistent with dimer **13** in $CDCl_3$, an nOe interaction was observed between the pyrrolidinone and adjacent diketopiperazine hydrogen atoms (**H33**↔**H23**), but the nOe between **H33**↔**H25** was faint, and hard to distinguish from noise.

* Coupling constant for **H4** has not been stated due to co-incidence with residual $CHCl_3$ peak. In addition, slight over-integration is present in the regions between 2.90-2.00 due to the appearance of a large unknown broad peak

(**Figures S32-S34**) This is consistent with retention of the dipole-opposed conformation, about *N*-C32 and *N*-C28 with the pyrrolidinone and diketopiperazine carbonyl groups positioned *anti* to the pyridine nitrogen atom. Unfortunately, due to the proximity of **H10** (5.33 ppm) and **H20** (5.28 ppm), potential nOe interactions were not discernible to further demonstrate inter-DKP interactions.

In addition, very faint nOe interactions between the C_α-H(CH_2CO_2H) and *meta*-pyridine peaks, (**H23↔H29**, **H23↔H18**, **H10↔H16**) as well as **H33↔H31** were observed. (**Figure S34**) Although, these nOe interactions were not observed in $CDCl_3$, suggesting that DMSO resulted in a slight disruption to the conformation with increased rotation about the *N*-C32, *N*-C28, and *N*-C19 bonds. Similar faint nOe's were also visible for dimer **6** in $CDCl_3$ and therefore, the weak nature of these correlations is consistent with the foldamer significantly populating the dipole opposed conformation, though their presence does suggest increased rotation relative to the study in $CDCl_3$.

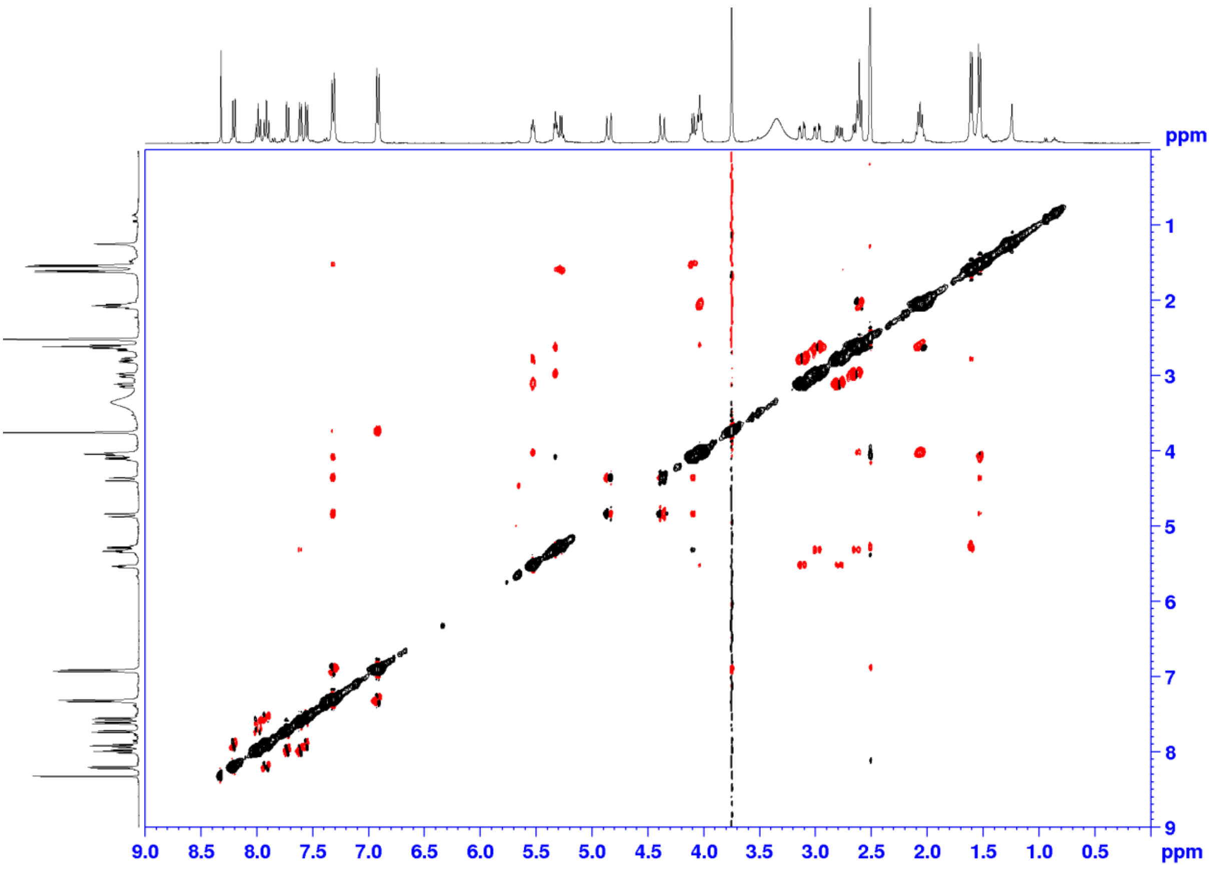

Figure S32: Full ROESY spectrum of dimer **13** (5 mM), in d_6-DMSO

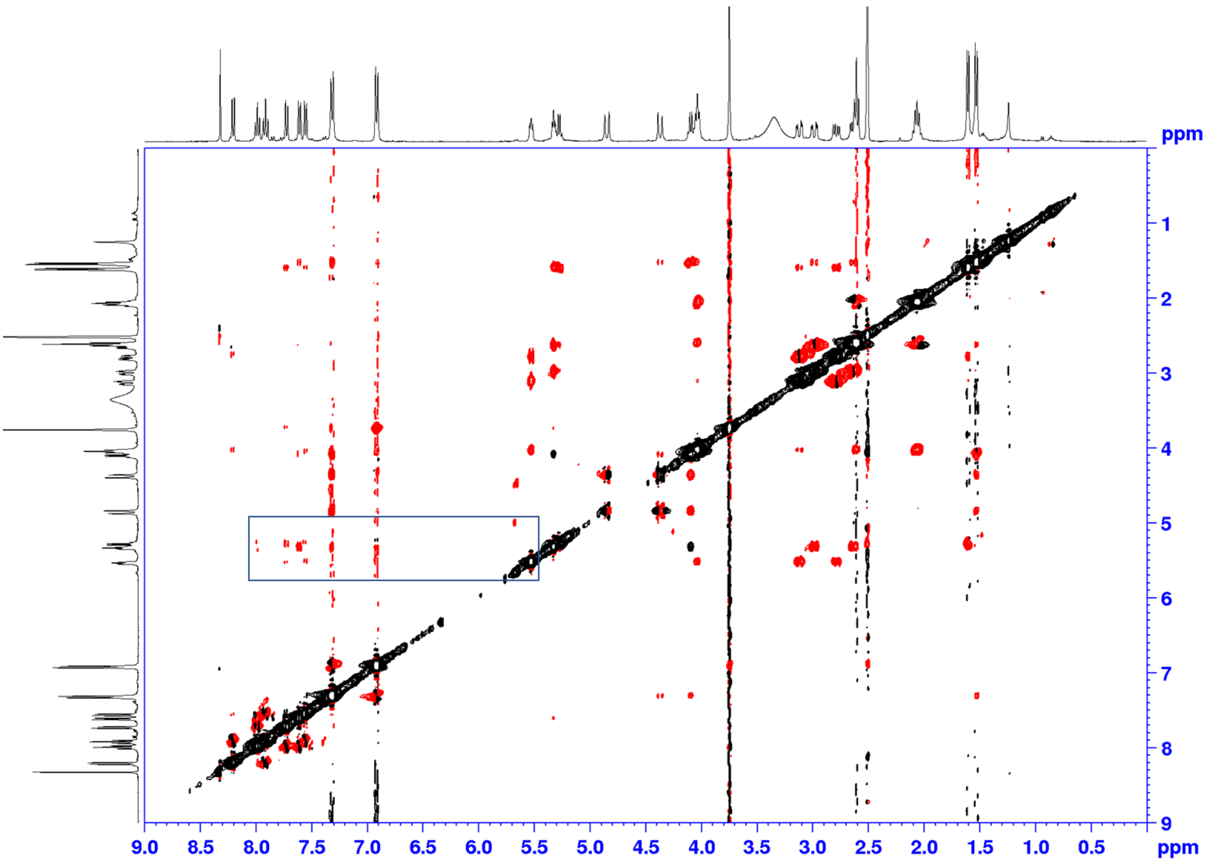

Figure S33: Full ROESY spectrum of dimer **13** (5 mM), with weak interactions in d_6-DMSO

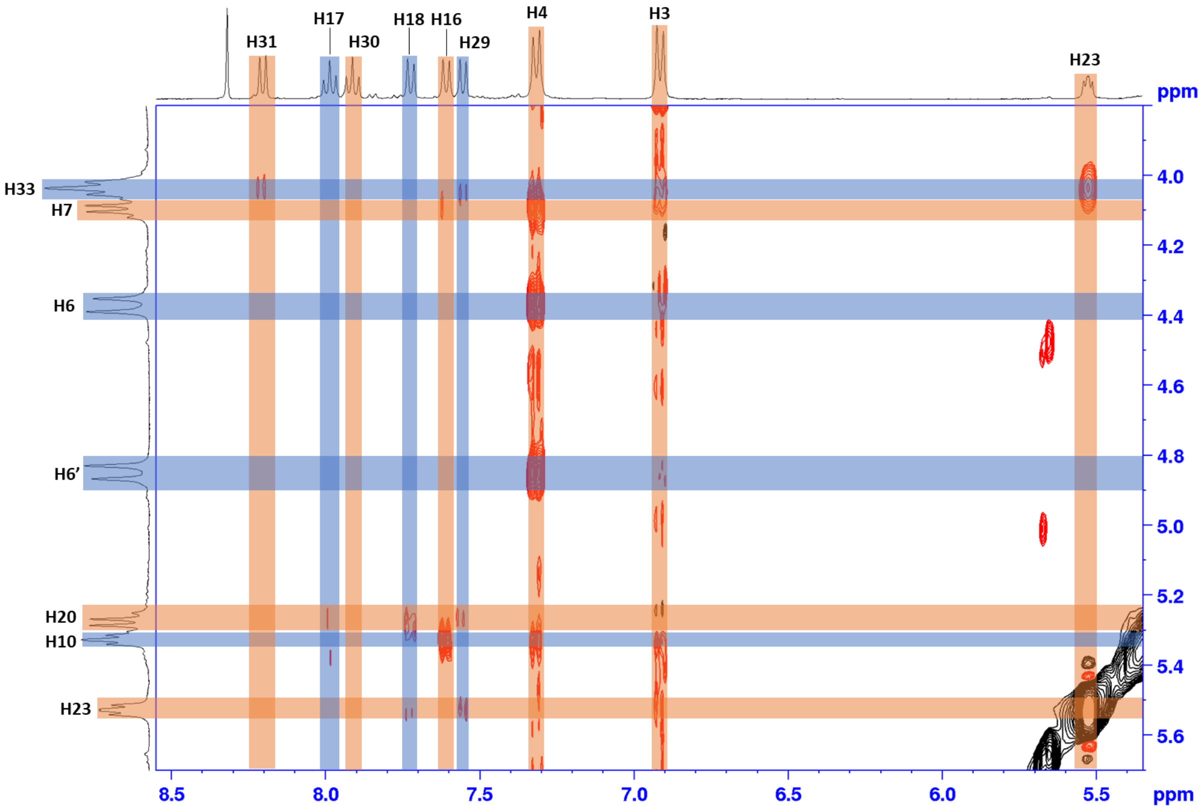

Figure S34: Zoomed view of greatest interest of dimer **13** (5 mM), in d_6-DMSO

Dimer 13 in D_2O buffer ($NaDCO_3$/Na_2CO_3 [0.1 M]; pH 9.5)

Following the conformational study in d_6-DMSO, the conformation was then analysed in D_2O. Under neutral conditions, dimer **13** was insoluble, however, solubility was achieved under basic conditions using an aqueous buffer of $NaDCO_3$/Na_2CO_3 [0.1 M]; pH 9.5.

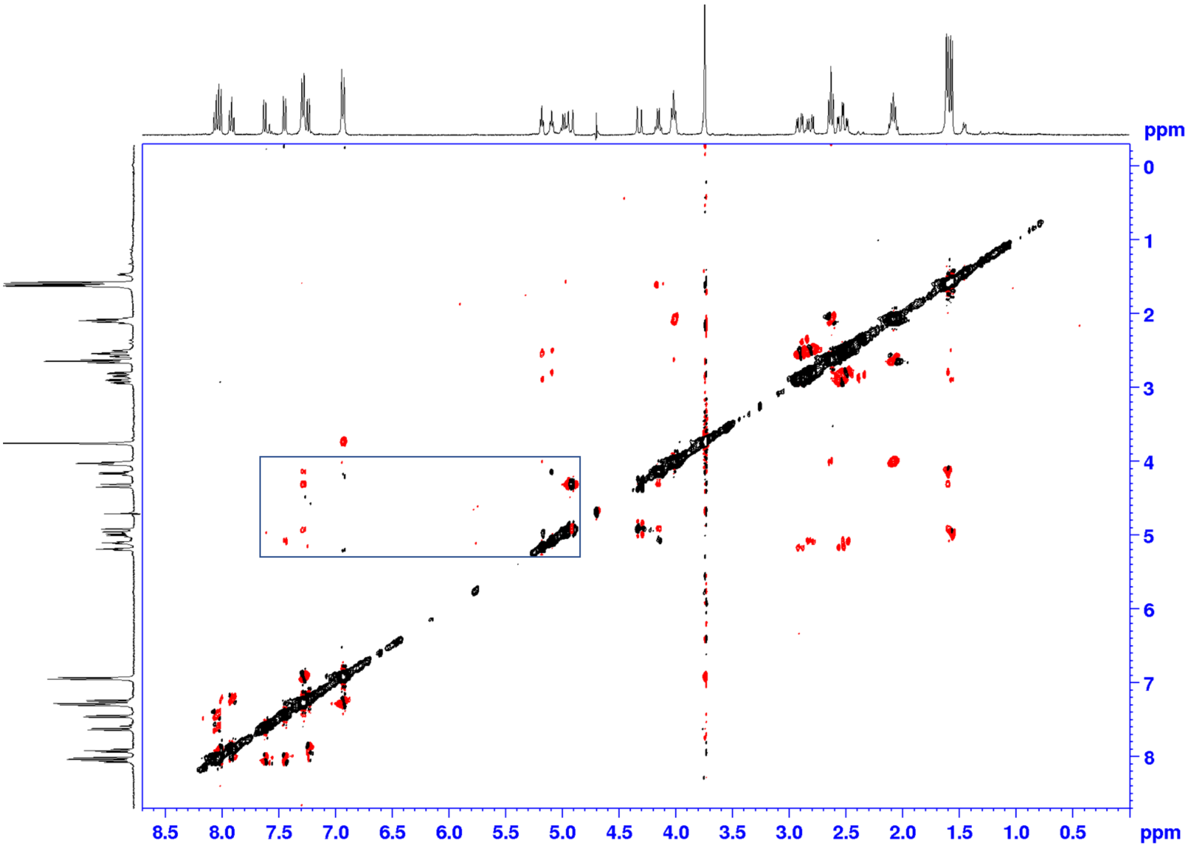

Figure S35: Full ROESY spectrum of dimer **13** (5 mM), with weak interactions in $NaDCO_3$/Na_2CO_3 in D_2O [0.1M]

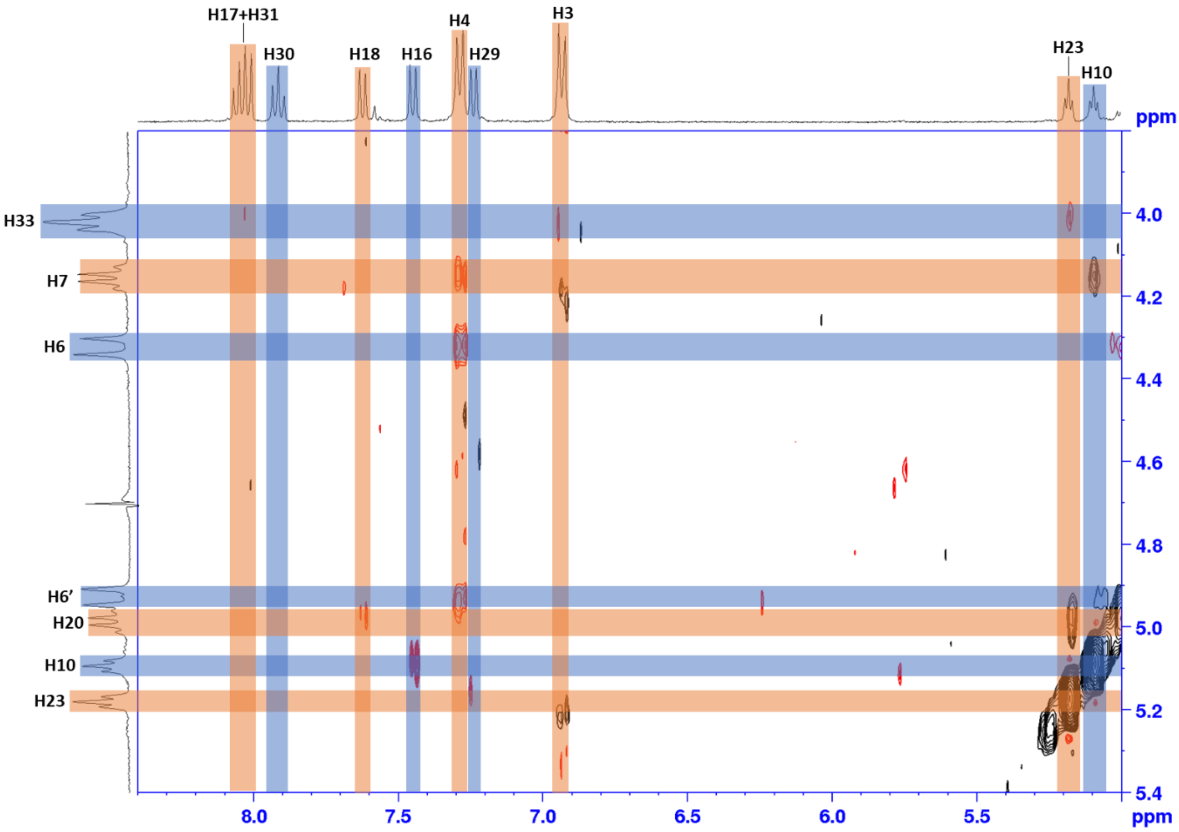

Figure S36: Zoomed view of greatest interest of dimer **13** (5 mM), in $NaDCO_3$/Na2CO3 in D_2O [0.1M]

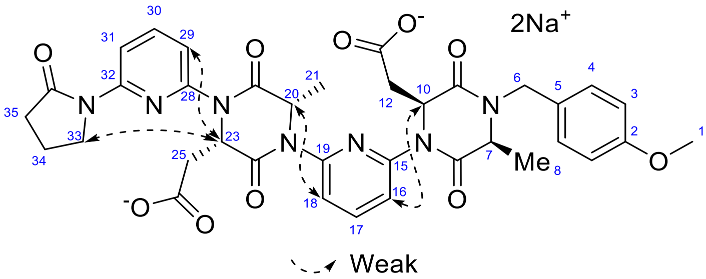

Figure S37: nOe correlations ($NaDCO_3$/Na_2CO_3 in D_2O, 400 MHz) from ROESY spectrum of **13** (5 mM), dashed black arrows are weak cross-peaks.

As with the $CDCl_3$ and d_6-DMSO, in the buffer solution, a weak nOe interaction was observed between the pyrrolidinone and adjacent diketopiperazine hydrogen atoms (**H33↔H23**), while the nOe between **H33↔H25** could not be observed. Furthermore, comparing the conformation with that of DMSO, some of the previously present DKP-*meta*-pyridine interactions were not detected; (**H23↔H18 & H20↔H29**), which might suggest some rotation about *N*-C19. (**Figure 35-36**) Those that were detected became more apparent but still somewhat weak, suggesting a slight loss in conformational control. However, when comparing this to the acid-switched/fully protonated dimer [(*syn*,*syn*)-**13**·2H]$^{2+}$, the interactions are not as strong/intense, suggesting some loss of control but not complete disruption of the conformation. This could

be in response to multiple factors, firstly, the Lewis basic pyridine may be interacting with Na^+ resulting in metal-temptation, and a more *syn*-like conformation. (**Figure S38**)

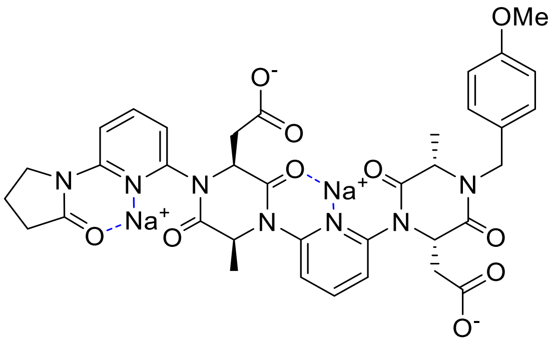

Figure S38: Potential loss of conformational control for **13**, due to coordination to Na^+.

Alternatively, the decrease in conformational rigidity could be in response to the increased solvent dipole moment and hydrogen bonding of D_2O disrupting the dipole-repulsion. To rule out the involvement metal/cation-temptation the use of tetrabutylammonium hydroxide (**TBAOH**) in D_2O was examined, as the far bulkier and less coordinating nature would prevent any form of templation.

Dimer 13 in D_2O (*TBAOH* [10 mM])

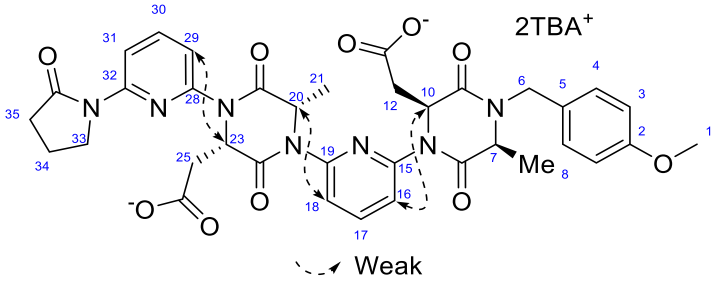

Figure S39: nOe correlations (TBAOH in D_2O, 400 MHz) from ROESY spectrum of **13** (5 mM), dashed black arrows are weak cross-peaks.

Due to it's much more basic nature, only 2 equiv. of TBAOH (10 mM) was required to fully deprotonate the two carboxylic acid moieties, with dimer **13** readily dissolving into an aqueous solution containing the TBAOH.

Comparing the nOe interactions between dimer **13** in the aqueous solution of 0.1M $NaDCO_3$/Na2CO3 via 10 mM TBAOH, visually, there was very little difference in the intensities of the nOe interaction, (**Figure S40-41**) with the only noticeable difference being the absence of the interaction between **H33↔H23**, which might suggest a slight rotation of the pyrrolidinone moiety away from the DKP.

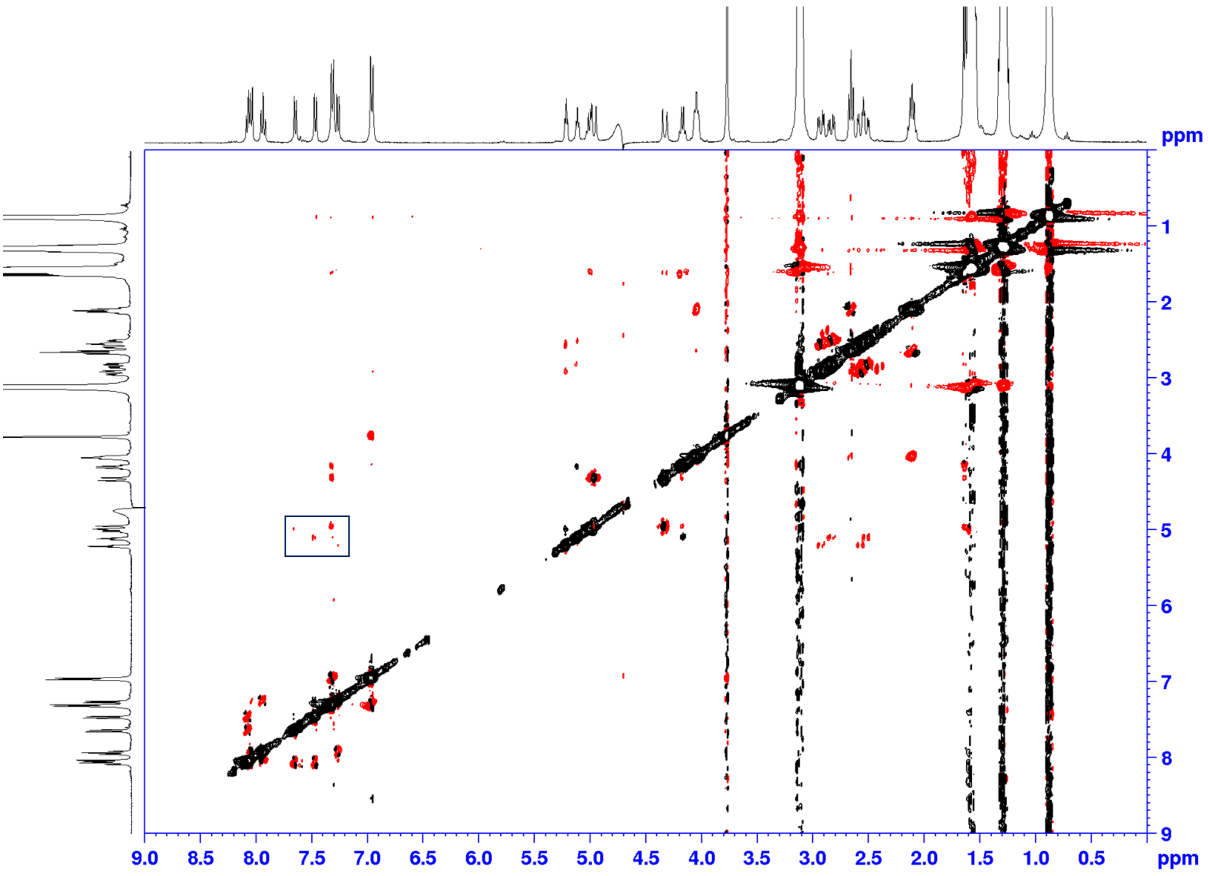

Figure S40: Full ROESY spectrum of dimer **13** (5 mM), with weak interactions in TBAOH in D_2O [10 MM]

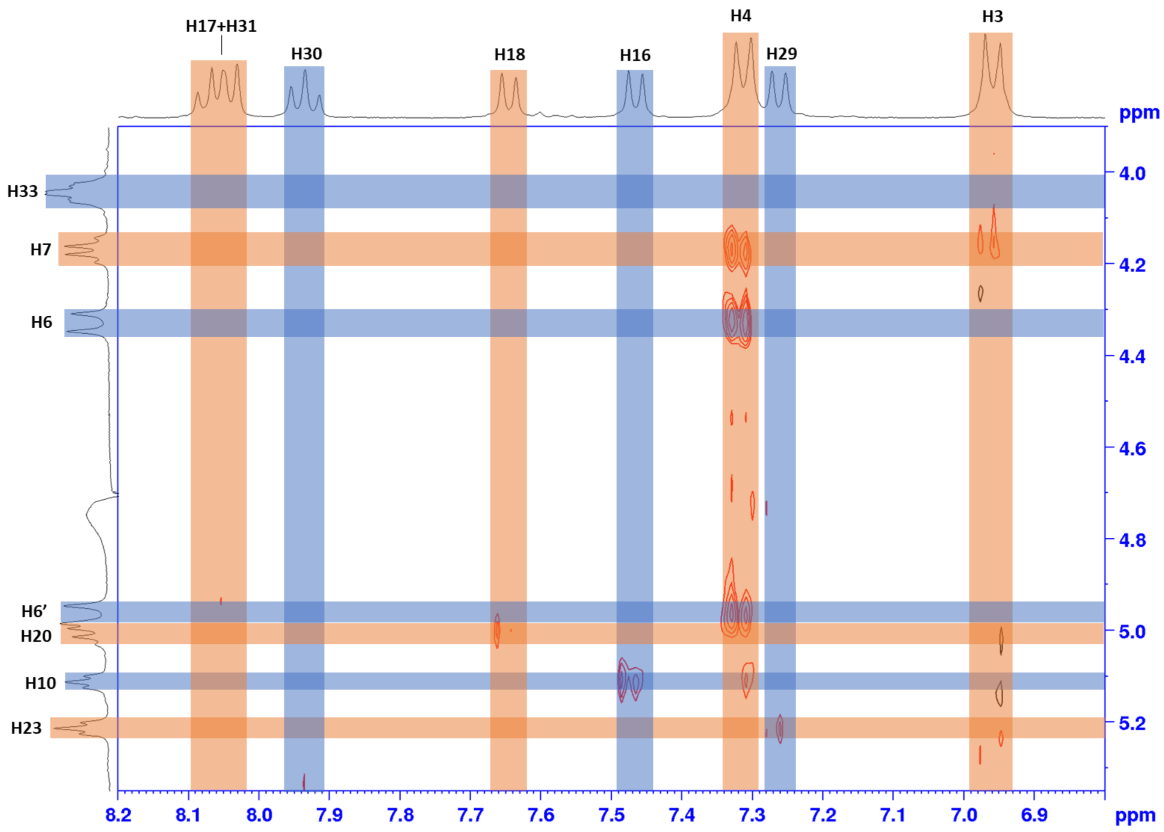

Figure S41: Zoomed view of greatest interest of dimer **13** (5 mM), in TBAOH in D_2O [10 MM]

Therefore, to provide a semi-quantitative comparison between the two aqueous solutions, the cross-peak areas were integrated, with these values being standardised by reference to the geminal interaction between **H35↔H34** since this interaction should remain unaffected by changes in conformational (**Figure S42**). To determine the difference in nOe interactions and therefore relative difference in conformational control, the percentage difference in the integrated area of the TBAOH was compared with the integrated area of the buffered solution, with the sign of the value indicating the improvement (**-**) or loss (+) in conformational control.

$$\%\Delta \text{ in nOe interaction between TBAOH and buffer} = \frac{TBAOH\ (H\leftrightarrow H) - Buffer\ ((H\leftrightarrow H))}{TBAOH\ (H\leftrightarrow H) + Buffer\ ((H\leftrightarrow H))} * 100.$$

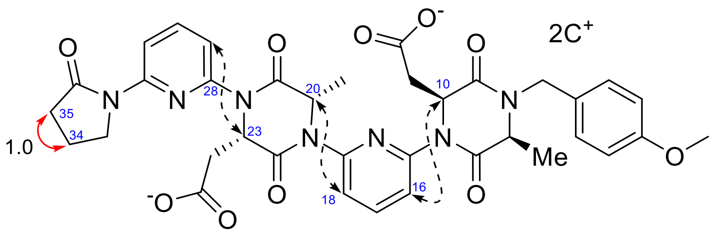

Figure S42: Semi-quantitative measuring of the relative strengths of the nOe interaction of dimer **13** in D_2O

Table 1: Comparison of Dimer **13** acid nOe crosspeak areas when in an aqueous solution of TBAOH 10 mM vs. $NaDCO_3/Na_2CO_3$ [0.1 M]

	**H29↔H23**	**H20↔H18**	**H16↔H10**
$NaDCO_3/Na_2CO_3$ [0.1 M]	0.0646	0.0574	0.1080
TBAOH [10 mM]	0.0693	0.0606	0.1149
Δ % of nOe interactions in TBAOH vs. $NaDCO_3/Na_2CO_3$	+3.5%	+2.7%	+3.1%

Comparing this value (**Table 1**), the nOe's were slightly stronger in the presence of TBAOH vs. $NaDCO_3$/Na2CO$_3$, implying a slight loss in control, but since all interactions remained consistently close (2.7-3.5% difference in integrated areas) this loss wasn't significant. Since tetrabutyl ammonium cation resulted in a slight increase in nOe this therefore indicates that the cation does not have a significant influence on the conformation of the diacetate foldamer. This suggests the more polar nature of D_2O was the primary cause of the observed partial loss in conformational control for both aqueous solutions as compared to the behaviour in $CDCl_3$ or DMSO.

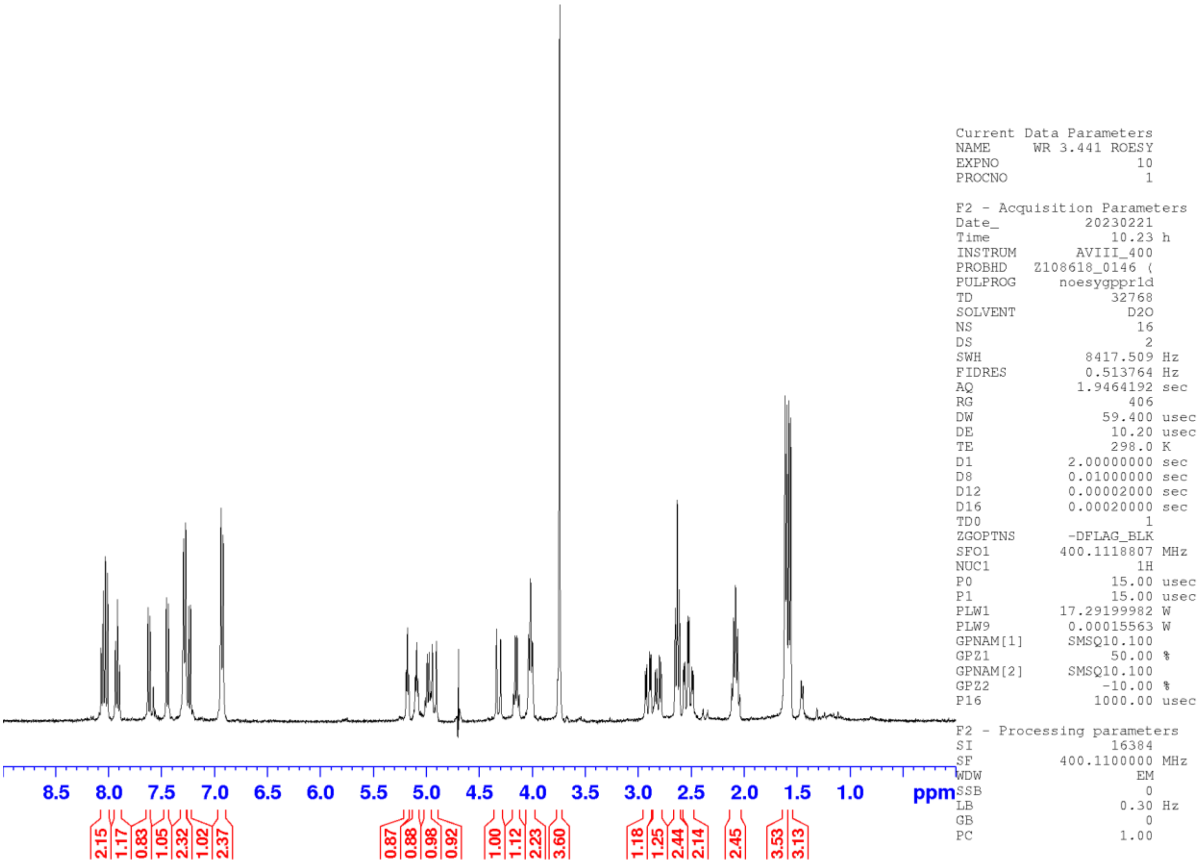

Figure S43: Proton spectrum of dimer **13** (5 mM), in $NaDCO_3$/Na_2CO_3 in D_2O [0.1M]

δ_H (400 MHz, D_2O): 8.07-7.99 (2H, m, **H17+H31**), 7.91 (1H, t, *J* 7.9 Hz, **H30**), 7.62 (1H, d, *J* 7.9 Hz, **H18**), 7.45 (1H, d, *J* 8.0 Hz, **H16**), 7.27 (1H, d, *J* 8.4 Hz, **H4**), 7.24 (1H, d, *J* 7.8 Hz, **H29**), 6.93 (1H, d, *J* 8.5 Hz, **H3**), 5.18 (1H, t, *J* 5.0 Hz, **H23**), 5.09 (1H, t, *J* 5.0 Hz, **H10**), 4.98 (1H, q, *J* 6.9 Hz, **H20**), 4.92 (1H, d, *J* 15.4 Hz, **H6'**), 4.32 (1H, d, *J* 15.3 Hz, **H6**), 4.15 (1H, q, *J* 7.0 Hz, **H7**), 4.05-3.98 (2H, m, **H33**), 3.74 (3H, s, **H1**), 2.90 (1H, dd, *J* 16.2, 5.5 Hz, **H25'**), 2.80 (1H, dd, *J* 16.0, 6.0 Hz, **H12'**), 2.63 (1H, t, *J* 7.9 Hz, **H35**), 2.57-2.47 (2H, m, **H12+H25**), 2.07 (1H, quint, *J* 7.8 Hz, **H34**), 1.60 (3H, d, *J* 7.1 Hz, **H21**), 1.57 (3H, d, *J* 7.1 Hz, **H8**);

5 Acid-Mediated Conformational Change

As discussed within the paper, acid-mediated conformational change is a useful method of altering foldamer structure and function. (**See paper for references**) Protonation has been used to alter the conformation of pyridine-imidazolidin-2-one foldamers and resulted in a complete switch from a linear, dipole-opposed structure to a curved hydrogen-bonded structure.[17] To further investigate the diketopiperazine-pyridine foldamer shape, an acid-switching experiment was conducted (**Scheme 1**).

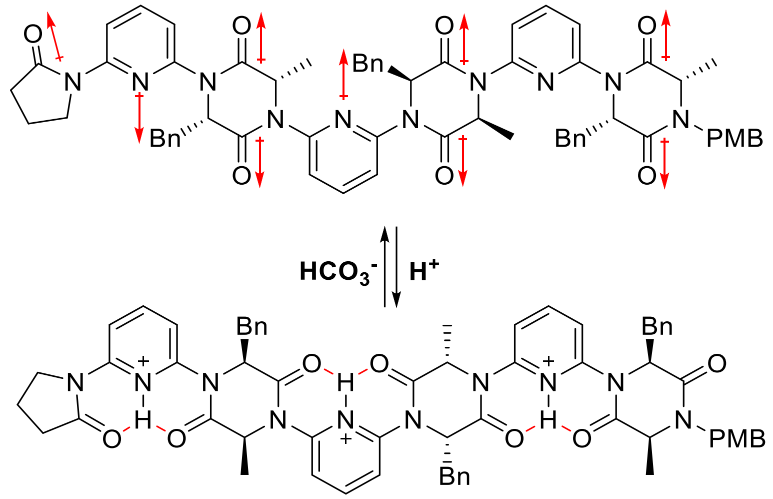

Scheme 1: Proposed reversible acid-mediated conformational change of a diketopiperazine-pyridine foldamer from a dipole-opposed to hydrogen-bonded conformation.

5.1 Dimer 6 Acid-Switching Experiment

The PMB-protected dimer species **6** was dissolved in $CDCl_3$ (9 mM). Portions of dilute *d*-trifluoroacetic acid (*d*-TFA) (1.26 M), concentrated *d*-TFA and TfOH were added (**Table 2**). After each addition, a ^1H NMR spectrum was obtained. The final 'switched' conformation was then analysed by Rotating Frame Overhauser Effect Spectroscopy (ROESY).

Table 2: Dimer **6** acid titration with dilute (1.26 M) *d*-TFA, concentrated *d*-TFA and TfOH.

Entry	Dilute *d*-TFA (µL)	Concentrated *d*-TFA (µL)	TfOH (µL)	Cumulative Equivalent	
				TFA	TfOH
1	0	-	-	0	-
2	5	-	-	1	-
3	5	-	-	2	-
4	10	-	-	4	-
5	20	-	-	8	-
6	40	-	-	16	-
7	-	5	-	27	-
8	-	5	-	37	-
9	-	10	-	58	-
10	-	20	-	99	-
11	-	40	-	181	-
12	-	80	-	345	-
13	-	80	-	509	-
14	-	-	0.6	509	1
15	-	-	0.6	509	2
16	-	-	1.1	509	4
17	-	-	2.2	509	8

After the addition of 509 equivalents of *d*-TFA (**Table 2, Entry 13**), the peaks of the terminal monomer unit, in particular the pyridine peaks, had shifted significantly by ^{1}H NMR. Analysis of the nOe correlations confirmed that the terminal pyridine had been protonated and undergone the expected conformational change from the dipole-opposed to the hydrogen-bonded structure. A stronger acid, TfOH, was added to induce conformational change of the second monomer unit to reach the fully "switched" conformation (**Table 2Table , Entry 17**) & (**Scheme 2**). The sequential switching of the monomer units was likely due to increased allylic strain experienced by the second monomer unit during this transformation.

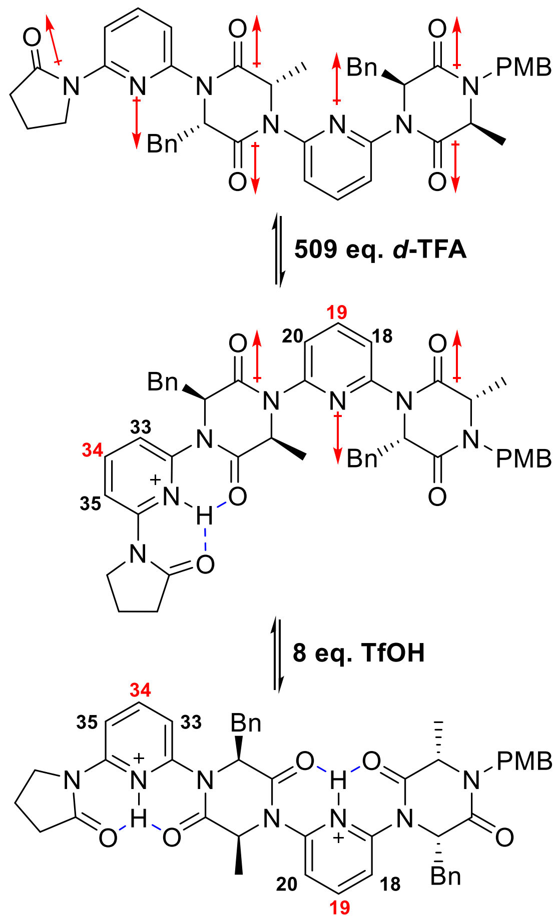

Scheme 2: Three-state switch of the DKP-pyridine dimer **6** from the dipole-opposed to hydrogen-bonded conformation through the addition of *d*-TFA and TfOH.

As acid was added and conformational change occurred, both pyridine *para* hydrogen peaks (**H19** and **H34**) were observed to shift significantly downfield by 0.5-0.6 ppm (**Figure S44**). In contrast, the pyridine *meta* hydrogen peaks shifted upfield upon protonation. The terminal pyridine's *meta* hydrogen (**H35**) moved significantly upfield by approximately 1.2 ppm whereas the *meta* hydrogen (**H33**) shifted by only 0.35 ppm. The *meta* hydrogen peaks of the second pyridine unit (**H18** and **H20**) shifted slightly upfield by 0.1 ppm.

The shifting of the *para* hydrogen peaks downfield is an expected response to protonation as the pyridine is now more electron deficient. The upfield shifting of the *meta* hydrogen peaks did not follow this expected trend. This was also observed during the acid-mediated conformational change of imidazolidinone-pyridine foldamers by Hamilton and co-workers.[17] It was determined that the upfield shifting was an indicator of conformational switching, as these hydrogen atoms were now removed from the deshielding environment of the neighbouring carbonyl groups.

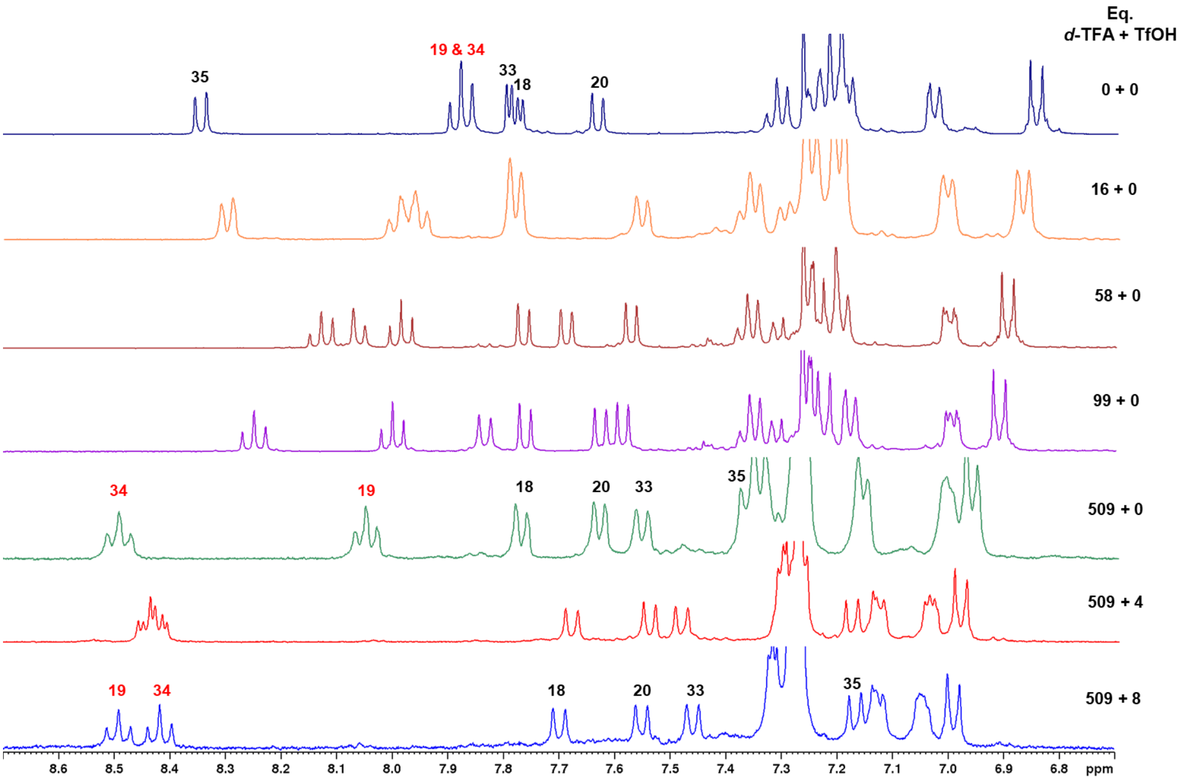

Figure S44: Overlaid ^{1}H NMR spectra showing the shifting of dimer **6** pyridine peaks as increasing equivalents of acid (*d*-TFA and TfOH) were added.

5.1.1 Neutralisation/Reversal of the Conformationally Switched Dimer 6

Following the examination of the influence of an acidic environment on the conformation of dimer **6**, it was then examined if could be returned to its initial neutral conformation via neutralisation of the acidic NMR solution. To do so, protonated dimer **6** in $CDCl_3$ was diluted with $CHCl_3$, and washed three times with saturated aqueous sodium bicarbonate. The organic layer was then dried over $MgSO_4$, filtered through celite, and concentrated in-vacuo.

Looking at the NMR spectrum of the recovered and neutralised dimer **6** from the acid-switching experiment, all peaks had returned to their initial positions before protonation, with *meta*-pyridine peaks (**H18/20** & **H33/35**) shifting back downfield and the *para*-pyridine (**H19 & H34**) upfield (**Figure S44**), thereby demonstrating that the dimer's "conformational-switching" was reversible. However, although the conformation did return to its initial state, a minor change in the structure was observed by ^{1}H NMR, (**Figures S44-S45**) as it appeared that the hydrogens *ortho* to the methoxy group of the PMB moiety had become deuteriated in the process (**Scheme 3, A**). The methoxy substituent directed the deuteration by electrophilic aromatic substitution to the *ortho* position as outlined in the proposed mechanism in **Scheme 3, B**. This deuteration was not observed immediately upon protonation, but occurred over the course of several hours while the NMR titration studies were conducted.

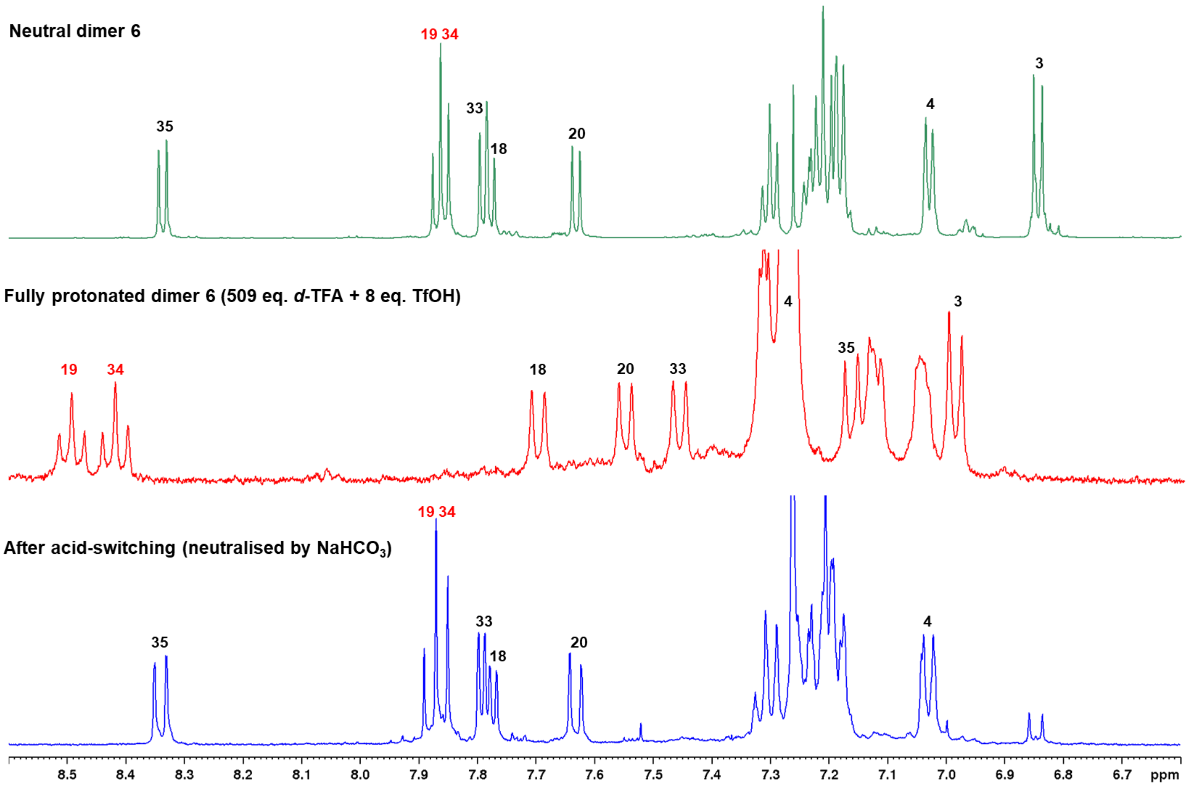

Figure S45: Overlaid ^{1}H NMR spectra showing the shifting of dimer **6** pyridine peaks from neutral, to fully protonated, and after neutralisation with $NaHCO_3$

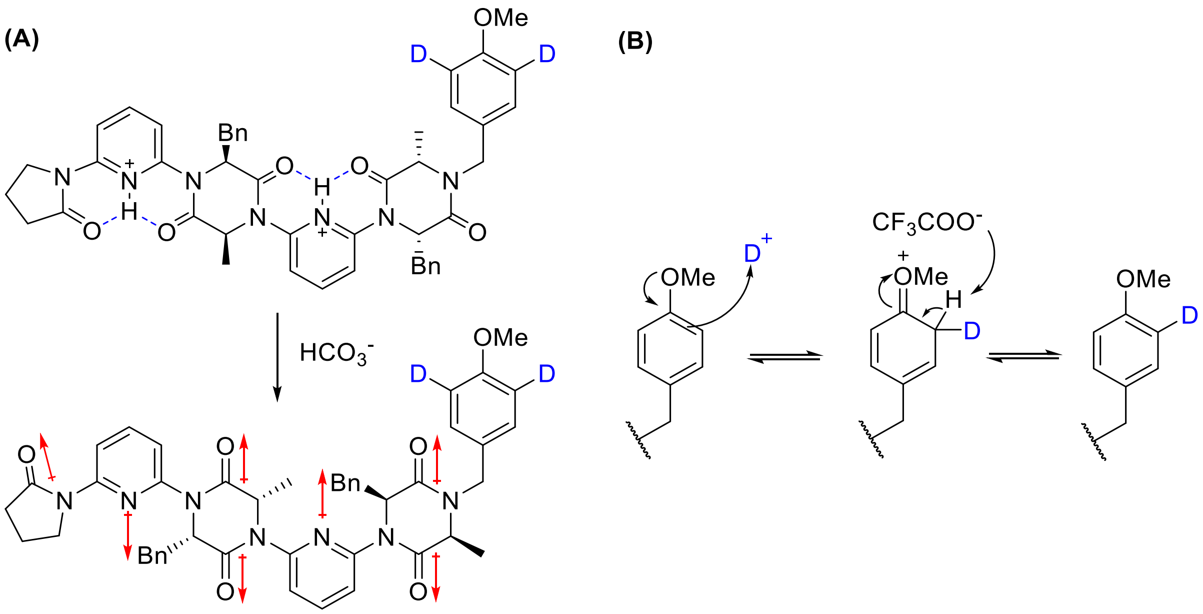

Scheme 3: (A) Treatment of the deuterated dimer in $CDCl_3$ with saturated, aqueous $NaHCO_3$ resulted in conversion back to the dipole-opposed conformation with deuteration of the PMB group. **(B)** Proposed mechanism for the deuteration of the PMB group at the position *ortho* to the methoxy substituent.

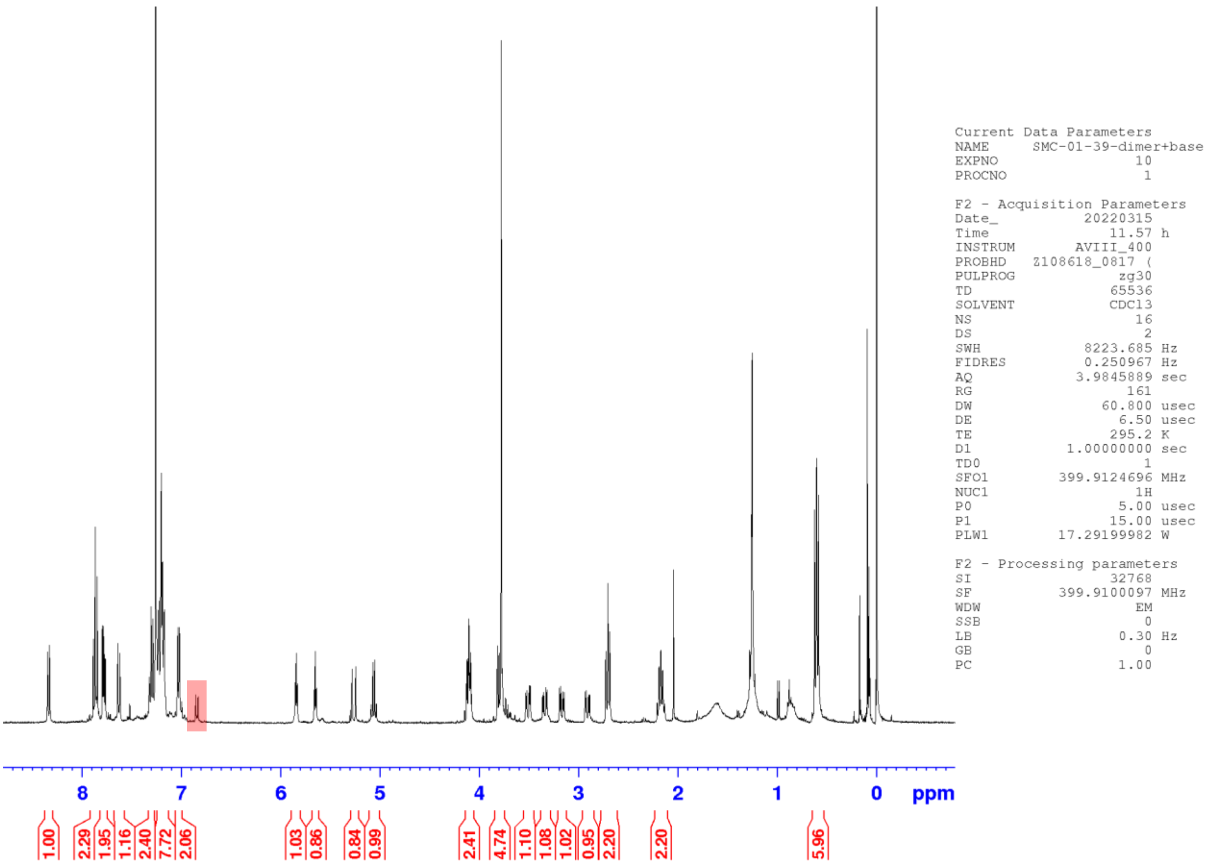

Figure S46: ^{1}H NMR of dimer **6** after $NaHCO_3$ washes (400 MHz, $CDCl_3$). Highlighted in red is the residual protio signal due to the PMB *ortho* hydrogens.

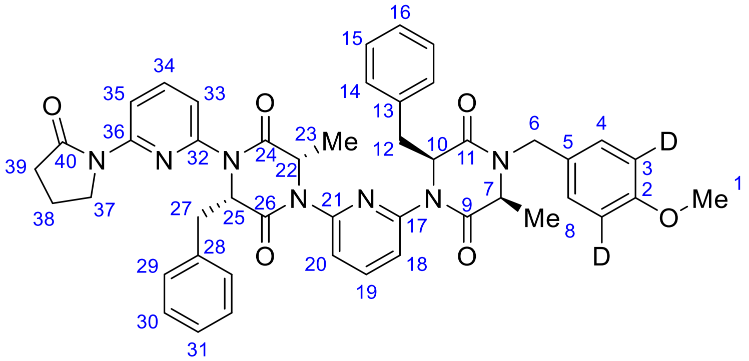

δ$_H$ (400 MHz, $CDCl_3$): 8.34 (1H, d, *J* 8.0 Hz, **H35**), 7.89-7.84 (2H, m, **H19**, **H34**), 7.79 (1H, d, *J* 4.3 Hz, **H33**), 7.77 (1H, d, *J* 4.5 Hz, **H18**), 7.63 (1H, d, *J* 7.4 Hz, **H20**), 7.33-7.15 (10H, m, **H4**, **H15-16**, **H29-31**), 7.03 (2H, d, *J* 6.6 Hz, **H14**), 5.84 (1H, t, *J* 4.5 Hz, **H25**), 5.65 (1H, t, *J* 4.5 Hz, **H10**), 5.26 (1H, d, *J* 14.6 Hz, **H6'**), 5.06 (1H, q, *J* 7.1 Hz, **H22**), 4.15-4.08 (2H, m, **H37**), 3.84-3.79 (2H, m, **H6**, **H7**), 3.78 (3H, s, **H1**), 3.51 (1H, dd, *J* 14.0, 4.1 Hz, **H27'**), 3.34 (1H, dd, *J* 14.0, 4.2 Hz, **H12'**), 3.17 (1H, dd, *J* 13.9, 5.0 Hz, **H27**), 2.91 (1H, dd, *J* 14.0, 4.7 Hz, **H12**), 2.71 (2H, t, *J* 8.0 Hz, **H39**), 2.20-2.13 (2H, m, **H38**), 0.62 (6H, 2d, **H8**, **H23**).

5.2 Dimer 6 Acid-Switching Conformational Control Spectra

Acid-switched dimer 6, mono-protonated

ROESY, $CDCl_3$, 600 MHz, t_{mix} = 0.2 s

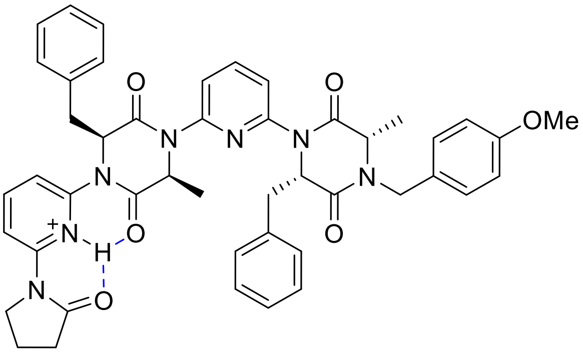

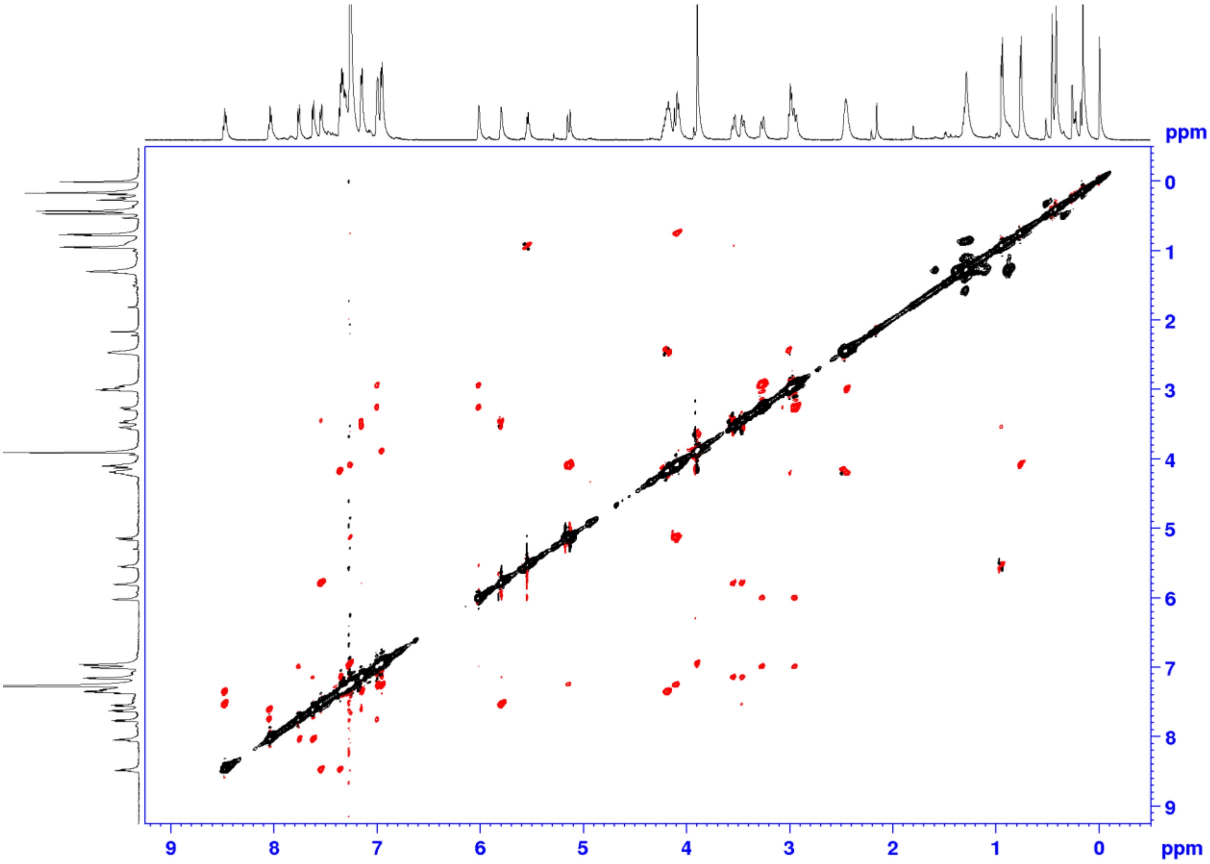

Figure S47: Full ROESY spectrum of mono-protonated acid-switched dimer **6**

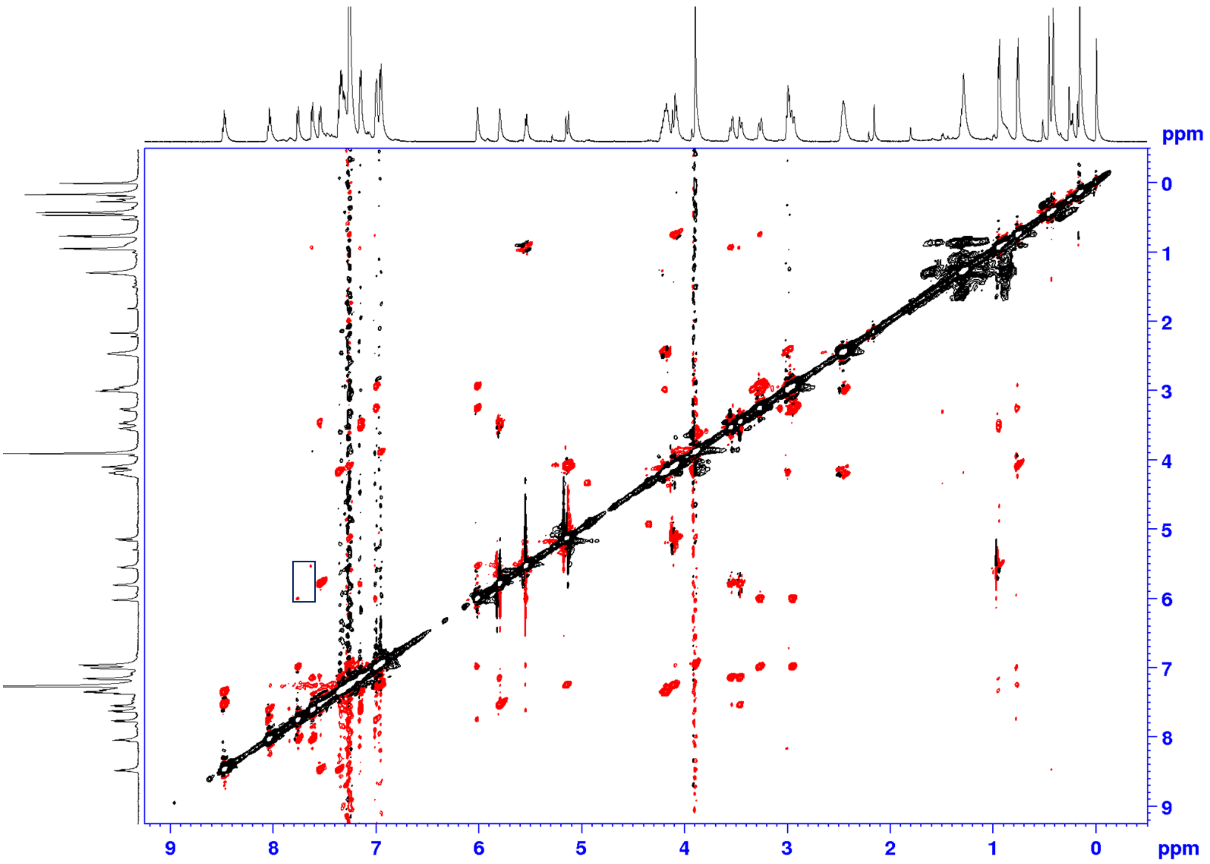

Figure S48: Full ROESY spectrum of mono-protonated acid-switched dimer **6,** with weak interactions

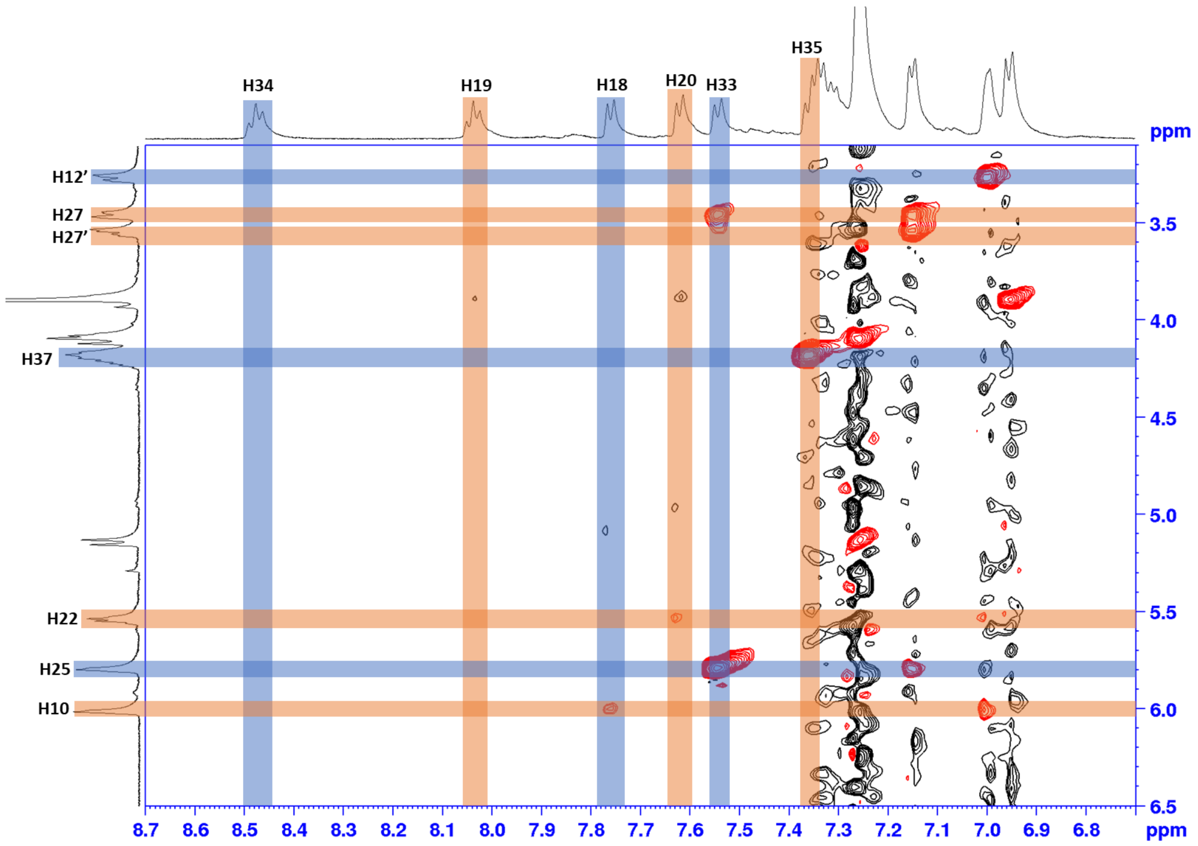

Figure S49: Zoomed area of acid switched mono-protonated dimer **6**, displaying both weak and strong cross-peaks

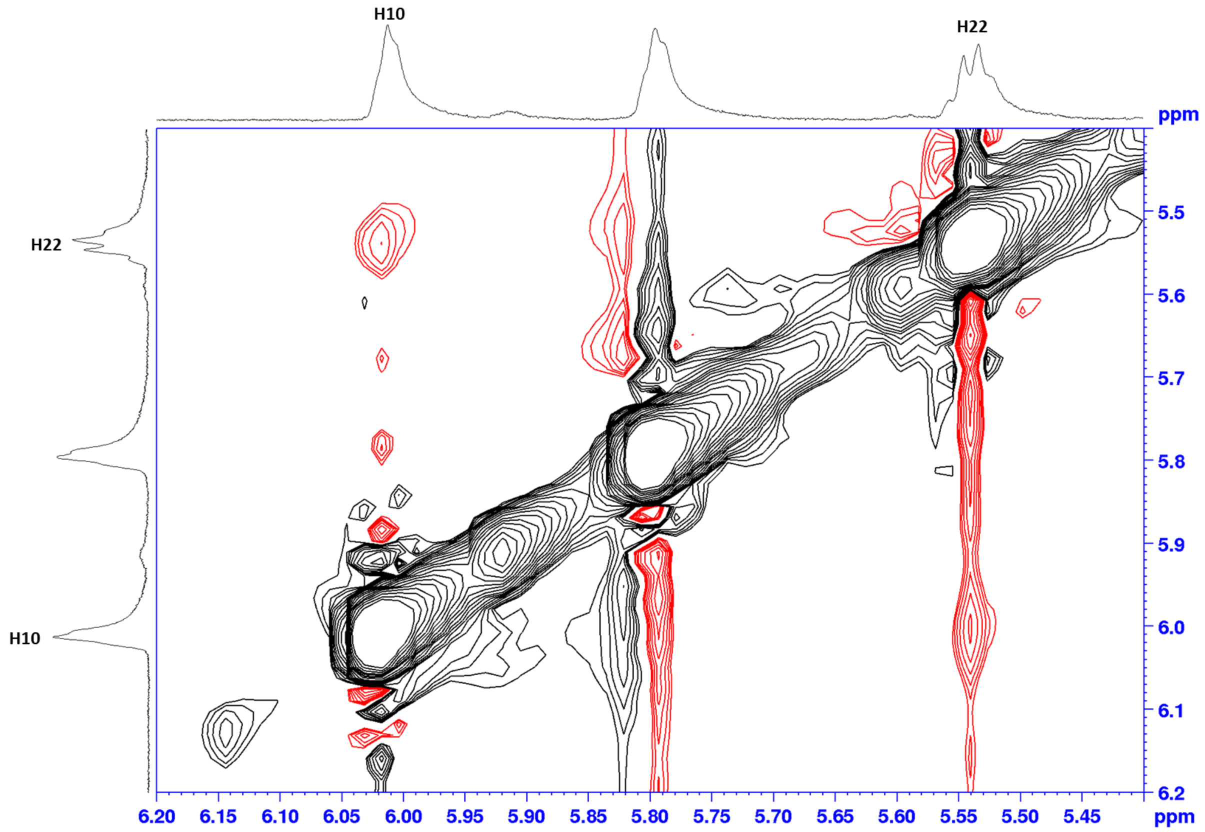

Figure S50: Zoomed area of acid switched mono-protonated dimer **6**, displaying strong inter-residue (**H22**↔**H10**) cross-peaks

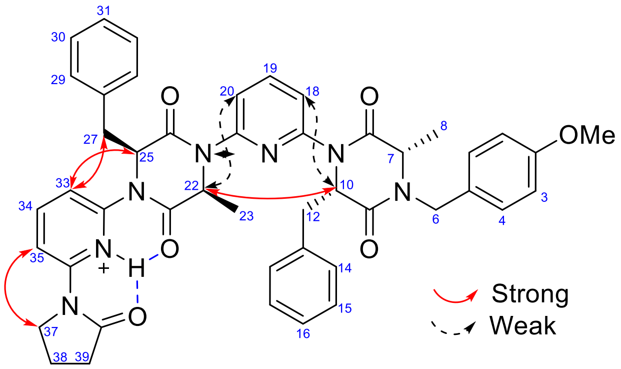

Figure S51: Mono-protonated dimer conformation stabilised through hydrogen-bonding (blue dashed lines). nOe correlations ($CDCl_3$, 600 MHz) from the ROESY spectrum confirm the predicted conformation. Solid red arrows are strong cross-peaks; dashed black arrows are weak cross-peaks.

After the addition of 509 equivalents of *d*-TFA (**Table 2, Entry 13**), but prior to the addition of TfOH, a ROESY spectrum was obtained which indicated that only one of the pyridine moieties had undergone protonation. This was evident by the appearance of strong interactions between the terminal pyridine *meta* hydrogens and pyrrolidinone/diketopiperazine groups (**H37↔H35**, **H33↔H25**, **H33↔H27**) whilst the nOe interactions between (**H22↔H20**, **H18↔H10**, **H18↔H12**) remained identical i.e., very weak, to those observed interaction in its neutral form. (**Figures S47-S50**) This stark contrast in nOe intensities is therefore consistent with a partially switched conformation.

In addition, strong inter-residue nOes between **H22↔H10** could also still be observed in the mono-protonated structure, (**Figure S51**) further providing evidence for a partially switched dimer, as upon full protonation of the backbone, **H22** and **H10** would no longer be close proximity.

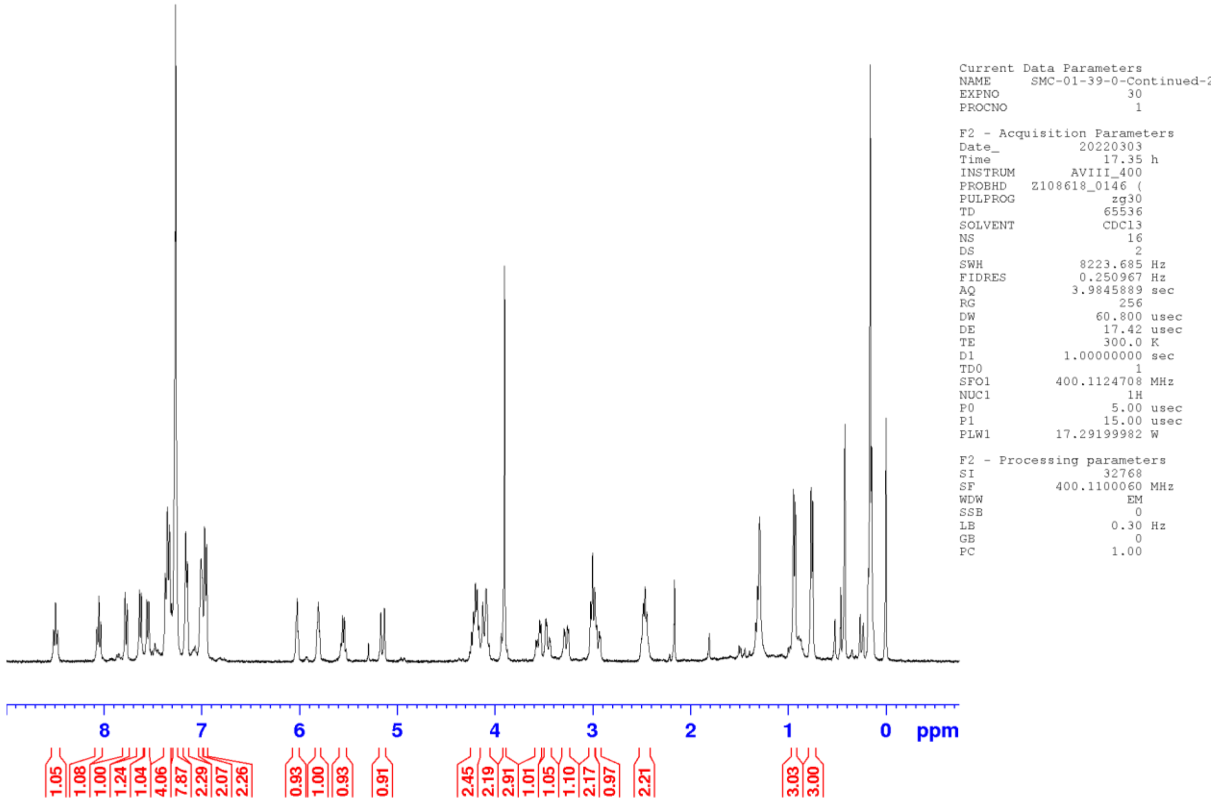

Figure S52: ^{1}H NMR of dimer **6** after addition of *d*-TFA (509 equiv.) (400 MHz, $CDCl_3$).

δ_H (400 MHz, $CDCl_3$): 8.49 (1H, t, *J* 8.5 Hz, **H34**), 8.05 (1H, t, *J* 7.7 Hz, **H19**), 7.77 (1H, d, *J* 8.3 Hz, **H18**), 7.63 (1H, d, *J* 8.2 Hz, **H20**), 7.56 (1H, d, *J* 8.3 Hz, **H33**), 7.39-7.31 (4H, m, **H30-31, H35**), 7.29-7.24 (5H, m, **H4**, **H15-16**)*, 7.16 (2H, d, *J* 6.8 Hz, **H29**), 7.02-6.98 (2H, m, **H14**), 6.96 (2H, d, *J* Hz, **H3**), 6.03 (1H, bt, **H10**), 5.81 (1H, bt, **H25**), 5.55 (1H, q, *J* 7.4 Hz, **H22**), 5.15 (1H, d, *J* 14.8 Hz, **H6'**), 4.25-4.15 (2H, m, **H37**), 4.14-4.05 (2H, m, **H6+H7**), 3.90 (3H, s, **H1**),3.56 (1H, dd, *J* 14.5, 5.8 Hz, **H27'**), 3.46 (1H, dd, *J* 14.4, 4.2 Hz, **H27**), 3.27 (1H, dd, *J* 14.5, 4.4 Hz, **H12'**), 3.00 (2H, t, *J* 7.7 Hz, **H39**), 2.97-2.92 (1H, m, **H12**), 2.45 (2H, quint, *J* 7.7 Hz, **H38**), 0.93 (3H, d, *J* 7.2 Hz, **H23**), 0.76 (3H, d, *J* 7.2 Hz, **H8**).

* Slight over-integration of aromatic region due to co-incidence with $CHCl_3$ peak

Acid-switched dimer 6, di-protonated

ROESY, CDCl3, 600 MHz, t_{mix} = 0.2 s

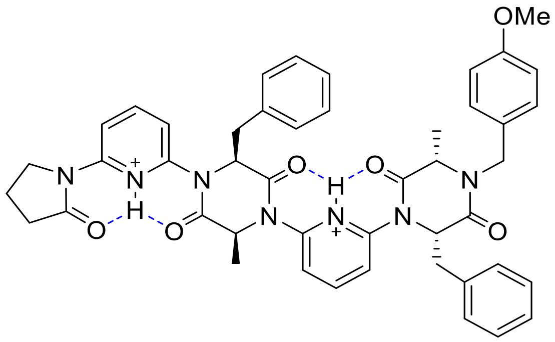

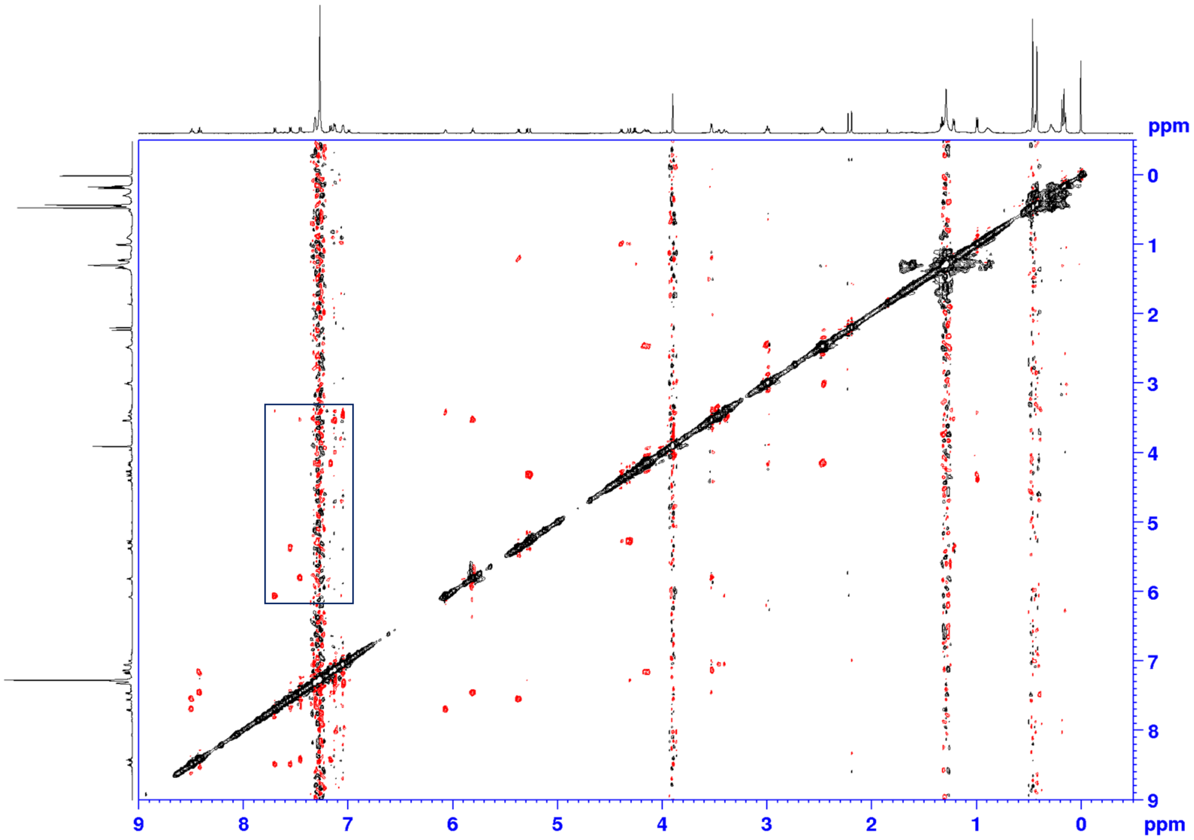

Figure S53: Full ROESY spectrum of fully acid switched dimer **6**

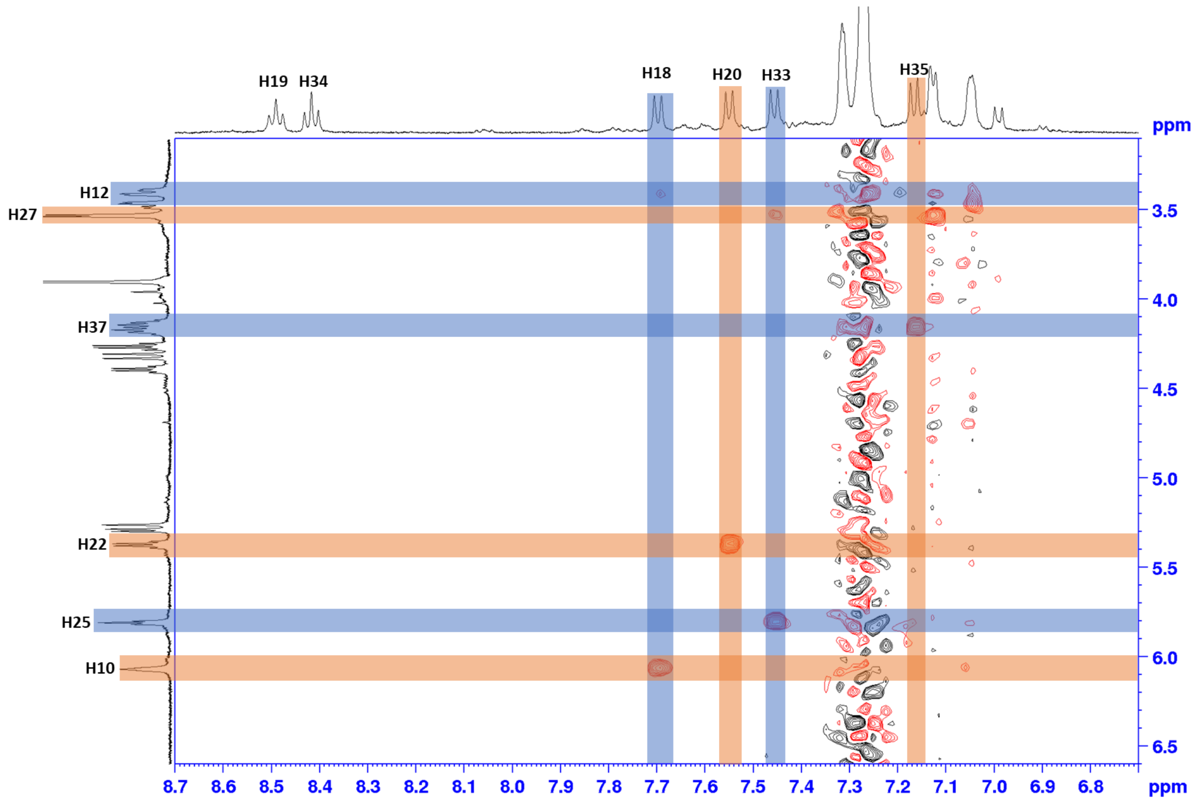

Figure S54: Zoomed area of acid switched di-protonated dimer **6**, displaying both weak and strong cross-peaks

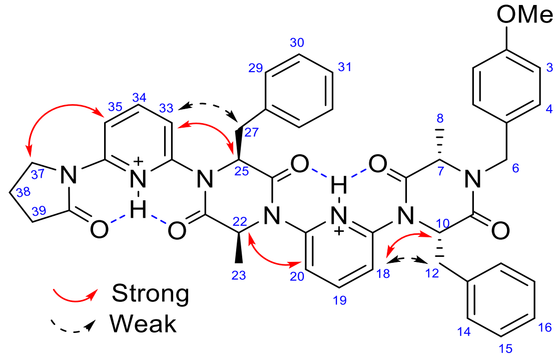

Figure S55: Protonated dimer **6** conformation stabilised through hydrogen-bonding (blue dashed lines). nOe correlations ($CDCl_3$, 600 MHz) from the ROESY spectrum confirm the predicted conformation. Solid red arrows are strong cross-peaks; dashed black arrows are weak cross-peaks.

A ROESY spectrum was obtained after the addition of both *d*-TFA and TfOH (**Table 2, Entry 17**) and the nOe correlations agreed with the protonated, hydrogen-bonded conformation (**Figures S53-S54**). Strong interactions were observed between the terminal pyridine *meta* hydrogens and pyrrolidinone/diketopiperazine groups (**H37↔H35**, **H33↔H25**, **H33↔H27**). This contrasted with the dipole-opposed ROESY spectrum which had weak or completely absent pyridine interactions. (**See Figures S6-S8**) Additionally, interactions were observed between the second pyridine's *meta* hydrogens and the adjacent diketopiperazine units (**H22↔H20**, **H18↔H10**, **H18↔H12**). (**Figure S54**) These correlations confirmed that

both pyridines had been protonated and had “switched” from the dipole-opposed conformation to a hydrogen-bond stabilised structure.

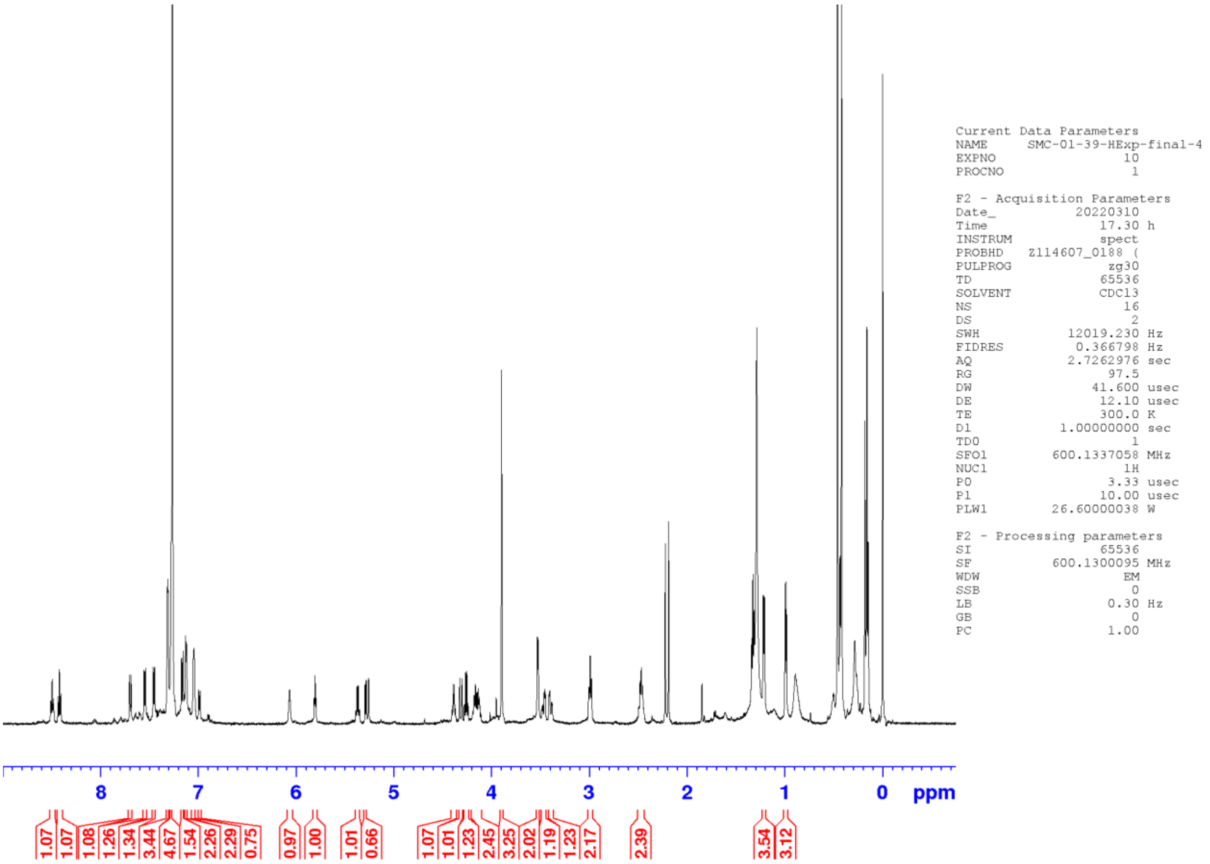

Figure S56: ^{1}H NMR of dimer **6** after addition of *d*-TFA (509 equiv.) + TfOH (8 equiv.) (600 MHz, $CDCl_3$).

δ_H (600 MHz, $CDCl_3$):[*] 8.48 (1H, t, *J* 8.7 Hz, **H19**), 8.41 (1H, t, *J* 8.5 Hz, **H34**), 7.69 (1H, d, *J* 8.7 Hz, **H18**), 7.54 (1H, d, *J* 8.6 Hz, **H20**), 7.45 (1H, d, *J* 8.7 Hz, **H33**), 7.33-7.30 (3H, m, **H15-16**), 7.26 (5H, m, **H4**, **H30-31**), 7.16 (1H, d, *J* 8.6 Hz, **H35**), 7.12 (2H, d, *J* 6.8 Hz, **H29**), 7.06-7.00 (2H, m, **H14**), 6.98 (1H, d, *J* 9.3 Hz, **H3**), 6.06 (1H, bs, **H10**), 5.80 (1H, t, *J* 5.6 Hz, **H25**), 5.36 (1H, q, *J* 7.4 Hz, **H22**), 5.26 (1H, d, *J* 14.7 Hz, **H6’**), 4.38 (1H, q, *J* 7.2 Hz, **H7**), 4.31 (1H, d, *J* 14.5 Hz, **H6**), 4.20-4.09 (2H, m, **H37**), 3.89 (3H, s, **H1**), 3.54-3.50 (2H, d, *J* 5.7 Hz, **H27**), 3.46 (1H, dd, *J* 15.0, 5.4 Hz, **H12’**), 3.39 (1H, dd, *J* 14.9, 4.0 Hz, **H12**), 2.98 (2H, t, *J* 8.0 Hz, **H39**), 2.47 (2H, quint, *J* 7.7 Hz, **H38**), 1.21 (3H, d, *J* 7.2 Hz, **H23**), 0.98 (3H, d, *J* 7.1 Hz, **H8**).

---

[*] Due to partial in-situ deuteration of PMB, the integration of **H3** is below the expected value of 2 protons.

5.3 Attempted Acid-Mediated Conformational Switching of Dimer 6 in *d6*-DMSO

As retention of the dipole-controlled neutral conformation had been demonstrated in *d6*-DMSO, (**See Section 3.3**) we examined if the formation of the acid-switched/protonated conformation was also retained in a *d6*-DMSO. Dimer **6** was dissolved in *d6*-DMSO (9 mM), and the same equivalents of acid (*d*-TFA and TfOH) used in the sequential switching were added directly to the sample.

Starting with 509 equiv. *d*-TFA, which in $CDCl_3$ had shown to result in mono-protonation of the pyrrolidine-pyridine-DKP unit, 509 equiv. *d*-TFA was directly added to dimer **6**. However, after the addition *d*-TFA, it was apparent that no protonation had taken place, with none of the characteristic downfield shift of **H34** or upfield shift of **H35** being observed. The only noticeable difference was that all peaks had shifted slightly upfield (~0.02-0.10 ppm) (**Figure S57**). As increasing amounts of *d*-TFA interfered with the shimming of trimer **8** above 786 equiv. of *d*-TFA, it was then opted to start adding TfOH. However, after adding up to 8 equiv. of TfOH, no changes in the pyridine peak positions were noted. As it was thought that may have been due to the reduced in acidity of TfOH in *d6*-DMSO, a further 56 equiv. of TfOH (64 equiv. in total) was directly added to see if full protonation of dimer **6** could be achieved, however, there was still no difference in the spectra between 8 equiv. and 64 equiv. bar a slight shifting in peaks upfield (0.10 ppm), indicating no protonation had taken place. (**Figure S57**)

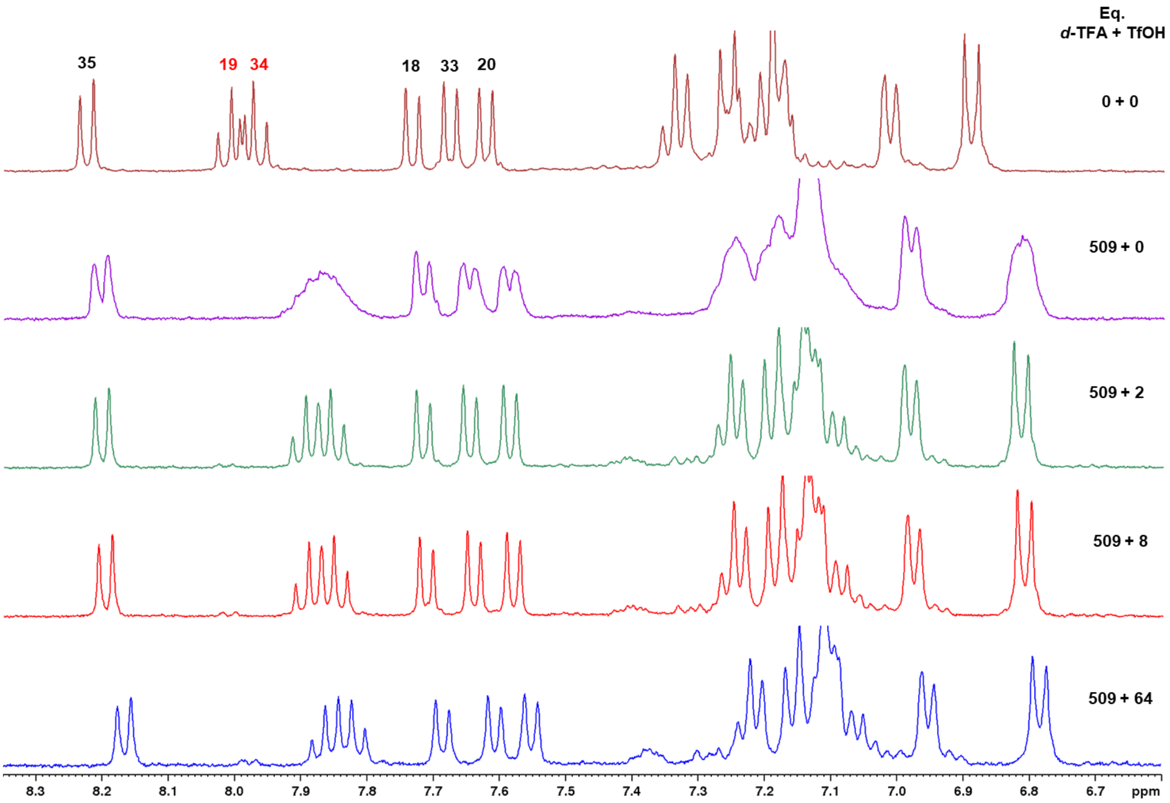

Figure S57: Overlaid ^{1}H NMR spectra in *d6*-DMSO, showing the absence of significant shifting of dimer **6** pyridine peaks upon increasing equivalents of acid (*d*-TFA and TfOH).

Since a substantial excess of TFA (509 equiv.) and TfOH (64 equiv.), was unable to result in any conformational switching, it was demonstrated that acid-mediated conformational was not achievable in *d6*-DMSO. A significant reason for this may be that DMSO acts as a buffer for the strong acids: the pK_{BH+} value for DMSO has been reported as ~0-1.[18] The inability to form the protonated conformation may also in part be due to *d6*-DMSO's hydrogen bond acceptor ability, potentially disrupting the six-member hydrogen bond network formed upon protonation of the pyridine. This negative impact of DMSO on the stability of hydrogen-bond control foldamers is well noted, with examples being seen in Jiang *et al.*'s aromatic triazole foldamers,[19] comprising of repeating unit of isobutyl 4-chloro/flurobenzoate, forming a central intramolecular hydrogen-bond network between triazole protons and halogen atoms, (**Figure 58**) resulting in the foldamer occupying a helical conformation. This helical conformation however was disrupted but upon dissolving in *d6*-DMSO, due to DMSO completive hydrogen-bond acceptor ability.

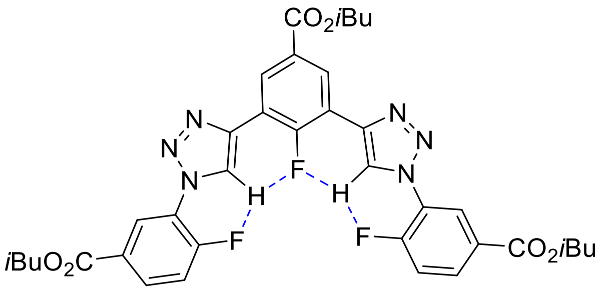

Figure S58: Jiang *et al.*'s aromatic triazole foldamer, with hydrogen bond network highlighted in blue

Huc *et al.*[20] noted that the tertiary structure of their helix-turn-helix abiotic foldamers comprising of 8-amino-2-quinolinecarboxylic acid and 6-aminomethyl-4-hydroxy-2-pyridinecarboxylic acid units linked via a terephalohydraide moiety, (**Figure S59**) was disrupted when studied in *d6*-DMSO, with its presence interfering with the intermolecular-hydrogen bonding between the adjacent nitrogen-contain arene units, which gave rise to the *PP*-folded structure in $CDCl_3$, but *PM*-unfolded structure in *d6*-DMSO, owing to the individual helix unit's intramolecular hydrogen bonding system remaining unaffected in *d6*-DMSO. The sensitively was then further demonstrated via titration *d6*-DMSO into $CDCl_3$, required between 18-23% volume to result in disruption of the inter-helix hydrogen bond system.

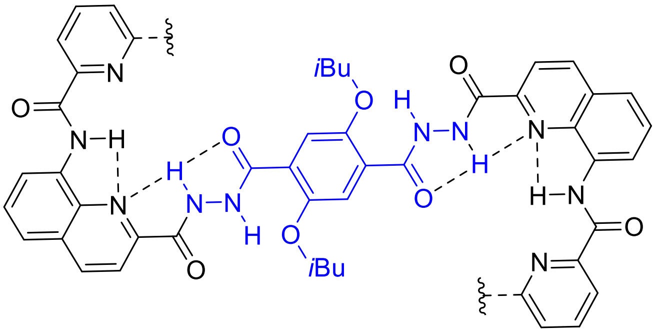

Figure S59: Huc *et al.*'s helix-turn-helix foldamer's central unit

Lastly, although not *d6*-DMSO, Cuccia *et al.*'s[21] helical foldamer comprising of alternating aromatic nitrogen-containing heterocycles (pyridazine, pyrimidine or pyrazine) and methyl-substituted aromatic carbocycles linked via a urea, experience a loss of conformation when dissolved in *d4*-MeOD, (**Figure S60**) interfering with the six-membered intra-molecular hydrogen system between the urea and nitrogen containing arene, thereby disfavouring the *trans*-oid orientation that enabled the helical conformation.

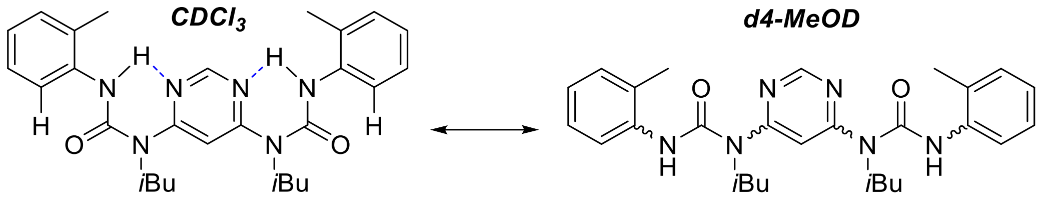

Figure S60: Cuccia *et al.*'s heterocyclic-urea foldamer undergoing a loss of conformation from $CDCl_3$ to *d4*-MeOD

5.4 Monomer 4 Acid-Switching Experiment and Conformational Control Spectra

As the dimer had demonstrated partial depending on strength of the acid added, with 509 equiv. TFA on result in mono-protonation of the pyrrolidinone-DKP unit. It was therefore examined if adding 509 equiv. of TFA directly added to monomer **4** in $CDCl_3$ (9 mM) would result in the same switching behaving, providing further evidence that this is the unit the undergoes the first conformational change in the presence of acidic conditions.

After the addition of 509 equivalents of *d*-TFA a NOESY spectrum was then obtained and comparing it with the spectrum of neutral monomer **4** (**See Section 3.2, Figure S4**), there was a very strong increase in the nOes between the *meta*-pyridine peaks and the adjacent pyrrolidinone (**H15↔H13**) and DKP (**H19↔H11 and H21↔H11**), (**Figure S62-63**) indicating the pyridine moieties had undergone protonation and associated conformational change further providing evidence that in the presence of TFA it is the first diketopiperazine moiety that becomes protonated.

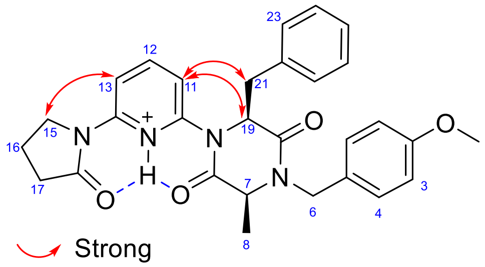

Figure S61: Protonated monomer **4** conformation stabilised through hydrogen-bonding (blue dashed lines). nOe correlations ($CDCl_3$, 400 MHz) from the NOESY spectrum confirm the predicted conformation. Solid red arrows are strong cross-peaks

Acid-switched monomer 4, mono-protonated

NOESY, $CDCl_3$, 400 MHz, t_{mix} = 0.6 s

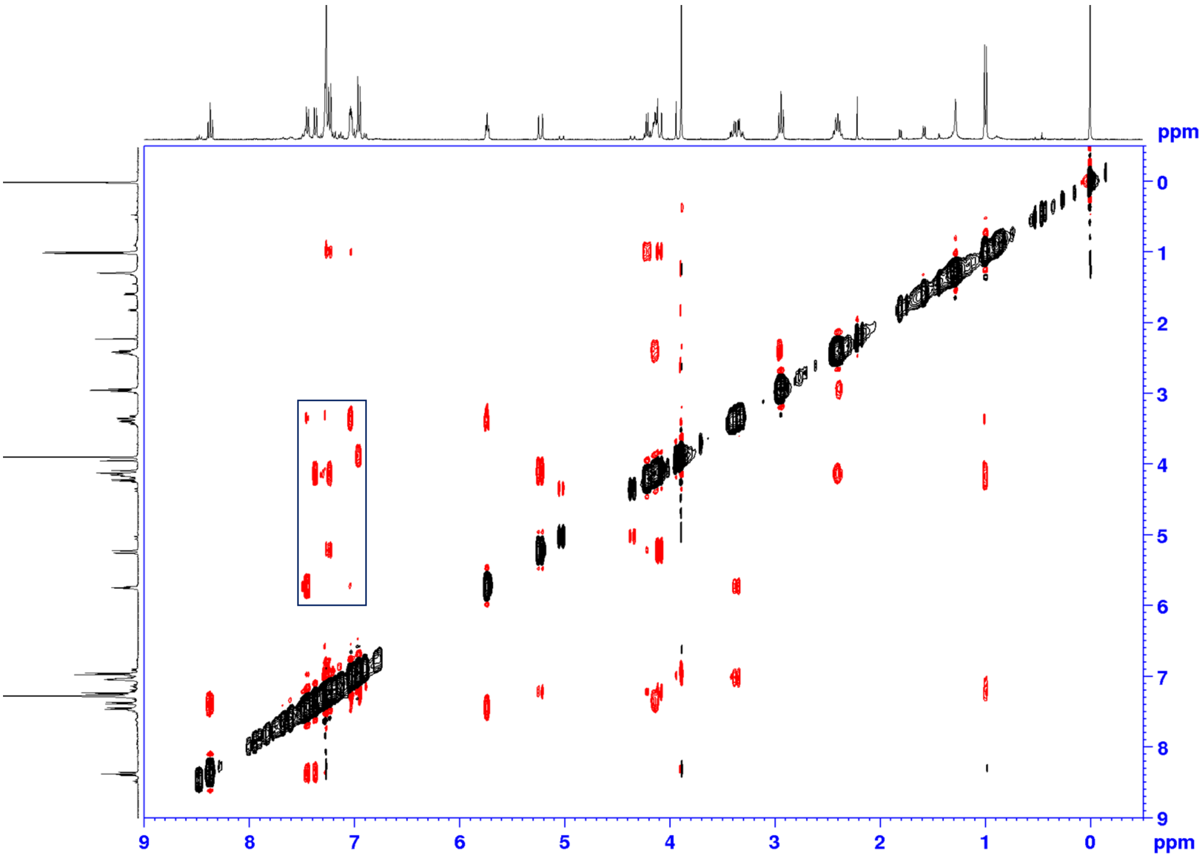

Figure S62: Full NOESY spectrum of protonated acid-switched monomer **6**

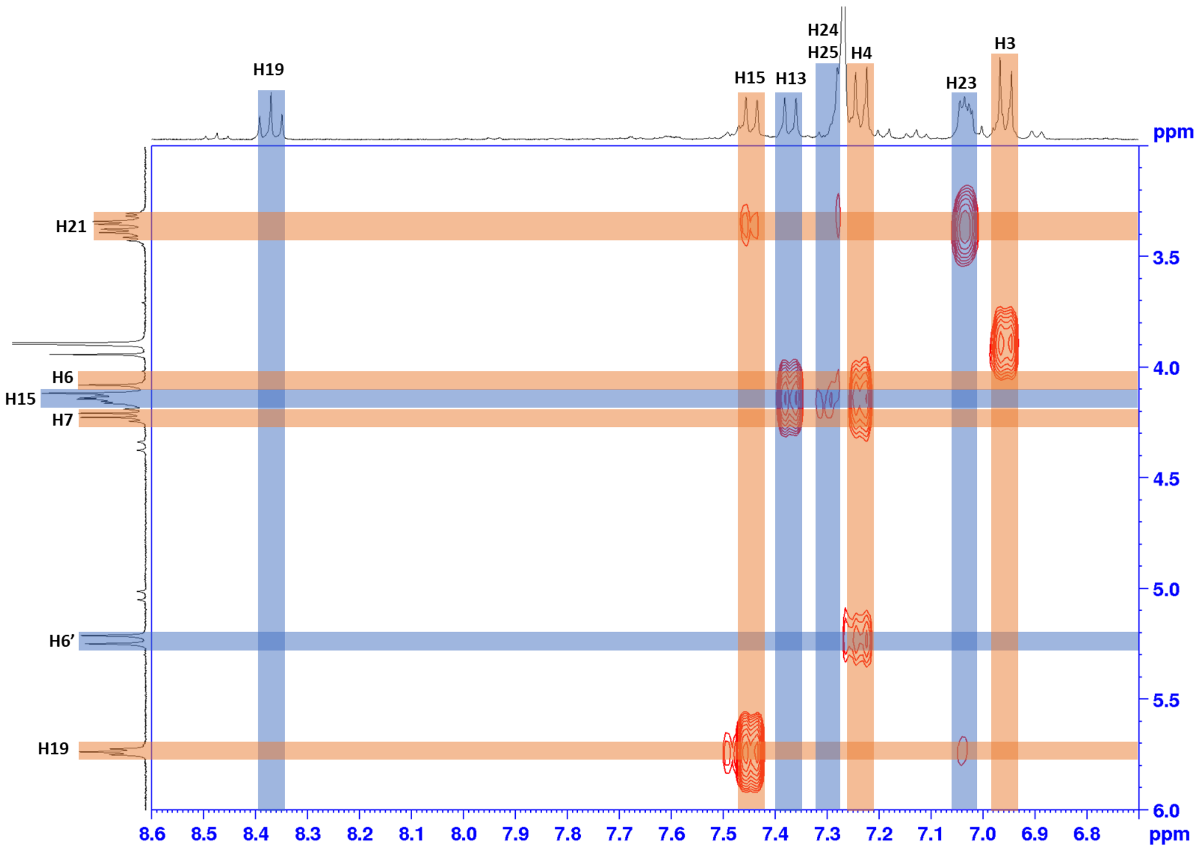

Figure S63: Zoomed area of acid switched protonated monomer **4**

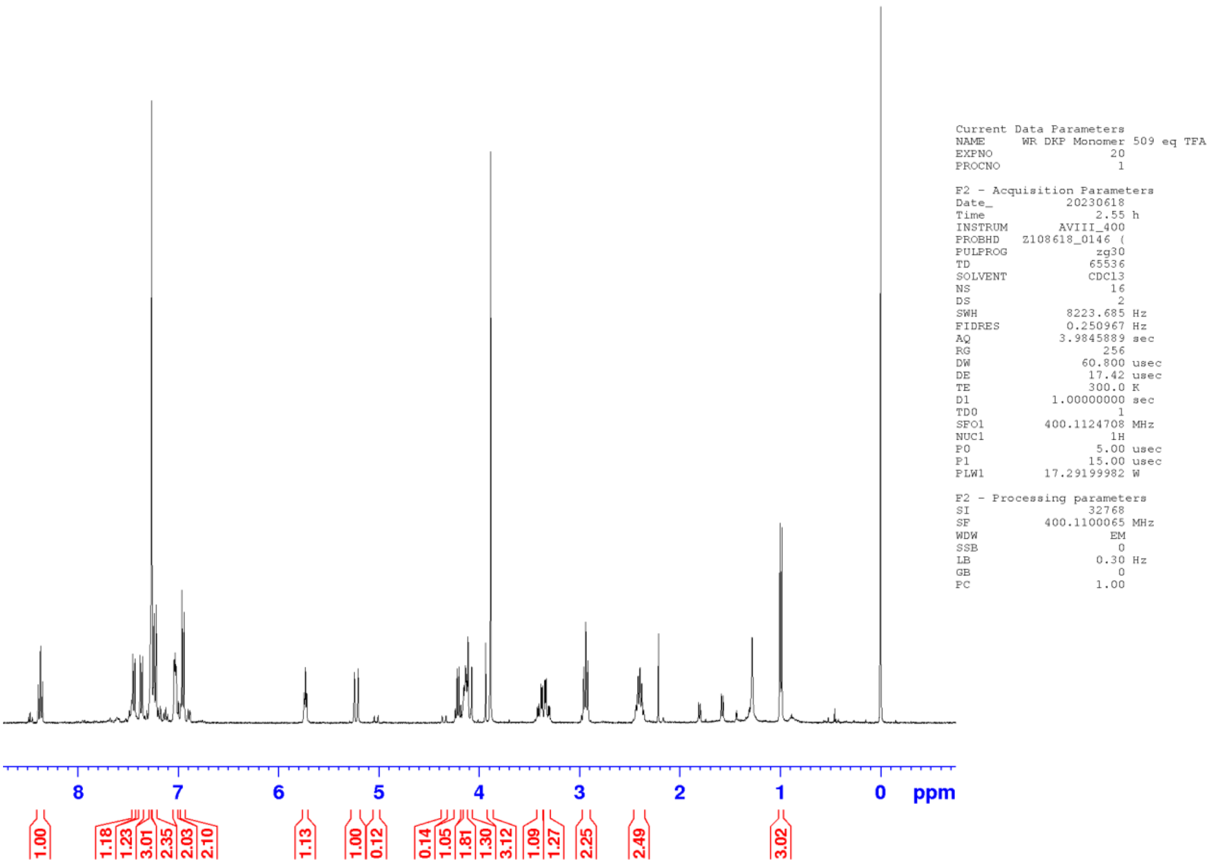

Figure S64: ^{1}H NMR of monomer **4** after addition of *d*-TFA (509 equiv.) (400 MHz, $CDCl_3$).

δ_H (400 MHz, $CDCl_3$): 8.37 (1H, t, *J* 8.6 Hz, **H19**), 7.44 (1H, d, *J* 8.5 Hz, **H15**), 7.37 (1H, d, *J* 8.7 Hz, **H13**), 7.30-7.26 (3H, m, **H24 + H25**), 7.24 (2H, d, *J* 8.8 Hz, **H23**), 7.05-7.02 (2H, m, **H23**), 6.96 (2H, d, *J* 8.8 Hz, **H3**), 5.73 (1H, t, *J* 5.03 Hz, **H19**), 5.23 (1H, d, *J* 14.8 Hz, **H6'**), 4.21 (1H, q, *J* Hz, **H7**), 4.16-4.11 (2H, m, **H15**), 4.09 (1H, d, *J* 14.9 Hz, **H6**), 3.88 (3H, s, **H1**), 3.40 (1H, dd, *J* 14.5, 6.0 Hz, **H21'**), 3.32 (1H, dd, *J* 14.5, 4.8 Hz, **H21**), 2.96 (2H, t, *J* 8.1 Hz, **H17**), 2.40 (2H, quint, *J* 7.8 Hz, **H16**), 1.00 (3H, d, *J* 7.1 Hz, **H8**).

5.5 Dimer 13 Acid-Switching Experiment

To demonstrate that the same stimulus response to the acidic condition was still retained with foldamer bearing different sidechain functionality, the acid titration experiment was also conducted with the PMB-protected dimer species **13**, bearing the carboxylic acid**.**

The dimer was dissolved in $CDCl_3$ (9 mM) and portions concentrated d-TFA and TfOH were added (**Table 3**). After each addition, a ^1H NMR spectrum was obtained and the final 'switched' conformation was analysed by Rotating Frame Overhauser Effect Spectroscopy (ROESY).

Table 3: Dimer **13** acid titration with concentrated *d*-TFA and TfOH.

Entry	Concentrated *d*-TFA (µL)	TfOH (µL)	Cumulative equivalent	
			***d*-TFA**	**TfOH**
1	0	-	0	-
2	22.6	-	50	-
3	22.6	-	100	-
4	67.8	-	250	-
5	113	-	500	-
6	-	0.5	500	1
7	-	0.5	500	2
8	-	1.0	500	4
9	-	2.0	500	8

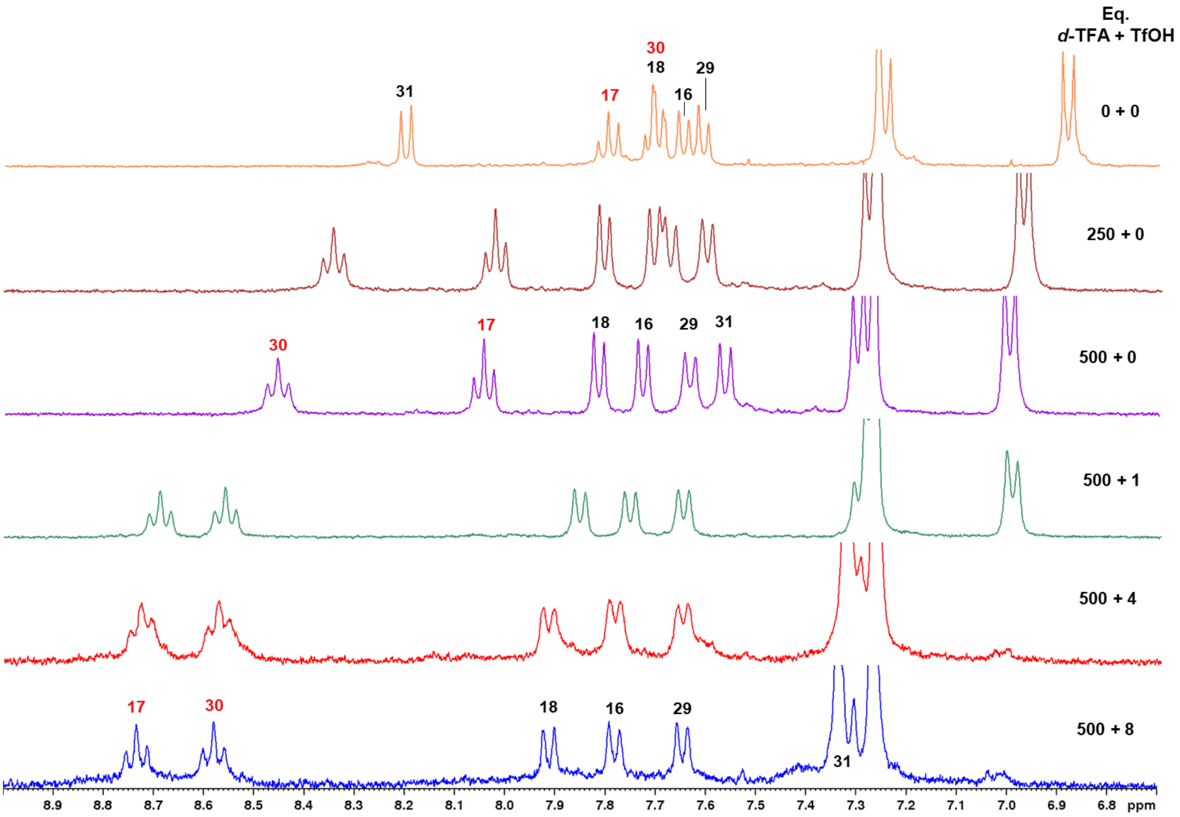

Figure S65: Overlaid ^{1}H NMR spectra showing the shifting of dimer **13** pyridine peaks as increasing equivalents of acid (*d*-TFA and TfOH) were added.

Akin to dimer **6** bearing the benzyl-sidechain, sequential switching of each monomer unit was also observed during the titration of dimer **13**. (**Figure S65**) After 500 equivalents of *d*-TFA (**Table 3**, **Entry 5**), only the terminal pyridine was observed to have "switched" conformation by ^{1}H NMR and ROESY spectroscopy. Full conformational change was achieved after the addition of 8 equivalents of TfOH (**Table 3, Entry 9**). In addition, after 4.0 equiv. of TfOH, the disappearance of **H3** can be seen, suggesting that it has undergone deuteration via the mechanism shown in **Scheme 3, B**.

In trends consistent with those observed during the titration of dimer **6**, upon addition of increasing amounts of acid, significant shifting of the pyridine peaks was observed by ^{1}H NMR (**Figure S65**). The pyridine *para* hydrogen peaks (**H17** and **H30**) shifted downfield by 0.7-0.9 ppm whereas the *meta* hydrogen peaks shifted upfield. The terminal *meta* hydrogen (**H31**) shifted considerably upfield by 0.9 ppm whereas the other *meta* hydrogens (**H16**, **H18**, and **H29**) shifted downfield by 0.10-0.20 ppm, in contrast to the analogue's *meta* hydrogens of dimer **6** which shifted 0.10-0.30 ppm upfield.

5.6 Dimer 13 Acid-Switching Conformational Control Spectra

Acid-switched dimer 13, mono-protonated

ROESY, $CDCl_3$, 400 MHz, t_{mix} = 0.2 s

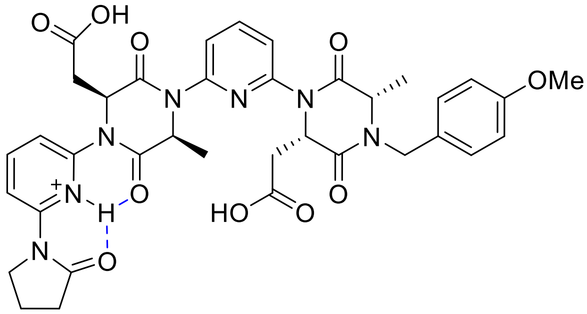

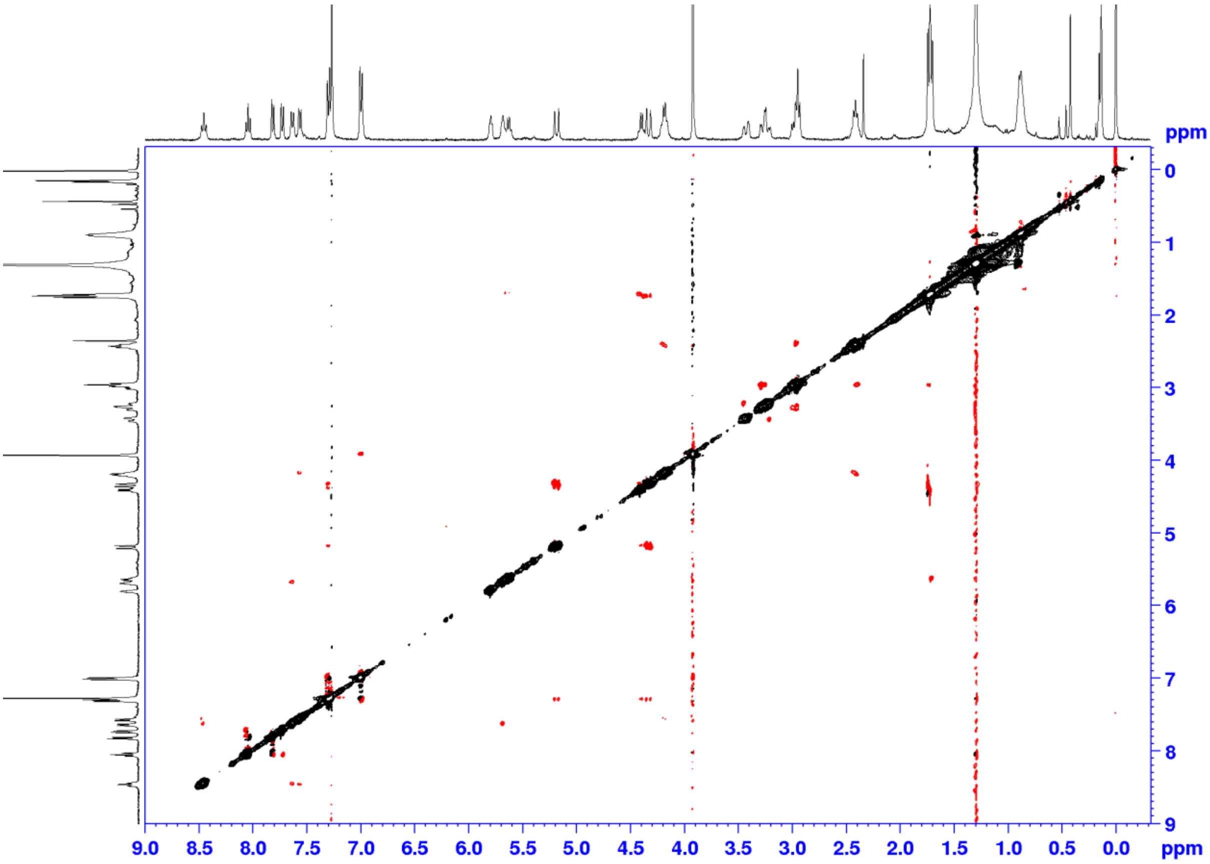

Figure S66: Full ROESY spectrum of mono-protonated acid-switched dimer **13**

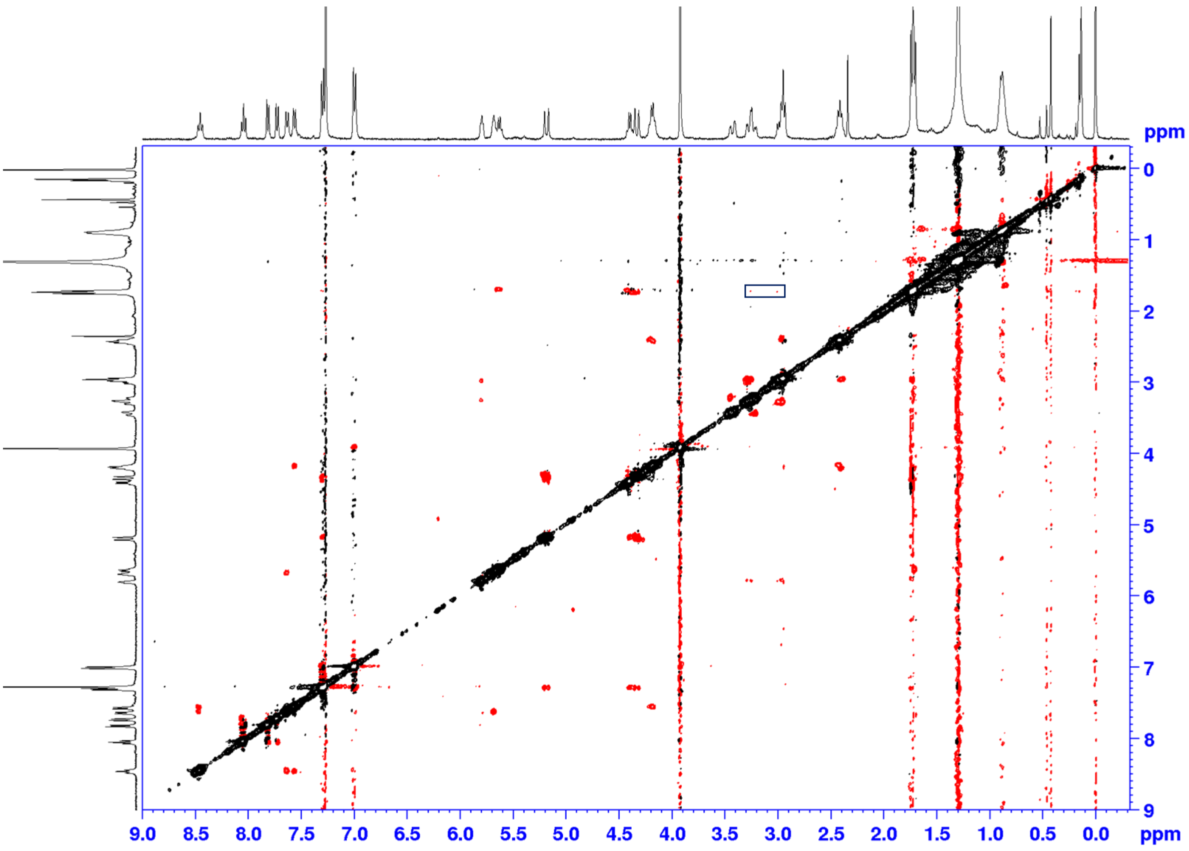

Figure S67: Full ROESY spectrum of mono-protonated acid-switched dimer **13,** with weak inter-residue interactions between **H21↔H12**, highlighted by a navy rectangle.

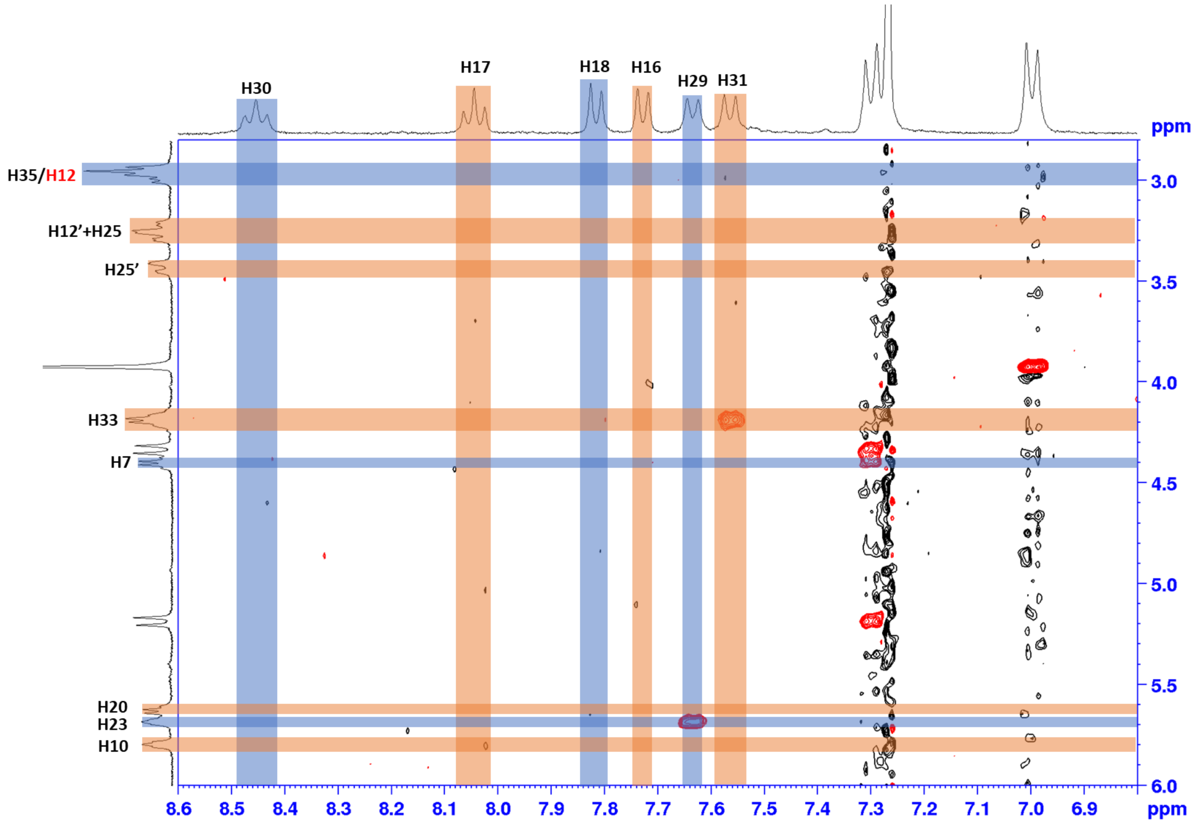

Figure S68: Zoomed area of acid switched mono-protonated dimer **13**

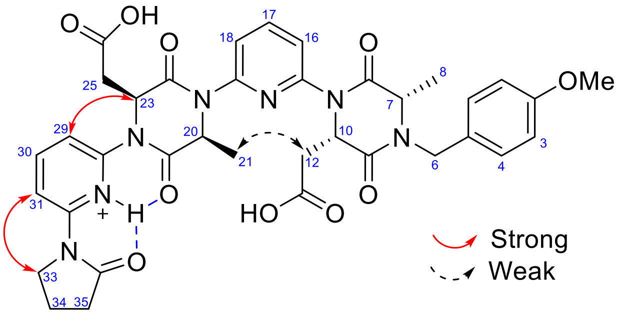

Figure S69: Mono-protonated dimer **13** conformation stabilised through hydrogen-bonding (blue dashed lines). nOe correlations ($CDCl_3$, 400 MHz) from the ROESY spectrum confirm the predicted conformation. Solid red arrows are strong cross-peaks; dashed black arrows are weak cross-peaks.

After the addition of 500 equivalents of *d*-TFA (**Table 3, Entry 5**), but prior to the addition of TfOH, a ROESY spectrum was obtained which indicated that only one of the pyridine moieties had undergone protonation. This was evident by the appearance of strong interactions between the terminal pyridine *meta* hydrogens and pyrrolidinone/diketopiperazine groups (**H33↔H31** and **H29↔H23**), (**Figure S66**) however, unlike dimer **6**, no interactions between the CH_2's of **H25** and the *meta*-pyridine **H29** were visible. This isn't too unusual though as the mono-protonated trimer **8** also lacked these analogous nOe interactions.

An additional interaction that increased in intensitie was the appearance of a weak nOe between **H21↔H12**, (**Figures S67-S68**) which was not present in the neutral form, (**See Section 4.3**) further suggesting a change in relative conformation between the two DKP-pyridine units.

Further evidence for the partial switching could be seem in the absence of nOe interactions between **H20↔H18** or **H16↔H10**, which indicates that unit had not undergone any switching as there was no increase in the nOe intensities, since this lack of nOe interactions was consistent with dimer **13** in its neutral form in chloroform. (**See section 4.3**)

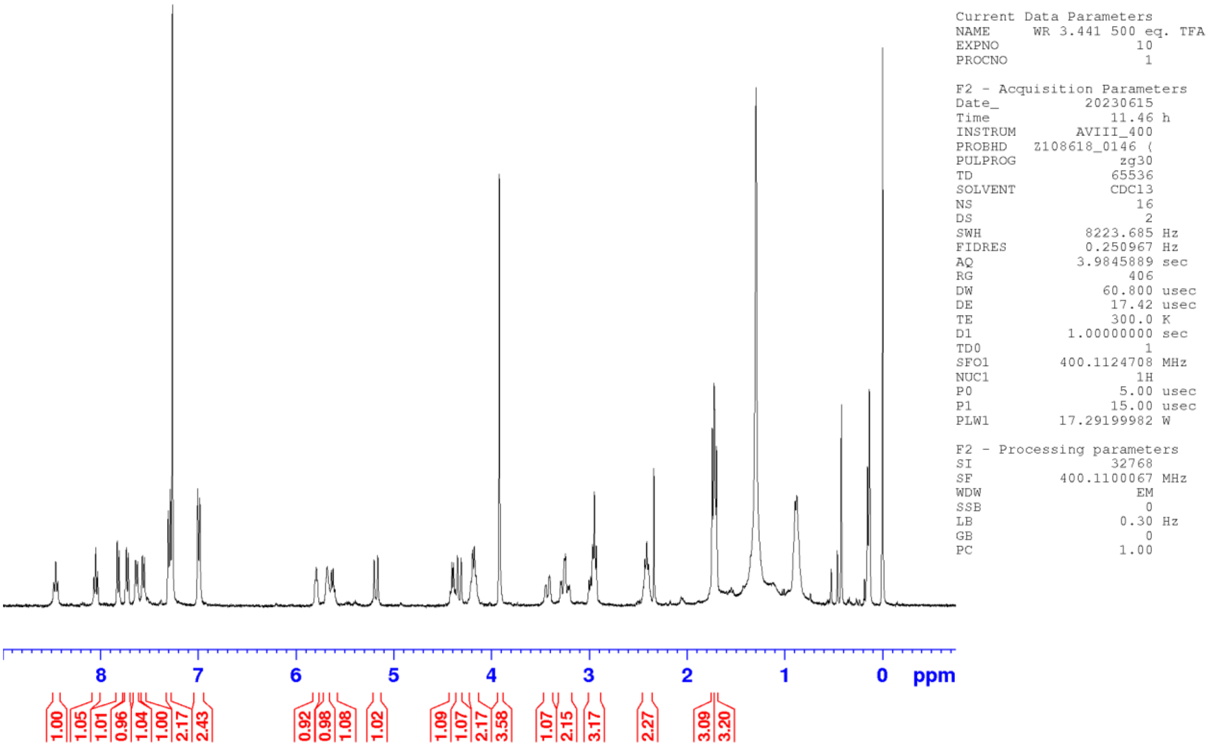

Figure S70: ^{1}H NMR of dimer **13** after addition of *d*-TFA (500 equiv.) (400 MHz, $CDCl_3$).

δ_H (400 MHz, $CDCl_3$): 8.45 (1H, t, *J* 8.8 Hz, **H30**), 8.04 (1H, t, *J* 8.1 Hz, **H17**), 7.82 (1H, d, *J* 8.2 Hz, **H18**), 7.73 (1H, d, *J* 8.2 Hz, **H16**), 7.64 (1H, d, *J* 8.2 Hz, **H29**), 7.57 (1H, d, *J* 8.6 Hz, **H31**), 7.30 (1H, d, *J* 8.7 Hz, **H4**), 7.00 (1H, d, *J* 8.4 Hz, **H3**), 5.79 (1H, bt, **H10**), 5.68 (1H, bt, **H23**), 5.63 (1H, q, *J* 7.3 Hz, **H20**), 5.18 (1H, d, *J* 14.5 Hz, **H6'**), 4.40 (1H, q, *J* 7.2 Hz, **H7**), 4.33 (1H, d, *J* 14.8 Hz, **H6**), 4.23-4.12 (2H, m, **H33**), 3.92 (3H, s, **H1**), 3.46-3.38 (1H, m, **H25'**), 3.31-3.19 (2H, m, **H12'+H25**), 3.01-2.91 (1H, m, **H12**), 2.94 (2H, t, *J* 8.0 Hz, **H35**), 2.41 (2H, quint, *J* 7.6 Hz, **H34**), 1.74 (3H, d, *J* 7.3 Hz, **H21**), 1.71 (3H, d, *J* 7.4 Hz, **H8**).

Acid-switched dimer 13, di-protonated

ROESY, $CDCl_3$, 400 MHz, t_{mix} = 0.2 s

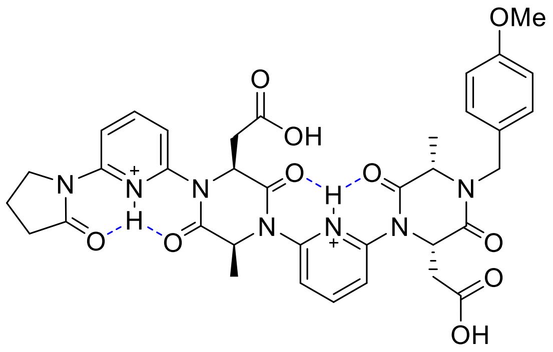

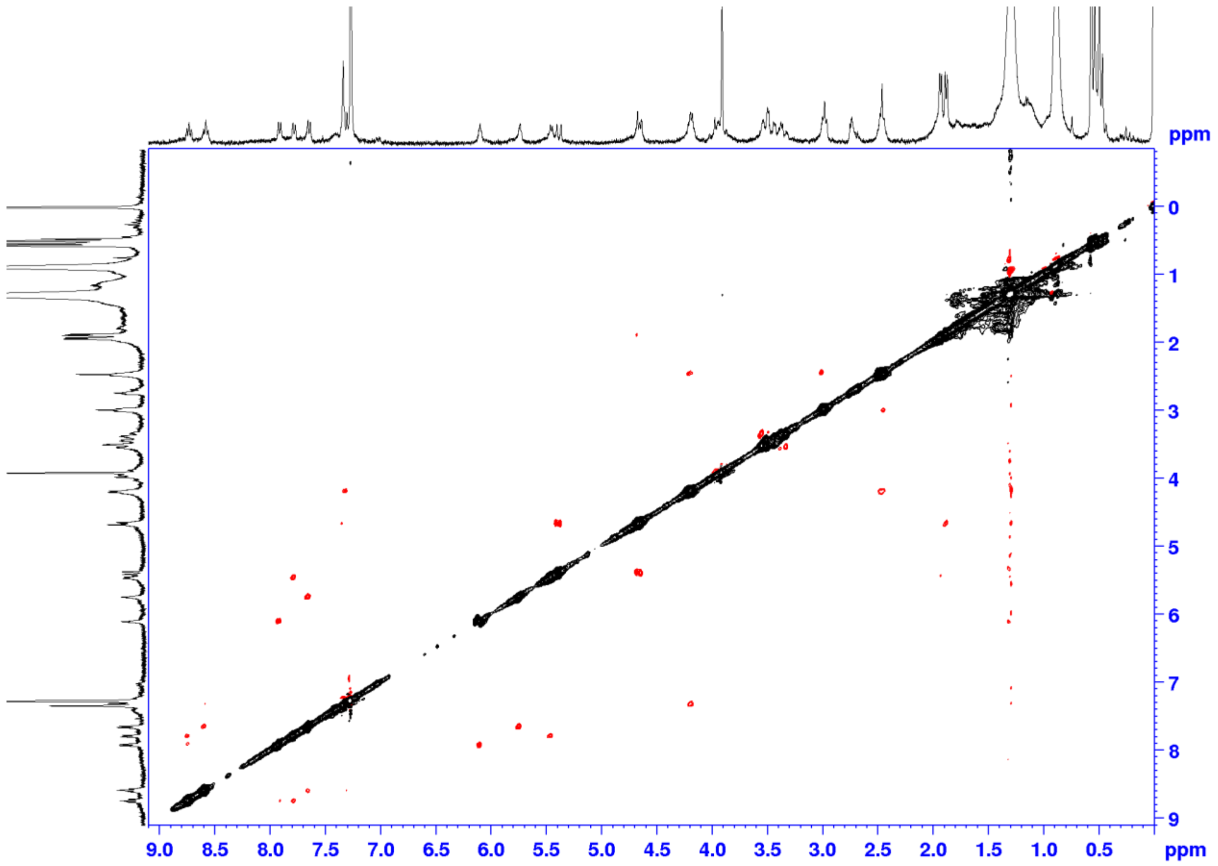

Figure S71: Full ROESY spectrum of fully acid switched dimer **13**

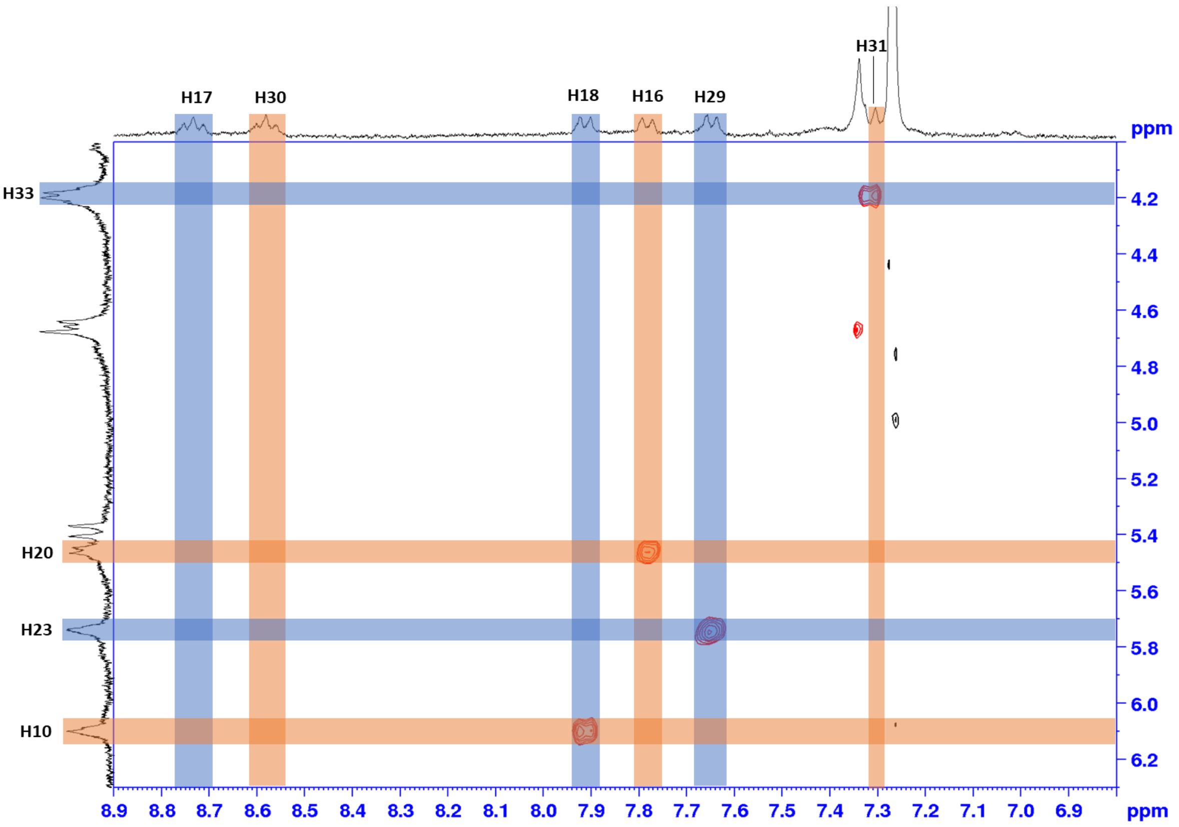

Figure S72: Zoomed area of acid switched di-protonated dimer **13**, displaying both strong cross-peaks

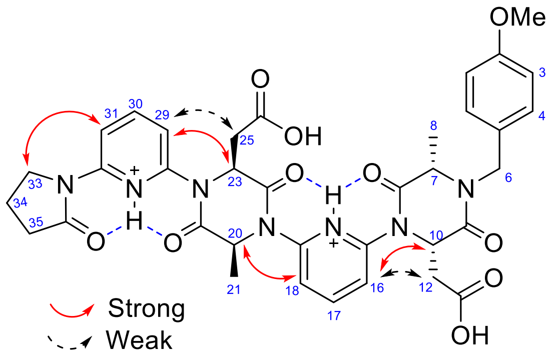

Figure S73: Protonated dimer **13** conformation stabilised through hydrogen-bonding (blue dashed lines). nOe correlations ($CDCl_3$, 400 MHz) from the ROESY spectrum confirm the predicted conformation. Solid red arrows are strong cross-peaks; dashed black arrows are weak cross-peaks.

A ROESY spectrum was obtained after the addition of both *d*-TFA and TfOH (**Table 3, Entry 9**) and the nOe correlations agreed with the fully protonated, hydrogen-bonded conformation (**Figures S71-S72**). This was indicated by the formation of strong nOe interactions between (**H20↔H18** and **H16↔H10**), which had previously been non-existent in the neutral (**Figure S28**) and monoprotonated (**Figure S68**) conformations. In addition, the strong nOe interactions between **H33↔H31** and **H29↔H23** still remained, therefore confirming that both pyridines had been protonated and had "switched" from the dipole-opposed conformation to a hydrogen-bond stabilised structure.

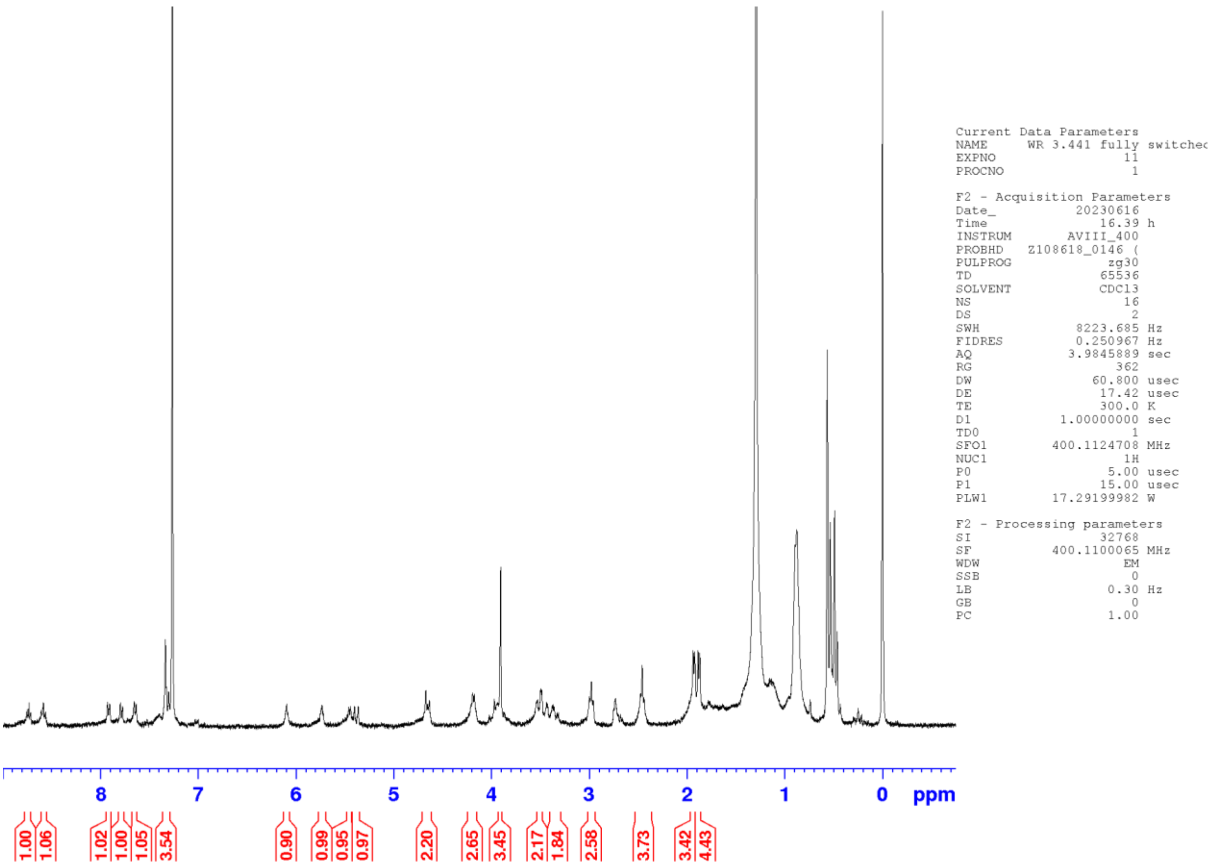

Figure S74: ^{1}H NMR of dimer **13** after addition of *d*-TFA (500 equiv.) + TfOH (8 equiv.) (400 MHz, $CDCl_3$).

δ_H (400 MHz, $CDCl_3$):* 8.73 (1H, t, *J* 8.3 Hz, **H17**), 8.58 (1H, t, *J* 8.2 Hz, **H30**), 7.91 (1H, d, *J* 8.9 Hz, **H18**), 7.78 (1H, d, *J* 8.7 Hz, **H16**), 7.65 (1H, d, *J* 8.7 Hz, **H29**), 7.36-7.29 (3H, m, **H4+H31**), 6.10 (1H, bt, **H10**), 5.74 (1H, bt, **H23**), 5.46 (1H, bq, *J* 7.3 Hz, **H20**), 5.38 (1H, d, *J* 14.7 Hz, **H6'**), 4.69-4.61 (2H, m, **H6+H7**),4.23-4.12 (2H, m, **H33**), 3.91 (3H, s, **H1**), 3.57-3.47 (2H, m, **H12'+H25'**), 3.40-3.31 (2H, m, **H12+H25**), 2.98 (2H, t, *J* 8.1 Hz, **H35**), 2.51-2.40 (2H, m, **H34**), 1.93 (3H, d, *J* 7.1 Hz, **H21**), 1.87 (3H, d, *J* 6.9 Hz, **H8**).

* Upon addition of TfOH, the peak associated with **H3** is no longer visible, presumably due to in-situ deuteration of PMB via the mechanism shown in **Scheme 3, B**

5.7 Trimer 8 Acid-Switching Experiment

The acid titration experiment was also conducted with the PMB-protected trimer species **8**. The trimer was dissolved in $CDCl_3$ (9 mM) and portions of dilute *d*-trifluoroacetic acid (*d*-TFA) (1.26 M), concentrated *d*-TFA and TfOH were added (**Table 4**). After each addition, a ^{1}H NMR spectrum was obtained and the final 'switched' conformation was analysed by Rotating Frame Overhauser Effect Spectroscopy (ROESY).

Table 4: Trimer **8** acid titration with dilute (1.26 M) *d*-TFA, concentrated *d*-TFA and TfOH.

Entry	Dilute *d*-TFA (μL)	Concentrated *d*-TFA (μL)	TfOH (μL)	Cumulative equivalent	
				d-TFA	TfOH
1	0	-	-	0	-
2	5	-	-	1	-
3	5	-	-	2	-
4	10	-	-	4	-
5	20	-	-	8	-
6	40	-	-	16	-
7	-	5	-	26	-
8	-	5	-	37	-
9	-	10	-	58	-
10	-	20	-	99	-
11	-	40	-	183	-
12	-	40	-	266	-
13	-	50	-	370	-
14	-	100	-	578	-
15	-	100	-	786	-
16	-	200	-	1200	-
17	-	-	0.6	1200	1
18	-	-	0.6	1200	2
19	-	-	1.1	1200	4
20	-	-	2.2	1200	8
21	-	-	4.4	1200	16

Sequential and reversible switching of monomer units was also observed during the trimer titration. After 1200 equivalents of *d*-TFA (**Table 4**, **Entry 16**), only the terminal pyridine was observed to have "switched" conformation by ^{1}H NMR and ROESY spectroscopy. Full conformational change was achieved after the addition of 16 equivalents of TfOH (**Table 4, Entry 21**).

As increasing amounts of acid were added, significant shifting of the pyridine peaks was observed by ^{1}H NMR (**Figure S75**). The pyridine *para* hydrogen peaks (**H19**, **H34** and **H49**) shifted downfield by 0.7-0.9 ppm whereas the *meta* hydrogen peaks shifted upfield. The terminal *meta* hydrogen (**H50**) shifted considerably upfield by 1.2 ppm whereas the other *meta* hydrogens (**H18**, **H20**, **H33**, **H35** and **H48**) shifted slightly by 0.10-0.30 ppm. These trends were consistent with those observed during the dimer titration.

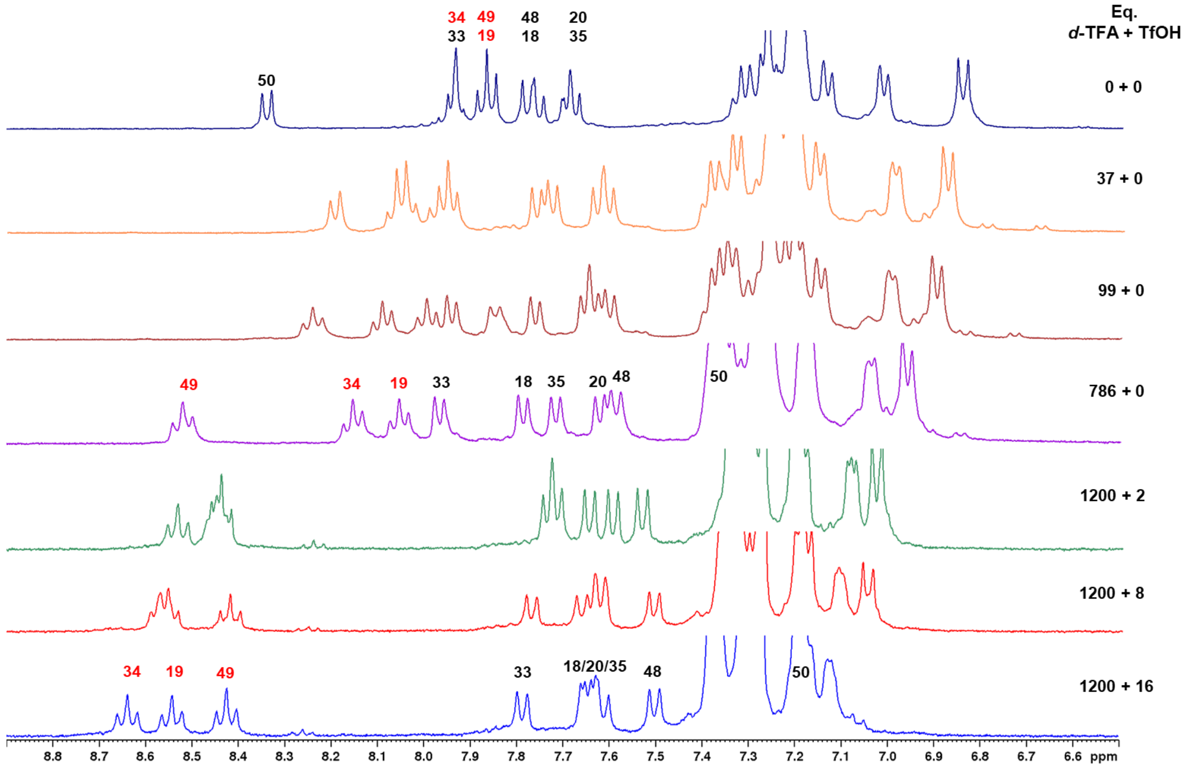

Figure S75: Overlaid ^{1}H NMR spectra showing the shifting of trimer **8** pyridine peaks as increasing equivalents of acid (*d*-TFA and TfOH) were added.

5.7.1 Neutralisation/Reversal of the Conformationally Switched Trimer 8

The trimer was also shown to reversibly change upon treatment of the protonated species in $CDCl_3$ with saturated, aqueous sodium bicarbonate resulting in conversion back to the dipole-opposed conformation. (**Figure S76**) The trimer also underwent the same deuteration of the hydrogens *ortho* to the methoxy group of the PMB moiety, as seen with dimer **6**, (**Scheme 3, A**).

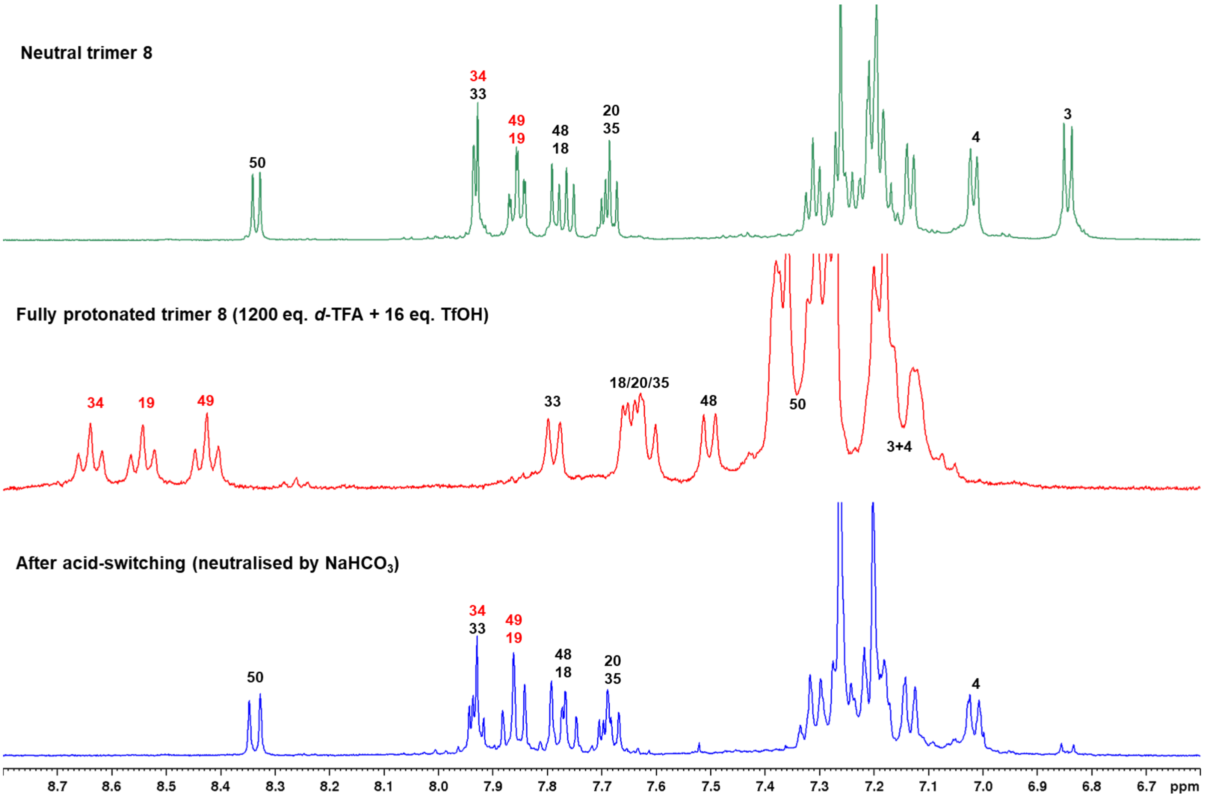

Figure S76: Overlaid ^1H NMR spectra showing the shifting of trimer **8** pyridine peaks from neutral, to fully protonated, and after neutralisation with $NaHCO_3$

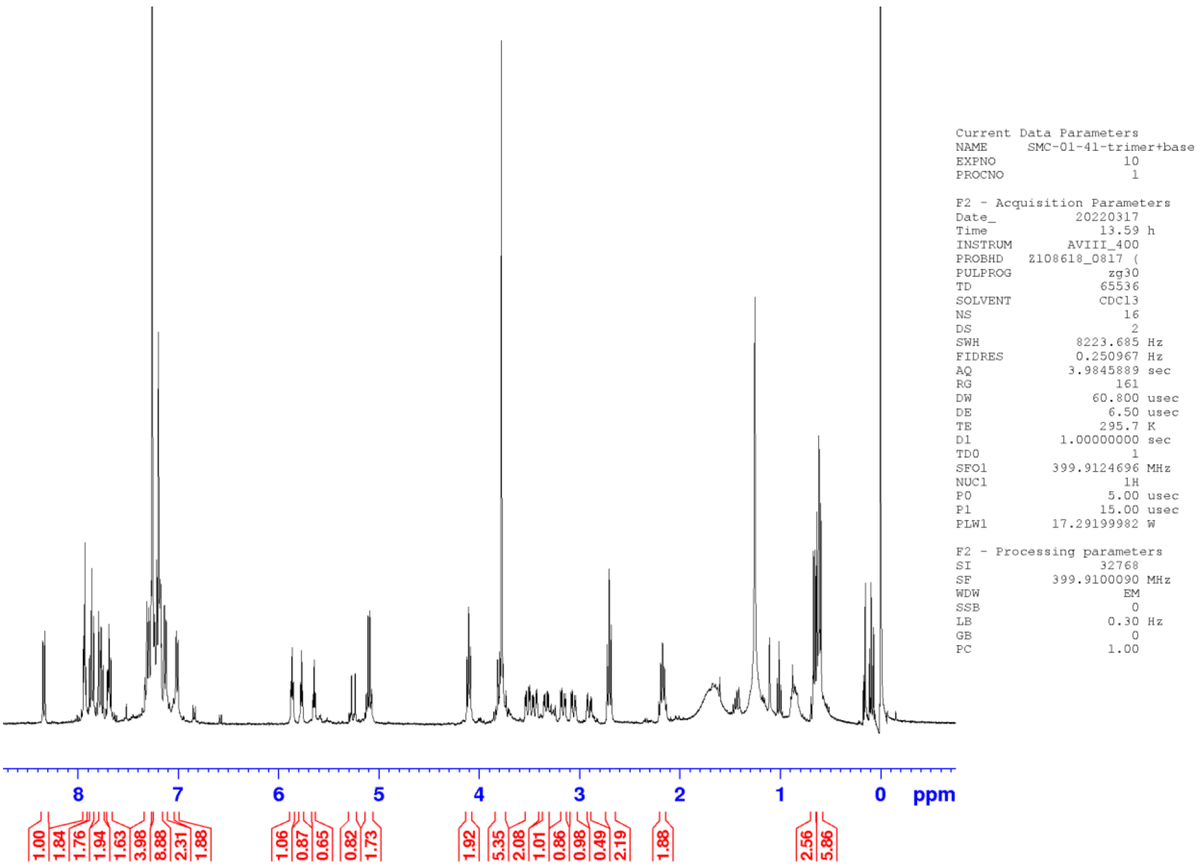

Figure S77: ^{1}H NMR of neutral trimer **8** after $NaHCO_3$ washes.

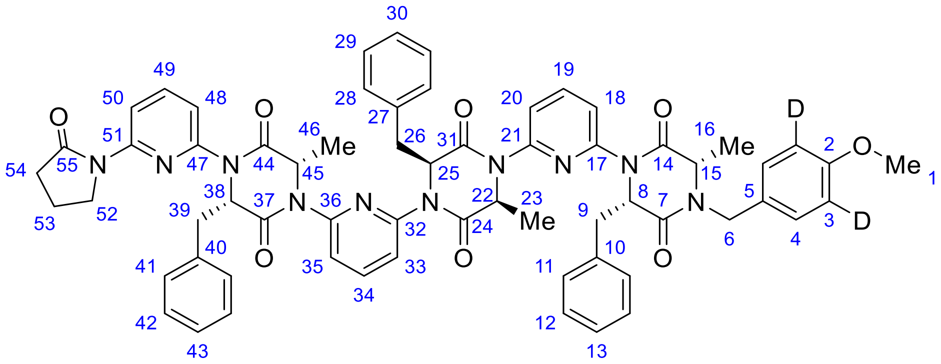

δ_H (400 MHz, $CDCl_3$): 8.34 (1H, d, *J* 8.1 Hz, **H50**), 7.95-7.91 (2H, m, **H33**, **H34**), 7.88-7.83 (2H, m, **H19**, **H49**), 7.80-7.74 (2H, m, **H18**, **H48**), 7.71-7.66 (2H, m, **H20**, **H35**), 7.35-7.16 (13H, m, **H4**, **H12-13**, **H41-43**, **H29-30**), 7.13 (2H, d, *J* 7.0 Hz, **H28**), 7.02 (2H, d, *J* 8.0 Hz, **H11**), 5.87 (1H, t, *J* 4.5 Hz, **H38**), 5.77 (1H, t, *J* 4.5 Hz, **H25**), 5.65 (1H, t, *J* 4.4 Hz, **H8),** 5.25 (1H, d, *J* 14.6 Hz, **H6'**), 5.10 (2H, q, *J* 7.1 Hz, **H22**, **H45**), 4.10 (2H, t, *J* 7.5 Hz, **H52**), 3.83-3.78 (2H, m, **H6**, **H15**), 3.78 (3H, s, **H1**), 3.52 (1H, dd, *J* 13.9, 4.0 Hz, **H39'**), 3.45 (1H, dd, *J* 14.0, 4.4 Hz, **H26'**), 3.33 (1H, dd, *J* 14.0, 4.3 Hz, **H9'**), 3.17 (1H, dd, *J* 14.0, 5.0 Hz, **H39**), 3.06 (1H, dd, *J* 13.9, 4.7 Hz, **H26**), 2.90 (1H, dd, *J* 14.0, 4.6 Hz, **H9**), 2.70 (2H, t, *J* 8.1 Hz, **H54**), 2.21-2.13 (2H, quint, *J* 7.6 Hz, **H53**), 0.66 (3H, d, *J* 7.1 Hz, **H23**), 0.64-0.63 (6H, m, **H16***,* **H46**).

5.8 Trimer 8 Acid-Switching Conformational Control Spectra

Acid-switched trimer 8, mono-protonated

ROESY, $CDCl_3$, 600 MHz, t_{mix} = 0.2 s

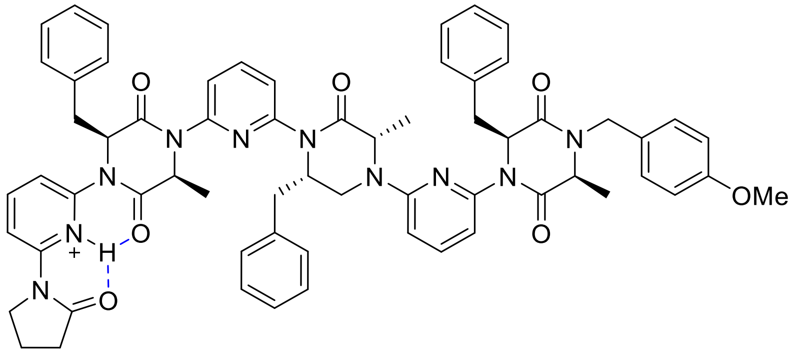

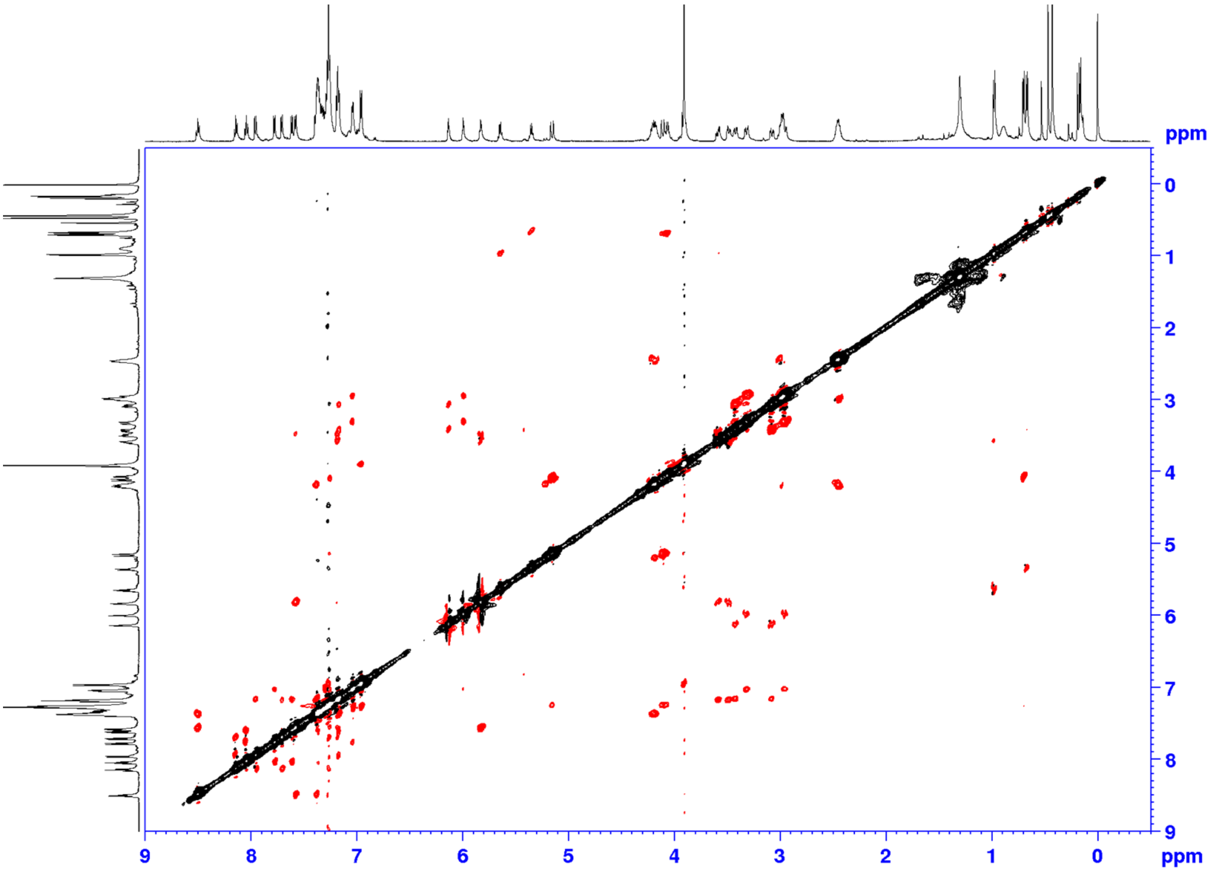

Figure S78: Full ROESY spectrum of mono-protonated acid-switched trimer **8**

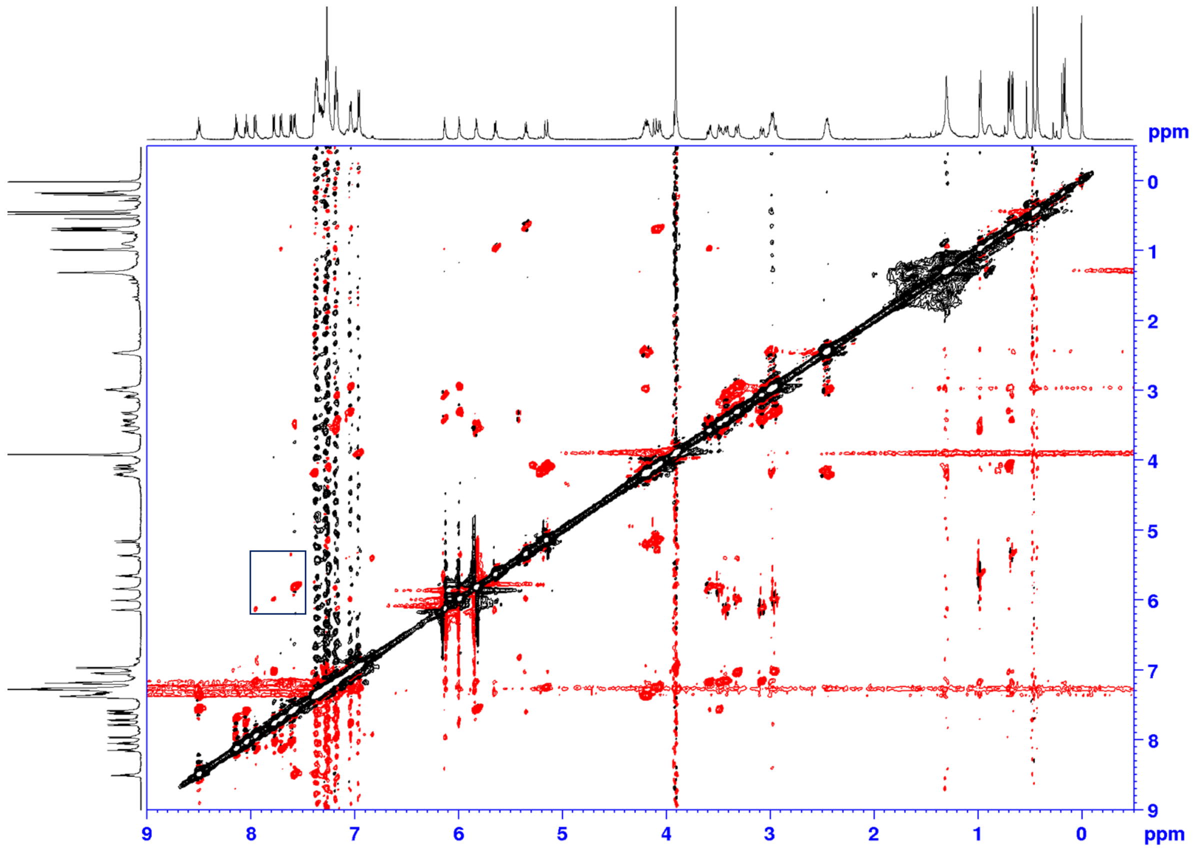

Figure S79: Full ROESY spectrum of mono-protonated acid-switched trimer **8,** with weak interactions

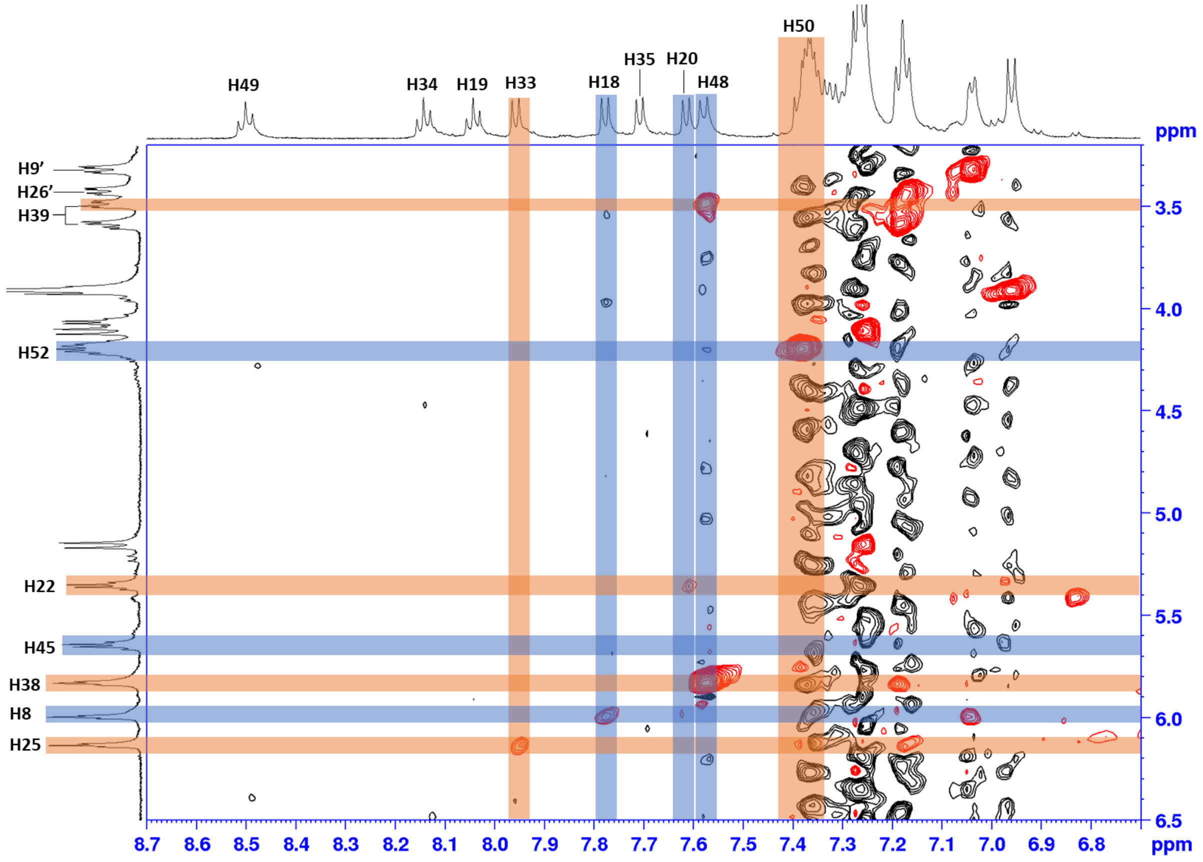

Figure S80: Zoomed area of acid switched mono-protonated trimer **8**, displaying both weak and strong cross-peaks

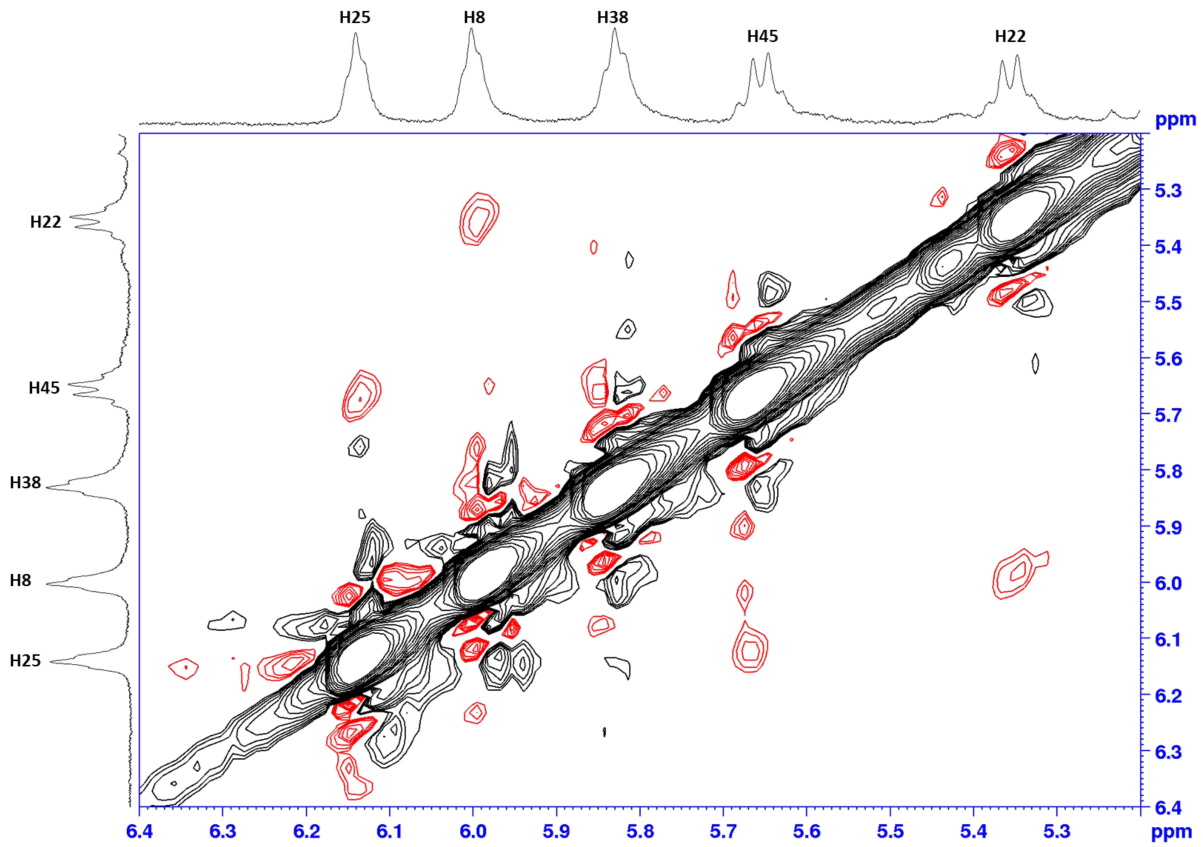

Figure S81: Zoomed area of acid switched mono-protonated trimer **8**, displaying strong inter-residue (**H45↔H25** and **H22↔H8**) cross-peaks

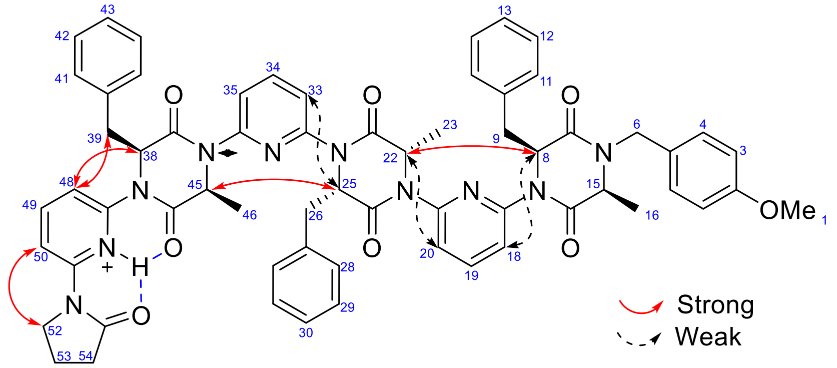

Figure S82: Mono-protonated trimer **8** conformation stabilised through hydrogen-bonding (blue dashed lines). nOe correlations ($CDCl_3$, 600 MHz) from the ROESY spectrum confirm the predicted conformation. Solid red arrows are strong cross-peaks; dashed black arrows are weak cross-peaks.

After the addition of 786 equivalents of *d*-TFA (**Table 4, Entry 15**), a ROESY spectrum was obtained which indicated that only one of the pyridine moieties had undergone protonation. This was evident by the appearance of strong interactions between the terminal pyridine *meta* hydrogens and pyrrolidinone/diketopiperazine groups (**H52↔H50**, **H48↔H38**, **H48↔H39**) (**Figure S79-80**) whilst the nOe interactions between (**H33↔H25**, **H22↔H20**, **H18↔H8**) remained identical i.e., very weak, to those observed interaction in its neutral form.

(**Figure 15-17**) This stark contrast in nOe intensities is therefore consistent with a partially switched conformation.

Akin to the strong inter-residue nOe present in dimer **6**, analogues inter-reside nOe interactions were also present between the individual DKP unit, with interactions between **H45↔H25** and **H22↔H8** being observed. (**Figure S81**) Again, this in in good agreement with a partially switched/mono-protonated conformation, as in a fully protonated state these interactions would not be present due to the increased distance between the adjacent protons.

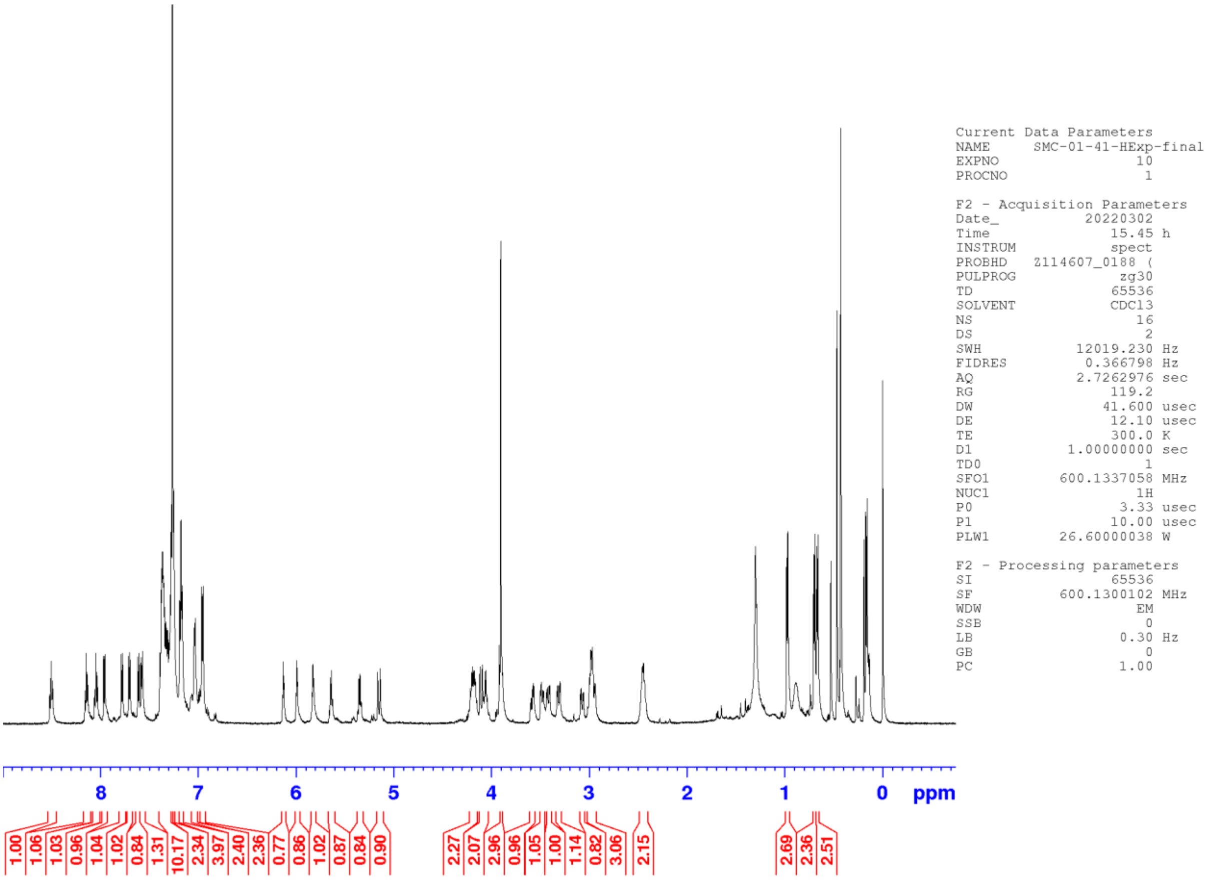

Figure S83: ^{1}H NMR of trimer **8** after addition of *d*-TFA (786 equiv.) (600 MHz, $CDCl_3$).

δ$_H$ (600 MHz, $CDCl_3$): 8.50 (1H, t, *J* 8.3 Hz, **H49**), 8.14 (1H, t, *J* 8.0 Hz, **H34**), 8.04 (1H, t, *J* 7.8 Hz, **H19**), 7.96 (1H, d, *J* 8.0 Hz, **H33**), 7.78 (1H, d, *J* 8.0 Hz, **H18**), 7.71 (1H, d, *J* 7.9 Hz, **H35**), 7.61 (1H, d, *J* 8.1 Hz, **H20**), 7.57 (1H, d, *J* 8.2 Hz, **H48**), 7.41-7.21 (12H, m, **H4, H12-13, H29-30, H42-43, H50**),7.19-7.13 (4H, m, **H28+H41**), 7.05-7.00 (2H, m, **H11**), 6.65 (2H, d, *J* 8.2 Hz, **H3**), 6.14 (1H, bt, **H25**), 6.00 (1H, bt, **H8**), 5.83 (1H, bt, **H38**), 5.65 (1H, q, *J* 7.2 Hz, **H45**), 5.36 (1H, q, *J* 7.3 Hz, **H22**), 5.15 (1H, d, *J* 14.7 Hz, **H6'**), 4.24-4.12 (2H, m, **H52**), 4.12-4.00 (2H, m, **H6 + H15**), 3.90 (3H, s, **H1**), 3.58 (1H, dd, *J* 14.2, 5.6 Hz, **H39'**), 3.48 (1H, dd, *J* 14.4, 4.5 Hz, **H39**), 3.42 (1H, dd, *J* 14.6, 4.6 Hz, **H26'**), 3.31 (1H, dd, *J* 14.4, 4.5 Hz, **H9'**), 3.07

(1H, dd, *J* 14.5, 4.5 Hz, **H25**), 3.02-2.92 (3H, m, **H9+H54**), 2.44 (2H, quint, *J* 4.7 Hz, **H53**), 0.94 (3H, d, *J* 7.3 Hz, **H46**), 0.67 (3H, d, *J* 7.0 Hz, **H16**), 0.63 (3H, d, *J* 7.0 Hz, **H23**).

Acid-switched trimer 8, tri-protonated

ROESY, $CDCl_3$, 600 MHz, t_{mix} = 0.2 s

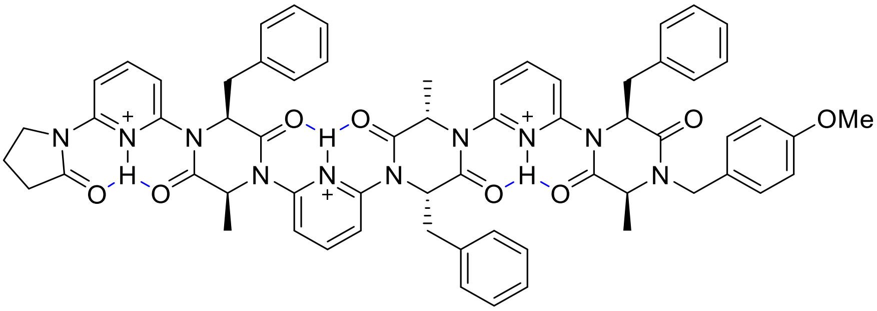

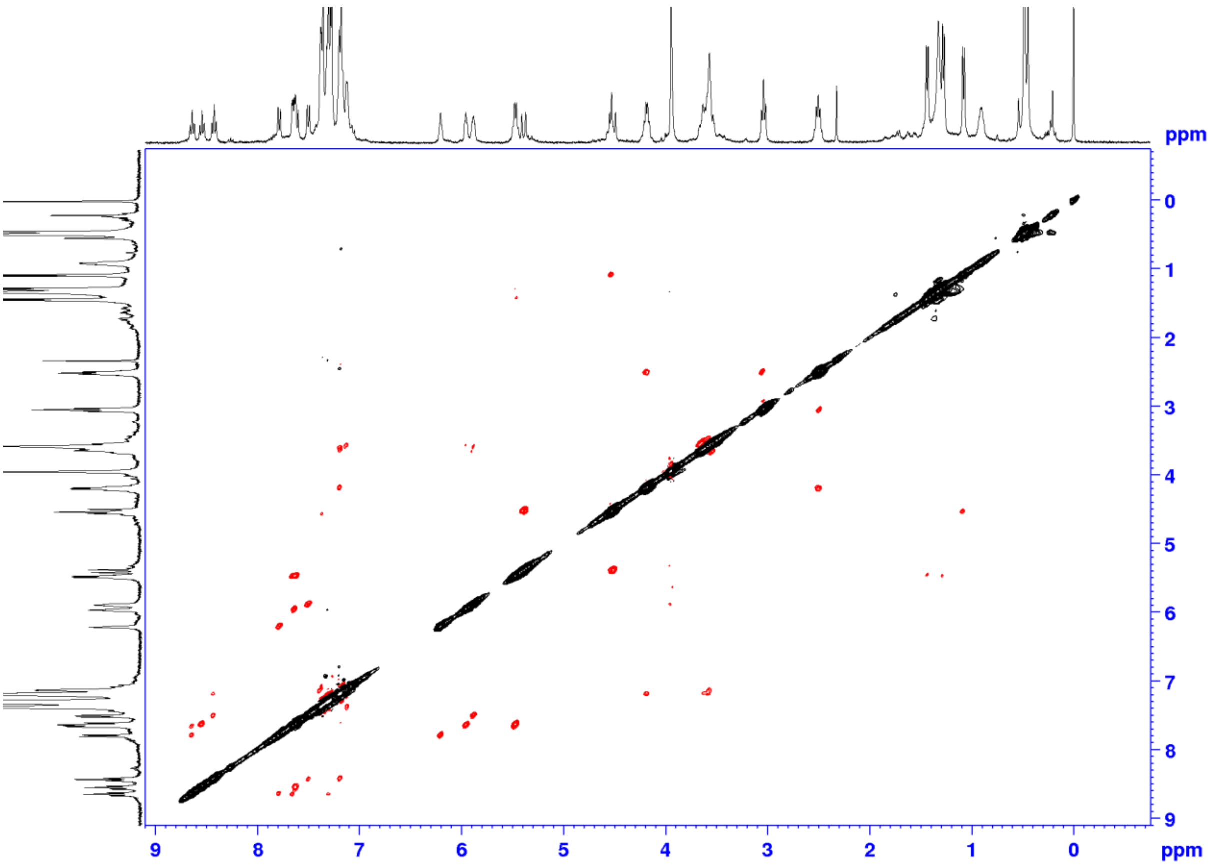

Figure S84: Full ROESY spectrum of tri-protonated acid switched trimer **8**

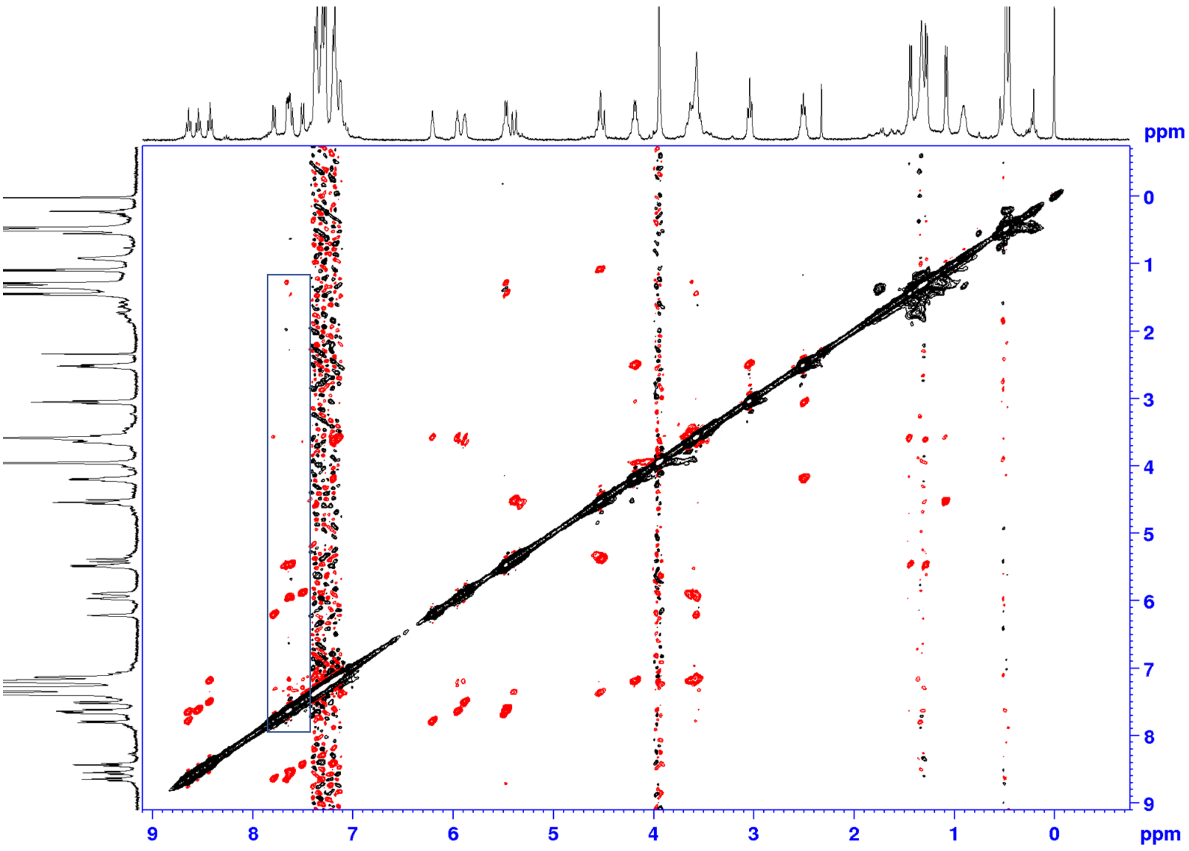

Figure S85: Full ROESY spectrum of tri-protonated acid switched trimer **8**, with weak interactions

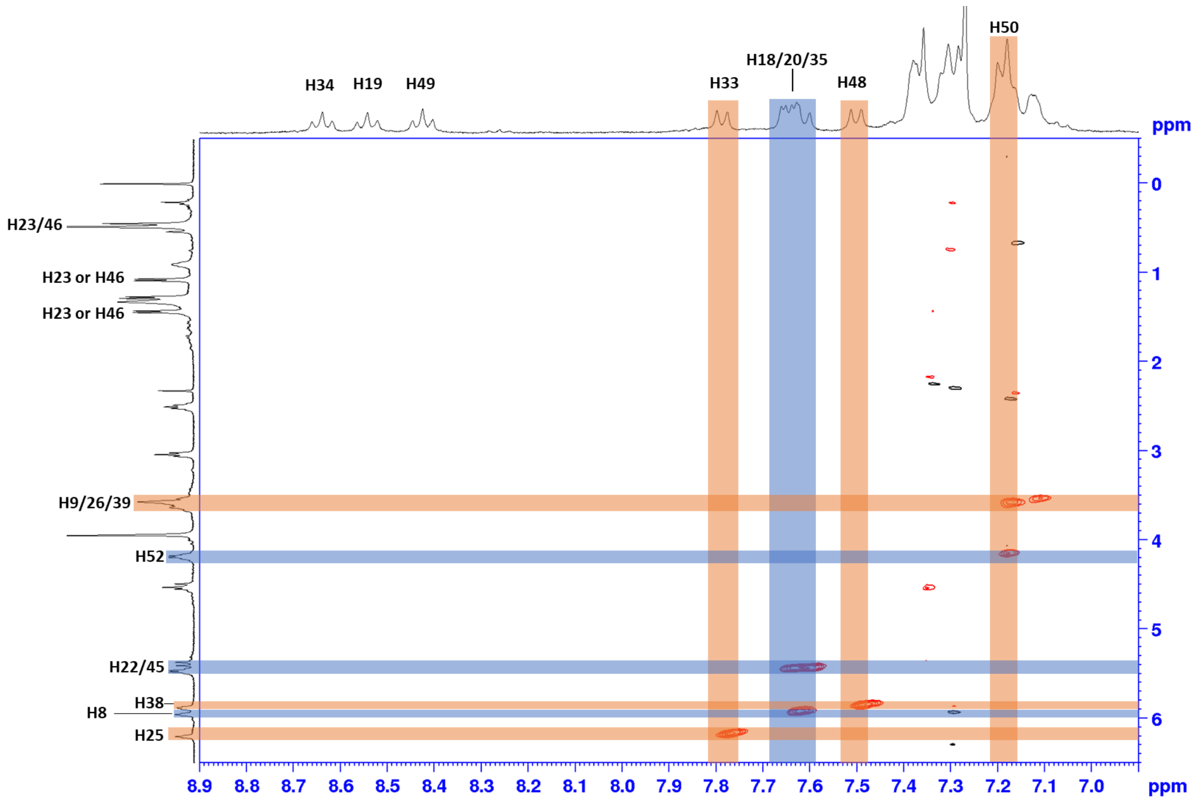

Figure S86: Zoomed area of acid switched trimer **8**, displaying strong cross-peaks

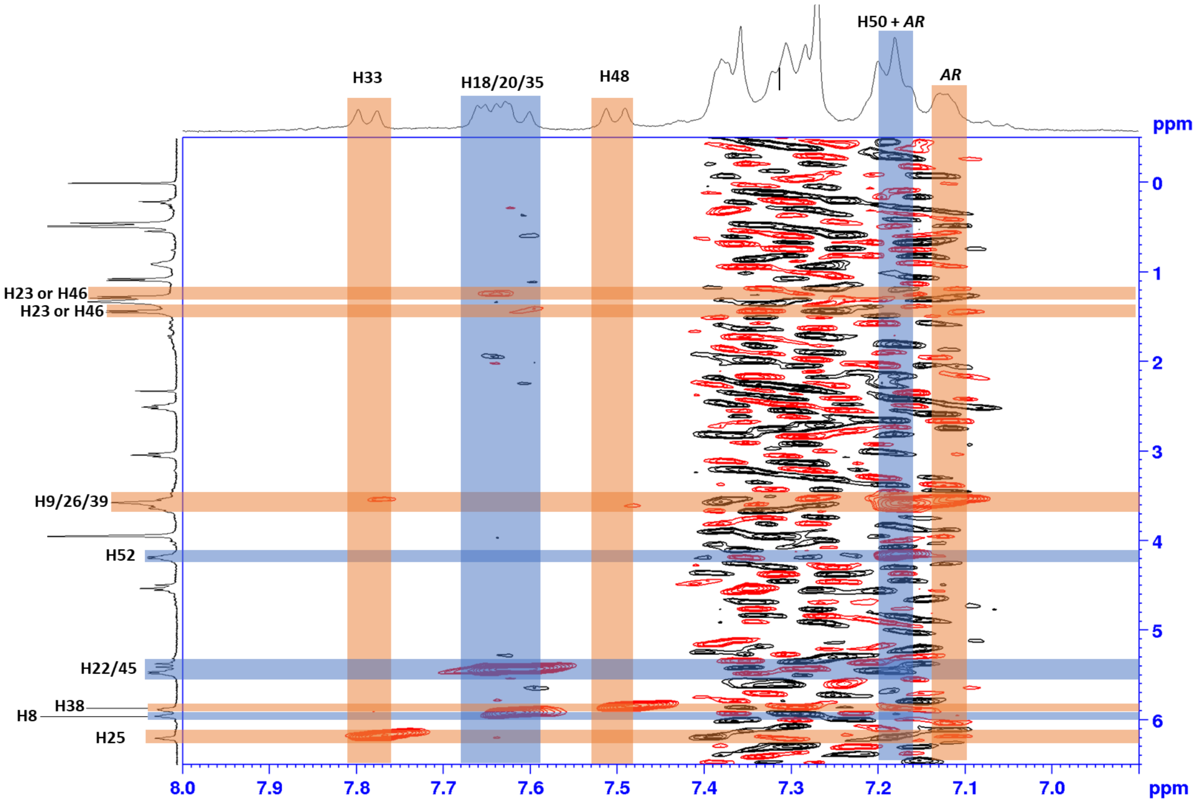

Figure S87: Zoomed area of acid switched trimer **8**, displaying both weak and strong cross-peaks

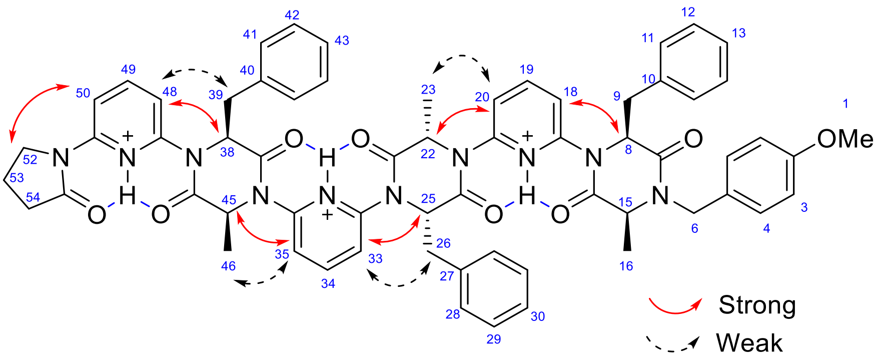

Figure S88: Protonated trimer **8** conformation stabilised through hydrogen-bonding (blue dashed lines). nOe correlations ($CDCl_3$, 600 MHz) from the ROESY spectrum confirm the predicted conformation. Solid red arrows are strong cross-peaks; dashed black arrows are weak cross-peaks.

The ROESY spectrum was examined to confirm the predicted protonated conformation. Strong cross-peaks were observed between the pyrrolidinone and pyridine hydrogen atoms (**H52↔H50**), in addition to strong cross-peaks between the pyridine *meta* hydrogens and the diketopiperazine hydrogens (**H48↔H38**, **H45↔H35**, **H33↔H25**, **H22↔H20**, **H18↔H8**), (**Figures S84-S86**) and weak cross-peaks between the pyridine meta hydrogens and adjacent sidechain functionality; CH_3 (**H46↔H35** and **H23↔H20**) and CH_2Ph (**H48↔H39** and **H33↔H27**). (**Figure S87**) Thereby indicating these pyridine unit had also undergone protonation. These results further indicated that protonation was causing a switch from the dipole-opposed structure to a hydrogen-bonded structure whilst retaining the overall zig-zag

shape. Another interesting feature observed upon the acid-mediated conformational change was that the diketopiperazine sidechains were now projected from opposite faces than in the dipole-opposed structure.

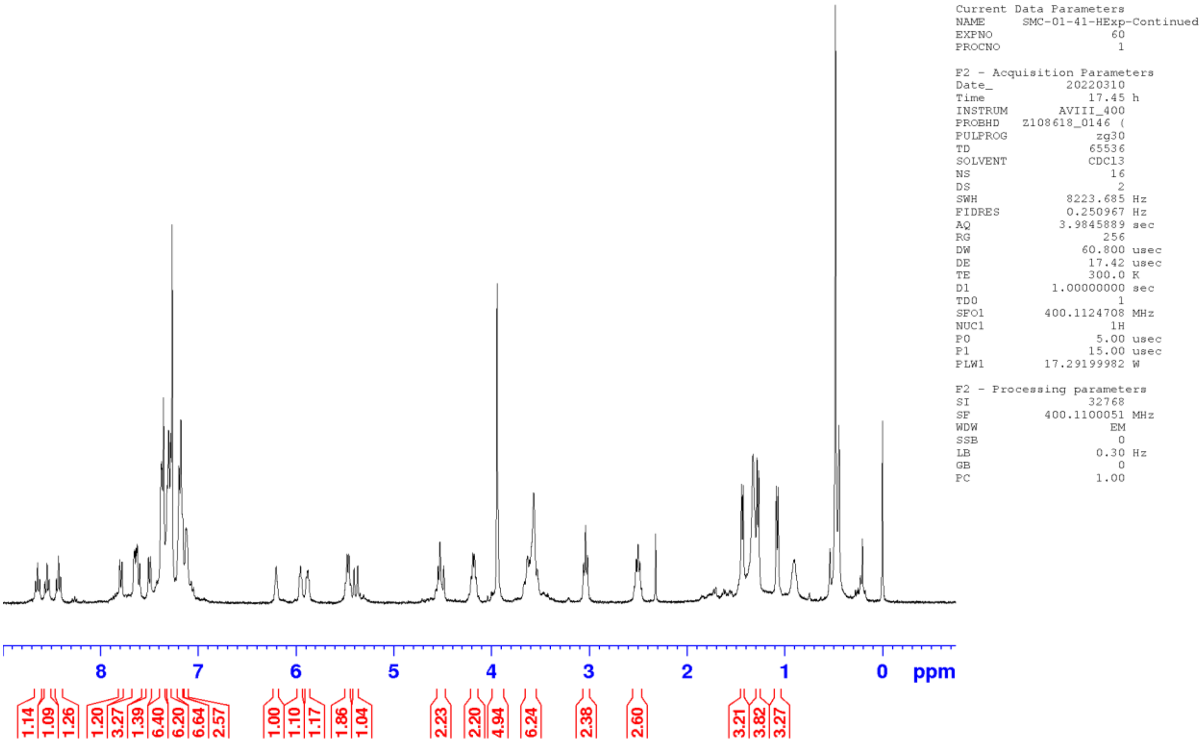

Figure S89: ^{1}H NMR of trimer **8** after addition of *d*-TFA (1200 equiv.) + TfOH (16 equiv.) (400 MHz, $CDCl_3$).

δ_H (400 MHz, $CDCl_3$): 8.64 (1H, t, *J* 8.6 Hz, **H34**), 8.54 (1H, t, *J* 8.8 Hz, **H19**), 8.42 (1H, t, *J* 8.6 Hz, **H49**), 7.78 (1H, d, *J* 8.8 Hz, **H33**), 7.69-7.53 (3H, m, **H18**, **H20**, **H35**), 7.50 (1H, d, *J* 8.7 Hz, **H48**), 7.42-6.99 (20H, m, **H3-4**, **H11-13**, **H28-30**, **H41-43, H50**), 6.20 (1H, bt, **H25**), 5.95 (1H, bt, **H8**), 5.90-5.76 (1H, m, **H38**), 5.55-5.43 (2H, m, **H22**, **H45**), 5.39 (1H, d, *J* 14.6 Hz, **H6'**), 4.55-4.47 (2H, m, **H6**, **H15**), 4.26-4.14 (2H, m, **H52**), 3.94 (3H, s, **H1**), 3.64-3.54 (6H, m, **H9**, **H26**, **H39**), 3.03 (2H, t, *J* 8.2 Hz, **H54**), 2.50 (2H, quint, *J* 7.7 Hz, **H53**), 1.42 (3H, d, *J* 7.3 Hz, **H23/H46**), 1.27 (3H, d, *J* 7.4 Hz, **H23/H46**), 1.07 (3H, d, *J* 7.4 Hz, **H16**).

6 Further Discussion on the Issue of Epimerisation of 10

Incompatibility of Buchwald-Hartwig cross coupling of aspartic acid-derived monomer 3b with 2-pyrrolidinone

During the initial synthesis of the homo-oligomers of aspartic acid-derived monomer it was discovered when trying to form the terminal unit **10** via Buchwald-Hartwig cross-coupling, formation of the desired product was hampered by epimerisation. Initially, on a small 50 mg scale, the reaction behaved as expected, but scaling the reaction led to significant epimerisation (3:1), (**Scheme 4**) something that had not been observed for the phenylalanine/alanine series or in the synthesis of aspartic acid-derived coupling unit, **3b**. This would therefore compromise the stereochemical integrity of the larger oligomers, making precise control of the project of the sidechain impossible and the synthesis intractable.

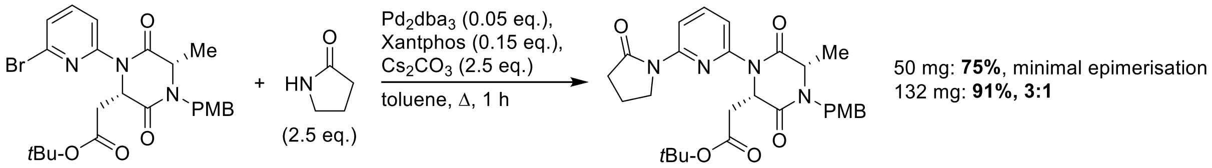

Scheme 4: Synthesis of monomer **10** under the standard Buchwald-Hartwig cross-coupling conditions

This issue of stereochemical robustness may be a result of the electron withdrawing *tert*-butyl ester moiety, which would increase the acidity of the α-proton making deprotonation and therefore epimerisation more facile. However, it remains unclear why such epimerization is not observed in the synthesis of monomer **3b** itself.

Therefore, alternative Buchwald-Hartwig cross-coupling conditions featuring diketopiperazine moieties were then examined. The first was replacing Xantphos with XPhos, this was in response to work by Boyd and Sperry *et al.*[22] who observed epimerisation of DKPs during Pd-catalysed *N*-arylation of indoles. (**Scheme 5**) They found that replacing Xantphos with XPhos led to improved reactivity and reduced epimerisation.

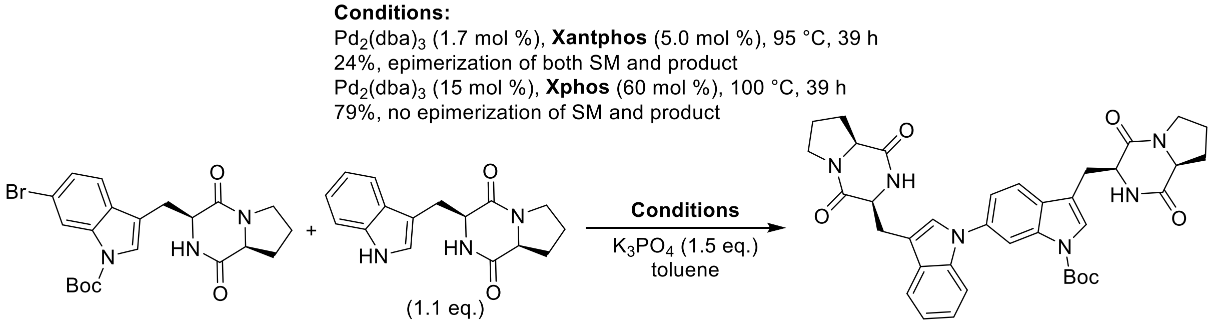

Scheme 5: Sperry *et al.*'s Pd-catalysed *N*-arylation of indoles bearing a DKP motif.

Initially, only the ligand was changed, with Cs_2CO_3 remaining as the base, and pleasingly this didn't affect reactivity, with consumption of the starting material being complete within an hour (**Scheme 6**). However, the use of XPhos did not supress epimerisation, even on a 50 mg scale, with it occurring in a 10:1 ratio. Therefore, the identical base (K_3PO_4) used in Sperry *et al.*'s initial report was examined, hoping that the weaker base may help further supress epimerisation. However, epimerisation of the product remained, albeit it to a slightly lesser extent.

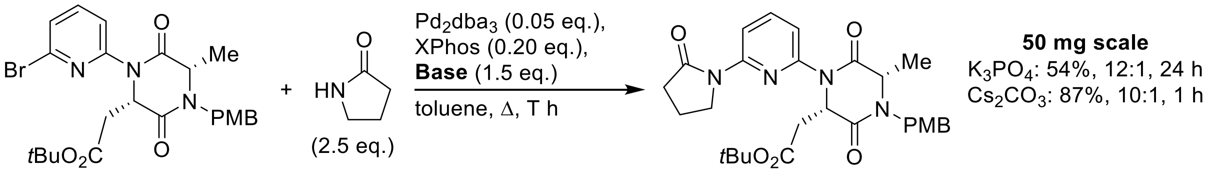

Scheme 6: Screening the use of XPhos to minimise epimerisation of **10** under the standard Buchwald-Hartwig cross-coupling conditions.

Following this the use of *t*-BuBrettPhos was examined, in the form of pre-catalyst complex ***t*-BuBrettPhos G3.** However, this too had no influence on supressing the epimerisation, with a 4.7: ratio of epimers forming when performed on a 115 mg scale. (**Scheme 7**)

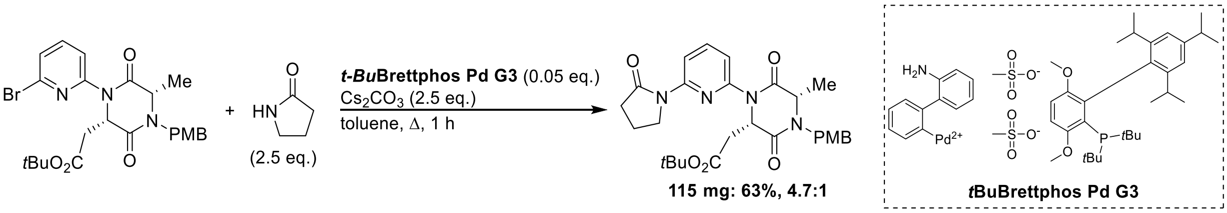

Scheme 7: Screening the use of *t*-BuBrettPhos to minimise epimerisation of **10** under the standard Buchwald-Hartwig cross-coupling conditions.

Lastly, as the use of $Pd_2(dba)_3$ with Xantphos or dialkylbiaryl phosphine ligands had no positive influence on epimerisation, alternative Buchwald-Hartwig cross-coupling conditions featuring a starting material with a stereocenter adjacent to an amine/carbonyl and using a different palladium sources and ligand was examined. Hence the use of $Pd(OAc)_2$ and (*R*)-BINAP was trialled, with Schmitt *et al.*[23] using these conditions in the *N*-heteroarylation α-aminoesters, with no epimerization being noted. (**Scheme 8**)

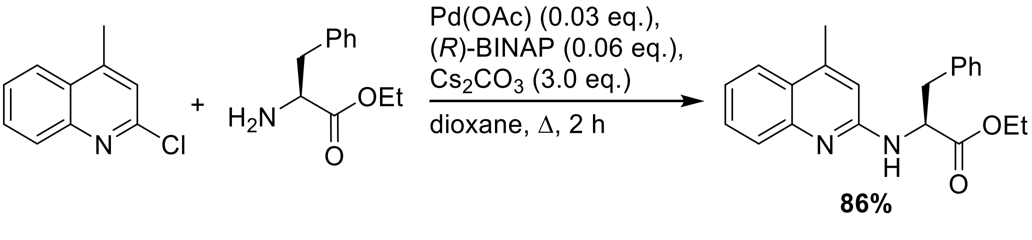

Scheme 8: Schmitt *et al.*'s *N*-heteroarylation of α-aminoesters

However, like the previous attempts, the use of $Pd(OAc)_2$ and (*R*)-BINAP had no influence on supressing epimerization, resulting in the greatest amount of epimerization seen in all of the conditions examined. (**Scheme 9**) This therefore indicated that Buchwald-Hartwig cross-coupling would be incompatible with the synthesis of aspartic acid-derived monomer **3b**, and that another synthetic tactic was required.

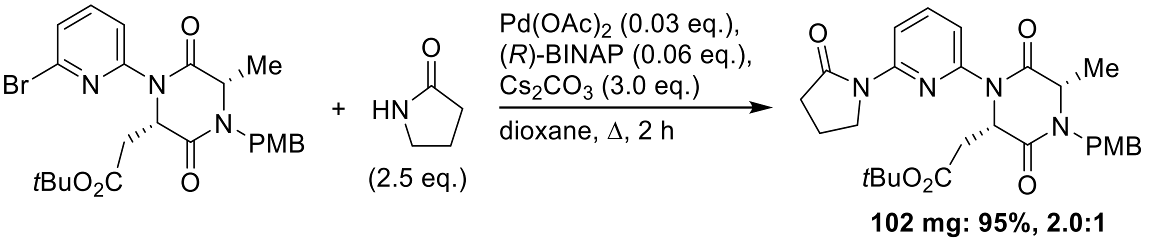

Scheme 9: Attempted synthesis of **10** using Schmitt *et al.*'s Buchwald-Hartwig conditions.

As typical Buchwald-Hartwig cross-coupling conditions had proved to be incompatible we looked at alternative methods aryl and amide cross-couplings, with Goldberg/Ullman-type coupling a being a potential alternative. Rajanbabu *et al.*[24] have reported the superiority of copper *vs*. palladium-catalysed N-arylation of diketopiperazines in avoiding epimerization.

Looking at examples of Goldberg/Ullman-type cross-coupling being utilised for 2-pyrrolidiones and diketopiperazine moieties, a few can be seen utilising CuI, DMEDA, and K_2CO_3 with success in good to high yields, and therefore these sets of conditions were examined. (**Scheme 10**)

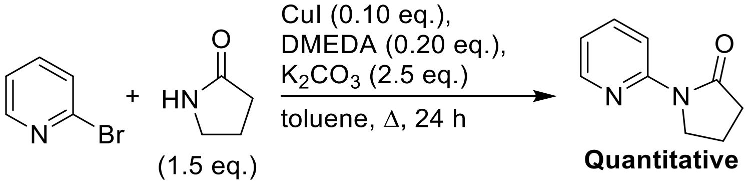

Scheme 10: Shibata *et al.* *N*-arylation of 2-pyrrolidione using Goldberg cross-coupling

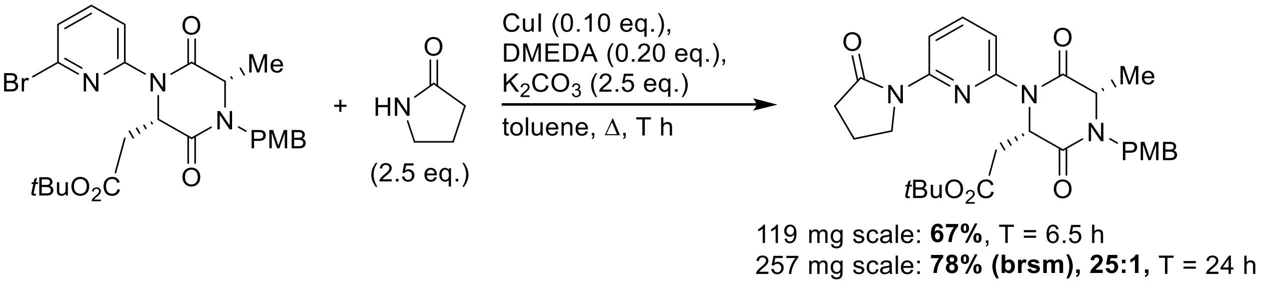

Scheme 11: Goldberg cross-coupling of monomer **10** with 2-pyrrolidione

Using Shibata *et al.*[8] (**Scheme 10**) cross-coupling conditions, synthesis of **10** was then examined on 119 mg scale. Pleasing, monomer **3b** and 2- pyrrolidinone could readily be

prepared in a yield of 67%, without any epimerization being detected. (**Scheme 11**) The reaction was therefore scaled up, as with $Pd_2(dba)_3$/Xantphos/Cs_2CO_3, epimerization was more prevalent at a larger scale. However, upon scaling up the reaction to 257 mg, a larger portion of starting material was still visible after 7 hours, therefore a second batch of CuI (0.15 equiv.) and DMEDA (0.30 equiv.) was added to the reaction mixture and left to heat for a further 14 h. Although this did improve consumption of the starting material, a small amount of starting material was still visible via TLC. Therefore, the reaction was therefore prematurely stopped in case extended heating resulted in epimerization. From the crude NMR a small amount of epimerisation occurred (25:1), however, this minor epimer could easily be removed via flash-column chromatography, in addition to the unreacted starting material being easily separated as well.

As synthesis of the terminal monomer **10** had been successful, formation of the dimer was then examined under the same conditions, with deprotected monomer **11** undergoing cross-coupling to **3b**, in a moderate yield of 45% at 20 mg scale (**Scheme 12**). By contrast, when synthesis of **12** was attempted under palladium-catalysed conditions an intractable mixture of presumed diastereomers was formed.

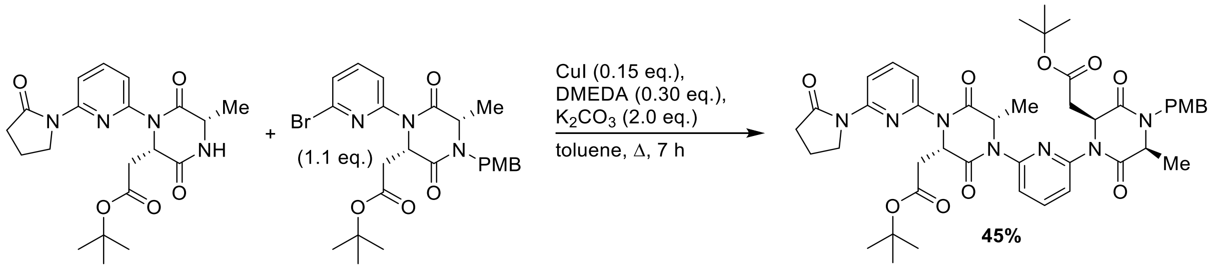

Scheme 12: Goldberg cross-coupling to synthesise the aspartic acid-derived DKP dimer **12**

The dimer synthesis was then scaled up, and it was at this point we discovered that the reaction was found to be somewhat sensitive to the purity of the copper catalyst, with the reaction not proceeding with the previously used and older CuI (98%, Sigma-Aldrich). Therefore, a fresh and newly purchased source of CuI (99.999%, Sigma-Aldrich) was utilised on the scale-up, with synthesis of the dimer being achieved in a yield of 64%, albeit with a slightly longer reaction time of 24 hours. (**Scheme 13**)

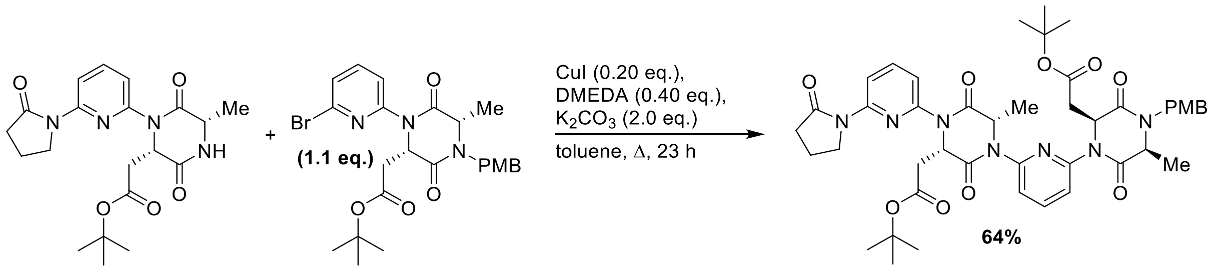

Scheme 13: Repeating of Goldberg cross-coupling to from dimer **12**, using fresh sample of CuI (99.999%)

This new CuI iodide was also utilised in an additional synthesis of terminal unit **10** (198 mg), (**Scheme 14**) using 0.20 equiv. of CuI and 0.40 equiv. of DMEDA. Again, the new CuI resulted in improved reactivity, with full consumption of the starting material after 24 hours, and a slight improvement in the yield to 79%, with no noticeable epimerization. Further demonstrating the dependence of the reaction towards the quality and age of the CuI source used.

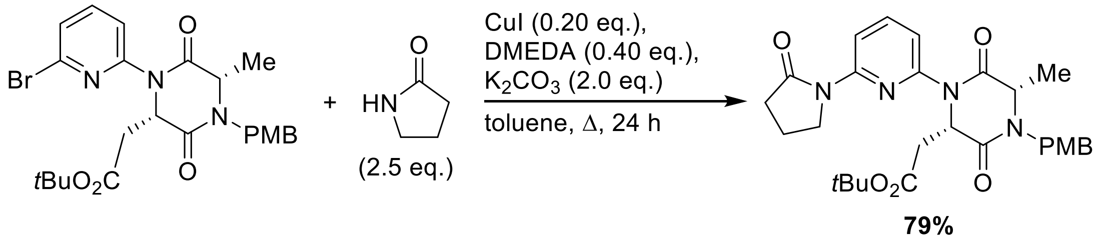

Scheme 14: Repeating of Goldberg cross-coupling to from monomer **10**, using fresh sample of CuI

7 Infrared Spectroscopy

To help further elucidate potential difference in the solid and solution-phase conformation of foldamer's neutral conformation, the use of FT-IR was examined to see if any changes in the IR-spectrum between the two states was observable, and if would relate to any potential difference in conformation between the two.

This analysis was carried out using an Agilent Cary 630 spectrometer equipped with either an attenuated total reflectance (ATR) accessory for the solid samples (using the neat solid) or the DialPath accessory for the solution phase, with the samples being deposited as a 0.1 M solution in $CHCl_3$, with a background scan of $CHCl_3$ ran before each sample to minimise absorbances resulting from $CHCl_3$.

7.1 IR Spectra of Neutral Foldamers

We believed the amide peaks might be particularly diagnostic of conformation, since they should be subject to electronic effects originating from conjugation with the adjacent azenes and dipolar repulsion. Looking at the spectra below of the monomer **4** (**Figures S90-S91**), dimers **6** (**Figures S92-S93**) & **13** (**Figures S94-S95**), and trimer **8**, (**Figures S96-S97**) and comparing the difference between the solid and solution-phase IR, overall, the spectra are very similar, suggesting there is minimal conformational difference between the two phases. For example, the solid-state spectrum of monomer **4** contains sharp peaks at 1710, 1670, and 1640 cm^{-1}, likely arising from the three amide carbonyl groups present within the molecule. The solution state spectrum displays a slightly red-shifted peak at 1702 cm^{-1}, and a broad peak at 1653 cm^{-1}, likely corresponding to the two lower wavenumber absorptions displayed in the solid phase. The same broadening is observed for the solution *vs*. solid-phase spectra for all the compounds investigated and may suggest rotation about the pyridine-pyrrolidinone bond, which would be expected in solution due to greater conformation freedom. However, the small degree of change is consistent with the NMR and XRD data, which indicate that the solution and solid-phase conformations are similar.

7.1.1 IR of Monomer 4

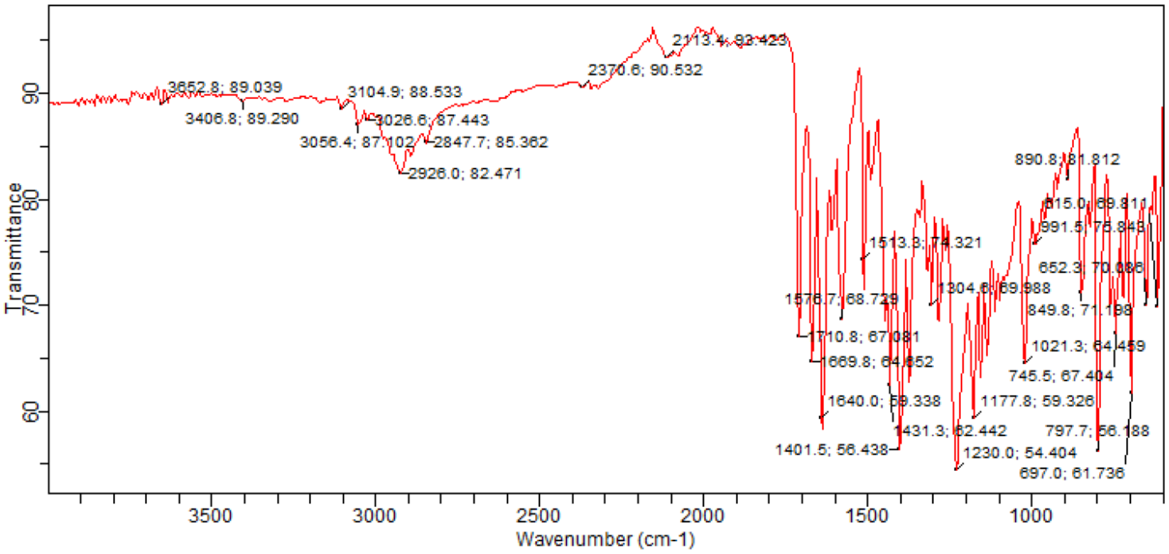

Figure S90: Solid-phase IR of monomer **4**

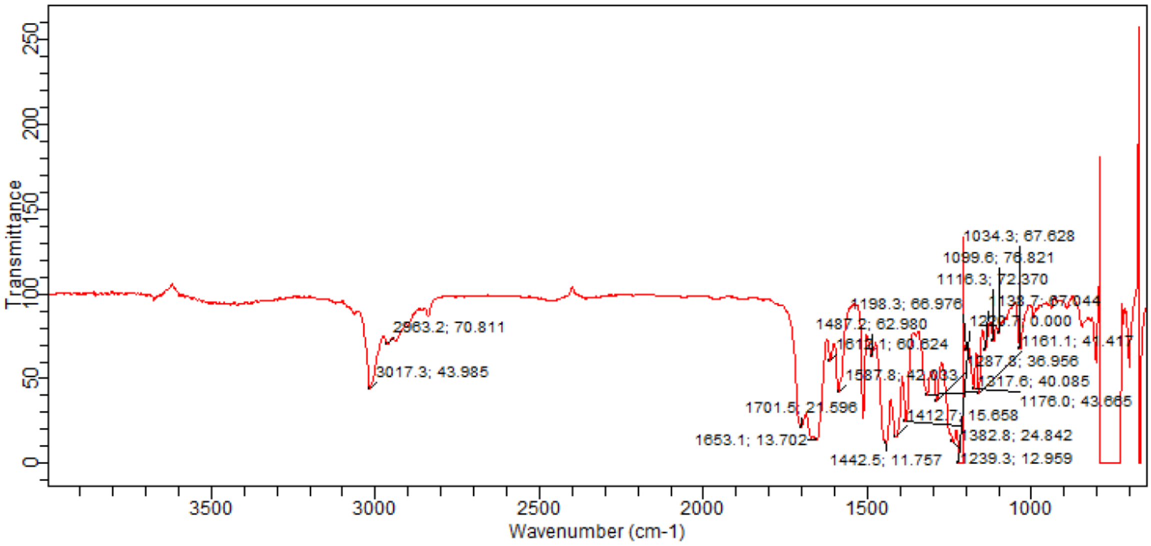

Figure S91: Solution-phase IR of monomer **4**

7.1.2 IR of Dimer 6

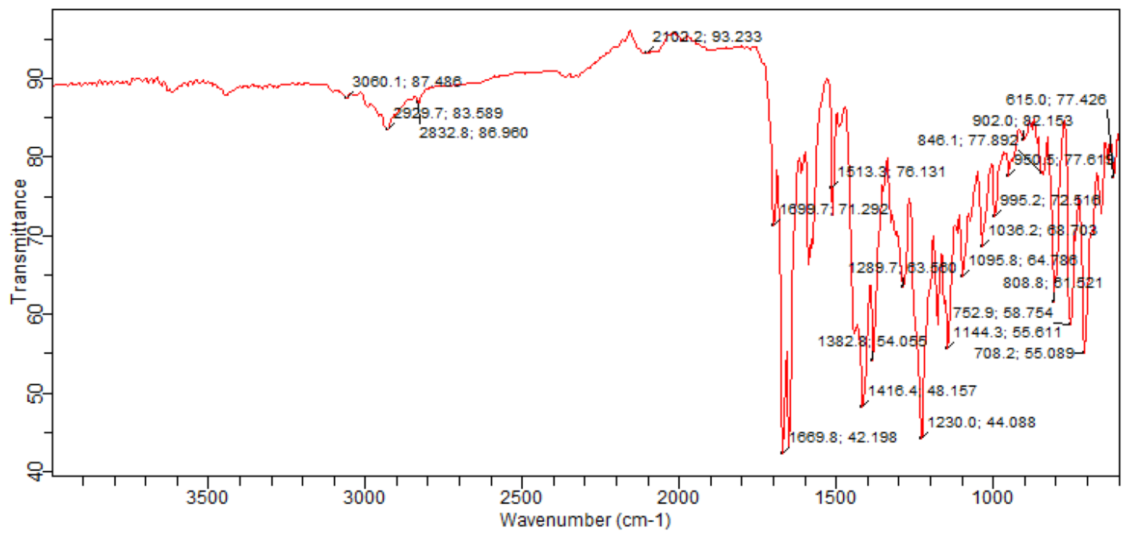

Figure S92: Solid-phase IR of dimer **6**

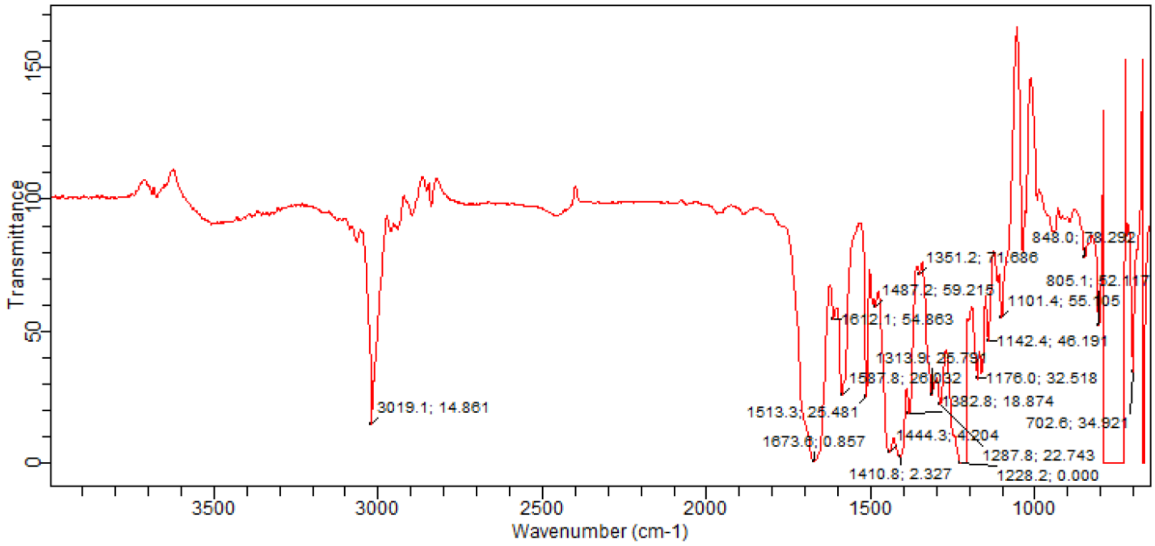

Figure S93: Solution-phase IR of dimer **6**

7.1.3 IR of Dimer 13

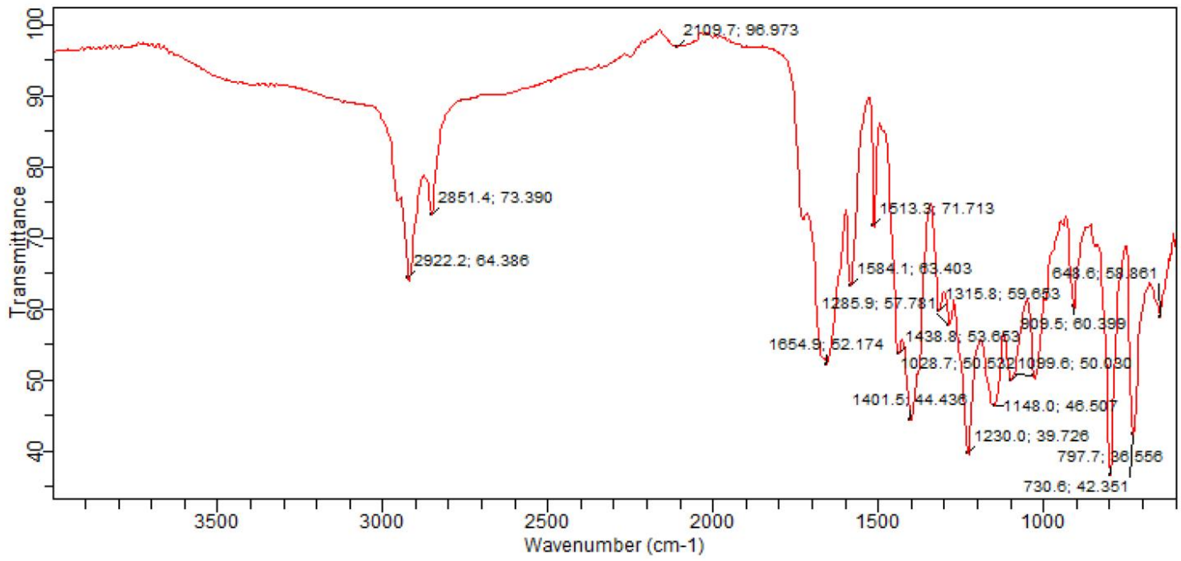

Figure S94: Solid-phase IR of dimer **13**

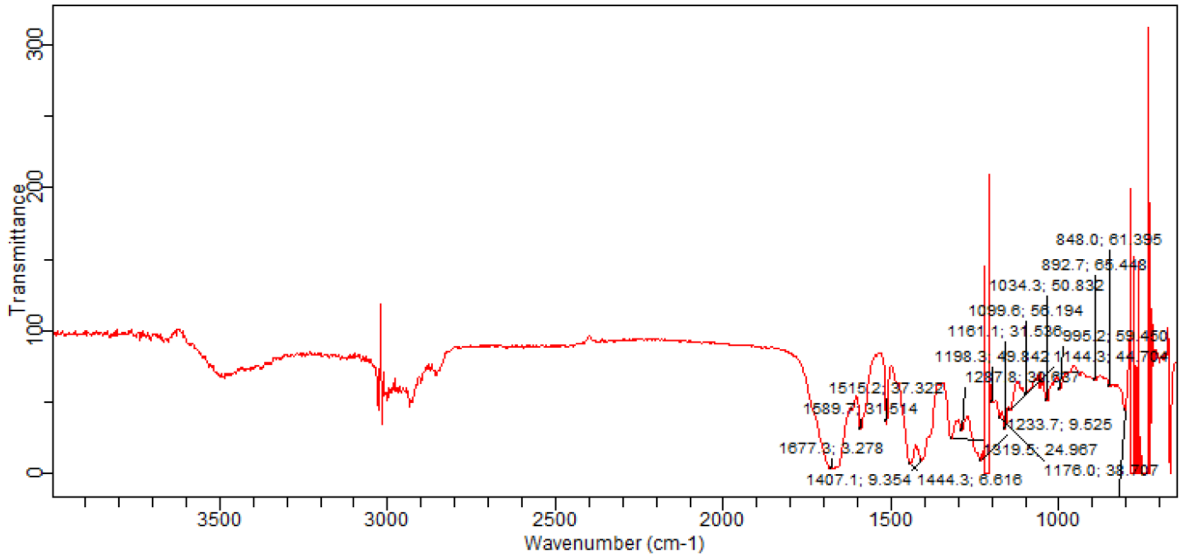

Figure S95: Solution-phase IR of dimer **13**

7.1.4 IR of trimer 8

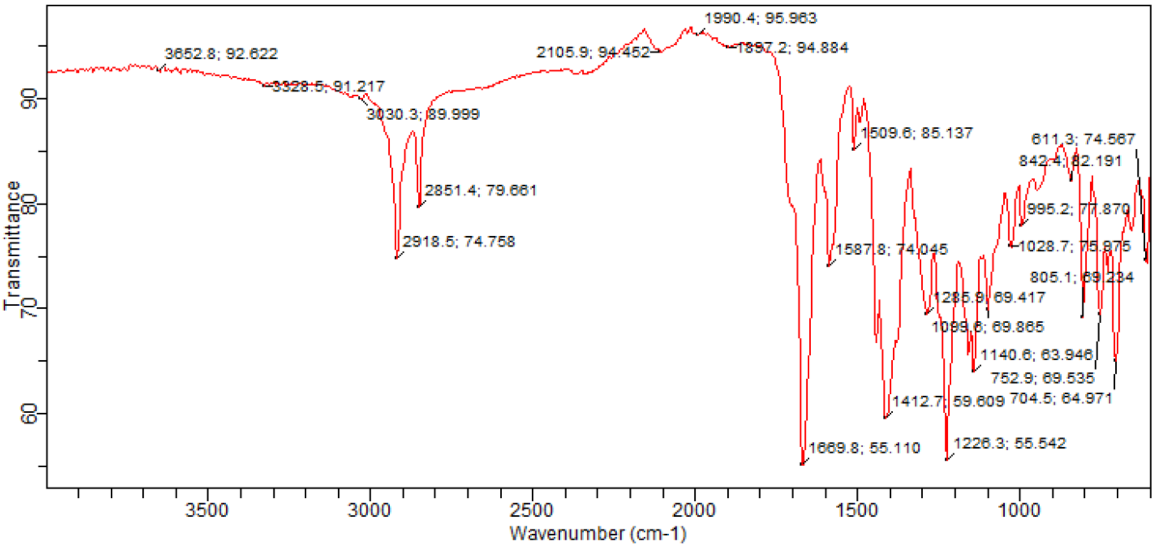

Figure S96: Solid-phase IR of trimer **8**

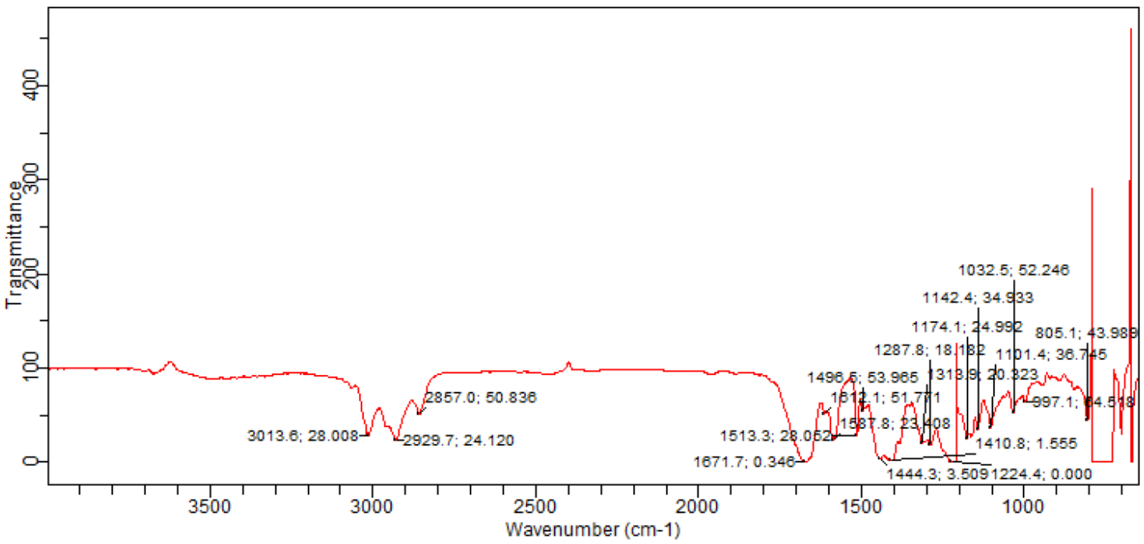

Figure S97: Solution-phase IR of trimer **8**

7.2 Attempts to Obtain IR Spectra of Protonated Foldamers

7.2.1 Solid-Phase

As the protonated dimer and trimer's conformation had only been studied in the solution phase via the use of ROESY spectroscopy, we considered whether IR spectroscopy might provide a means to probe the conformation of the protonated compounds in the solid phase.

To probe the feasibility isolating the fully acid switched conformation, trimer **8** was treated to the identical conditions used in the acid switching experiment i.e., 786 equiv. of TFA and 16 equiv. of TfOH in a 9 mM of $CHCl_3$. The solution was then left for stir for 10 min, and the

solvent evaporated under a stream of compressed air and then concentrated down further in-vacuo. This yielded the trimer **8** as a thick viscous oil.

Running the IR of the oil however yielded an IR spectrum that was very broad and undefined, making a direct comparison difficult, (**Figure S98**) as this broadness may have been a result due to the large excess of TfOH; which due to its high boiling point may not have been fully removed, rather than a conformational change. Because cleanly isolating protonated solid was not a facile without potentially affecting its protonated form, e.g., via extraction or trituration, this therefore meant that the use of FT-IR for probing the protonation status in the solid-phase conformation would be incompatible.

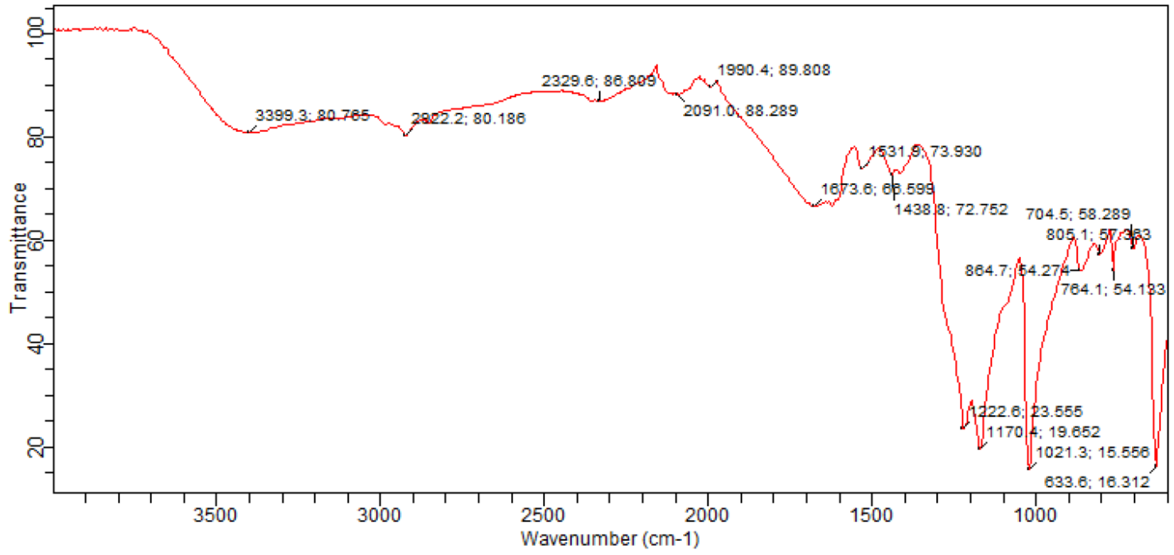

Figure S98: Attempted acquisition of solid-phase IR of protonated trimer **8**

7.2.2 Solution-Phase

With the solid-phase IR proving problematic, attempts to probe the difference in the solution-phase spectra of the neutral and protonated forms were made. However, acquiring the solution-phase IR under the standard protonation condition would interfere with the IR-spectrum due to the super excess of TFA used to protonate the oligomers, saturating the IR spectrum. Attempts were made to overcome this issue by examining if adding 2 equiv. of TfOH to dimer **6** in $CDCl_3$ (9 mM) would enable a the fully protonated conformation to be obtained, thereby mitigating the interference of a large excess of TFA and TfOH the IR-spec. However, upon addition of 2 equiv. of TfOH to dimer **6**, a solid crashed out of solution, indicating that in neat $CDCl_3$ the protonated species is not soluble, suggesting that in the acid-switching experiments, the super excess of *d*-TFA was aiding in dissolution of the protonated species in $CDCl_3$.

As the use of a more polar solvent (d_6-DMSO) has shown to inhibit the ability to form the protonated species, this therefore meant that the use of solution phase IR to probe conformational difference between the neutral and protonated species was also unsuitable.

8 X-ray Crystallography

8.1 Single Crystal Data for 4 (CCDC 2258873)

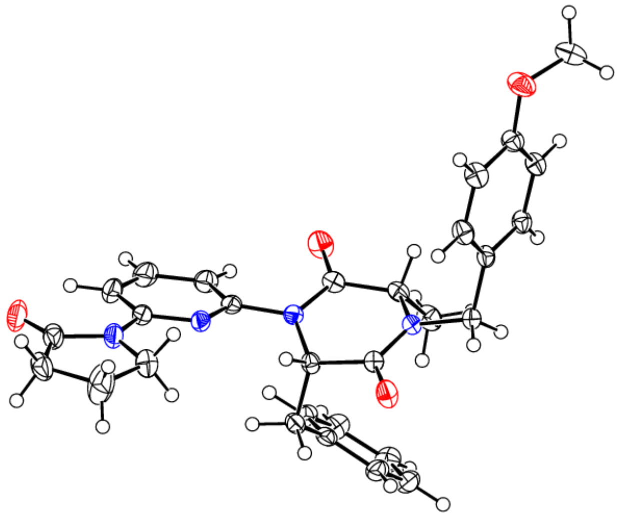

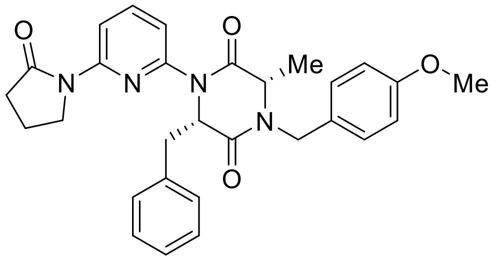

Crystal data	
Chemical formula	$C_{29}H_{30}N_4O_4$
M_r	498.57
Crystal system, space group	Orthorhombic, $P2_12_12_1$
Temperature (K)	100
a, *b*, *c* (Å)	11.72840 (1), 12.776599 (14), 16.331699 (14)
V (Å^3)	2447.29 (1)
Z	4
Radiation type	Cu $K\alpha$
μ (mm^{-1})	0.74
Crystal size (mm)	0.14 × 0.12 × 0.12
Data collection	
Diffractometer	Oxford Diffraction SuperNova
Absorption correction	Multi-scan<br>*CrysAlis PRO* (Rigaku Oxford Diffraction, 2017)
T_{min}, T_{max}	0.80, 1.00
No. of measured, independent and observed [$I > 2.0\sigma(I)$] reflections	22838, 4731, 4614
R_{int}	0.000
$(\sin \theta/\lambda)_{max}$ (Å^{-1})	0.615
Refinement	
$R[F^2 > 2\sigma(F^2)]$, $wR(F^2)$, S	0.035, 0.091, 0.99
No. of reflections	4731
No. of parameters	335
H-atom treatment	H-atom parameters constrained

$\Delta\rho_{max}$, $\Delta\rho_{min}$ (e Å^{-3})	0.37, −0.36
Absolute structure	Parsons, Flack & Wagner (2013), 2005 Friedel Pairs
Absolute structure parameter	−0.03 (8)

Computer programs: SuperNova, (Oxford Diffraction, 2010), *CrysAlis PRO* (Rigaku Oxford Diffraction, 2017), USER DEFINED *STRUCTURE* SOLUTION, *CRYSTALS* (Betteridge *et al.*, 2003), *CAMERON* (Watkin *et al.*, 1996).

8.2 Single Crystal Data for 6 (CCDC 2258872)

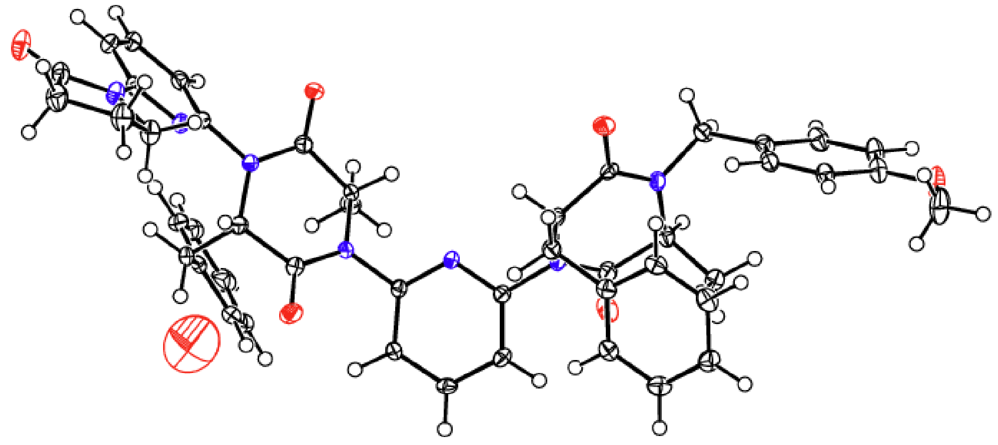

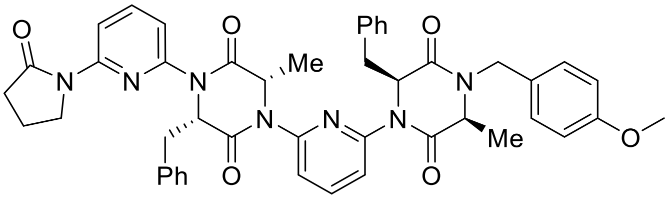

Crystal data	
Chemical formula	$C_{46}H_{45}N_7O_6 \cdot O$
M_r	807.89
Crystal system, space group	Monoclinic, $P2_1$
Temperature (K)	100
a, *b*, *c* (Å)	7.86, 34.34, 8.27
β (°)	118.049 (4)
V ($Å^3$)	1970.00 (7)
Z	2
Radiation type	Cu *K*α
μ (mm^{-1})	0.76
Crystal size (mm)	0.16 × 0.12 × 0.10
Data collection	
Diffractometer	Oxford Diffraction SuperNova
Absorption correction	Multi-scan *CrysAlis PRO* (Rigaku Oxford Diffraction, 2017)
T_{min}, T_{max}	0.76, 0.93
No. of measured, independent and observed [$I > 2.0\sigma(I)$] reflections	18457, 7287, 7149
R_{int}	0.000
$(\sin \theta/\lambda)_{max}$ ($Å^{-1}$)	0.616
Refinement	
$R[F^2 > 2\sigma(F^2)]$, $wR(F^2)$, S	0.047, 0.117, 1.01
No. of reflections	7287

No. of parameters	542
No. of restraints	1
H-atom treatment	H-atom parameters constrained
$\Delta\rho_{max}$, $\Delta\rho_{min}$ (e Å^{-3})	0.90, −1.05
Absolute structure	Parsons, Flack & Wagner (2013), 3321 Friedel Pairs
Absolute structure parameter	0.15 (9)

Computer programs: SuperNova, (Oxford Diffraction, 2010), *CrysAlis PRO* (Rigaku Oxford Diffraction, 2017), USER DEFINED *STRUCTURE* SOLUTION, *CRYSTALS* (Betteridge *et al.*, 2003), *CAMERON* (Watkin *et al.*, 1996).

9 NMR Spectra

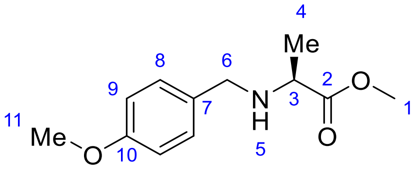

S1

^{1}H NMR

600 MHz

$CDCl_3$

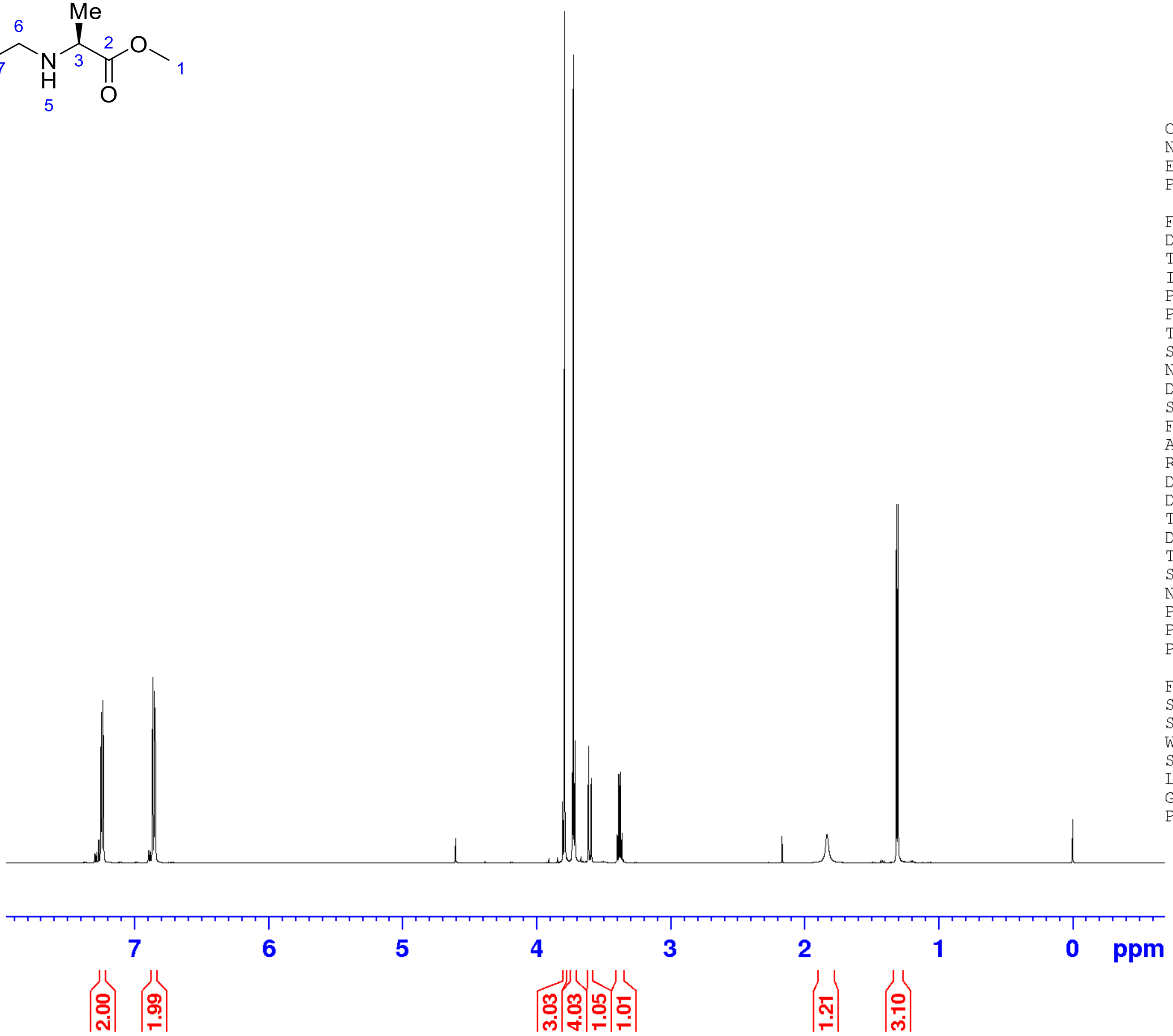


```
Current Data Parameters
NAME              SMC-01-29
EXPNO                    10
PROCNO                    1

F2 - Acquisition Parameters
Date_              20220202
Time                  23.27 h
INSTRUM               spect
PROBHD    Z114607_0188 (
PULPROG                zg30
TD                    65536
SOLVENT               CDCl3
NS                       16
DS                        2
SWH               12019.230 Hz
FIDRES             0.366798 Hz
AQ                2.7262976 sec
RG                    49.63
DW                   41.600 usec
DE                    12.10 usec
TE                    294.5 K
D1               1.00000000 sec
TD0                       1
SFO1            600.1337058 MHz
NUC1                     1H
P0                     3.33 usec
P1                    10.00 usec
PLW1            26.60000038 W

F2 - Processing parameters
SI                    65536
SF              600.1300117 MHz
WDW                      EM
SSB                       0
LB                     0.30 Hz
GB                        0
PC                     1.00
```

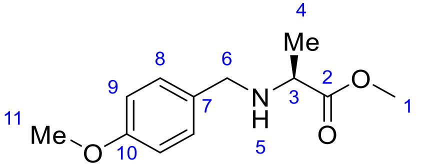

S1

^{13}C NMR

151 MHz

$CDCl_3$

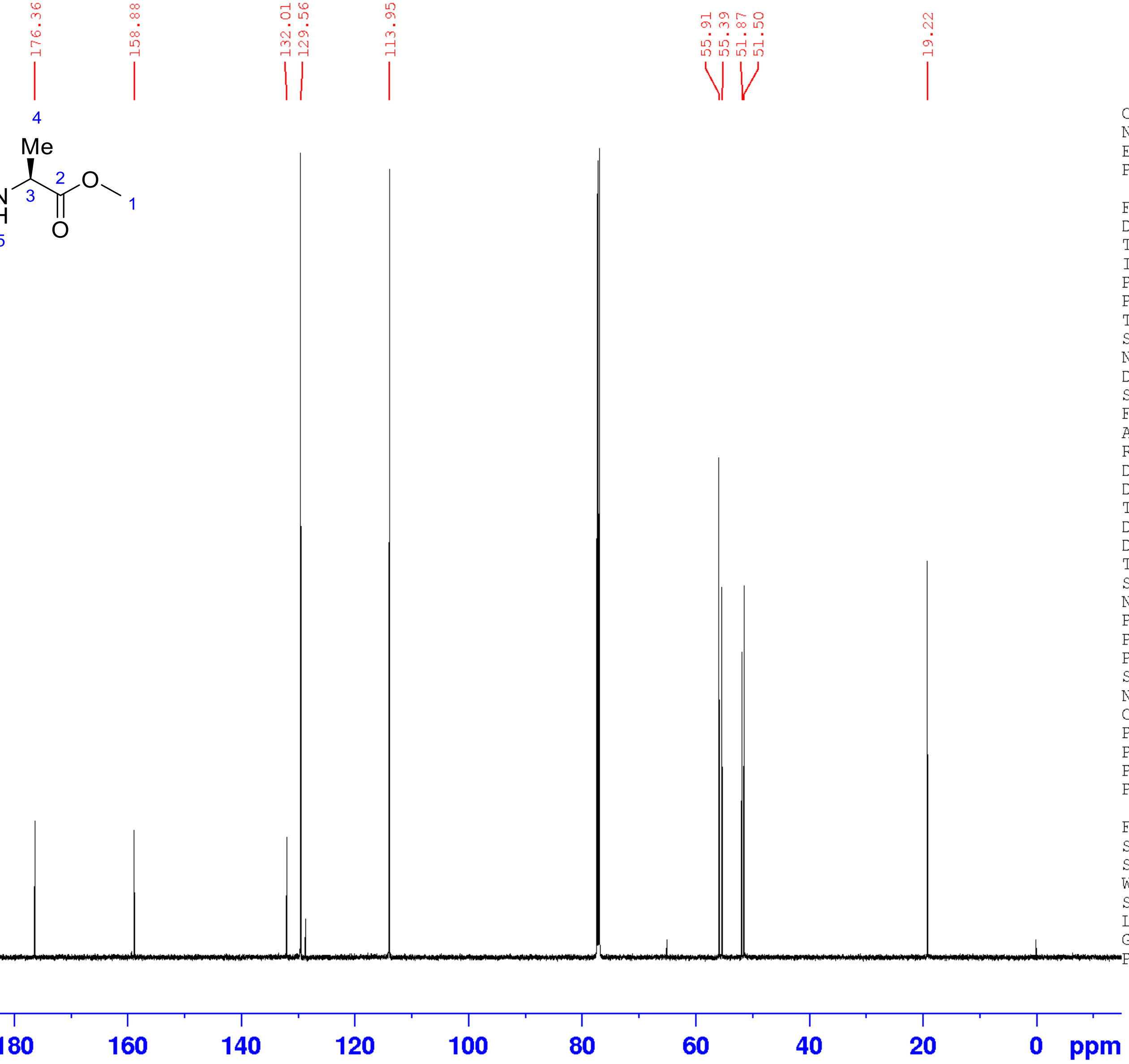


```
Current Data Parameters
NAME              SMC-01-29
EXPNO                    11
PROCNO                    1

F2 - Acquisition Parameters
Date_              20220203
Time                 0.19 h
INSTRUM               spect
PROBHD     Z114607_0188 (
PULPROG             zgpg30
TD                   65536
SOLVENT              CDCl3
NS                    1024
DS                       4
SWH             36231.883 Hz
FIDRES           1.105709 Hz
AQ              0.9043968 sec
RG                  186.92
DW                  13.800 usec
DE                    6.50 usec
TE                   296.4 K
D1             2.00000000 sec
D11            0.03000000 sec
TD0                      1
SFO1           150.9178988 MHz
NUC1                   13C
P0                    3.93 usec
P1                   11.80 usec
PLW1           85.00000000 W
SFO2           600.1324005 MHz
NUC2                    1H
CPDPRG[2           waltz65
PCPD2                70.00 usec
PLW2           27.00000000 W
PLW12           0.57327998 W
PLW13           0.28836000 W

F2 - Processing parameters
SI                   32768
SF             150.9027904 MHz
WDW                     EM
SSB                      0
LB                    1.00 Hz
GB                       0
PC                    1.40
```

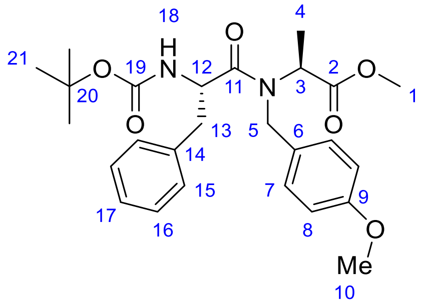

Major rotamer 1a

^{1}H NMR

600 MHz

$CDCl_3$

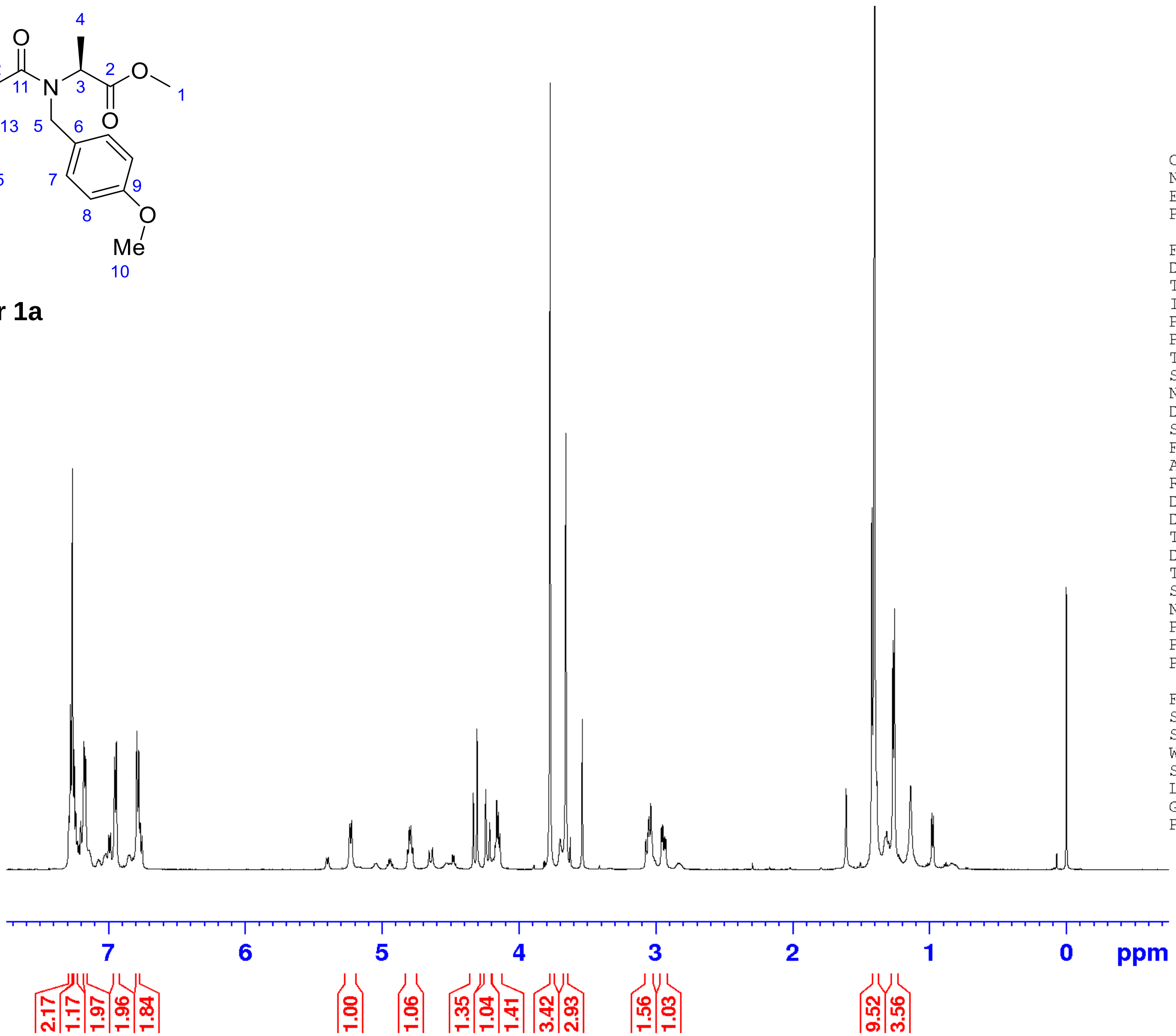


```
Current Data Parameters
NAME       SMC-01-30-600
EXPNO                 10
PROCNO                 1

F2 - Acquisition Parameters
Date_           20220225
Time                0.45 h
INSTRUM            spect
PROBHD    Z114607_0188 (
PULPROG             zg30
TD                 65536
SOLVENT            CDCl3
NS                    16
DS                     2
SWH            12019.230 Hz
FIDRES          0.366798 Hz
AQ             2.7262976 sec
RG                 74.91
DW                41.600 usec
DE                 12.10 usec
TE                 300.0 K
D1            1.00000000 sec
TD0                    1
SFO1         600.1337058 MHz
NUC1                  1H
P0                  3.33 usec
P1                 10.00 usec
PLW1         26.60000038 W

F2 - Processing parameters
SI                 65536
SF           600.1300141 MHz
WDW                   EM
SSB                    0
LB                  0.30 Hz
GB                     0
PC                  1.00
```

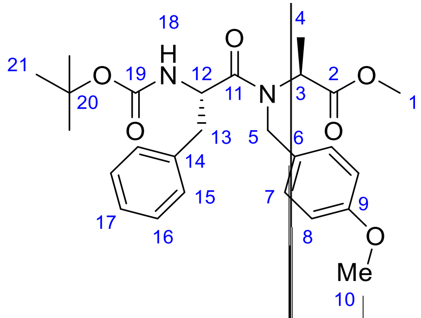

Minor rotamer 1a

^{1}H NMR

600 MHz

$CDCl_3$

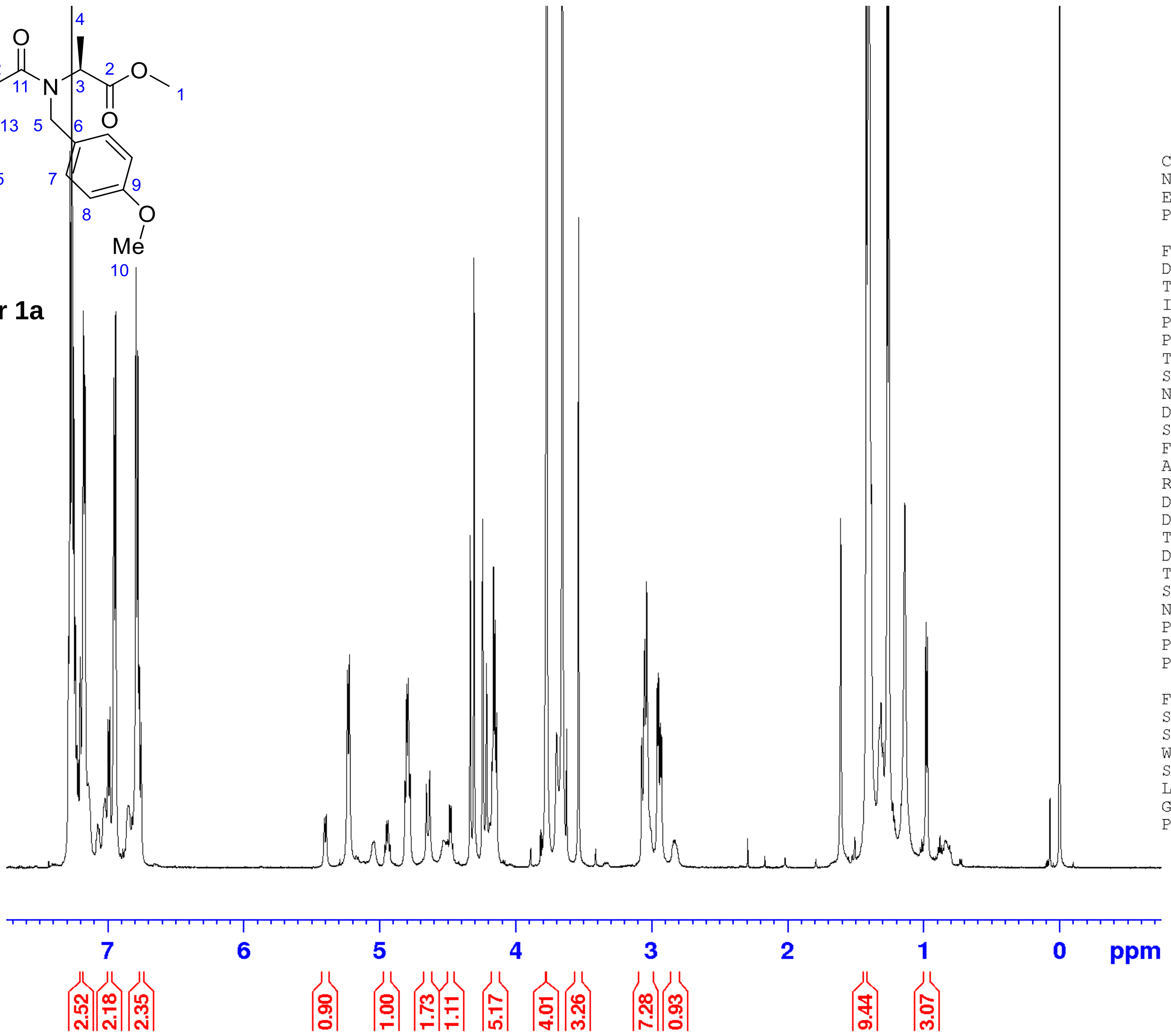


```
Current Data Parameters
NAME          SMC-01-30-600
EXPNO                    20
PROCNO                    1

F2 - Acquisition Parameters
Date_              20220225
Time                 0.45 h
INSTRUM               spect
PROBHD     Z114607_0188 (
PULPROG                zg30
TD                    65536
SOLVENT               CDCl3
NS                       16
DS                        2
SWH           12019.230 Hz
FIDRES         0.366798 Hz
AQ            2.7262976 sec
RG                    74.91
DW               41.600 usec
DE                12.10 usec
TE                300.0 K
D1           1.00000000 sec
TD0                       1
SFO1        600.1337058 MHz
NUC1                     1H
P0                 3.33 usec
P1                10.00 usec
PLW1        26.60000038 W

F2 - Processing parameters
SI                    65536
SF          600.1300141 MHz
WDW                      EM
SSB                       0
LB                   0.30 Hz
GB                        0
PC                     1.00
```

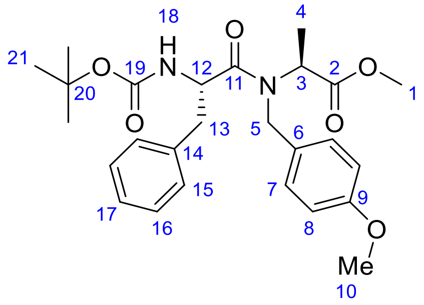

Major rotamer 1a

^{13}C NMR

151 MHz

$CDCl_3$

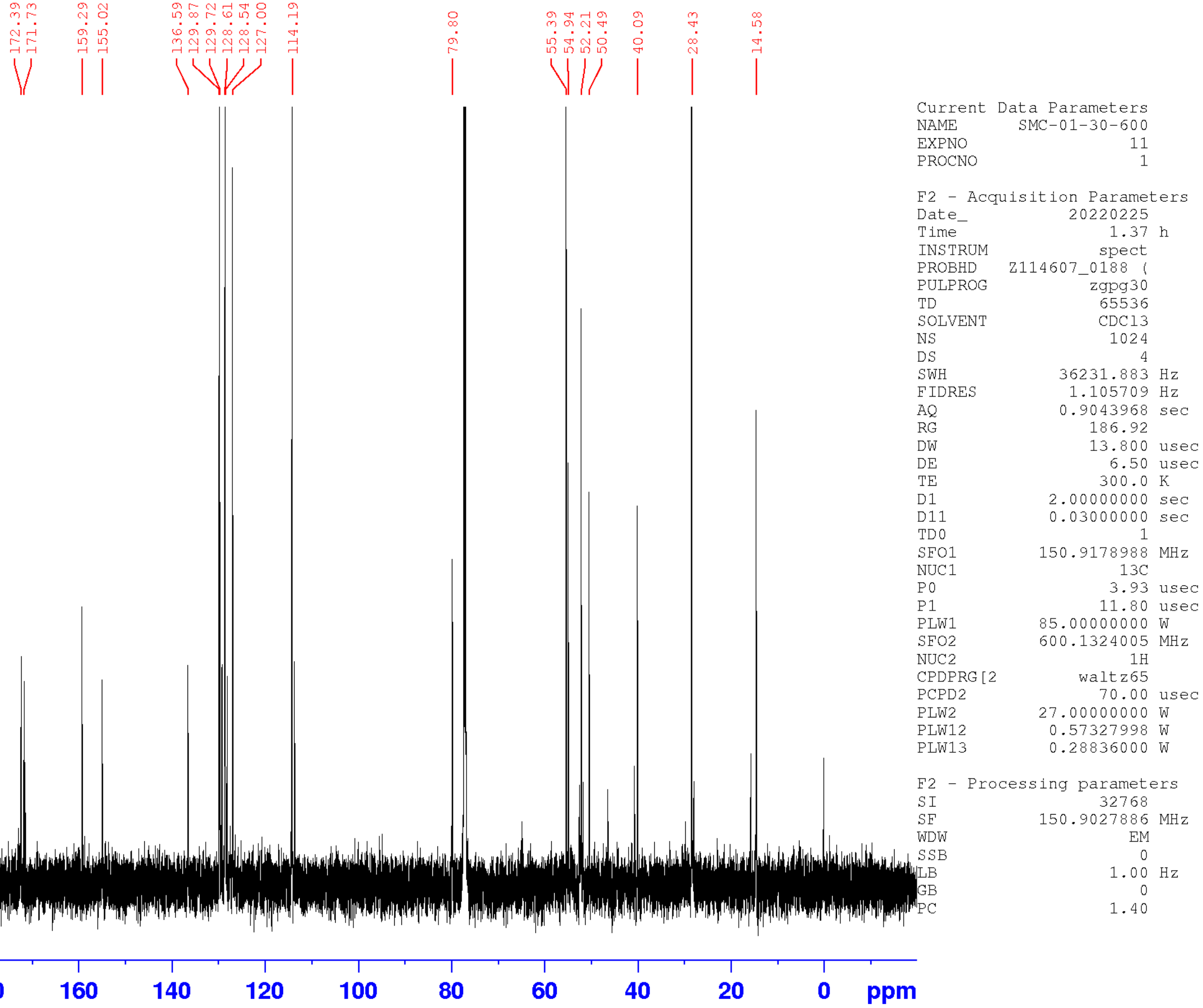

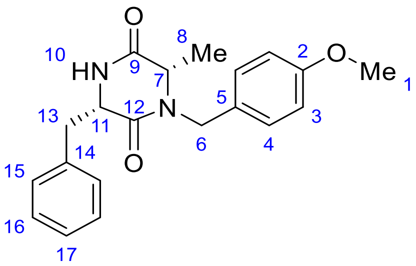

2a

^{1}H NMR

600 MHz

$CDCl_3$

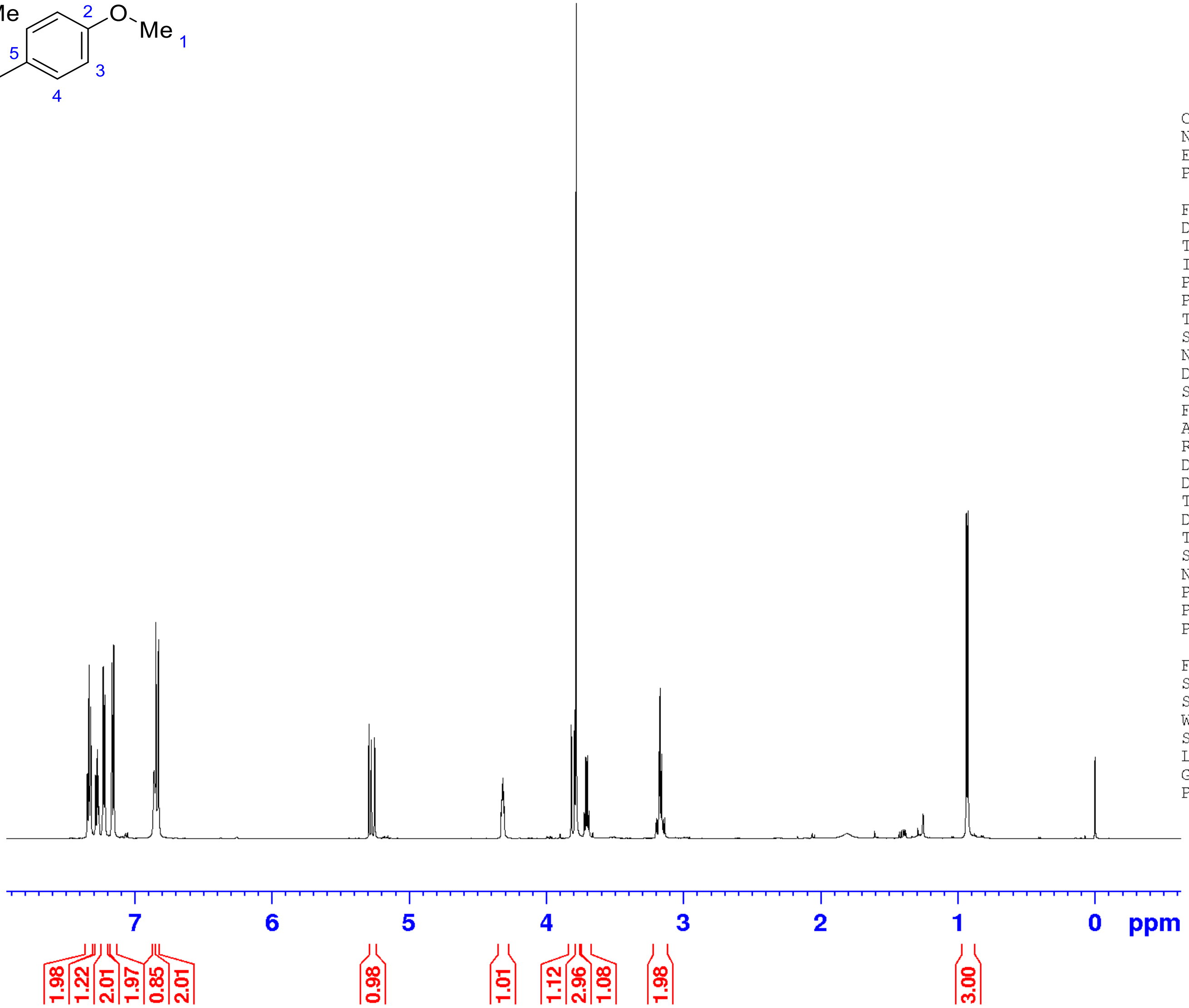


```
Current Data Parameters
NAME              SMC-01-32
EXPNO                    10
PROCNO                    1

F2 - Acquisition Parameters
Date_              20220203
Time                   1.46 h
INSTRUM               spect
PROBHD    Z114607_0188 (
PULPROG                zg30
TD                    65536
SOLVENT               CDCl3
NS                       16
DS                        2
SWH               12019.230 Hz
FIDRES             0.366798 Hz
AQ                2.7262976 sec
RG                    60.48
DW                   41.600 usec
DE                    12.10 usec
TE                    294.6 K
D1               1.00000000 sec
TD0                       1
SFO1            600.1337058 MHz
NUC1                     1H
P0                     3.33 usec
P1                    10.00 usec
PLW1            26.60000038 W

F2 - Processing parameters
SI                    65536
SF              600.1300114 MHz
WDW                      EM
SSB                       0
LB                     0.30 Hz
GB                        0
PC                     1.00
```

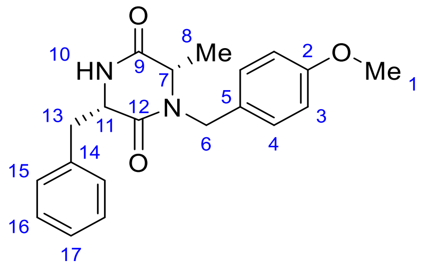

2a

^{13}C NMR

151 MHz

$CDCl_3$

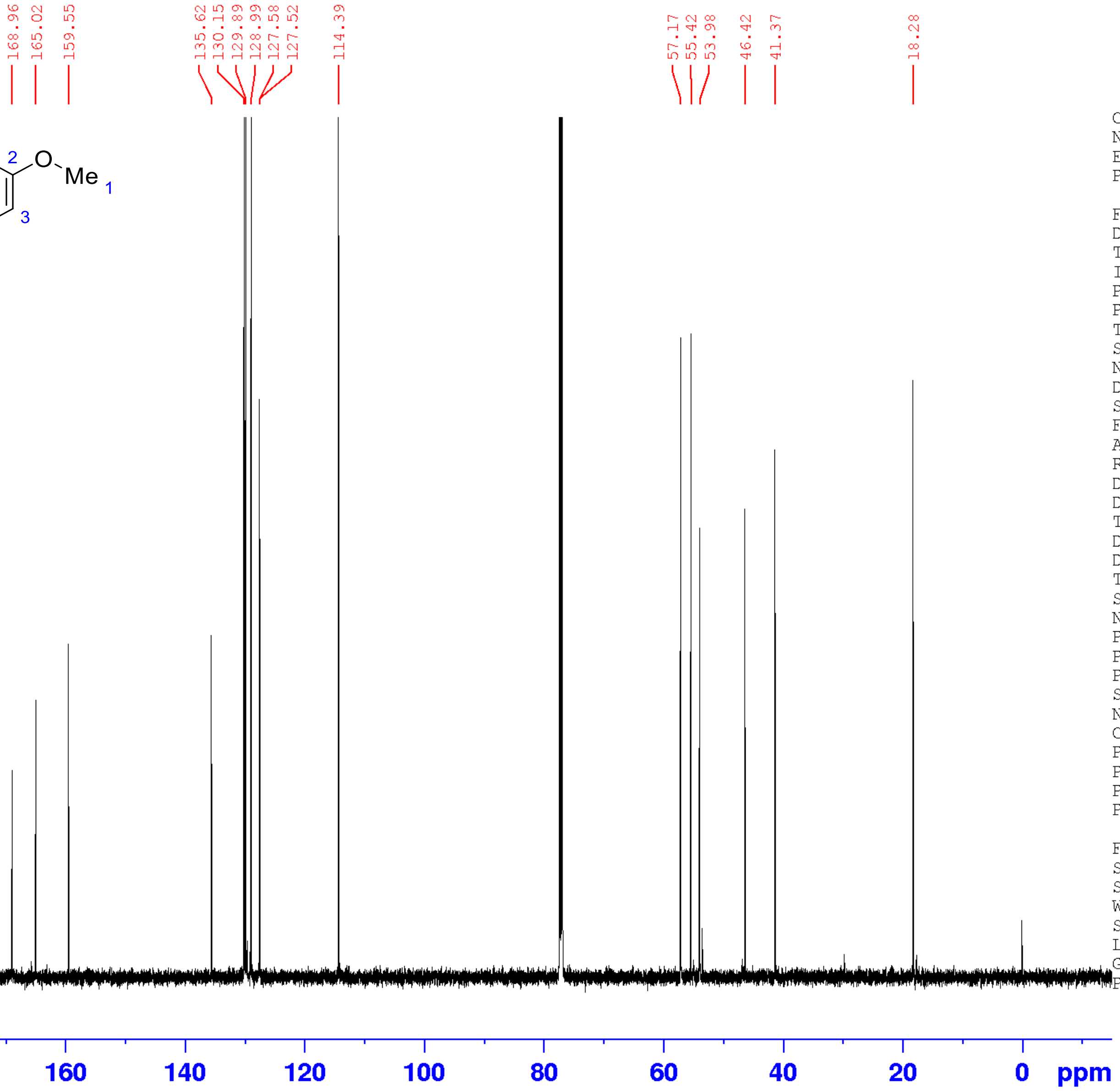


```
Current Data Parameters
NAME              SMC-01-32
EXPNO                    11
PROCNO                    1

F2 - Acquisition Parameters
Date_              20220203
Time                   2.37 h
INSTRUM               spect
PROBHD     Z114607_0188 (
PULPROG              zgpg30
TD                    65536
SOLVENT               CDCl3
NS                     1024
DS                        4
SWH               36231.883 Hz
FIDRES             1.105709 Hz
AQ                0.9043968 sec
RG                   186.92
DW                   13.800 usec
DE                     6.50 usec
TE                    296.4 K
D1               2.00000000 sec
D11              0.03000000 sec
TD0                       1
SFO1            150.9178988 MHz
NUC1                    13C
P0                     3.93 usec
P1                    11.80 usec
PLW1            85.00000000 W
SFO2            600.1324005 MHz
NUC2                     1H
CPDPRG[2            waltz65
PCPD2                 70.00 usec
PLW2            27.00000000 W
PLW12            0.57327998 W
PLW13            0.28836000 W

F2 - Processing parameters
SI                    32768
SF              150.9027914 MHz
WDW                      EM
SSB                       0
LB                     1.00 Hz
GB                        0
PC                     1.40
```

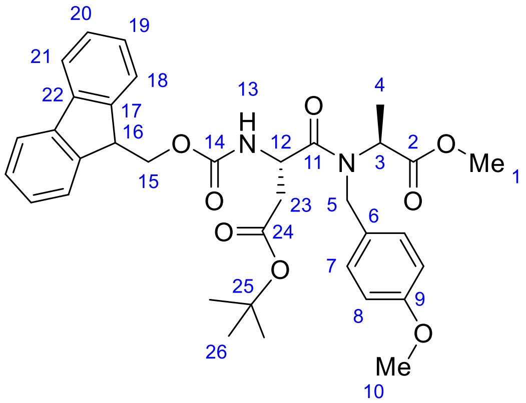

Major rotamer 1b

^{1}H NMR

600 MHz

$CDCl_3$

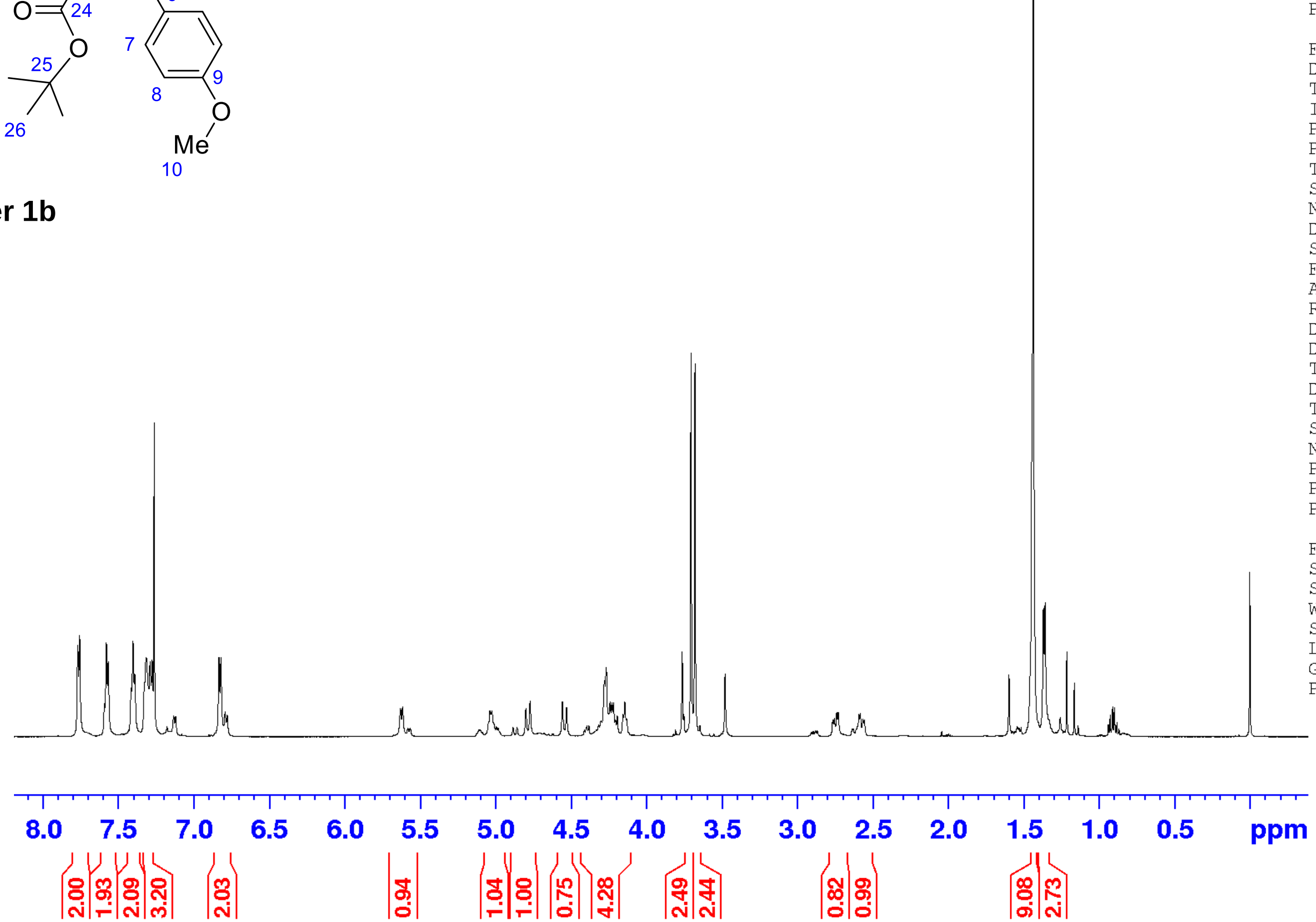


```
Current Data Parameters
NAME       SMC-01-42-600
EXPNO                 10
PROCNO                 1

F2 - Acquisition Parameters
Date_           20220228
Time               14.49 h
INSTRUM            spect
PROBHD    Z114607_0188 (
PULPROG             zg30
TD                 65536
SOLVENT            CDCl3
NS                    16
DS                     2
SWH            12019.230 Hz
FIDRES          0.366798 Hz
AQ             2.7262976 sec
RG                 83.95
DW                41.600 usec
DE                 12.10 usec
TE                 300.0 K
D1            1.00000000 sec
TD0                    1
SFO1         600.1337058 MHz
NUC1                  1H
P0                  3.33 usec
P1                 10.00 usec
PLW1         26.60000038 W

F2 - Processing parameters
SI                 65536
SF           600.1300144 MHz
WDW                   EM
SSB                    0
LB                  0.30 Hz
GB                     0
PC                  1.00
```

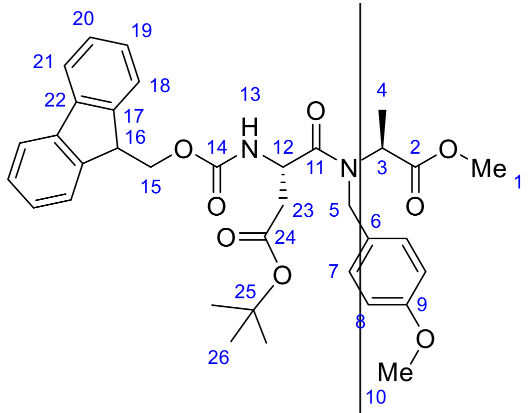

Minor rotamer 1b

^{1}H NMR

600 MHz

$CDCl_3$

Over-integration is a result of co-incidence with the major rotamer

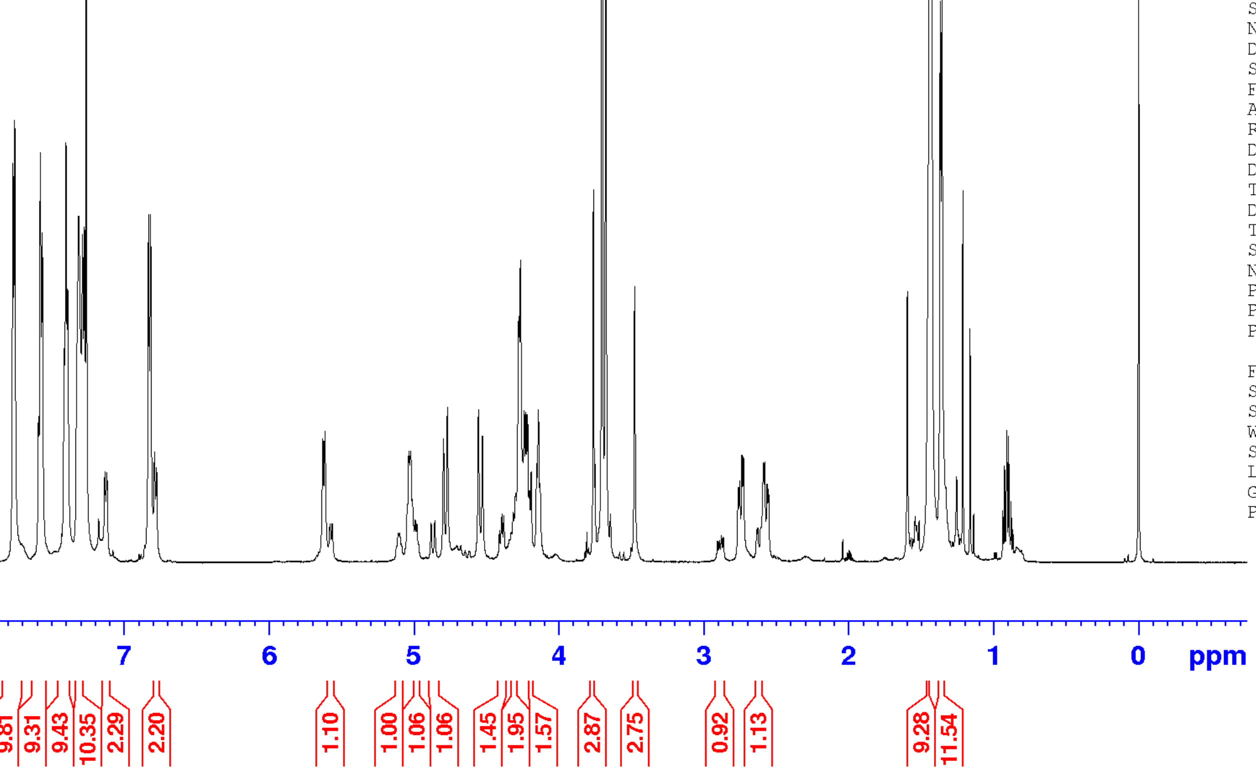


```
Current Data Parameters
NAME        SMC-01-42-600
EXPNO                  20
PROCNO                  1

F2 - Acquisition Parameters
Date_            20220228
Time                14.49 h
INSTRUM             spect
PROBHD    Z114607_0188 (
PULPROG              zg30
TD                  65536
SOLVENT             CDCl3
NS                     16
DS                      2
SWH             12019.230 Hz
FIDRES           0.366798 Hz
AQ              2.7262976 sec
RG                  83.95
DW                 41.600 usec
DE                  12.10 usec
TE                  300.0 K
D1             1.00000000 sec
TD0                     1
SFO1          600.1337058 MHz
NUC1                   1H
P0                   3.33 usec
P1                  10.00 usec
PLW1          26.60000038 W

F2 - Processing parameters
SI                  65536
SF            600.1300158 MHz
WDW                    EM
SSB                     0
LB                   0.30 Hz
GB                      0
PC                   1.00
```

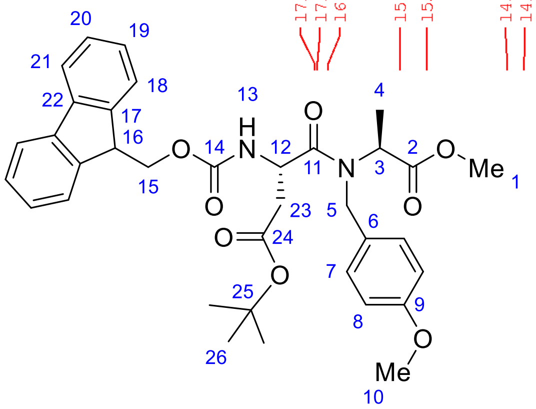

Major rotamer 1b

^{13}C NMR

151 MHz

$CDCl_3$

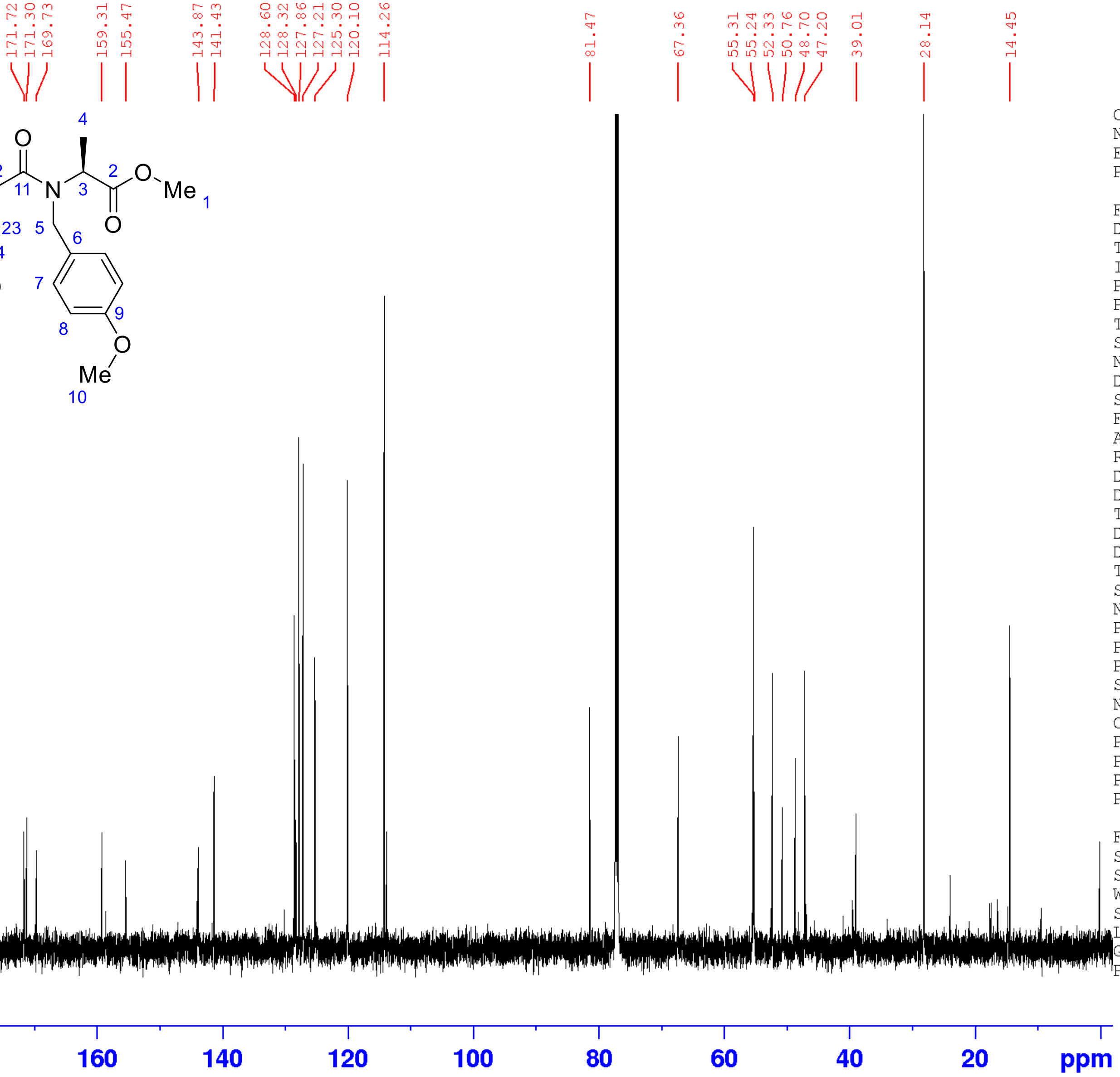

Current Data Parameters
NAME SMC-01-42-600
EXPNO 11
PROCNO 1

F2 - Acquisition Parameters
Date_ 20220228
Time 15.40 h
INSTRUM spect
PROBHD Z114607_0188 (
PULPROG zgpg30
TD 65536
SOLVENT CDCl3
NS 1024
DS 4
SWH 36231.883 Hz
FIDRES 1.105709 Hz
AQ 0.9043968 sec
RG 186.92
DW 13.800 usec
DE 6.50 usec
TE 300.0 K
D1 2.00000000 sec
D11 0.03000000 sec
TD0 1
SFO1 150.9178988 MHz
NUC1 13C
P0 3.93 usec
P1 11.80 usec
PLW1 85.00000000 W
SFO2 600.1324005 MHz
NUC2 1H
CPDPRG[2 waltz65
PCPD2 70.00 usec
PLW2 27.00000000 W
PLW12 0.57327998 W
PLW13 0.28836000 W

F2 - Processing parameters
SI 32768
SF 150.9027886 MHz
WDW EM
SSB 0
LB 1.00 Hz
GB 0
PC 1.40

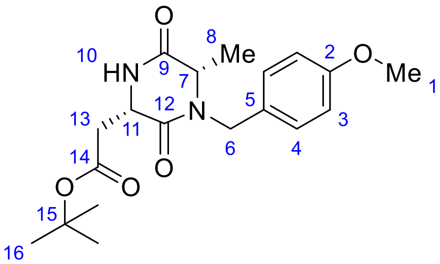

2b

^{1}H NMR

400 MHz

$CDCl_3$

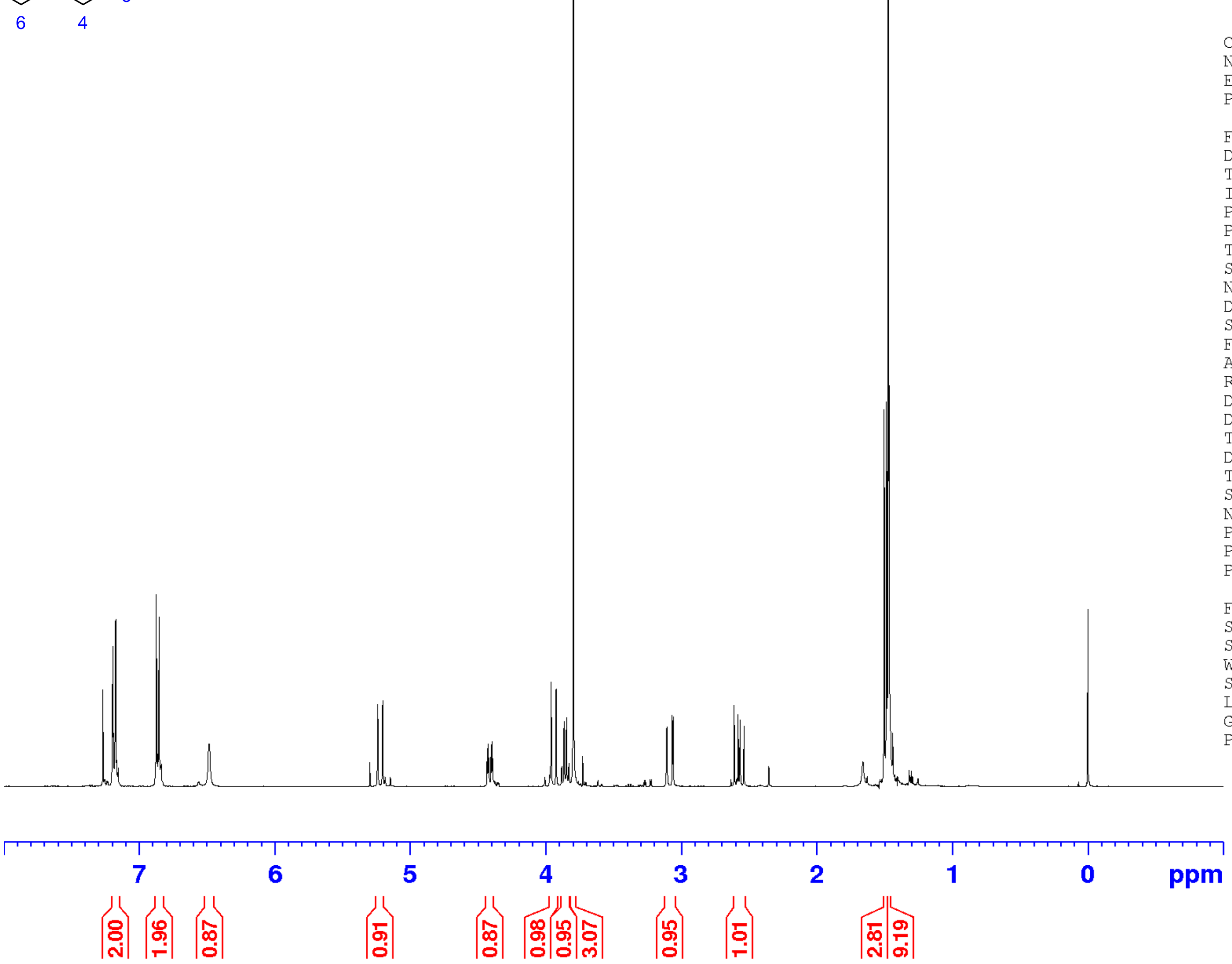


```
Current Data Parameters
NAME          WR 3.363 V2
EXPNO                  10
PROCNO                  1

F2 - Acquisition Parameters
Date_            20230120
Time               18.05 h
INSTRUM          AVIII_400
PROBHD      Z108618_0817 (
PULPROG              zg30
TD                  65536
SOLVENT             CDCl3
NS                     16
DS                      2
SWH             8223.685 Hz
FIDRES          0.250967 Hz
AQ             3.9845889 sec
RG                   50.8
DW                 60.800 usec
DE                   6.50 usec
TE                  295.2 K
D1            1.00000000 sec
TD0                     1
SFO1         399.9124696 MHz
NUC1                   1H
P0                   7.33 usec
P1                  22.00 usec
PLW1          31.62299919 W

F2 - Processing parameters
SI                  32768
SF           399.9100074 MHz
WDW                    EM
SSB                     0
LB                   0.30 Hz
GB                      0
PC                   1.00
```

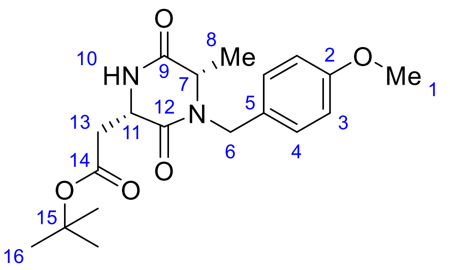

2b

^{13}C NMR

101 MHz

$CDCl_3$

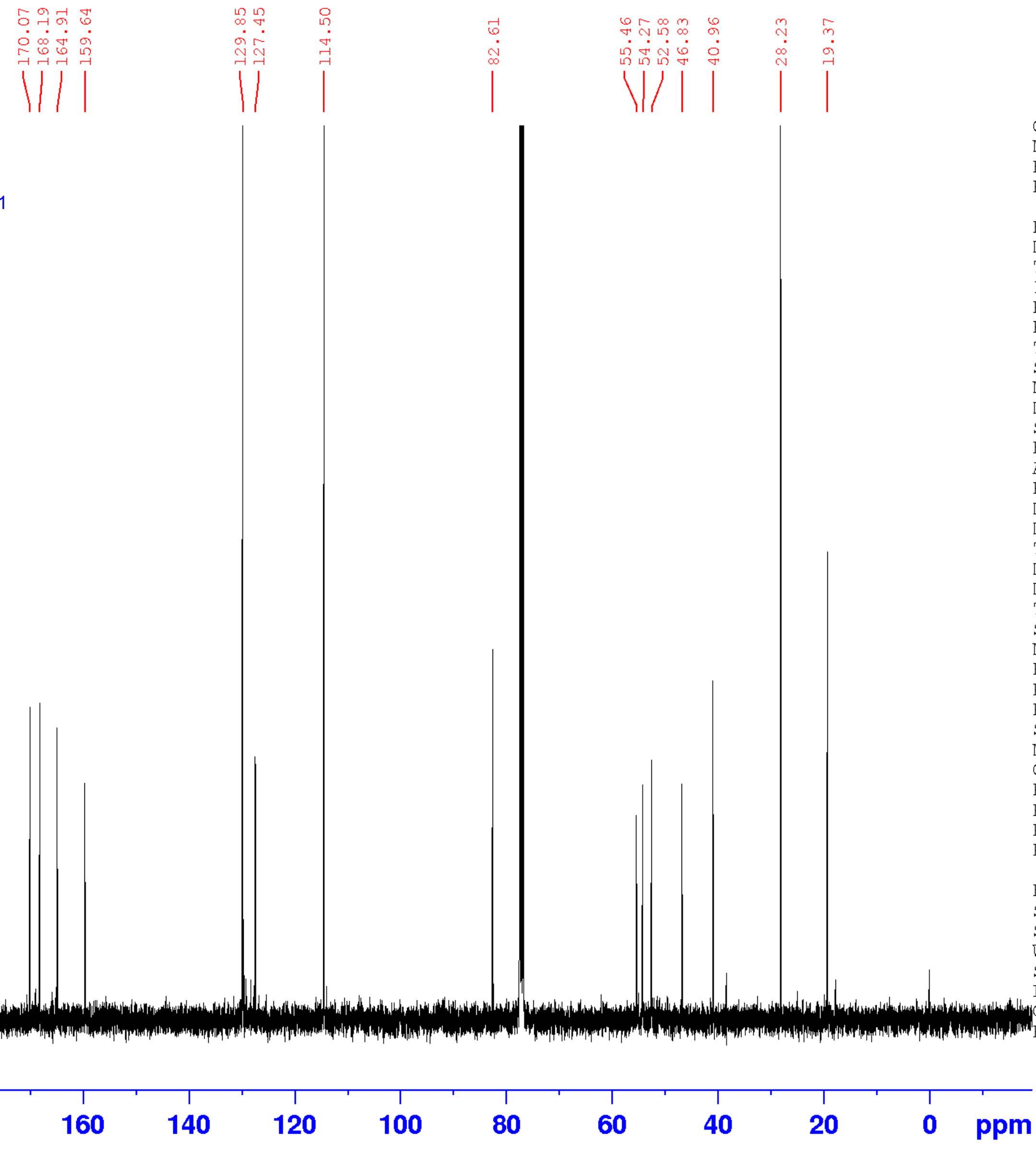

Current Data Parameters
NAME WR 3.363 V2
EXPNO 11
PROCNO 1

F2 - Acquisition Parameters
Date_ 20230121
Time 10.09 h
INSTRUM AVIII_400
PROBHD Z108618_0817 (
PULPROG zgpg30
TD 96150
SOLVENT CDCl3
NS 1024
DS 4
SWH 24038.461 Hz
FIDRES 0.500020 Hz
AQ 1.9999200 sec
RG 144
DW 20.800 usec
DE 6.50 usec
TE 298.9 K
D1 1.00000000 sec
D11 0.03000000 sec
TD0 1
SFO1 100.5675047 MHz
NUC1 13C
P0 1.93 usec
P1 5.80 usec
PLW1 96.68000031 W
SFO2 399.9115996 MHz
NUC2 1H
CPDPRG[2 waltz64
PCPD2 90.00 usec
PLW2 31.62299919 W
PLW12 0.48032999 W
PLW13 0.24160001 W

F2 - Processing parameters
SI 131072
SF 100.5574363 MHz
WDW EM
SSB 0
LB 1.00 Hz
GB 0
PC 1.40

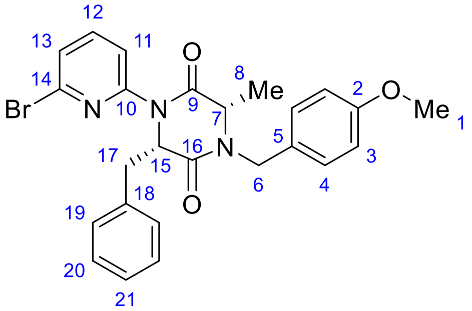

3a

^{1}H NMR

600 MHz

$CDCl_3$

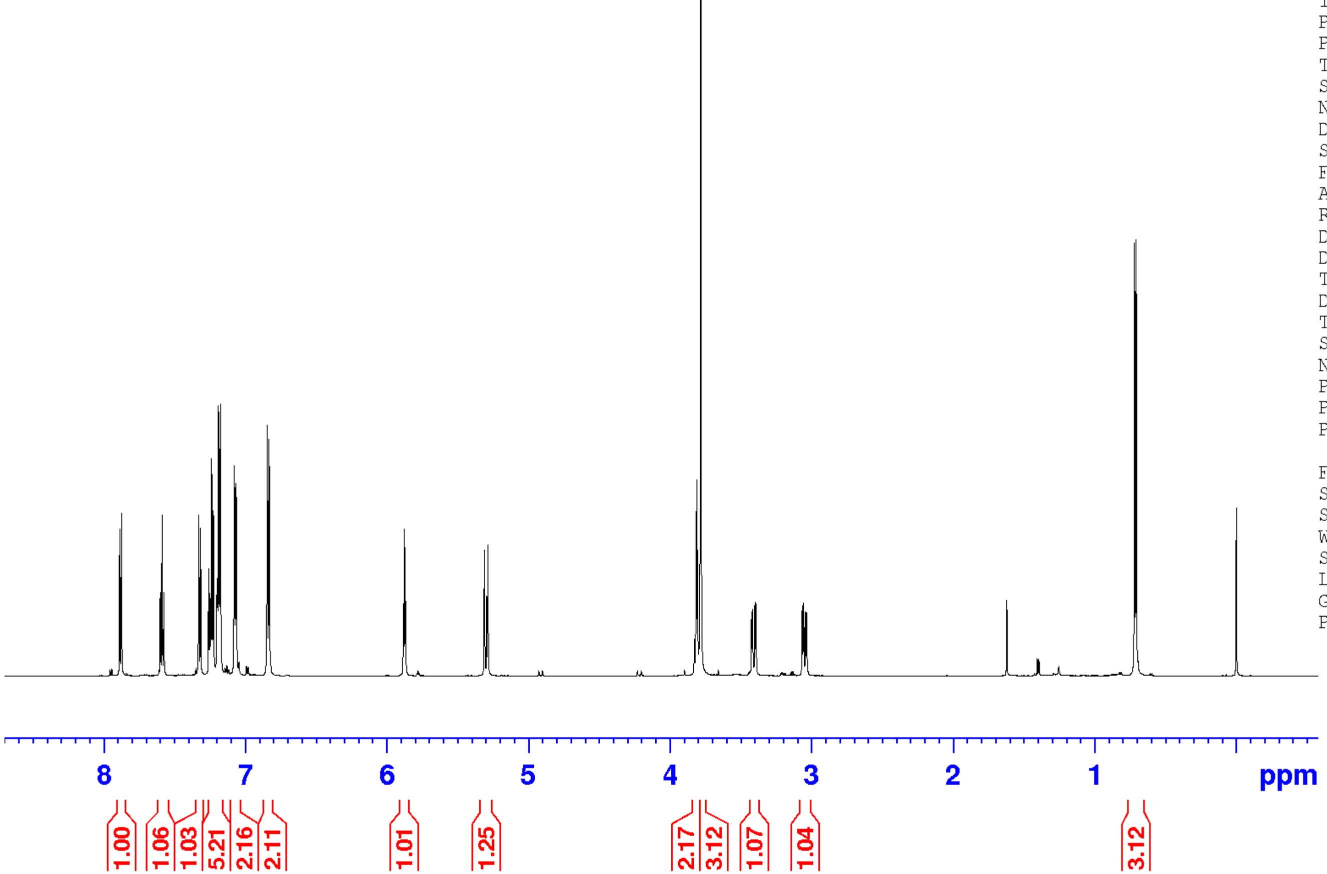


```
Current Data Parameters
NAME      SMC-01-33 (600)
EXPNO                  10
PROCNO                  1

F2 - Acquisition Parameters
Date_            20220203
Time                19.37 h
INSTRUM             spect
PROBHD    Z114607_0188 (
PULPROG              zg30
TD                  65536
SOLVENT             CDCl3
NS                     16
DS                      2
SWH             12019.230 Hz
FIDRES           0.366798 Hz
AQ              2.7262976 sec
RG                  74.91
DW                 41.600 usec
DE                  12.10 usec
TE                  295.0 K
D1             1.00000000 sec
TD0                     1
SFO1         600.1337058 MHz
NUC1                   1H
P0                   3.33 usec
P1                  10.00 usec
PLW1          26.60000038 W

F2 - Processing parameters
SI                  65536
SF           600.1300135 MHz
WDW                    EM
SSB                     0
LB                   0.30 Hz
GB                      0
PC                   1.00
```

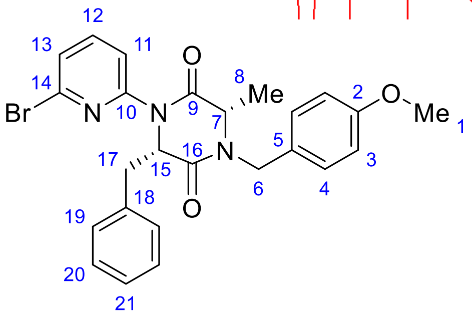

3a

^{13}C NMR

151 MHz

$CDCl_3$

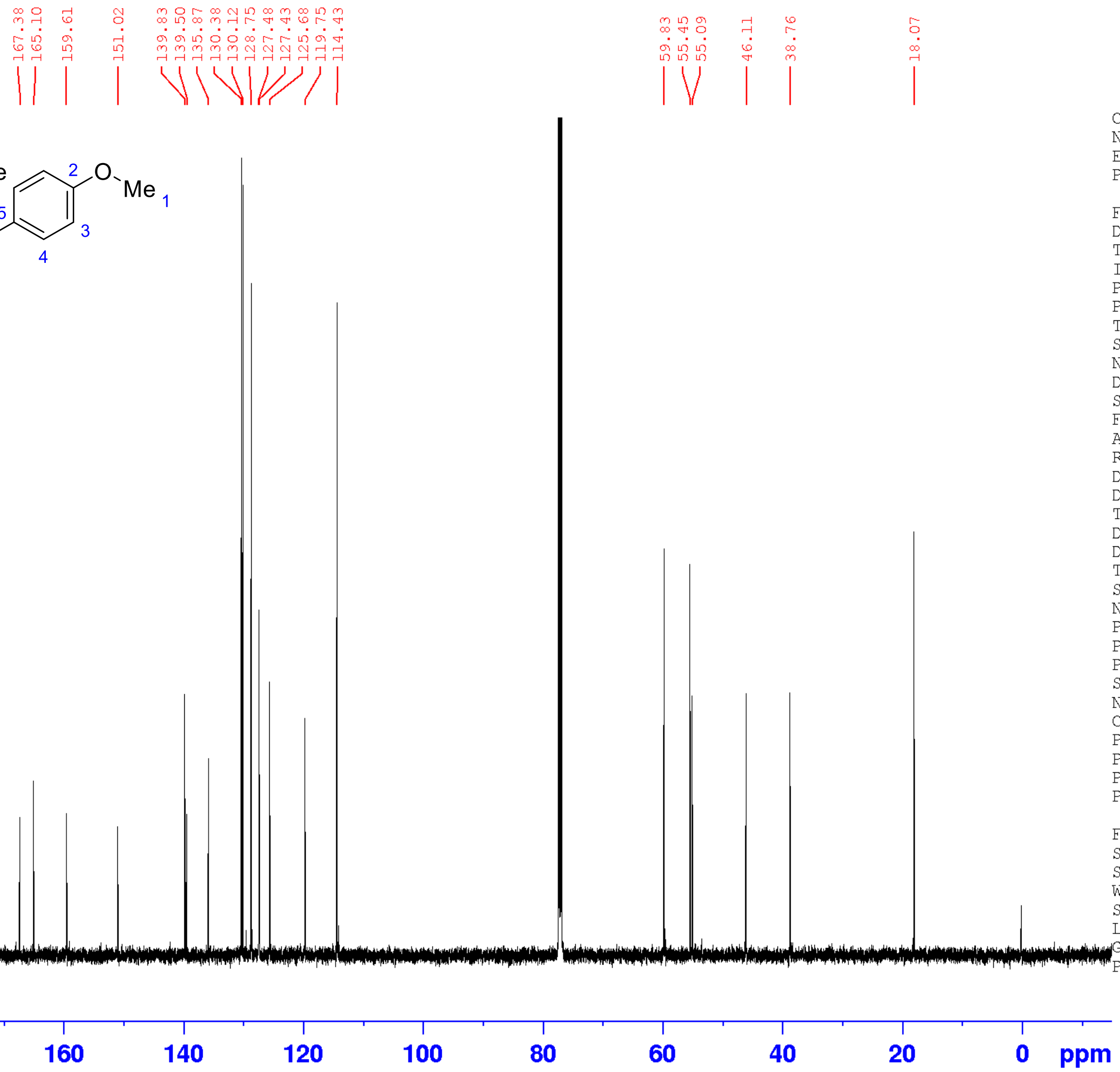

Current Data Parameters
NAME SMC-01-33 (600)
EXPNO 11
PROCNO 1

F2 - Acquisition Parameters
Date_ 20220203
Time 20.28 h
INSTRUM spect
PROBHD Z114607_0188 (
PULPROG zgpg30
TD 65536
SOLVENT CDCl3
NS 1024
DS 4
SWH 36231.883 Hz
FIDRES 1.105709 Hz
AQ 0.9043968 sec
RG 186.92
DW 13.800 usec
DE 6.50 usec
TE 296.8 K
D1 2.00000000 sec
D11 0.03000000 sec
TD0 1
SFO1 150.9178988 MHz
NUC1 13C
P0 3.93 usec
P1 11.80 usec
PLW1 85.00000000 W
SFO2 600.1324005 MHz
NUC2 1H
CPDPRG[2 waltz65
PCPD2 70.00 usec
PLW2 27.00000000 W
PLW12 0.57327998 W
PLW13 0.28836000 W

F2 - Processing parameters
SI 32768
SF 150.9027879 MHz
WDW EM
SSB 0
LB 1.00 Hz
GB 0
PC 1.40

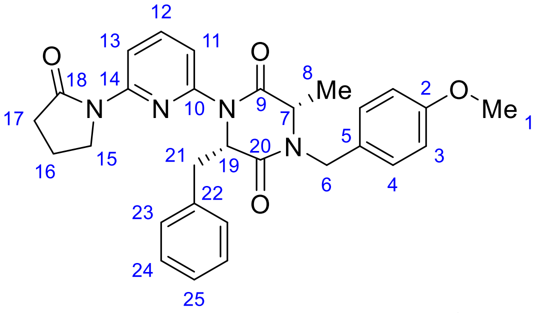

4

^{1}H NMR

600 MHz

$CDCl_3$

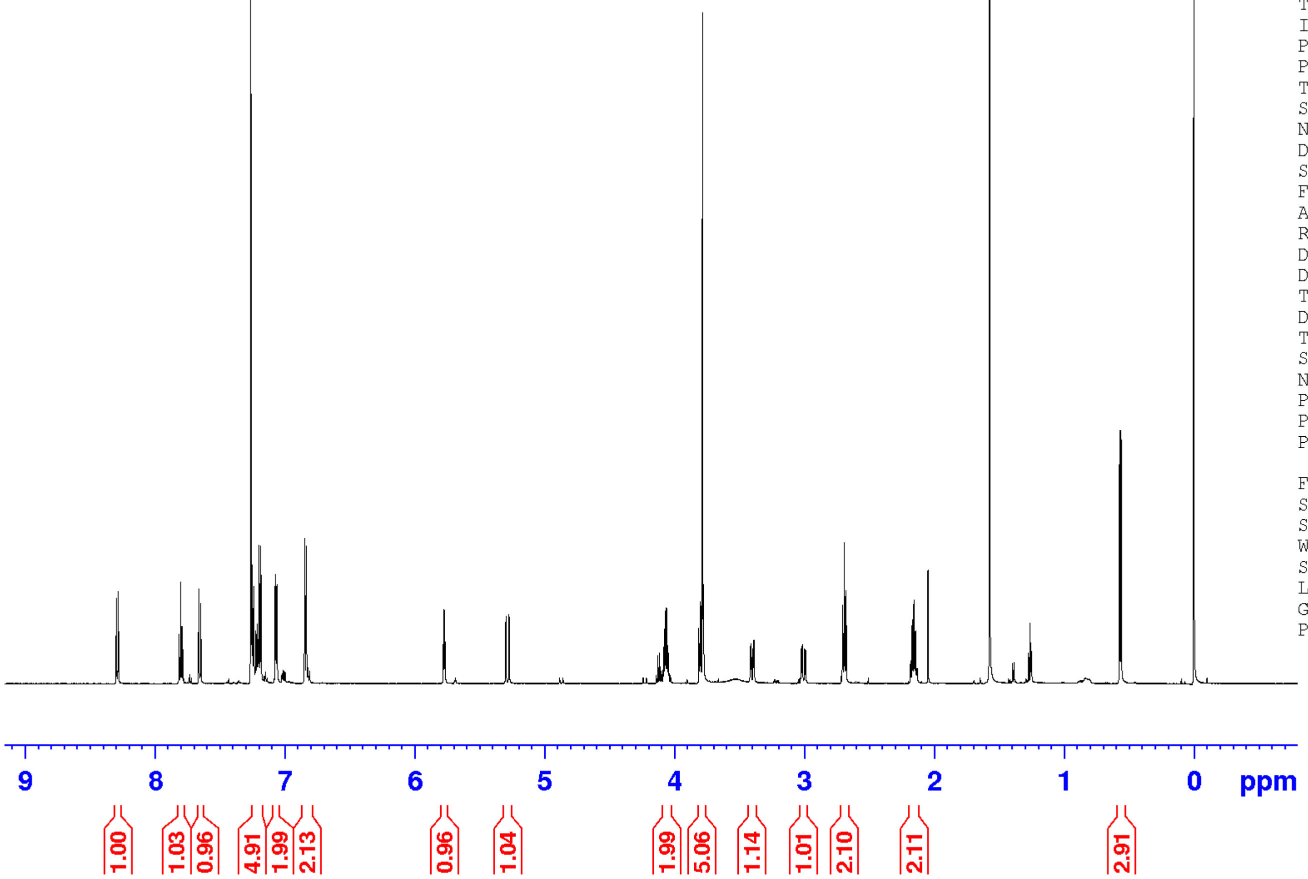


```
Current Data Parameters
NAME              SMC-01-26
EXPNO                    20
PROCNO                    1

F2 - Acquisition Parameters
Date_              20220202
Time                  15.44 h
INSTRUM               spect
PROBHD    Z114607_0188 (
PULPROG                zg30
TD                    65536
SOLVENT               CDCl3
NS                       16
DS                        2
SWH               12019.230 Hz
FIDRES             0.366798 Hz
AQ                2.7262976 sec
RG                    119.2
DW                   41.600 usec
DE                    12.10 usec
TE                    294.7 K
D1               1.00000000 sec
TD0                       1
SFO1            600.1337058 MHz
NUC1                     1H
P0                     3.33 usec
P1                    10.00 usec
PLW1            26.60000038 W

F2 - Processing parameters
SI                    65536
SF              600.1300132 MHz
WDW                      EM
SSB                       0
LB                     0.30 Hz
GB                        0
PC                     1.00
```

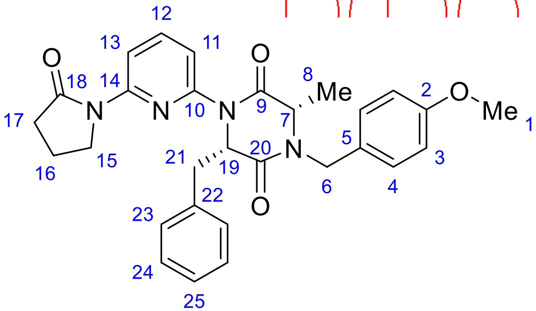

4

^{13}C NMR

151 MHz

$CDCl_3$

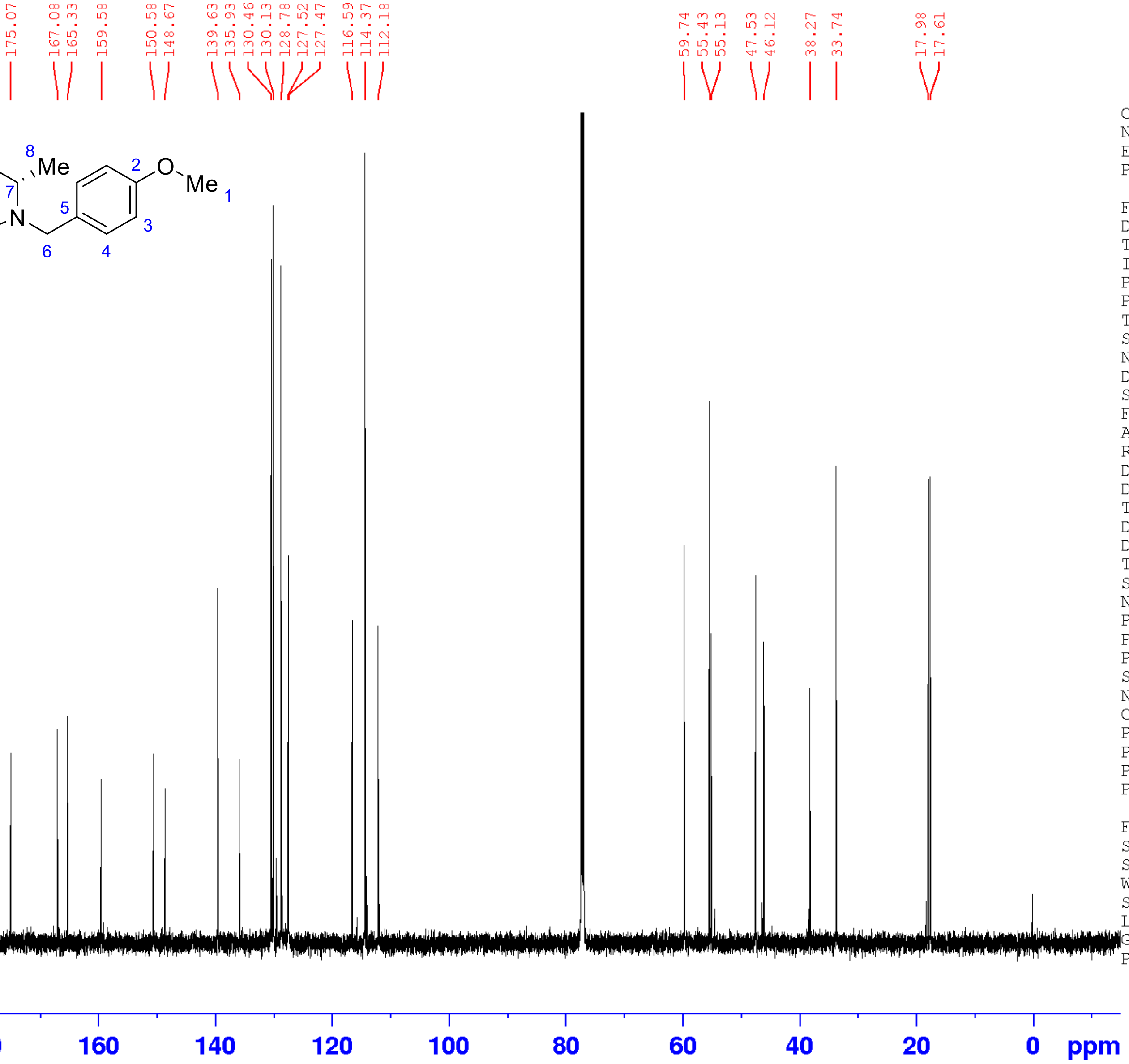


```
Current Data Parameters
NAME              SMC-01-26
EXPNO                    30
PROCNO                    1

F2 - Acquisition Parameters
Date_              20220203
Time                  18.15 h
INSTRUM               spect
PROBHD      Z114607_0188 (
PULPROG             zgpg30
TD                   65536
SOLVENT              CDCl3
NS                    1024
DS                       4
SWH              36231.883 Hz
FIDRES            1.105709 Hz
AQ               0.9043968 sec
RG                  186.92
DW                  13.800 usec
DE                    6.50 usec
TE                   296.9 K
D1              2.00000000 sec
D11             0.03000000 sec
TD0                      1
SFO1           150.9178988 MHz
NUC1                   13C
P0                    3.93 usec
P1                   11.80 usec
PLW1           85.00000000 W
SFO2           600.1324005 MHz
NUC2                    1H
CPDPRG[2           waltz65
PCPD2                70.00 usec
PLW2           27.00000000 W
PLW12           0.57327998 W
PLW13           0.28836000 W

F2 - Processing parameters
SI                   32768
SF             150.9027894 MHz
WDW                     EM
SSB                      0
LB                    1.00 Hz
GB                       0
PC                    1.40
```

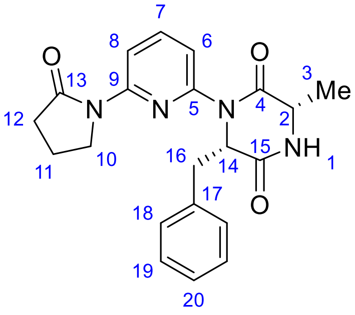

5

^{1}H NMR

600 MHz

$CDCl_3$

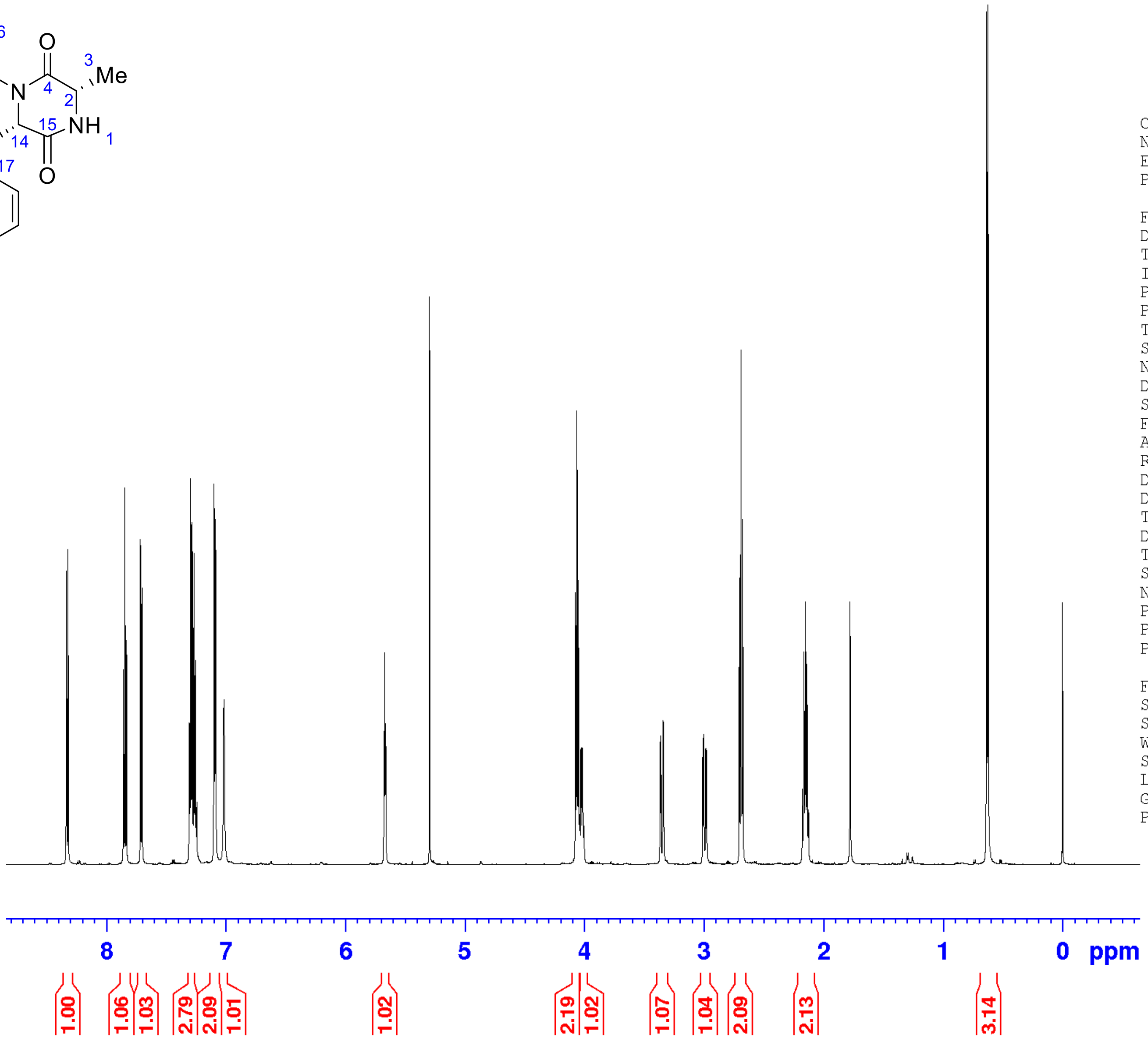

Current Data Parameters
NAME SMC-01-35
EXPNO 10
PROCNO 1

F2 - Acquisition Parameters
Date_ 20220210
Time 8.40 h
INSTRUM spect
PROBHD Z114607_0188 (
PULPROG zg30
TD 65536
SOLVENT CDCl3
NS 16
DS 2
SWH 12019.230 Hz
FIDRES 0.366798 Hz
AQ 2.7262976 sec
RG 68
DW 41.600 usec
DE 12.10 usec
TE 300.0 K
D1 1.00000000 sec
TD0 1
SFO1 600.1337058 MHz
NUC1 1H
P0 3.33 usec
P1 10.00 usec
PLW1 26.60000038 W

F2 - Processing parameters
SI 65536
SF 600.1300074 MHz
WDW EM
SSB 0
LB 0.30 Hz
GB 0
PC 1.00

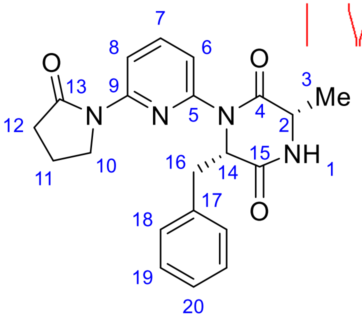

5

^{13}C NMR

151 MHz

$CDCl_3$

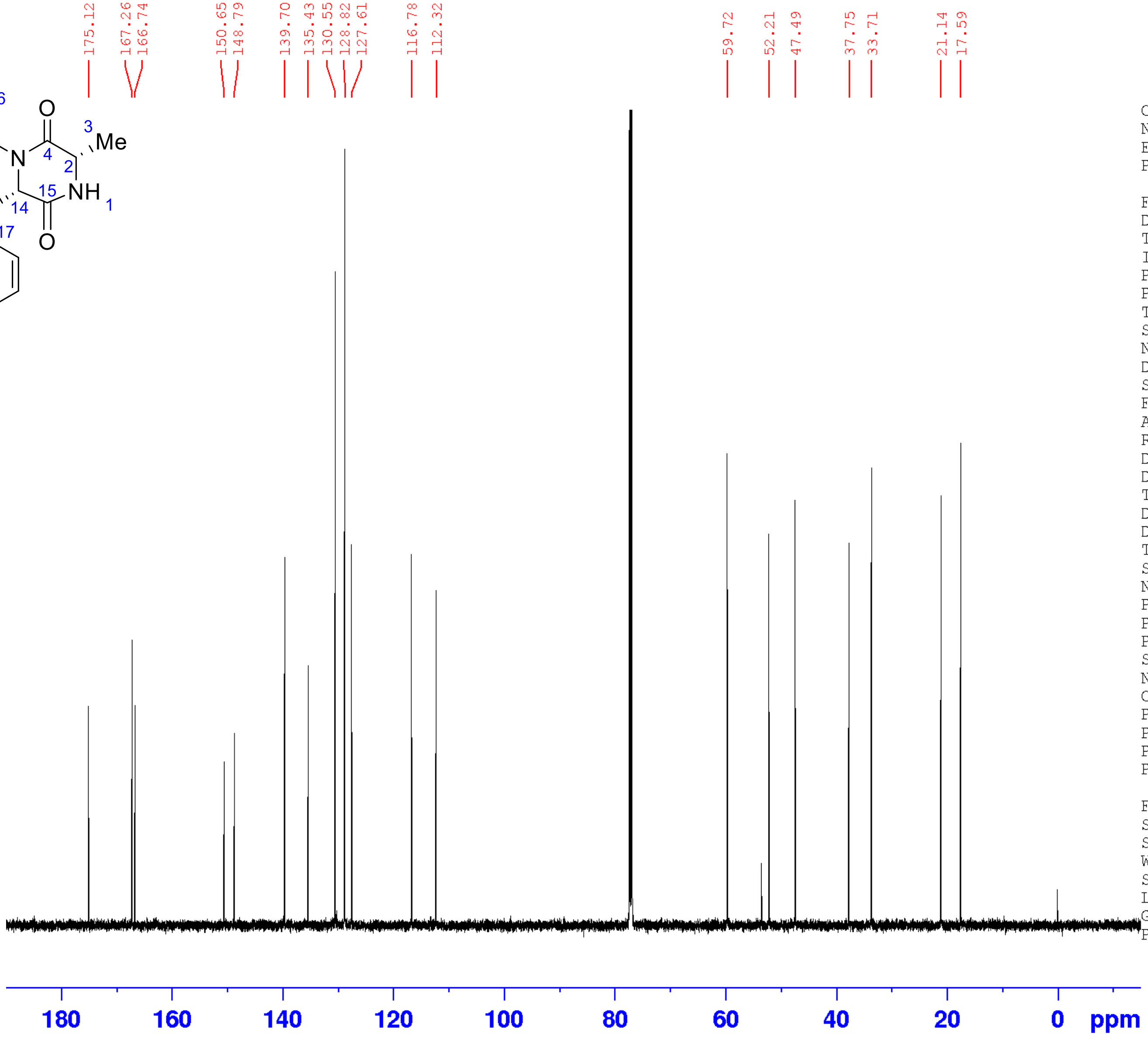

Current Data Parameters
NAME SMC-01-35
EXPNO 11
PROCNO 1

F2 - Acquisition Parameters
Date_ 20220210
Time 9.32 h
INSTRUM spect
PROBHD Z114607_0188 (
PULPROG zgpg30
TD 65536
SOLVENT CDCl3
NS 1024
DS 4
SWH 36231.883 Hz
FIDRES 1.105709 Hz
AQ 0.9043968 sec
RG 186.92
DW 13.800 usec
DE 6.50 usec
TE 300.2 K
D1 2.00000000 sec
D11 0.03000000 sec
TD0 1
SFO1 150.9178988 MHz
NUC1 13C
P0 3.93 usec
P1 11.80 usec
PLW1 85.00000000 W
SFO2 600.1324005 MHz
NUC2 1H
CPDPRG[2 waltz65
PCPD2 70.00 usec
PLW2 27.00000000 W
PLW12 0.57327998 W
PLW13 0.28836000 W

F2 - Processing parameters
SI 32768
SF 150.9027918 MHz
WDW EM
SSB 0
LB 1.00 Hz
GB 0
PC 1.40

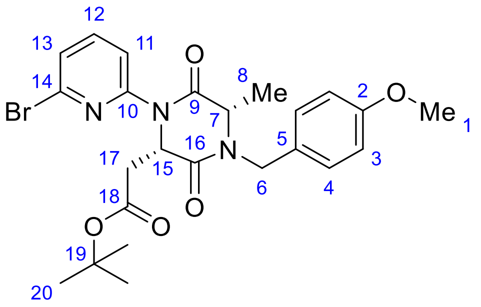

3b

^{1}H NMR

600 MHz

$CDCl_3$

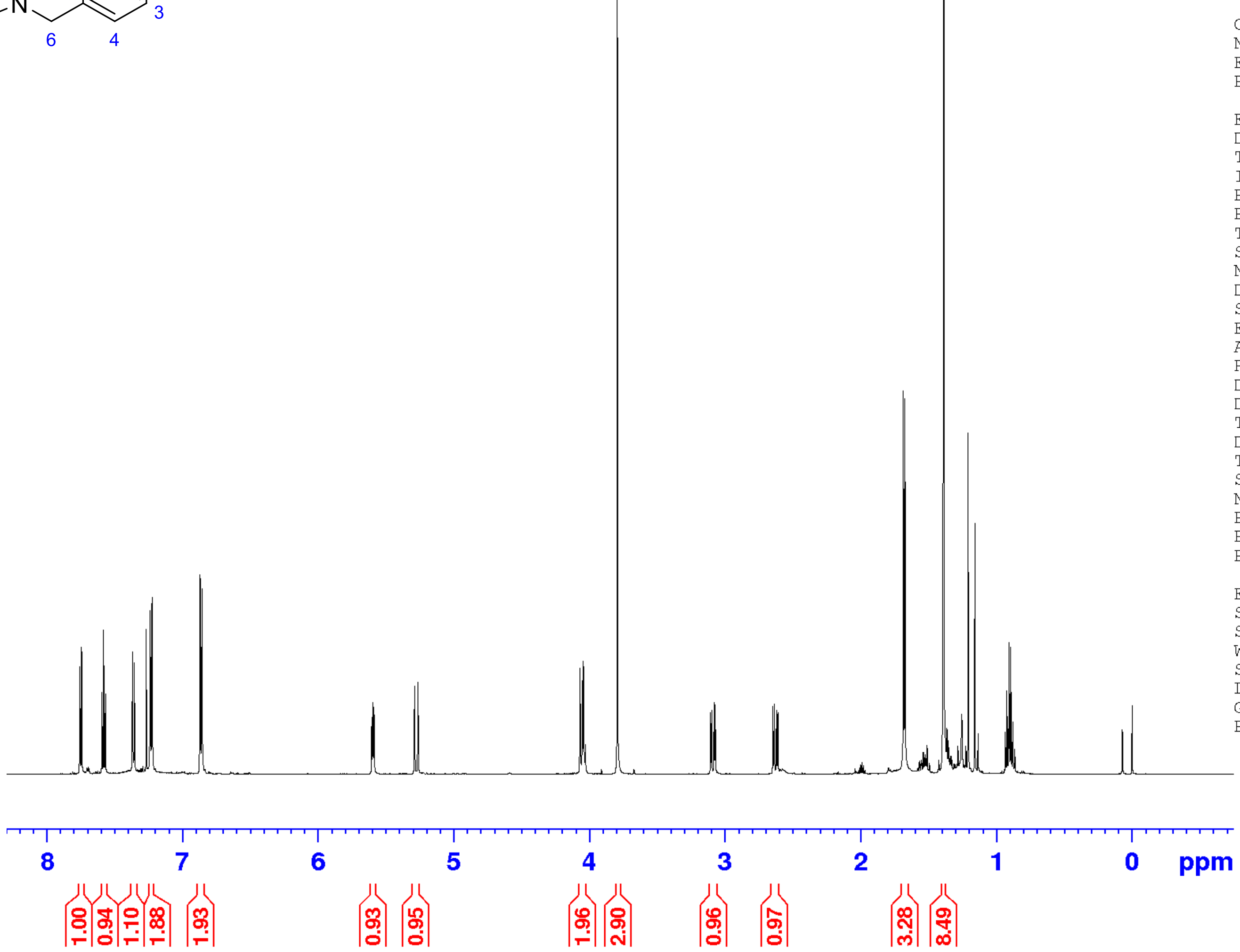


```
Current Data Parameters
NAME        SMC-01-45-600
EXPNO                  10
PROCNO                  1

F2 - Acquisition Parameters
Date_            20220304
Time                 6.11 h
INSTRUM             spect
PROBHD   Z114607_0188 (
PULPROG              zg30
TD                  65536
SOLVENT             CDCl3
NS                     16
DS                      2
SWH             12019.230 Hz
FIDRES           0.366798 Hz
AQ              2.7262976 sec
RG                  60.48
DW                 41.600 usec
DE                  12.10 usec
TE                  300.0 K
D1             1.00000000 sec
TD0                     1
SFO1          600.1337058 MHz
NUC1                   1H
P0                   3.33 usec
P1                  10.00 usec
PLW1          26.60000038 W

F2 - Processing parameters
SI                  65536
SF            600.1300109 MHz
WDW                    EM
SSB                     0
LB                   0.30 Hz
GB                      0
PC                   1.00
```

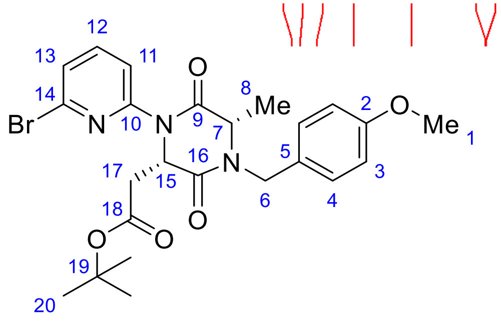

3b

^{13}C NMR

151 MHz

$CDCl_3$

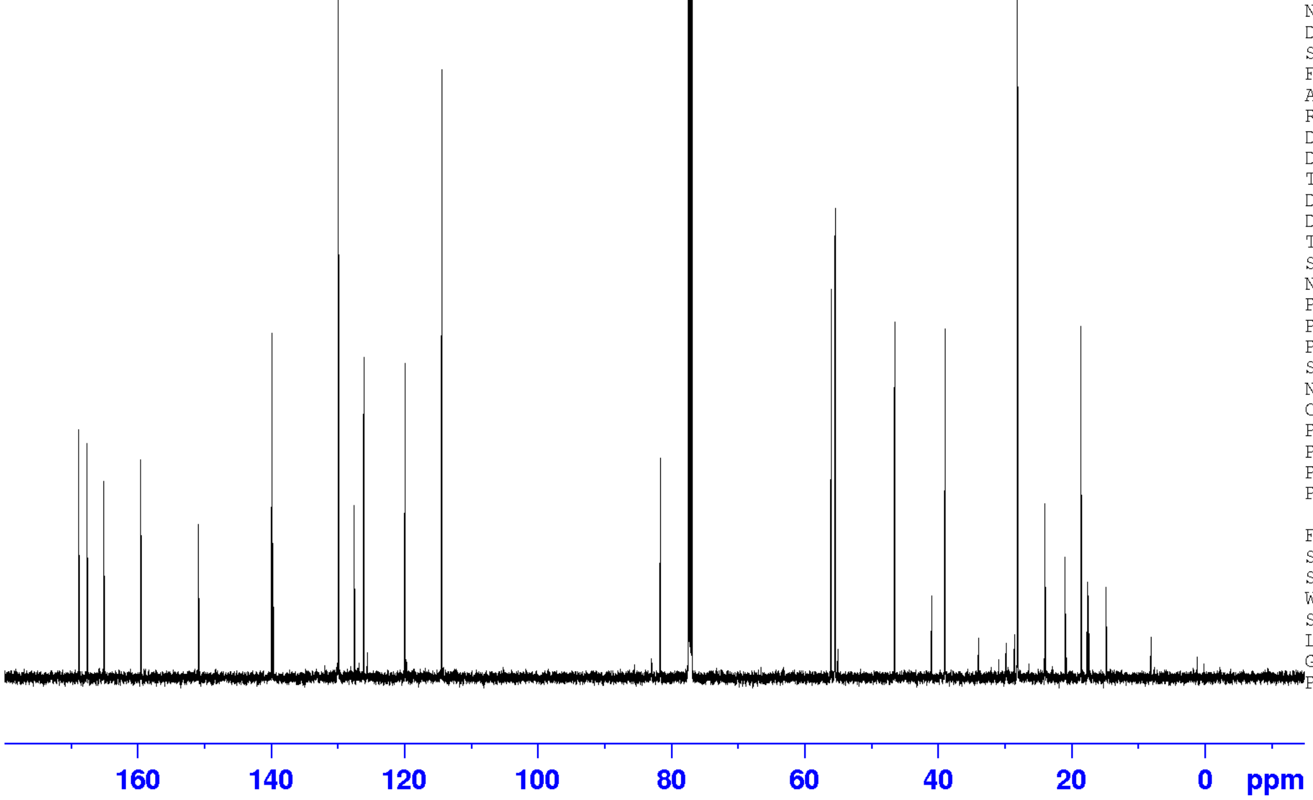


```
Current Data Parameters
NAME          SMC-01-45-600
EXPNO                    11
PROCNO                    1

F2 - Acquisition Parameters
Date_              20220304
Time                   7.03 h
INSTRUM               spect
PROBHD     Z114607_0188 (
PULPROG             zgpg30
TD                   65536
SOLVENT              CDCl3
NS                    1024
DS                       4
SWH              36231.883 Hz
FIDRES            1.105709 Hz
AQ               0.9043968 sec
RG                  186.92
DW                  13.800 usec
DE                    6.50 usec
TE                   300.0 K
D1              2.00000000 sec
D11             0.03000000 sec
TD0                      1
SFO1           150.9178988 MHz
NUC1                   13C
P0                    3.93 usec
P1                   11.80 usec
PLW1           85.00000000 W
SFO2           600.1324005 MHz
NUC2                    1H
CPDPRG[2           waltz65
PCPD2                70.00 usec
PLW2           27.00000000 W
PLW12           0.57327998 W
PLW13           0.28836000 W

F2 - Processing parameters
SI                   32768
SF             150.9027901 MHz
WDW                     EM
SSB                      0
LB                    1.00 Hz
GB                       0
PC                    1.40
```

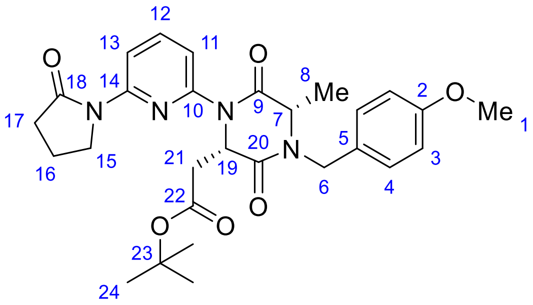

10

^{1}H NMR

400 MHz

$CDCl_3$

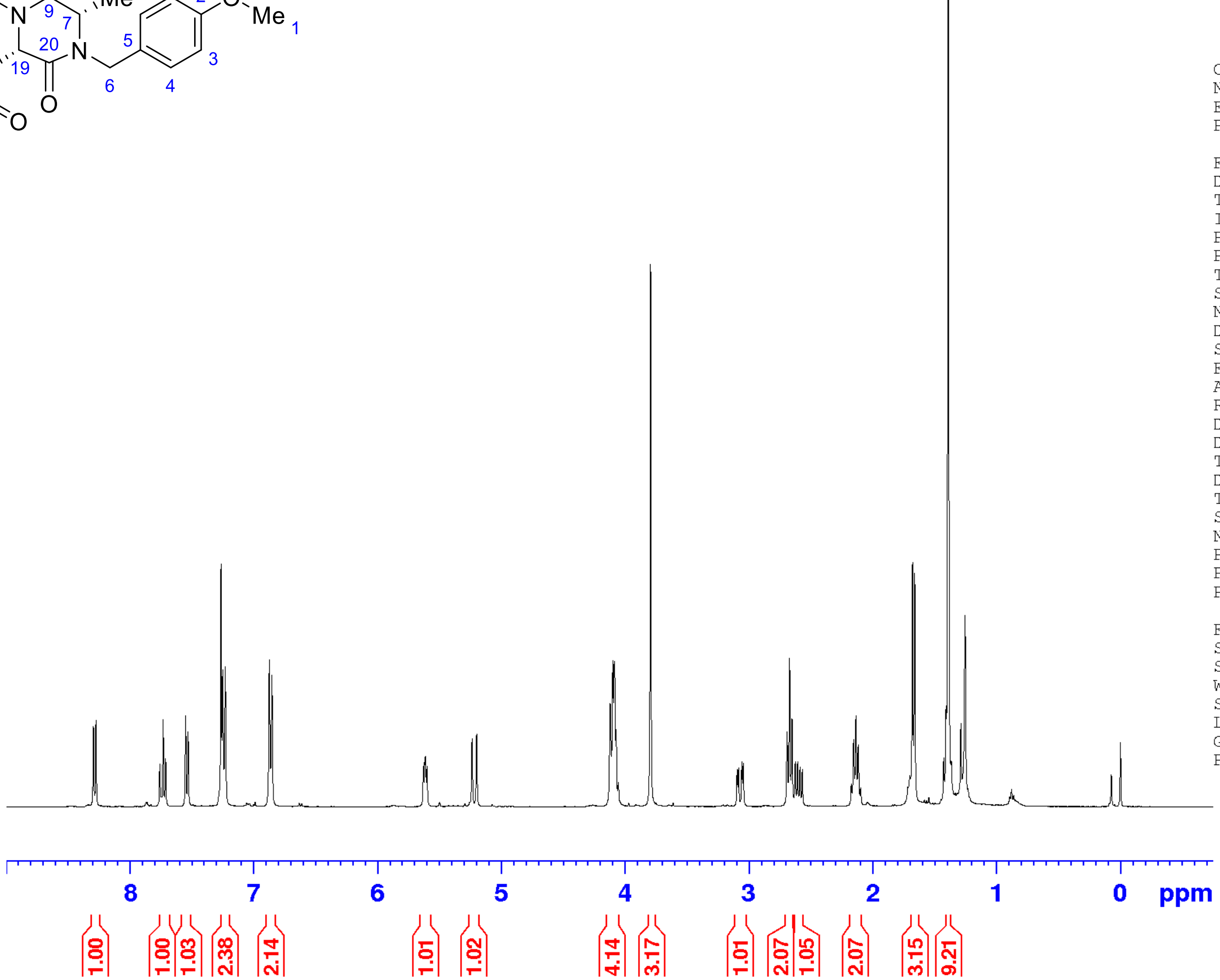


```
Current Data Parameters
NAME      WR 3.433 (V2)
EXPNO                10
PROCNO                1

F2 - Acquisition Parameters
Date_          20230301
Time             8.41 h
INSTRUM       AVIII_400
PROBHD   Z108618_0146 (
PULPROG            zg30
TD                65536
SOLVENT           CDCl3
NS                   16
DS                    2
SWH            8223.685 Hz
FIDRES         0.250967 Hz
AQ            3.9845889 sec
RG                  114
DW               60.800 usec
DE                17.42 usec
TE                300.0 K
D1           1.00000000 sec
TD0                   1
SFO1        400.1124708 MHz
NUC1                 1H
P0                 5.00 usec
P1                15.00 usec
PLW1        17.29199982 W

F2 - Processing parameters
SI                32768
SF          400.1100066 MHz
WDW                  EM
SSB                   0
LB                 0.30 Hz
GB                    0
PC                 1.00
```

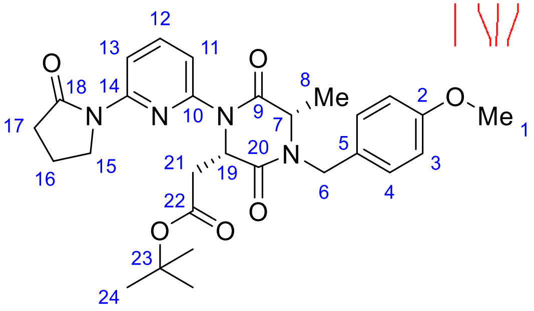

10

^{13}C NMR

101 MHz

$CDCl_3$

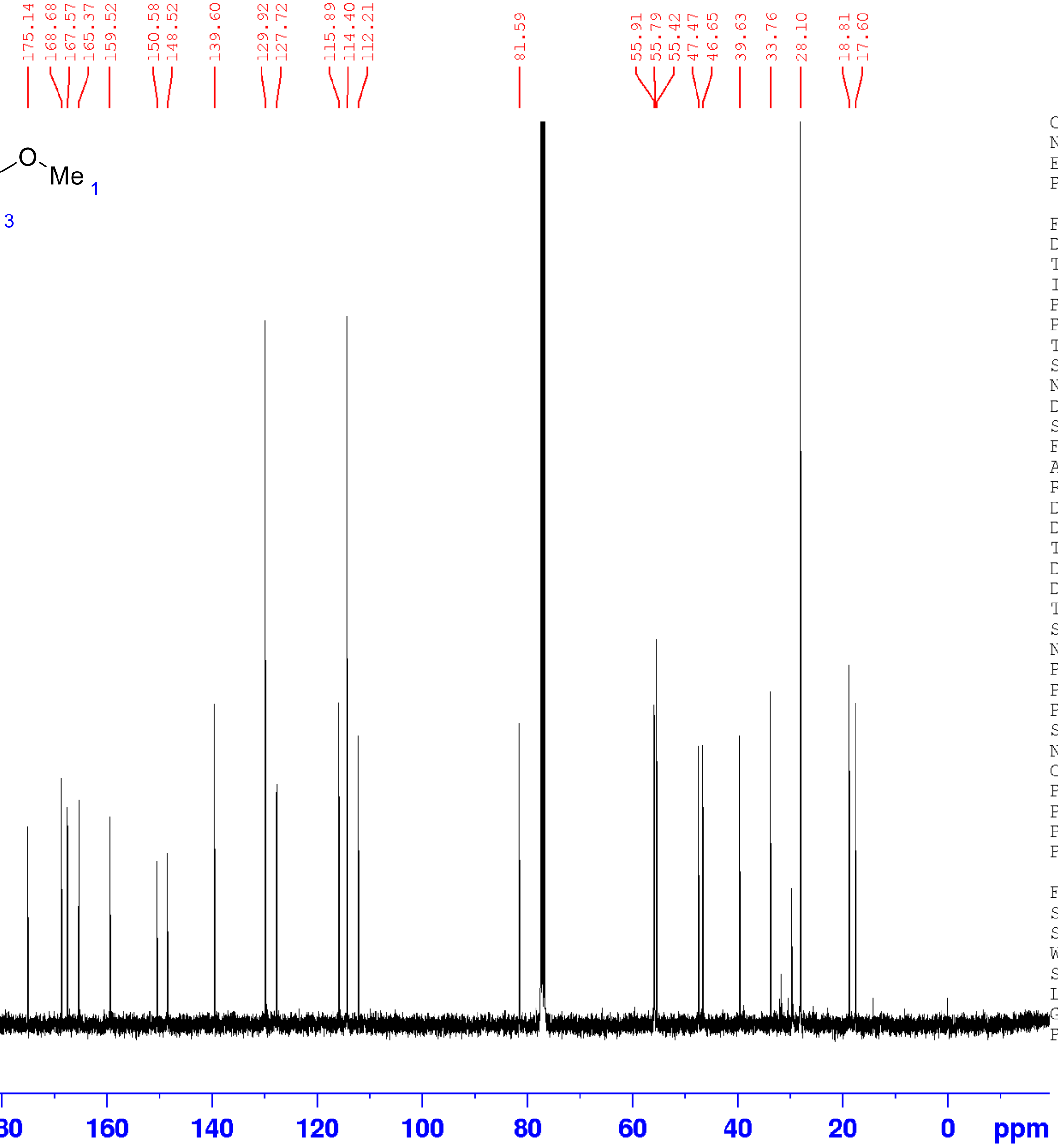

Current Data Parameters
NAME WR 3.433 (V2)
EXPNO 11
PROCNO 1

F2 - Acquisition Parameters
Date_ 20230301
Time 9.36 h
INSTRUM AVIII_400
PROBHD Z108618_0146 (
PULPROG zgpg30
TD 96150
SOLVENT CDCl3
NS 1024
DS 4
SWH 24038.461 Hz
FIDRES 0.500020 Hz
AQ 1.9999200 sec
RG 2050
DW 20.800 usec
DE 6.50 usec
TE 300.0 K
D1 1.00000000 sec
D11 0.03000000 sec
TD0 1
SFO1 100.6178003 MHz
NUC1 13C
P0 2.90 usec
P1 8.70 usec
PLW1 96.68000031 W
SFO2 400.1116004 MHz
NUC2 1H
CPDPRG[2 waltz64
PCPD2 90.00 usec
PLW2 17.29199982 W
PLW12 0.48032999 W
PLW13 0.24160001 W

F2 - Processing parameters
SI 131072
SF 100.6077288 MHz
WDW EM
SSB 0
LB 1.00 Hz
GB 0
PC 1.40

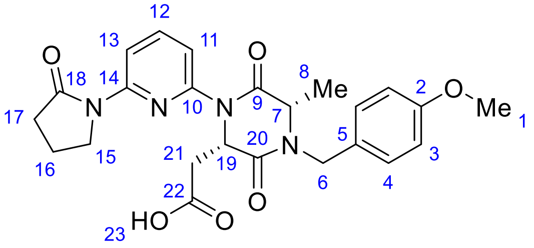

10a

^{1}H NMR

400 MHz

d_6-DMSO

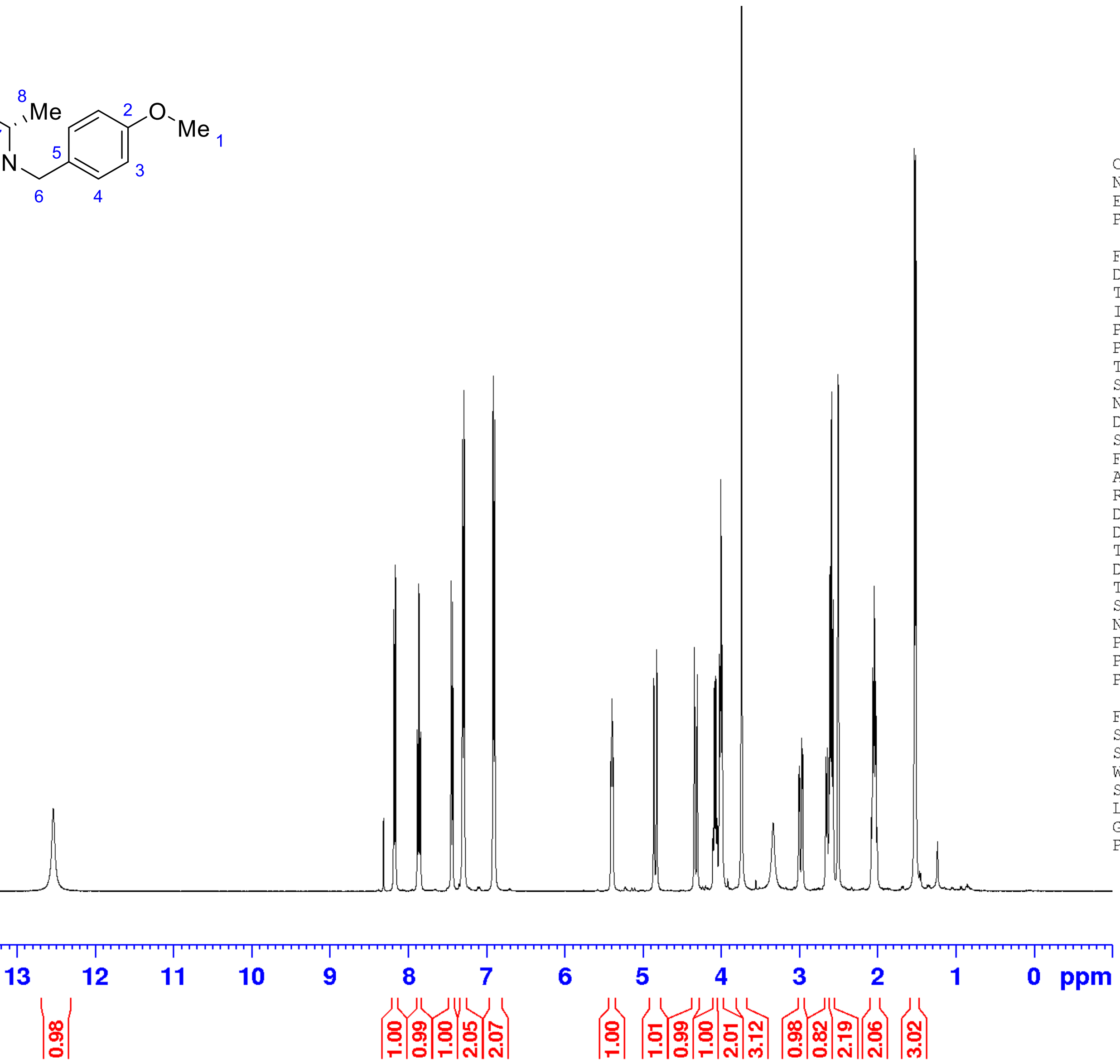

Current Data Parameters
NAME WR 3.451 (DMSO)
EXPNO 10
PROCNO 1

F2 - Acquisition Parameters
Date_ 20230310
Time 17.28 h
INSTRUM AVIII_400
PROBHD Z108618_0146 (
PULPROG zg30
TD 65536
SOLVENT DMSO
NS 16
DS 2
SWH 8223.685 Hz
FIDRES 0.250967 Hz
AQ 3.9845889 sec
RG 161
DW 60.800 usec
DE 17.42 usec
TE 300.0 K
D1 1.00000000 sec
TD0 1
SFO1 400.1124708 MHz
NUC1 1H
P0 5.00 usec
P1 15.00 usec
PLW1 17.29199982 W

F2 - Processing parameters
SI 32768
SF 400.1100000 MHz
WDW EM
SSB 0
LB 0.30 Hz
GB 0
PC 1.00

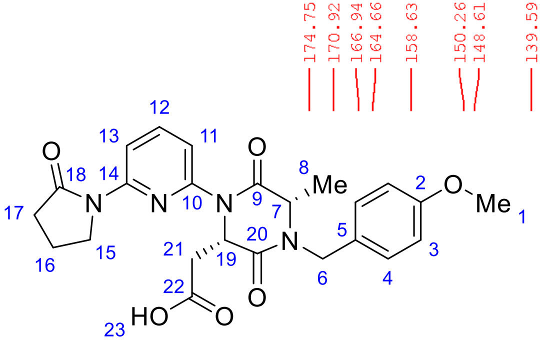

10a

^{13}C NMR

101 MHz

d$_6$-DMSO

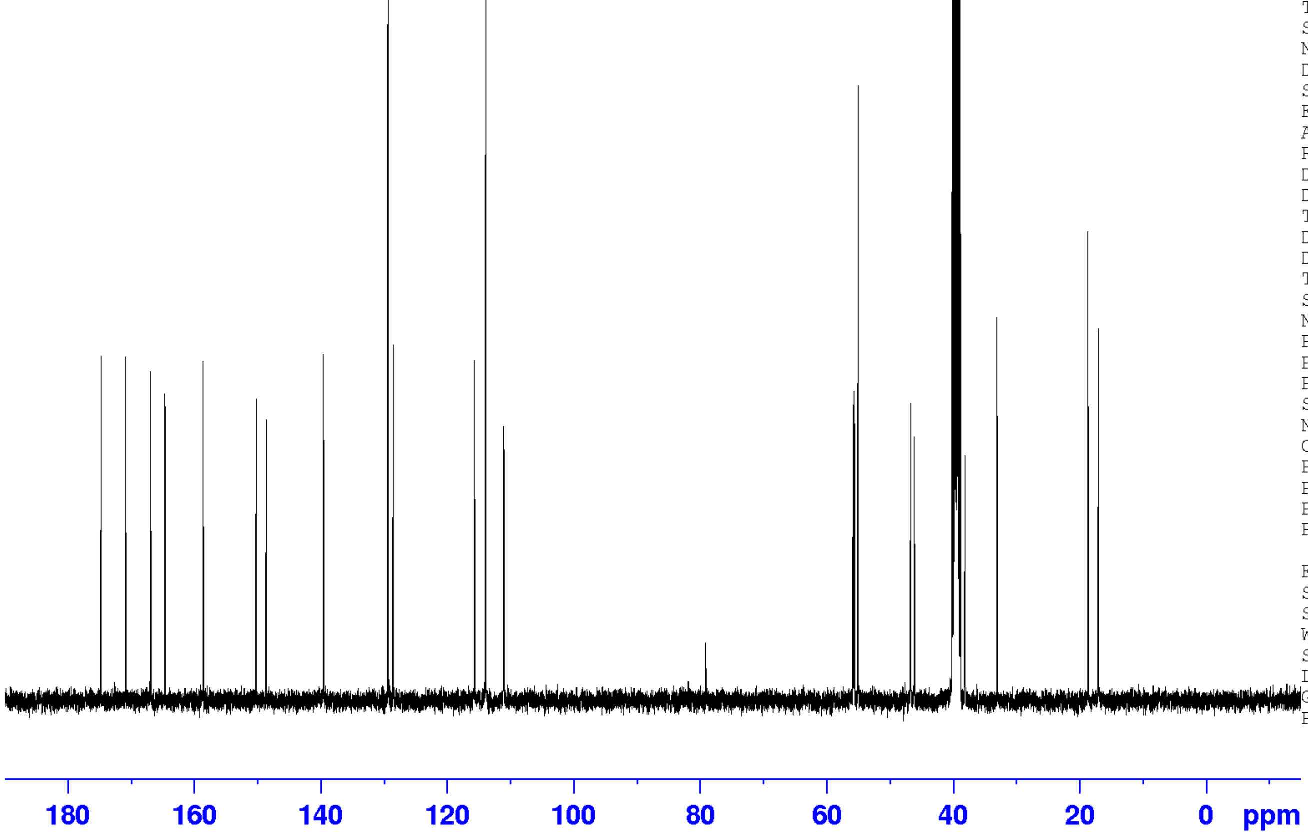

Current Data Parameters
NAME WR 3.451 (DMSO)
EXPNO 12
PROCNO 1

F2 - Acquisition Parameters
Date_ 20230311
Time 7.24 h
INSTRUM AVIII_400
PROBHD Z108618_0146 (
PULPROG zgpg30
TD 96150
SOLVENT DMSO
NS 2048
DS 4
SWH 24038.461 Hz
FIDRES 0.500020 Hz
AQ 1.9999200 sec
RG 2050
DW 20.800 usec
DE 6.50 usec
TE 300.0 K
D1 1.00000000 sec
D11 0.03000000 sec
TD0 1
SFO1 100.6178003 MHz
NUC1 13C
P0 2.90 usec
P1 8.70 usec
PLW1 96.68000031 W
SFO2 400.1116004 MHz
NUC2 1H
CPDPRG[2 waltz64
PCPD2 90.00 usec
PLW2 17.29199982 W
PLW12 0.48032999 W
PLW13 0.24160001 W

F2 - Processing parameters
SI 131072
SF 100.6077867 MHz
WDW EM
SSB 0
LB 1.00 Hz
GB 0
PC 1.40

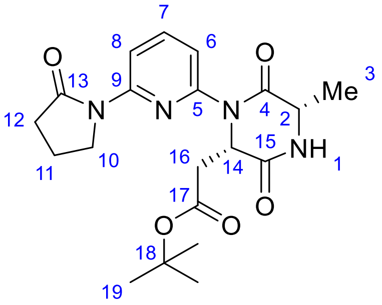

11

^{1}H NMR

600 MHz

$CDCl_3$

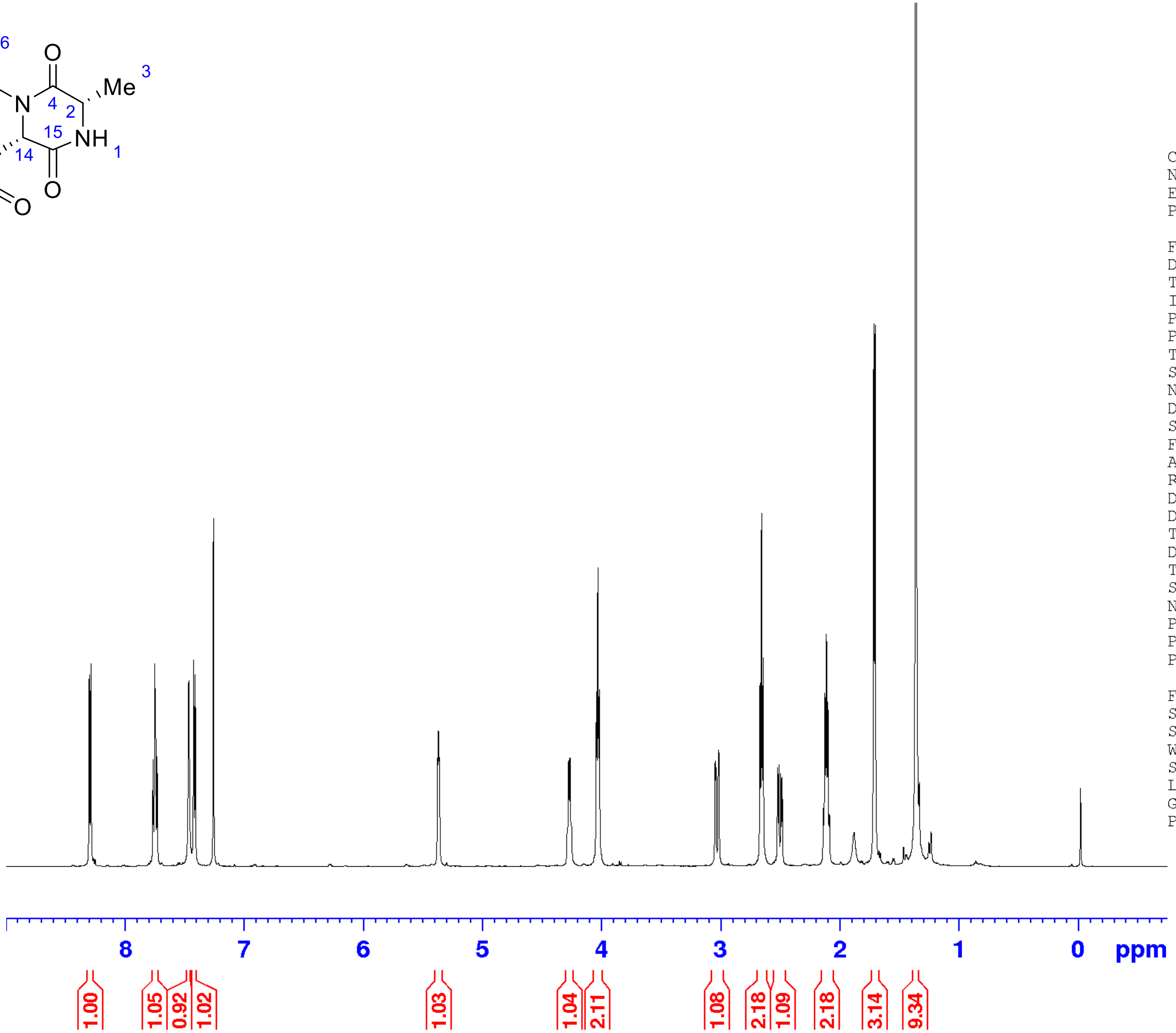


```
Current Data Parameters
NAME       WR 3.366 (600)
EXPNO                  10
PROCNO                  1

F2 - Acquisition Parameters
Date_            20220928
Time                17.31 h
INSTRUM             spect
PROBHD     Z114607_0188 (
PULPROG              zg30
TD                  65536
SOLVENT             CDCl3
NS                     16
DS                      2
SWH             12019.230 Hz
FIDRES           0.366798 Hz
AQ              2.7262976 sec
RG                  31.58
DW                 41.600 usec
DE                  12.10 usec
TE                  295.4 K
D1             1.00000000 sec
TD0                     1
SFO1          600.1337058 MHz
NUC1                   1H
P0                   3.33 usec
P1                  10.00 usec
PLW1          26.60000038 W

F2 - Processing parameters
SI                  65536
SF            600.1300140 MHz
WDW                    EM
SSB                     0
LB                   0.30 Hz
GB                      0
PC                   1.00
```

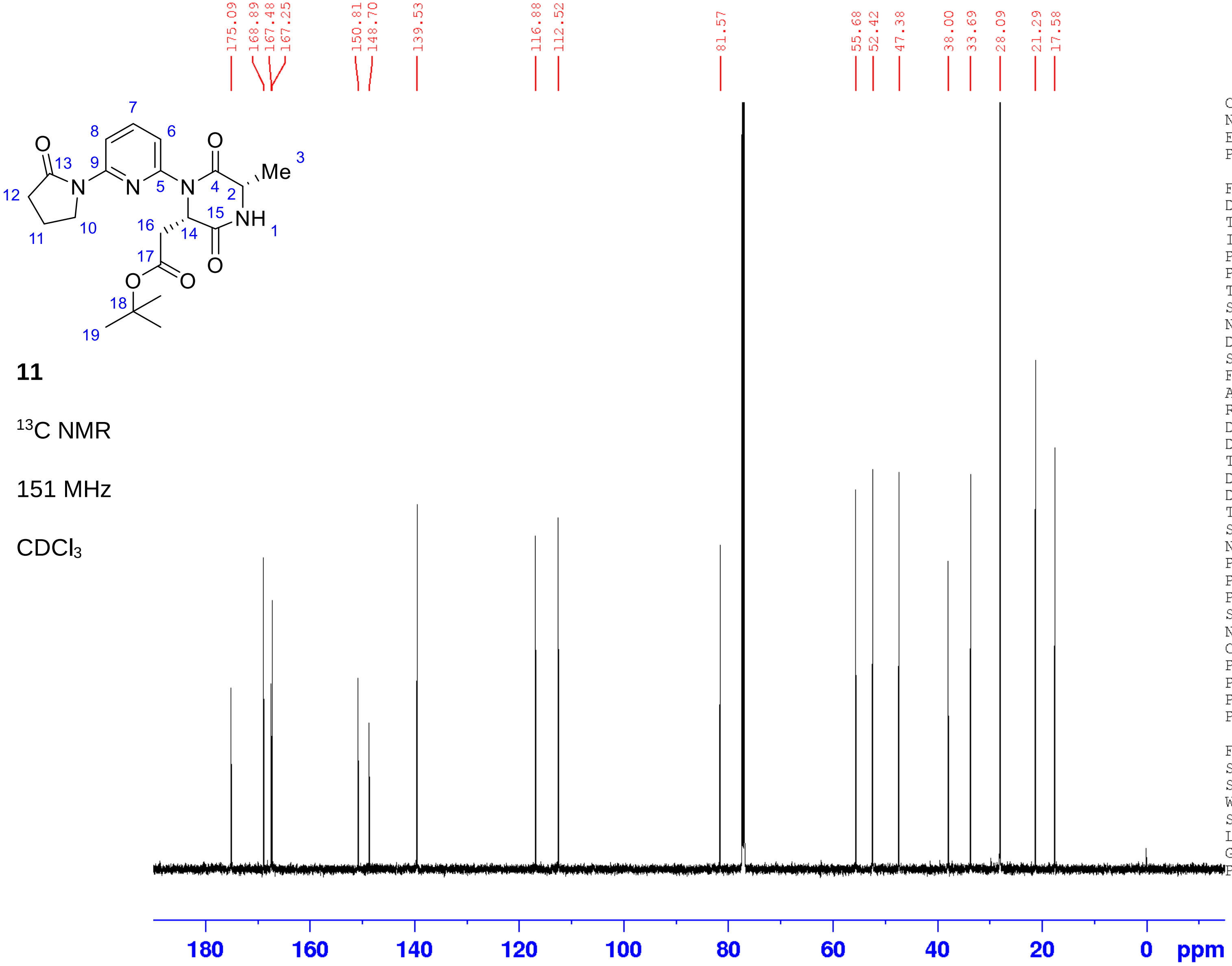

11

^{13}C NMR

151 MHz

$CDCl_3$

```
Current Data Parameters
NAME       WR 3.366 (600)
EXPNO                  11
PROCNO                  1

F2 - Acquisition Parameters
Date_            20220928
Time                18.23 h
INSTRUM             spect
PROBHD    Z114607_0188 (
PULPROG           zgpg30
TD                 65536
SOLVENT            CDCl3
NS                  1024
DS                     4
SWH            36231.883 Hz
FIDRES          1.105709 Hz
AQ             0.9043968 sec
RG                186.92
DW                13.800 usec
DE                  6.50 usec
TE                 297.2 K
D1            2.00000000 sec
D11           0.03000000 sec
TD0                    1
SFO1         150.9178988 MHz
NUC1                 13C
P0                  3.93 usec
P1                 11.80 usec
PLW1         85.00000000 W
SFO2         600.1324005 MHz
NUC2                  1H
CPDPRG[2         waltz65
PCPD2              70.00 usec
PLW2         27.00000000 W
PLW12         0.57327998 W
PLW13         0.28836000 W

F2 - Processing parameters
SI                 32768
SF           150.9027920 MHz
WDW                   EM
SSB                    0
LB                  1.00 Hz
GB                     0
PC                  1.40
```

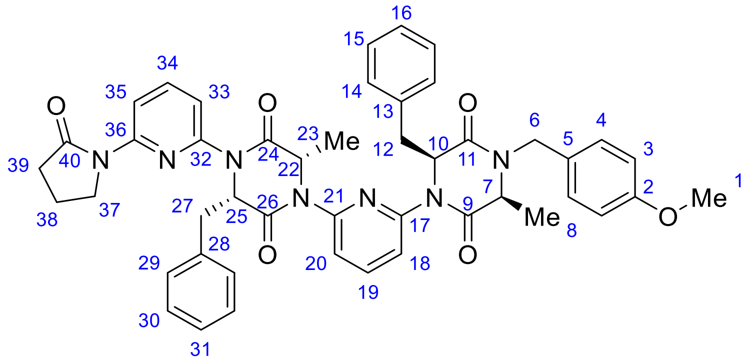

6

^{1}H NMR

600 MHz

$CDCl_3$

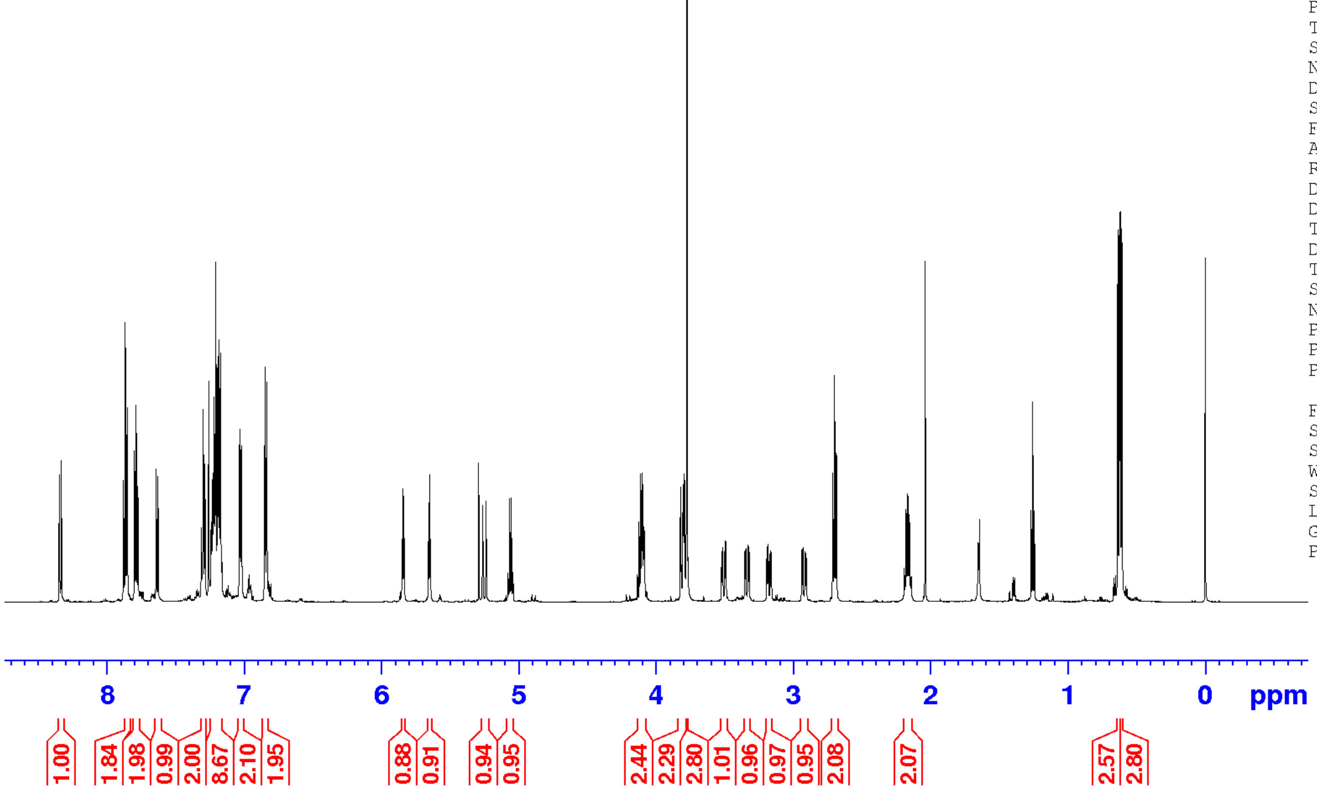


```
Current Data Parameters
NAME              SMC-01-39
EXPNO                    10
PROCNO                    1

F2 - Acquisition Parameters
Date_              20220212
Time                 1.11 h
INSTRUM               spect
PROBHD     Z114607_0188 (
PULPROG                zg30
TD                    65536
SOLVENT               CDCl3
NS                       16
DS                        2
SWH              12019.230 Hz
FIDRES            0.366798 Hz
AQ               2.7262976 sec
RG                       68
DW                  41.600 usec
DE                   12.10 usec
TE                   300.0 K
D1              1.00000000 sec
TD0                       1
SFO1           600.1337058 MHz
NUC1                     1H
P0                    3.33 usec
P1                   10.00 usec
PLW1           26.60000038 W

F2 - Processing parameters
SI                    65536
SF             600.1300128 MHz
WDW                      EM
SSB                       0
LB                     0.30 Hz
GB                        0
PC                     1.00
```

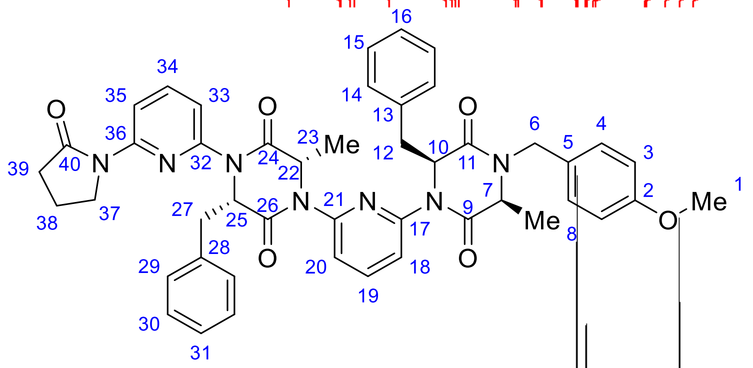

6

^{13}C NMR

151 MHz

$CDCl_3$

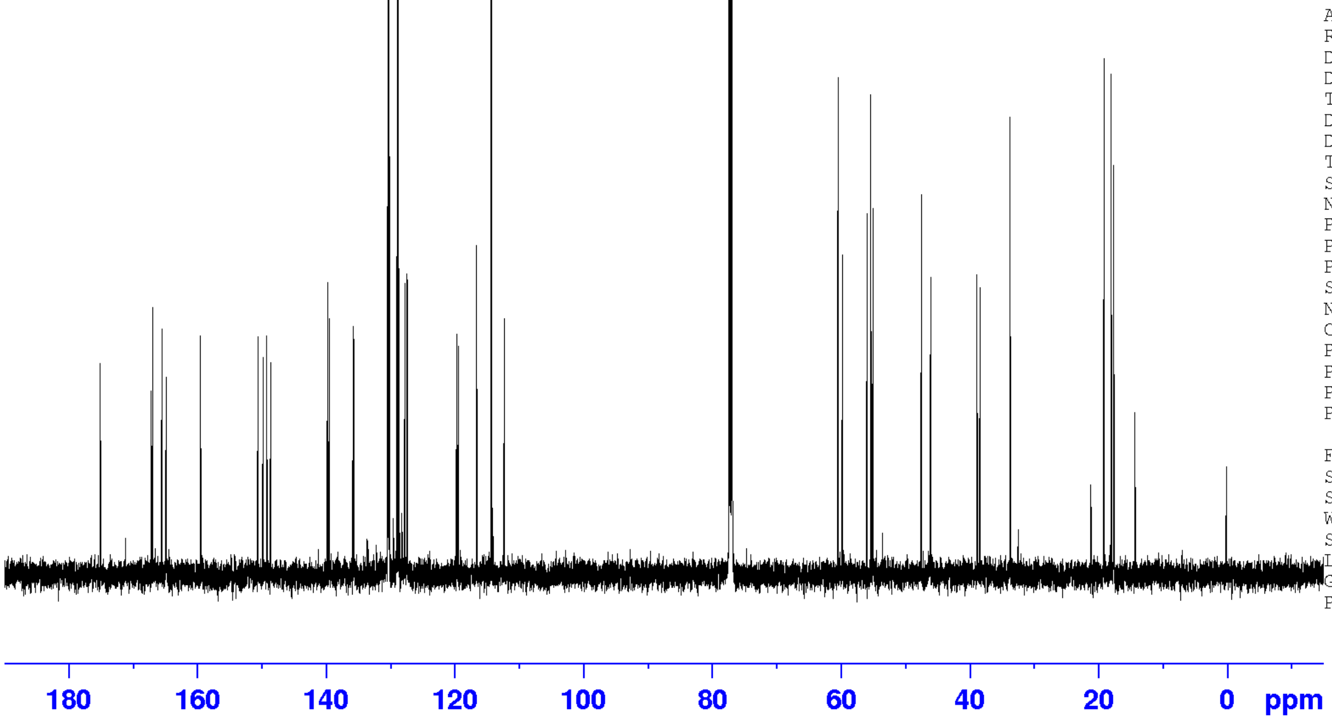

Current Data Parameters
NAME SMC-01-39
EXPNO 11
PROCNO 1

F2 - Acquisition Parameters
Date_ 20220212
Time 2.03 h
INSTRUM spect
PROBHD Z114607_0188 (
PULPROG zgpg30
TD 65536
SOLVENT CDCl3
NS 1024
DS 4
SWH 36231.883 Hz
FIDRES 1.105709 Hz
AQ 0.9043968 sec
RG 186.92
DW 13.800 usec
DE 6.50 usec
TE 300.0 K
D1 2.00000000 sec
D11 0.03000000 sec
TD0 1
SFO1 150.9178988 MHz
NUC1 13C
P0 3.93 usec
P1 11.80 usec
PLW1 85.00000000 W
SFO2 600.1324005 MHz
NUC2 1H
CPDPRG[2 waltz65
PCPD2 70.00 usec
PLW2 27.00000000 W
PLW12 0.57327998 W
PLW13 0.28836000 W

F2 - Processing parameters
SI 32768
SF 150.9027908 MHz
WDW EM
SSB 0
LB 1.00 Hz
GB 0
PC 1.40

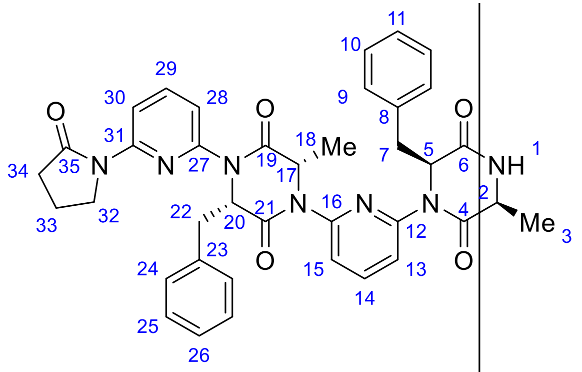

7

^{1}H NMR

600 MHz

$CDCl_3$

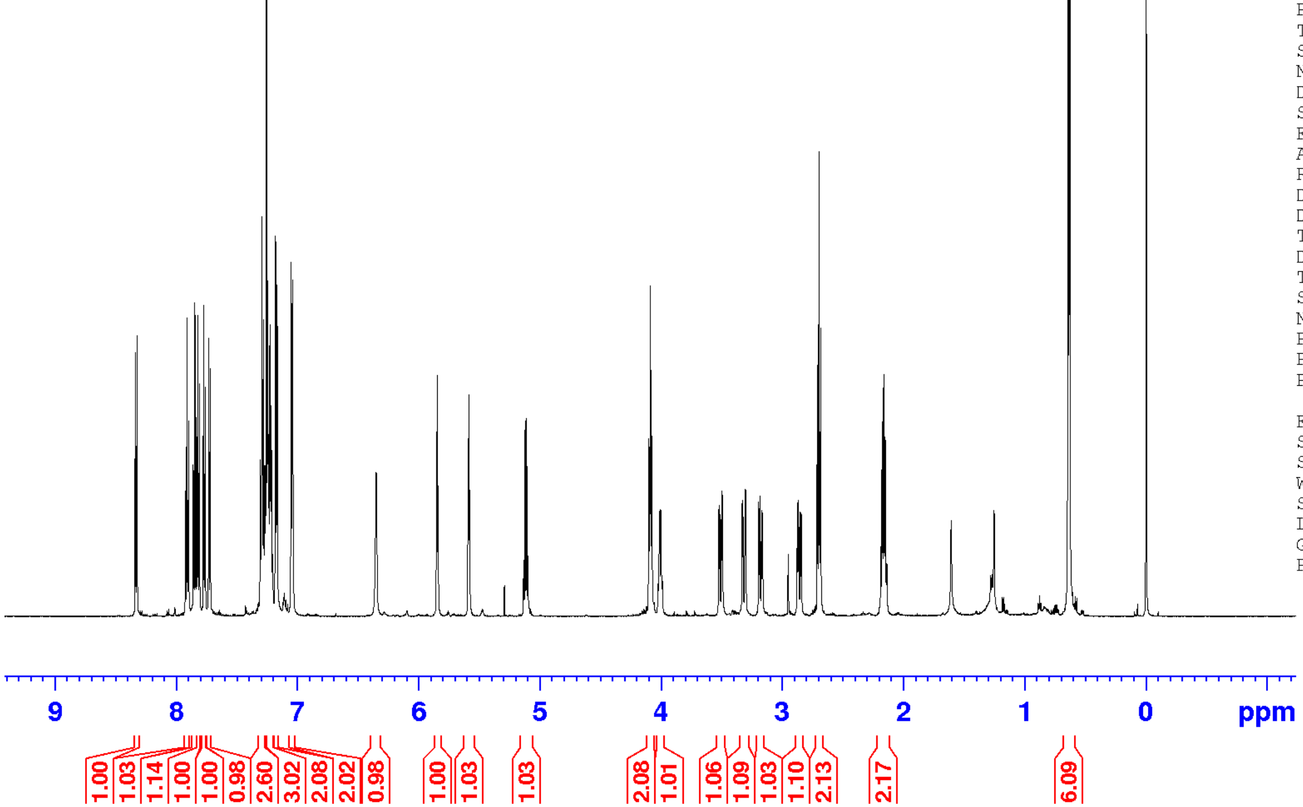


```
Current Data Parameters
NAME              SMC-01-40
EXPNO                    10
PROCNO                    1

F2 - Acquisition Parameters
Date_              20220217
Time                  20.22 h
INSTRUM               spect
PROBHD     Z114607_0188 (
PULPROG                zg30
TD                    65536
SOLVENT               CDCl3
NS                       16
DS                        2
SWH               12019.230 Hz
FIDRES             0.366798 Hz
AQ                2.7262976 sec
RG                     97.5
DW                   41.600 usec
DE                    12.10 usec
TE                    300.0 K
D1               1.00000000 sec
TD0                       1
SFO1            600.1337058 MHz
NUC1                     1H
P0                     3.33 usec
P1                    10.00 usec
PLW1            26.60000038 W

F2 - Processing parameters
SI                    65536
SF              600.1300145 MHz
WDW                      EM
SSB                       0
LB                     0.30 Hz
GB                        0
PC                     1.00
```

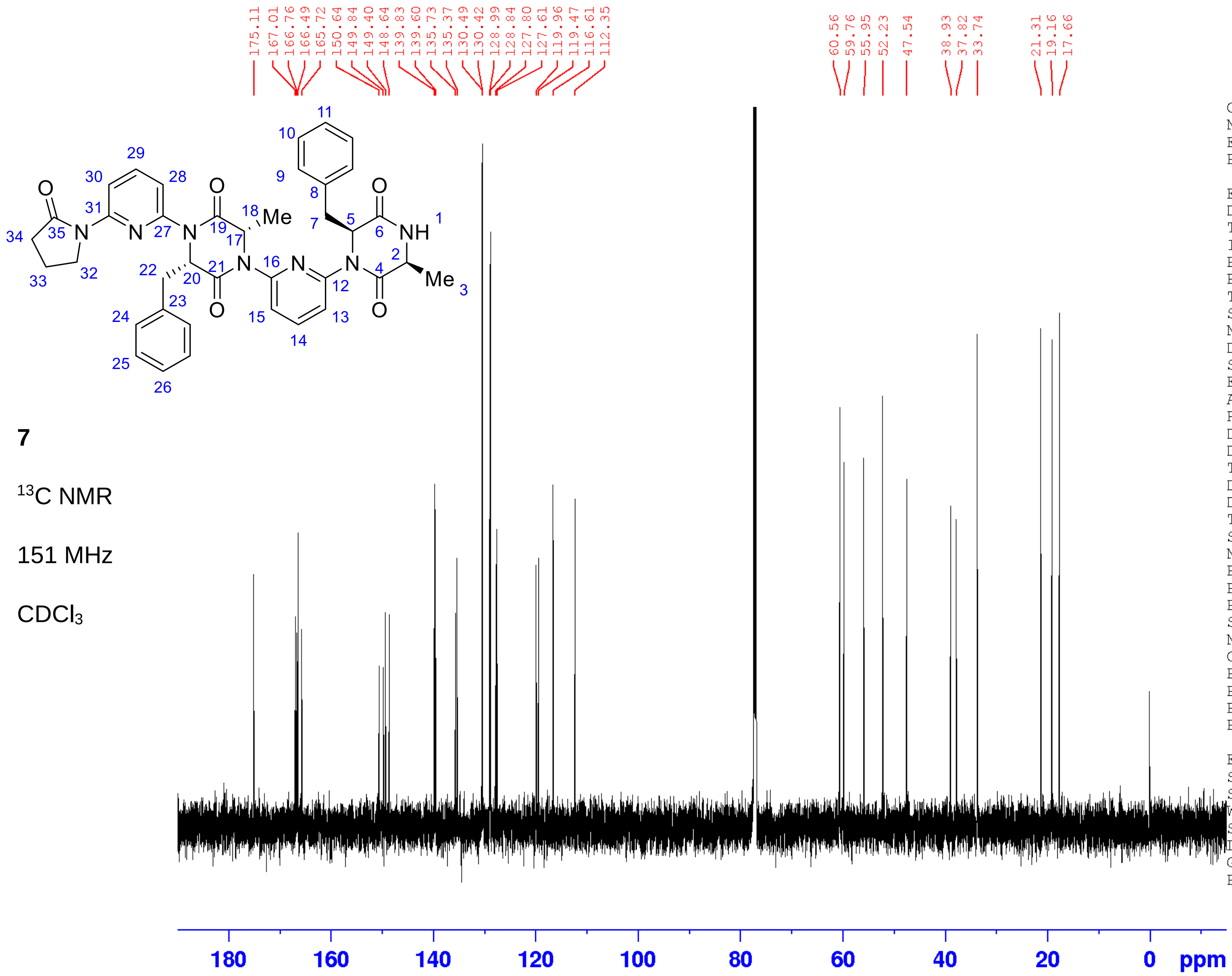

7

^{13}C NMR

151 MHz

$CDCl_3$

```
Current Data Parameters
NAME              SMC-01-40
EXPNO                    11
PROCNO                    1

F2 - Acquisition Parameters
Date_              20220217
Time                  21.14 h
INSTRUM               spect
PROBHD    Z114607_0188 (
PULPROG             zgpg30
TD                   65536
SOLVENT              CDCl3
NS                    1024
DS                       4
SWH              36231.883 Hz
FIDRES            1.105709 Hz
AQ               0.9043968 sec
RG                  186.92
DW                  13.800 usec
DE                    6.50 usec
TE                   300.0 K
D1              2.00000000 sec
D11             0.03000000 sec
TD0                      1
SFO1           150.9178988 MHz
NUC1                   13C
P0                    3.93 usec
P1                   11.80 usec
PLW1           85.00000000 W
SFO2           600.1324005 MHz
NUC2                    1H
CPDPRG[2           waltz65
PCPD2                70.00 usec
PLW2           27.00000000 W
PLW12           0.57327998 W
PLW13           0.28836000 W

F2 - Processing parameters
SI                   32768
SF             150.9027883 MHz
WDW                     EM
SSB                      0
LB                    1.00 Hz
GB                       0
PC                    1.40
```

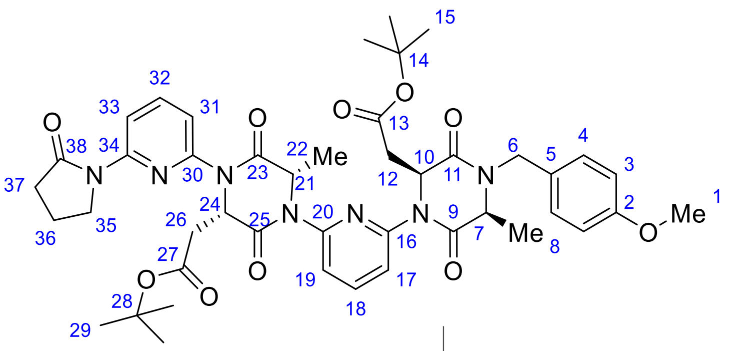

12

^1H NMR

400 MHz

$CDCl_3$

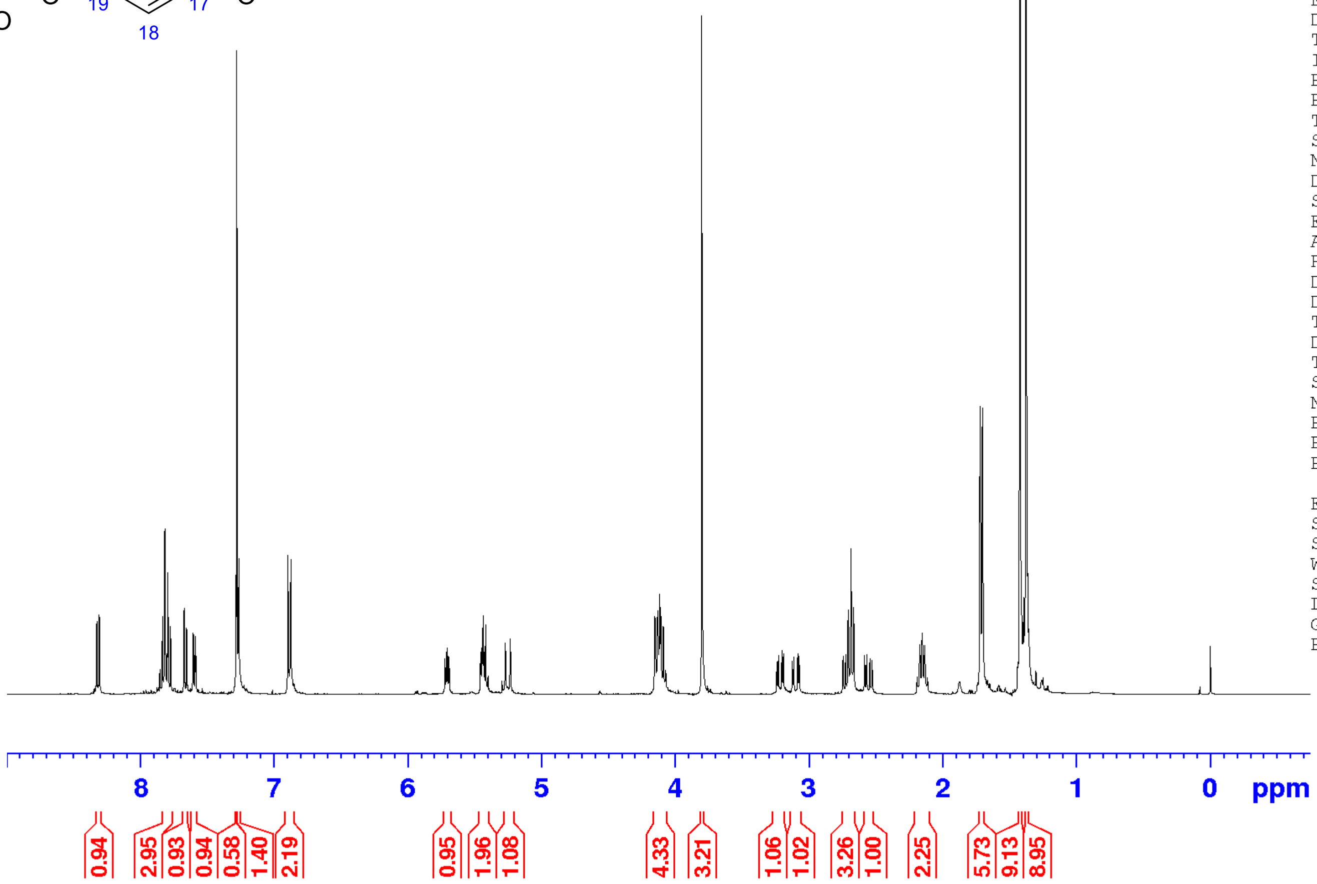


```
Current Data Parameters
NAME              WR 3.432
EXPNO                   10
PROCNO                   1

F2 - Acquisition Parameters
Date_             20230125
Time                 15.04 h
INSTRUM          AVIII_400
PROBHD     Z108618_0817 (
PULPROG               zg30
TD                   65536
SOLVENT              CDCl3
NS                      16
DS                       2
SWH               8223.685 Hz
FIDRES            0.250967 Hz
AQ               3.9845889 sec
RG                      18
DW                  60.800 usec
DE                    6.50 usec
TE                   296.3 K
D1              1.00000000 sec
TD0                      1
SFO1           399.9124696 MHz
NUC1                    1H
P0                    7.33 usec
P1                   22.00 usec
PLW1           31.62299919 W

F2 - Processing parameters
SI                   32768
SF             399.9100012 MHz
WDW                     EM
SSB                      0
LB                    0.30 Hz
GB                       0
PC                    1.00
```

12

^{13}C NMR

101 MHz

$CDCl_3$

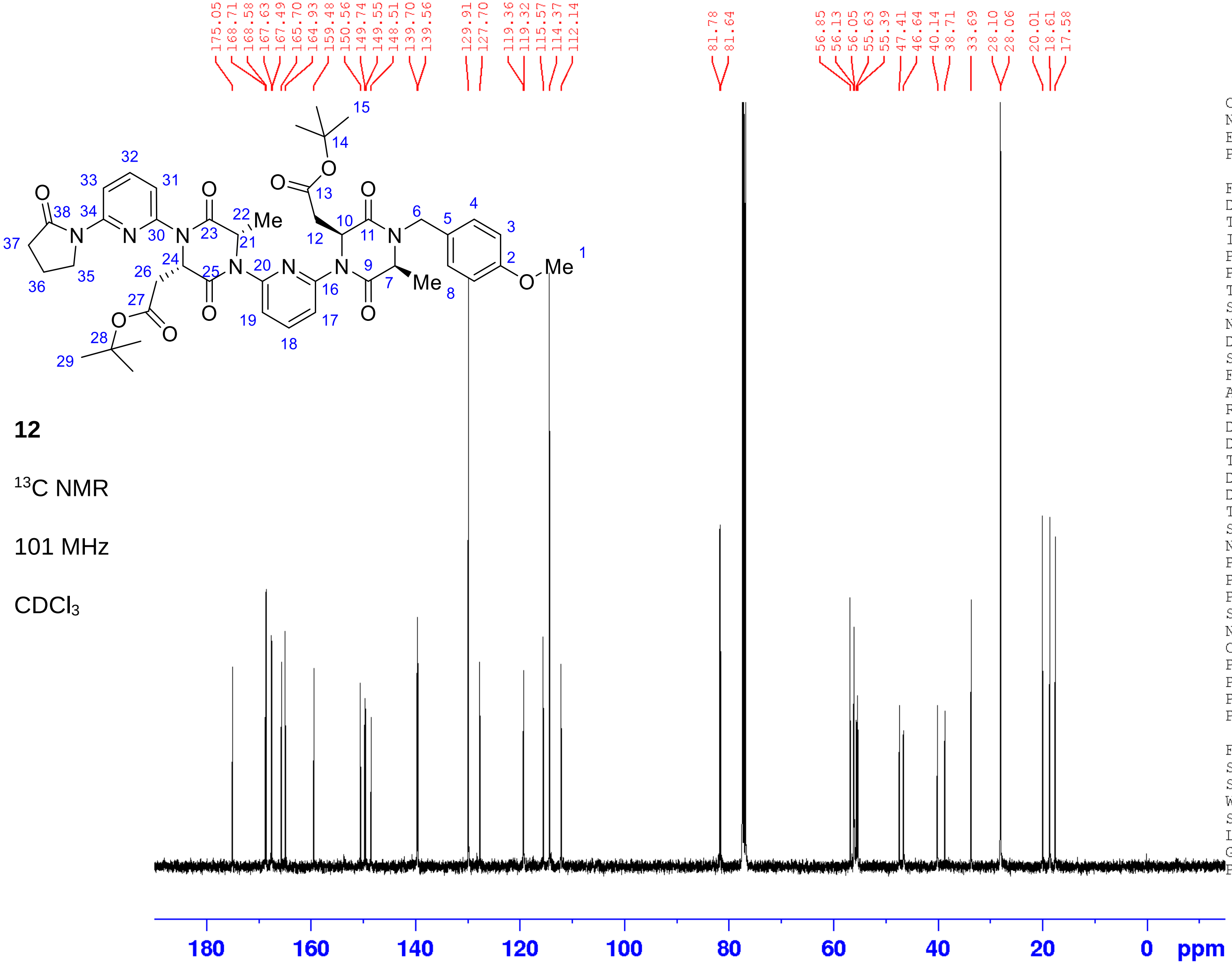

Current Data Parameters
NAME WR 3.432
EXPNO 11
PROCNO 1

F2 - Acquisition Parameters
Date_ 20230126
Time 0.28 h
INSTRUM AVIII_400
PROBHD Z108618_0817 (
PULPROG zgpg30
TD 96150
SOLVENT CDCl3
NS 1024
DS 4
SWH 24038.461 Hz
FIDRES 0.500020 Hz
AQ 1.9999200 sec
RG 181
DW 20.800 usec
DE 6.50 usec
TE 299.8 K
D1 1.00000000 sec
D11 0.03000000 sec
TD0 1
SFO1 100.5675047 MHz
NUC1 13C
P0 1.93 usec
P1 5.80 usec
PLW1 96.68000031 W
SFO2 399.9115996 MHz
NUC2 1H
CPDPRG[2 waltz64
PCPD2 90.00 usec
PLW2 31.62299919 W
PLW12 0.48032999 W
PLW13 0.24160001 W

F2 - Processing parameters
SI 131072
SF 100.5574416 MHz
WDW EM
SSB 0
LB 1.00 Hz
GB 0
PC 1.40

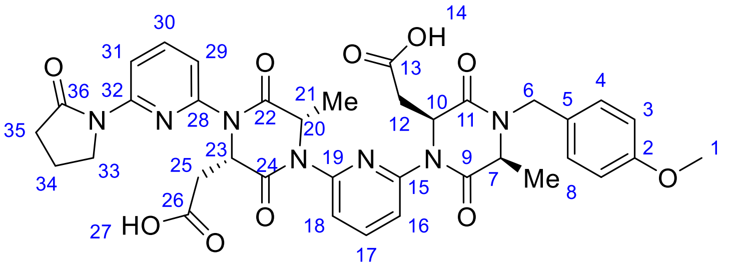

13

^{1}H NMR

400 MHz

d_6-DMSO

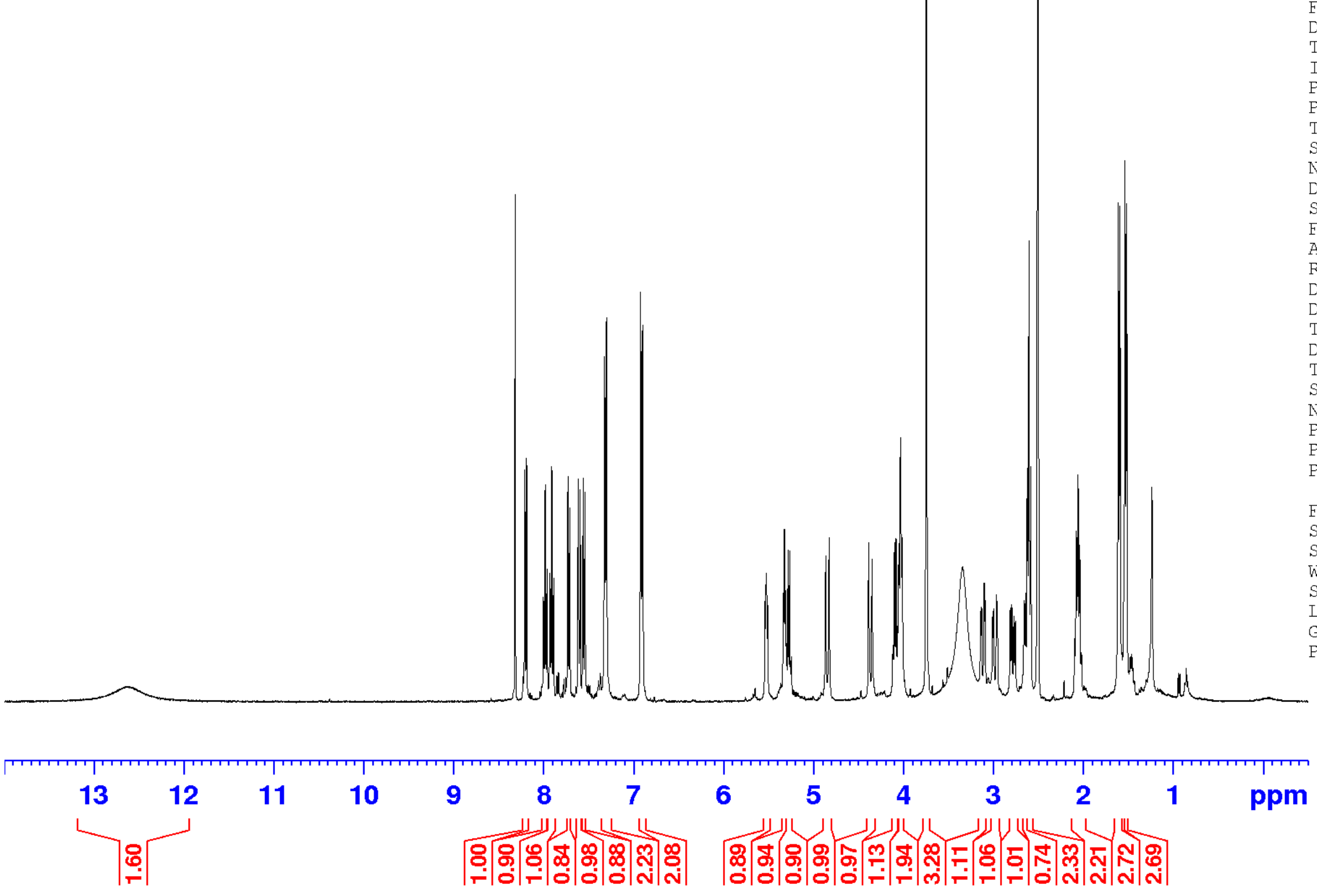


```
Current Data Parameters
NAME      WR 3.441 (DMSO)
EXPNO                  10
PROCNO                  1

F2 - Acquisition Parameters
Date_            20230317
Time               12.06 h
INSTRUM         AVIII_400
PROBHD   Z108618_0146 (
PULPROG              zg30
TD                  65536
SOLVENT              DMSO
NS                     16
DS                      2
SWH              8223.685 Hz
FIDRES           0.250967 Hz
AQ              3.9845889 sec
RG                    181
DW                 60.800 usec
DE                  17.42 usec
TE                  300.0 K
D1             1.00000000 sec
TD0                     1
SFO1          400.1124708 MHz
NUC1                   1H
P0                   5.00 usec
P1                  15.00 usec
PLW1          17.29199982 W

F2 - Processing parameters
SI                  32768
SF            400.1100000 MHz
WDW                    EM
SSB                     0
LB                   0.30 Hz
GB                      0
PC                   1.00
```

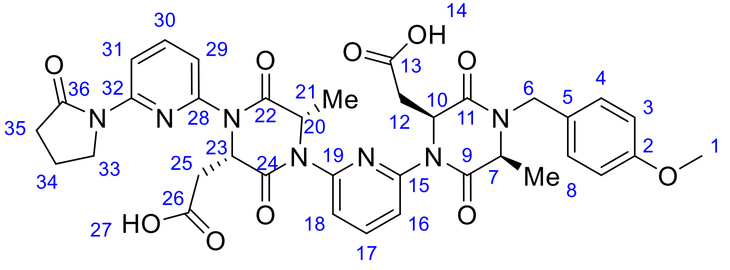

13

^{13}C NMR

101 MHz

d_6-DMSO

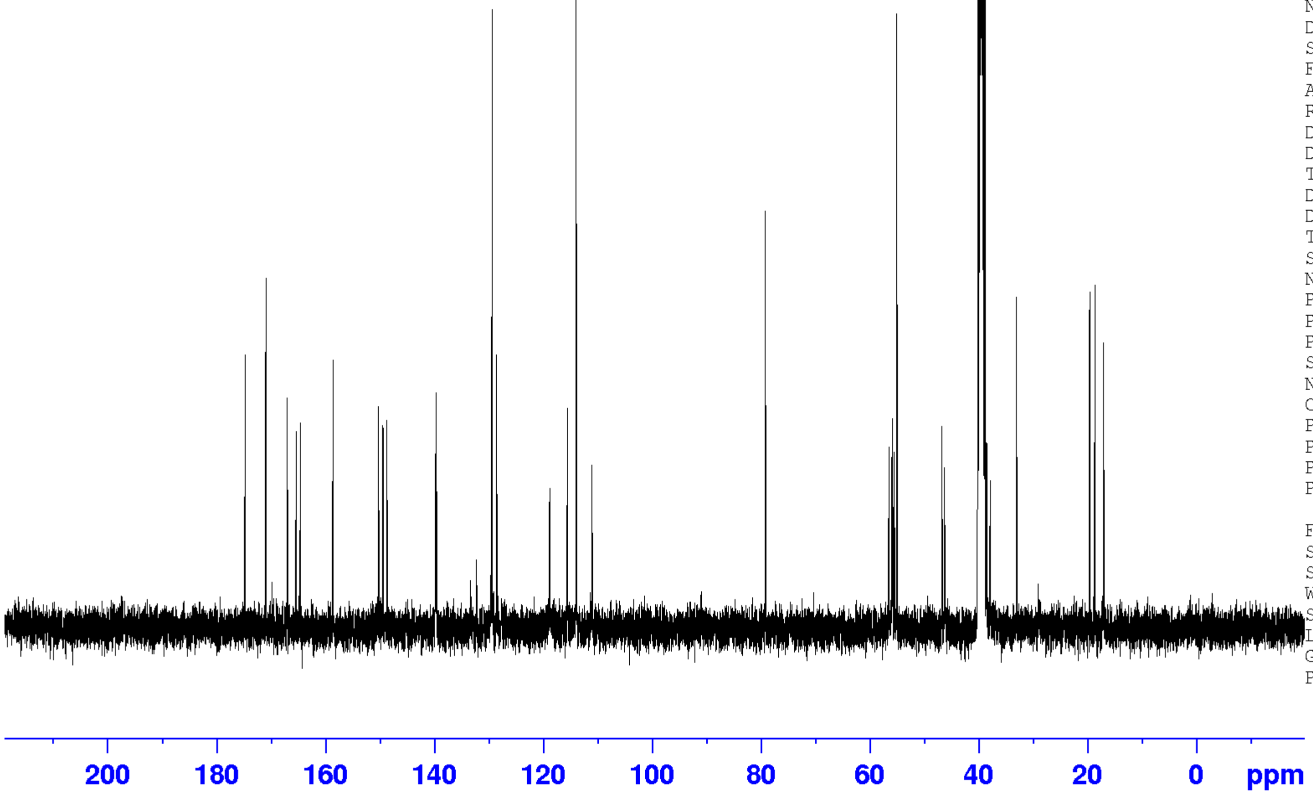


```
Current Data Parameters
NAME      WR 3.441 (DMSO)
EXPNO                  11
PROCNO                  1

F2 - Acquisition Parameters
Date_            20230317
Time                22.49 h
INSTRUM         AVIII_400
PROBHD    Z108618_0146 (
PULPROG           zgpg30
TD                 96150
SOLVENT             DMSO
NS                  3072
DS                     4
SWH            24038.461 Hz
FIDRES          0.500020 Hz
AQ             1.9999200 sec
RG                  2050
DW                20.800 usec
DE                  6.50 usec
TE                 300.0 K
D1            1.00000000 sec
D11           0.03000000 sec
TD0                    1
SFO1         100.6178003 MHz
NUC1                 13C
P0                  2.90 usec
P1                  8.70 usec
PLW1         96.68000031 W
SFO2         400.1116004 MHz
NUC2                  1H
CPDPRG[2         waltz64
PCPD2              90.00 usec
PLW2         17.29199982 W
PLW12         0.48032999 W
PLW13         0.24160001 W

F2 - Processing parameters
SI                131072
SF           100.6077869 MHz
WDW                   EM
SSB                    0
LB                  1.00 Hz
GB                     0
PC                  1.40
```

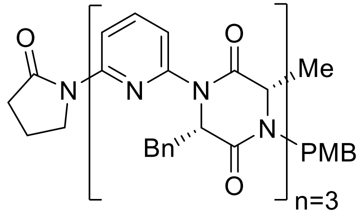

8

^{1}H NMR

600 MHz

$CDCl_3$

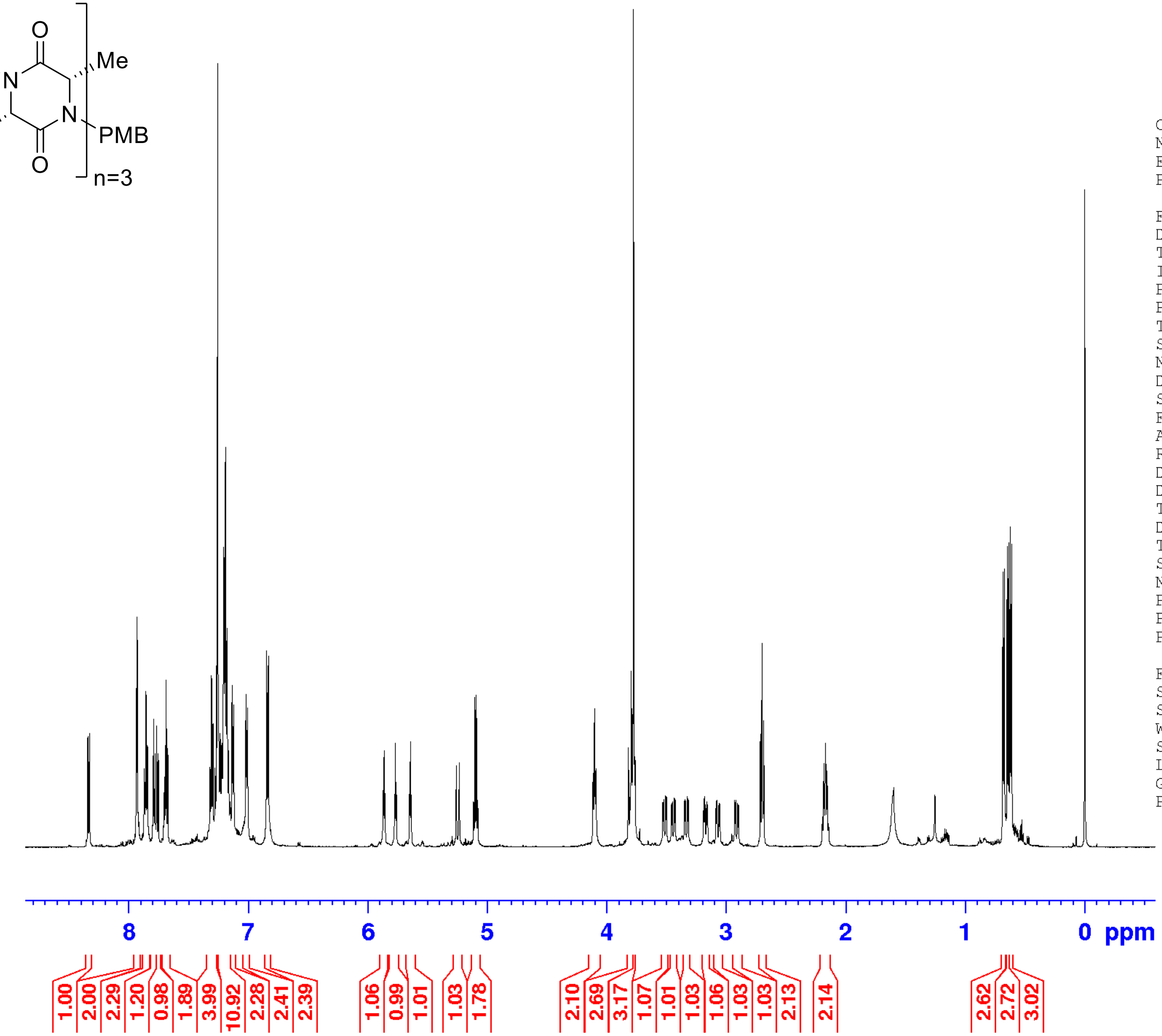


```
Current Data Parameters
NAME              SMC-01-41
EXPNO                    10
PROCNO                    1

F2 - Acquisition Parameters
Date_              20220224
Time                 9.51 h
INSTRUM               spect
PROBHD     Z114607_0188 (
PULPROG                zg30
TD                    65536
SOLVENT               CDCl3
NS                       16
DS                        2
SWH           12019.230 Hz
FIDRES         0.366798 Hz
AQ            2.7262976 sec
RG                     97.5
DW               41.600 usec
DE                12.10 usec
TE                300.0 K
D1           1.00000000 sec
TD0                       1
SFO1        600.1337058 MHz
NUC1                     1H
P0                 3.33 usec
P1                10.00 usec
PLW1        26.60000038 W

F2 - Processing parameters
SI                    65536
SF          600.1300151 MHz
WDW                      EM
SSB                       0
LB                   0.30 Hz
GB                        0
PC                     1.00
```

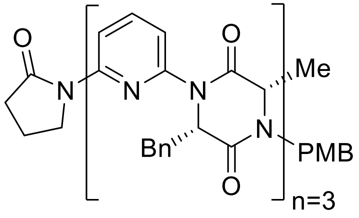

8

^{13}C NMR

151 MHz

$CDCl_3$

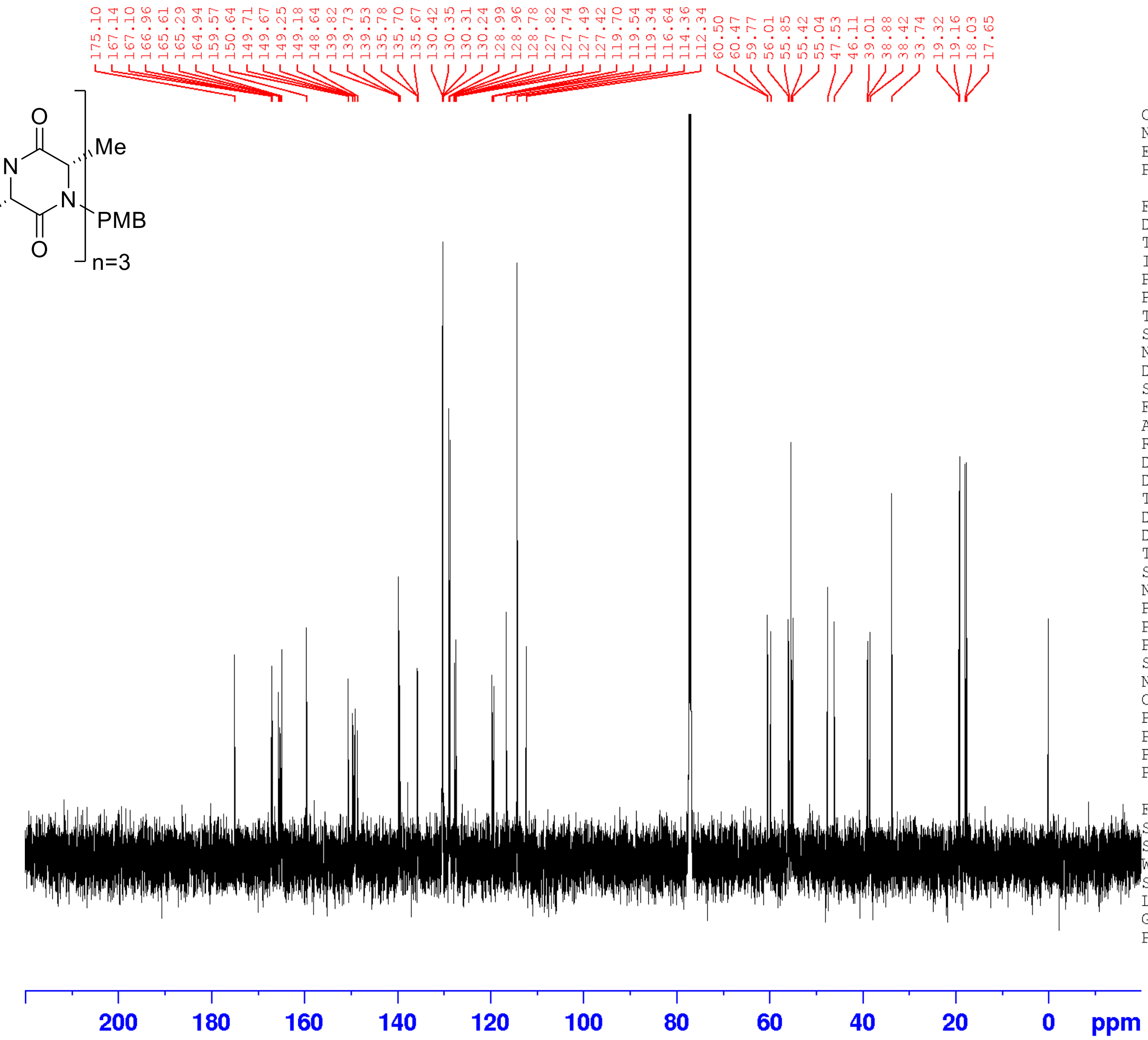

Current Data Parameters
NAME SMC-01-41
EXPNO 11
PROCNO 1

F2 - Acquisition Parameters
Date_ 20220224
Time 10.42 h
INSTRUM spect
PROBHD Z114607_0188 (
PULPROG zgpg30
TD 65536
SOLVENT CDCl3
NS 1024
DS 4
SWH 36231.883 Hz
FIDRES 1.105709 Hz
AQ 0.9043968 sec
RG 186.92
DW 13.800 usec
DE 6.50 usec
TE 300.0 K
D1 2.00000000 sec
D11 0.03000000 sec
TD0 1
SFO1 150.9178988 MHz
NUC1 13C
P0 3.93 usec
P1 11.80 usec
PLW1 85.00000000 W
SFO2 600.1324005 MHz
NUC2 1H
CPDPRG[2 waltz65
PCPD2 70.00 usec
PLW2 27.00000000 W
PLW12 0.57327998 W
PLW13 0.28836000 W

F2 - Processing parameters
SI 32768
SF 150.9027906 MHz
WDW EM
SSB 0
LB 1.00 Hz
GB 0
PC 1.40

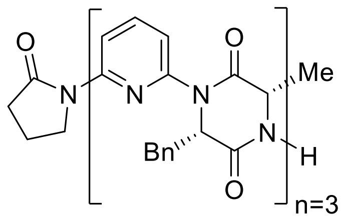

9

^{1}H NMR

600 MHz

$CDCl_3$

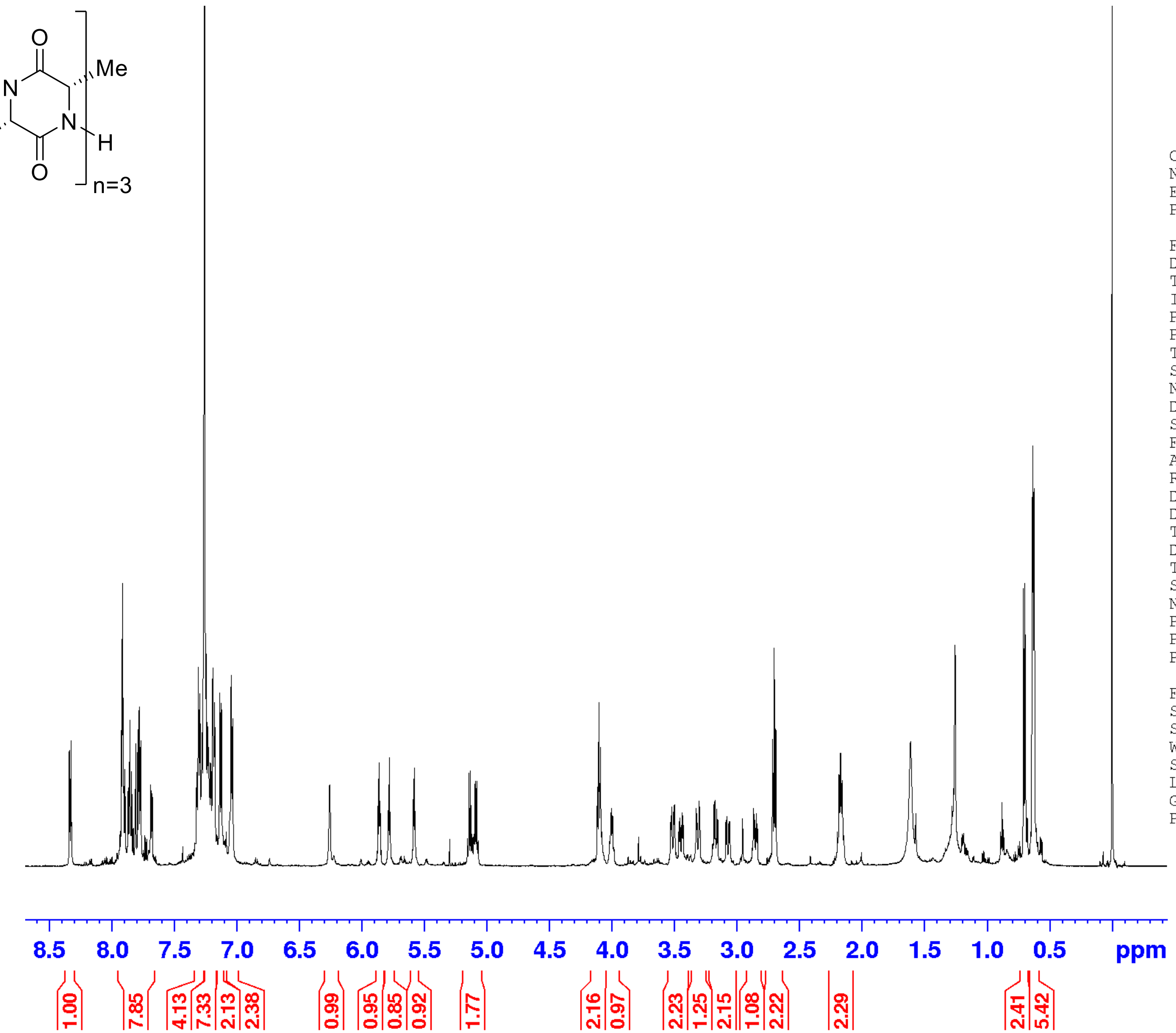


```
Current Data Parameters
NAME        SMC-01-48-600
EXPNO                  10
PROCNO                  1

F2 - Acquisition Parameters
Date_            20220311
Time             18.41 h
INSTRUM             spect
PROBHD    Z114607_0188 (
PULPROG              zg30
TD                  65536
SOLVENT             CDCl3
NS                     16
DS                      2
SWH             12019.230 Hz
FIDRES           0.366798 Hz
AQ              2.7262976 sec
RG                   97.5
DW                 41.600 usec
DE                  12.10 usec
TE                  300.0 K
D1             1.00000000 sec
TD0                     1
SFO1         600.1337058 MHz
NUC1                   1H
P0                   3.33 usec
P1                  10.00 usec
PLW1          26.60000038 W

F2 - Processing parameters
SI                  65536
SF           600.1300148 MHz
WDW                    EM
SSB                     0
LB                   0.30 Hz
GB                      0
PC                   1.00
```

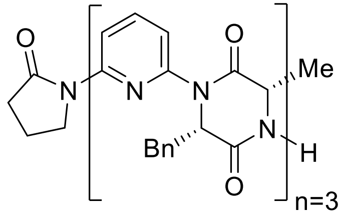

9

^{13}C NMR

151 MHz

$CDCl_3$

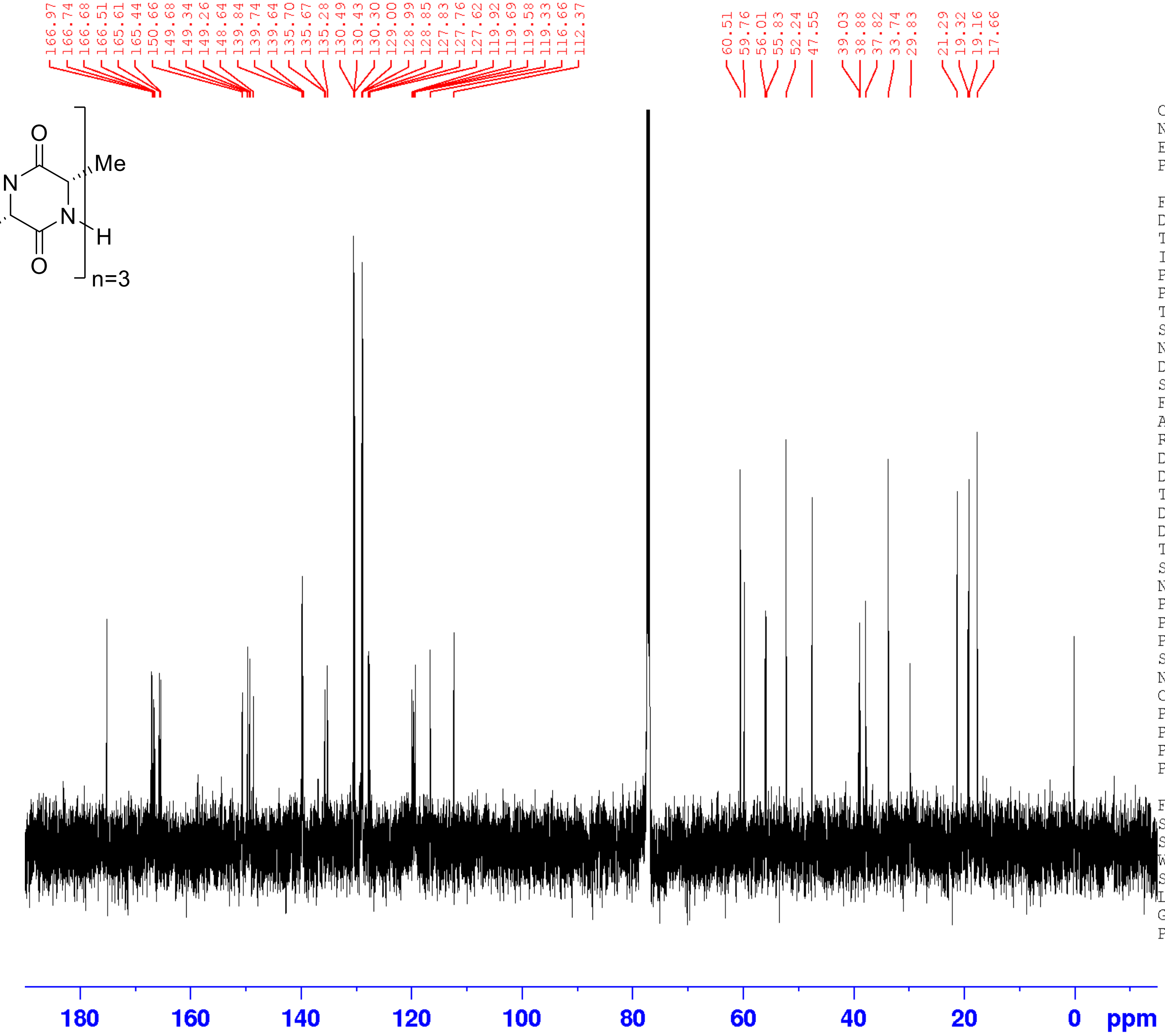

Current Data Parameters
NAME SMC-01-48-600
EXPNO 11
PROCNO 1

F2 - Acquisition Parameters
Date_ 20220311
Time 19.33 h
INSTRUM spect
PROBHD Z114607_0188 (
PULPROG zgpg30
TD 65536
SOLVENT CDCl3
NS 1024
DS 4
SWH 36231.883 Hz
FIDRES 1.105709 Hz
AQ 0.9043968 sec
RG 186.92
DW 13.800 usec
DE 6.50 usec
TE 300.0 K
D1 2.00000000 sec
D11 0.03000000 sec
TD0 1
SFO1 150.9178988 MHz
NUC1 13C
P0 3.93 usec
P1 11.80 usec
PLW1 85.00000000 W
SFO2 600.1324005 MHz
NUC2 1H
CPDPRG[2 waltz65
PCPD2 70.00 usec
PLW2 27.00000000 W
PLW12 0.57327998 W
PLW13 0.28836000 W

F2 - Processing parameters
SI 32768
SF 150.9027888 MHz
WDW EM
SSB 0
LB 1.00 Hz
GB 0
PC 1.40

10 References

1 S. S. Zalesskiy and V. P. Ananikov, *Organometallics*, **2012**, *31*, 2302–2309.

2 J. Cosier and A. M. Glazer, *J Appl Crystallogr*, **1986**, *19*, 105–107.

3 *CrysAlisPRO*, Oxford Diffraction/Agilent Technologies UK Ltd, Yarnton, England.

4 G. M. Sheldrick, *Acta Crystallographica Section A*, **2015**, *71*, 3–8.

5 P. W. Betteridge, J. R. Carruthers, R. I. Cooper, K. Prout and D. J. Watkin, *J Appl Crystallogr*, **2003**, *36*, 1487.

6 R. I. Cooper, A. L. Thompson and D. J. Watkin, *J Appl Crystallogr*, **2008**, *43*, 1100–1107.

7 Z. Lockhart and P. C. Knipe, *Angew. Chem. Int. Ed.*, **2018**, *57*, 8478–8482.

8 Y. K. Tahara, M. Michino, M. Ito, K. S. Kanyiva and T. Shibata, *Chem. Commun.*, **2015**, *51*, 16660–16663.

9 J. Yoshimura, M. Yamaura, T. Suzuki and H. Hashimoto, *Chem. Lett.*, **1983**, *12*, 1001-1002.

10 J. L. Baeza, G. Gerona-Navarro, M. J. P. De Vega, M. T. García-Lopez, R. González-Muñiz and M. Martín-Martínez, *J. Org. Chem.*, **2008**, *73*, 1704–1715.

11 S. Y. Han and Y. D. Gong, *Synth. Commun.*, ***2019***, 49, 3426–3434.

12 E. A. German, J. E. Ross, P. C. Knipe, M. F. Don, S. Thompson and A. D. Hamilton, *Angew. Chem. Int. Ed.*, **2015**, *54*, 2649–2652.

13 P. C. Knipe, S. Thompson and A. D. Hamilton, *Chem. Commun.*, **2016**, *52*, 6521–6524.

14 R. Gopalakrishnan, A. I. Frolov, L. Knerr, W. J. Drury and E. Valeur, *J. Med. Chem.*, **2016**, *59*, 9599–9621.

15 B. A. F. Le Bailly and J. Clayden, *Chem. Commun.*, **2016**, *52*, 4852–4863.

16 L. E. Bickerton, T. G. Johnson, A. Kerckhoffs and M. J. Langton, *Chem Sci*, **2021**, *12*, 11252–11274.

17 P. C. Knipe, S. Thompson and A. D. Hamilton, *Chem. Commun.*, **2016**, *52*, 6521–6524.

18 G. Scorrano, *Acc. Chem. Res.*, **1973**, *6*, 132–138.

19 J. Shang, N. M. Gallagher, F. Bie, Q. Li, Y. Che, Y. Wang and H. Jiang, *J. Org. Chem.*, **2014**, *79*, 5134–5144.

20 S. De, B. Chi, T. Granier, T. Qi, V. Maurizot and I. Huc, *Nat Chem*, **2018**, *10*, 51–57.

21 J. J. Mousseau, L. Xing, N. Tang and L. A. Cuccia, *Chem. Eur. J.*, **2009**, *15*, 10030–10038.

22 E. M. Boyd and J. Sperry, *Org. Lett*, **2014**, *16*, 5056–5059.

23 H. Hammoud, M. Schmitt, E. Blaise, F. Bihel and J. J. Bourguignon, *J. Org. Chem.*, **2013**, *78*, 7930–7937.

24 H. J. Lim, J. C. Gallucci and T. V. Rajanbabu, *Org. Lett.*, **2010**, *12*, 2162–2165.
